# Quaternary geomorphological and climatic changes associated with the diversification of Iberian freshwater fishes: The case of the genus *Cobitis* (Cypriniformes, Cobitidae)

**DOI:** 10.1002/ece3.8635

**Published:** 2022-03-01

**Authors:** Andrea Corral‐Lou, Silvia Perea, Anabel Perdices, Ignacio Doadrio

**Affiliations:** ^1^ Biodiversity and Evolutionary Biology Department Museo Nacional de Ciencias Naturales, CSIC Madrid Spain; ^2^ Consultores en Biología de la Conservación S.L. Madrid Spain; ^3^ 7180 Instituto de Biología Departamento de Zoología Universidad Nacional Autónoma de México Ciudad de México México

**Keywords:** *Cobitis*, hybridization, Iberian Peninsula, population structure and conservation, quaternary

## Abstract

We studied the population genetic structure of *Cobitis vettonica*, an endangered freshwater fish species endemic to the Iberian Peninsula, in order to propose a biogeographic model of the responses of species to the multiple changes that occurred in the Iberian hydrological system during the Quaternary period. We also deciphered the relationship of *C*. *vettonica* with its sister species *C*. *paludica*, particularly in sympatric areas, and provide genetic information for conservation purposes. To achieve this goal, we analyzed both mitochondrial and nuclear data (the *cytochrome b* and the *nuclear recombination activating 1* genes) and a battery of single‐nucleotide polymorphisms (SNPs) of 248 individuals of *C*. *vettonica* or *C*. *paludica* from 38 localities, including some sympatric ones, covering the entire distribution area of *C*. *vettonica*. We highlight the important role played by the hydrogeomorphological processes and climatic changes that occurred in the Iberian Peninsula during the Quaternary on both the population structure of *C*. *vettonica* and its relationship with its sister species *C*. *paludica*. Our results support the genetic introgression of populations at the eastern limit of the distribution of *C*. *vettonica*. Furthermore, we postulate genetic introgression in sympatric areas. Finally, we propose the establishment or expansion of four Operational Conservation Units (OCUs) for *C*. *vettonica*, and highlight the threat faced by its populations due to the low level of genetic diversity detected for some of its populations and genetic introgression with *C*. *paludica*, which could eventually displace *C*. *vettonica*, resulting in a loss of diversity in this species.

## Graphical abstract

We study the population genetics of *Cobitis vettonica* to propose an area‐specific biogeographic model of the species’ responses to the multiple changes that occurred in the Iberian hydrological system during the Quaternary. We also decipher the relationship of *C*. *vettonica* with its sister species *C*. *paludica*, particularly in sympatric areas, and provide genetic information for conservation purposes.

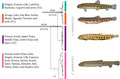

## 1 INTRODUCTION

The Quaternary is a geological period characterized by glacial–interglacial cycles that have dominated the global climate since 2.58 Mya to the present era (Gibbard et al., 2010; Pillans & Naish, 2004), which have had drastic consequences on the evolution of the biota of many regions. Due to advances in the field of phylogeography, we have a better understanding of the responses of organisms to Pleistocene events (Hewitt, 2004; Weiss & Ferrand, 2007). Classical studies support the role of Mediterranean peninsulas as refuges for fauna during the Quaternary, which provided the stock for recolonizations of northern and central Europe (Hewitt, 1996; Taberlet & Bouvet, 1994). The Iberian Peninsula is considered one of the most important refuges during this period, as shown by several studies, mainly of terrestrial fauna (Querejeta et al., 2017; Valdiosera et al., 2008).

Primary freshwater fishes (i.e., fishes strictly intolerant of saltwater; Myers, 1966) have limited dispersal abilities and are often restricted to specific hydrographic basins. Dispersal between basins during the Quaternary was possible mainly through downstream connections caused by the decrease in sea level during cold periods or by upstream piracy (Corral‐Lou et al., 2019; Mesquita et al., 2005; Perea & Doadrio, 2015). The formation of the Iberian fluvial network also culminated during this period, which affected the region's hydrogeomorphology and therefore the current evolutionary patterns found in primary freshwater fauna (Alonso et al., 2007; Corral‐Lou et al., 2021; Pais et al., 2012; Perea et al., 2016). Likewise, the current population structure of many Iberian freshwater fishes has been attributed to the interaction of various natural factors that occurred during the Quaternary such as the drying up of bodies of water, sea‐level fluctuations, tsunamis, stream piracy, isolation of basins, hydrogeomorphological changes, and secondary contact of two different basins (Casal‐López et al., 2017; Corral‐Lou et al., 2019; Gante et al., 2009; Perea et al., 2016).

**FIGURE 1:**
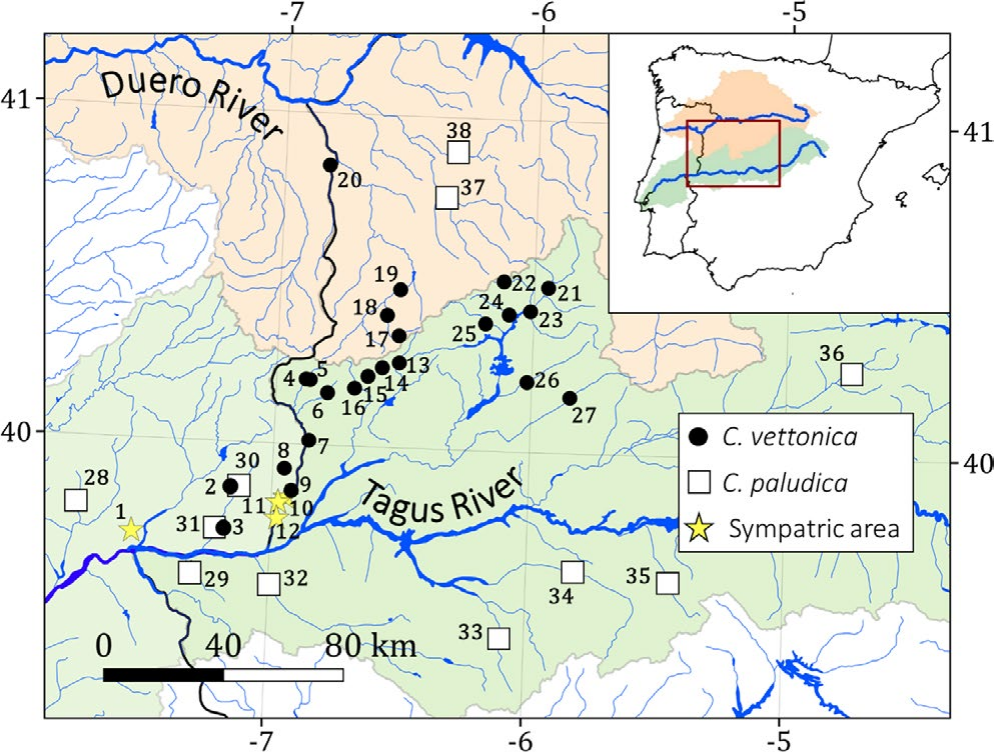
Sampling localities included in this study. The orange shading delimits the Duero Basin, and the green shading delimits the Tagus Basin. Numbers correspond to those listed in Table 1

Despite efforts made in the last several decades, there is still much to be learned about the impact of Quaternary changes on the evolutionary processes and patterns of diversification of Iberian freshwater fish populations. Especially those species with a restricted distribution range since they were probably more affected by climatic and geological changes during the Quaternary than species with larger ranges. In order to address some open evolutionary questions, such as the role of hydrogeomorphologic changes and genetic introgression in species evolution, we analyzed populations of *Cobitis vettonica* as a case study. This species is an endangered Iberian freshwater fish whose distribution is restricted to a few rivers in the Tagus and Duero basins in the mid‐western Iberian Peninsula (Figure 1). It inhabits rivers with low pH and water hardness levels, and gravel and rocky bottoms, and is more commonly found in the headwaters of these rivers (Carmona et al., 1999; Collares‐Pereira et al., 2021; Doadrio et al., 2011; Perdices & Coelho, 2020). In contrast, the sister species of *C*. *vettonica*, *Cobitis paludica* (Doadrio & Perdices, 2005; Perdices & Doadrio, 2001), is a generalist that inhabits most of the Iberian drainages including Tagus and Douro, with a preference for streams close to the main channel with high suspended solids, high water hardness, low transparency, low current velocity and muddy bottoms (Carmona et al., 1999; Doadrio et al., 2011). Sympatric zones of *C*. *vettonica* and *C*. *paludica* have been reported in the western limits of the distribution area of *C*. *vettonica* but genetic introgression between them has not been reported (Perdices & Coelho, 2020). However, in some eastern populations there has been mention of genetic introgression between *C*. *vettonica* and *C*. *paludica*, but no more information has been detailed (Doadrio et al., 2011, 2021). Sister species of Iberian freshwater fishes generally have an allopatric distribution that was established mainly before the Quaternary (Doadrio, 1988; Sousa‐Santos et al., 2019). Some species now show patterns of sympatry in certain areas as a consequence of secondary contacts during the Quaternary, which has led to genetic introgression, as is the case of some species within the genera of *Luciobarbus* and *Phoxinus* (Corral‐Lou et al., 2019; Denys et al., 2013; Machordom et al., 1990). In the case of *C*. *vettonica* and *C*. *paludica*, phylogenetic studies have shown they diverged during the Plio‐Pleistocene (Doadrio & Perdices, 2005; Sousa‐Santos et al., 2014). However, both the origin of the sympatric zones between *C*. *vettonica* and *C*. *paludica* and the plausible genetic introgression between them in the western distribution area of *C*. *vettonica* are unknown. All these points make fishes with restricted distribution areas as excellent models to decipher the Quaternary effects on the diversification of freshwater‐restricted taxa within a glacial refuge as the Iberian Peninsula (Gante et al., 2009).

Phylogeographic studies of Iberian primary freshwater fishes have mainly used mitochondrial genes combined with nuclear markers such as microsatellites or introns (Casal‐López & Doadrio, 2018; Corral‐Lou et al., 2019; Gonzalez et al., 2014, 2018; Perea & Doadrio, 2015). In recent years, phylogeographic and population genetics studies have taken advantage of next‐generation sequencing (NGS) technologies to broadly screen the genome at high resolution, yet some challenges remain in the analysis of NGS data such as the handling of large‐scale and complex data, the upstream process of pipeline, and the limited number of available reference genomes (McCormack et al., 2013; Tan et al., 2019). Combined analyses of single‐nucleotide polymorphisms (SNPs) with mitochondrial and nuclear markers have proven useful to provide more complete and more detailed phylogeographic and biogeographic models of the past and present relationships of populations of various species (Corral‐Lou et al., 2021; Mendes et al., 2019; Zarraonaindia et al., 2012).

The main aim of this study is to provide a robust biogeographic model for the species *C*. *vettonica* throughout its entire distribution as a witness of the evolution of the Iberian Peninsula throughout the Quaternary. We also decipher the relationship of *C*. *vettonica* with its sister species *C*. *paludica* at the limits of its distribution area. In addition, we assess the population structure and genetic diversity of the species across its distribution to revise the Operational Conservation Units (OCUs; Doadrio et al., 1996) previously established for *C*. *vettonica* (Doadrio et al., 2021) or to establish new ones. More detailed and accurate OCUs and deciphering the relationship with its sister species throughout its entire range are key for the effective management and conservation of the endangered *C*. *vettonica*. To achieve these goals, we analyzed the mitochondrial marker *cytochrome b* (*MT*‐*CYB*), the nuclear marker *recombination activating gene 1* (*RAG1*), and a set of SNPs obtained through next‐generation sequencing of populations throughout the entire distribution range of *C*. *vettonica* and some of *C*. *paludica* from adjacent sub‐basins.

## 2 MATERIALS AND METHODS

### 2.1 Sampling, DNA extraction, amplification and sequencing

We sampled 204 individuals of *C*. *vettonica* and 60 of *C*. *paludica* from a total of 38 localities (Figure 1; Table 1). The 27 localities (23 in the Tagus and 4 in the Duero) in which *C*. *vettonica* was found cover the entire known distribution area of the species, and includes its type locality (Árrago River, Tagus Basin). The samples of *C*. *paludica* were collected from 13 localities, including its type locality (Tiétar River, Tagus Basin): of these, 11 were either adjacent to those of *C*. *vettonica* or in sympatric localities in the Tagus Basin, and two were in the Duero Basin (Figure 1; Table 1). Tissue samples were obtained from the DNA and Tissue Collection at the National Museum of Natural Sciences of Madrid (MNCN–CSIC; Table S1). Sequences of *MT*‐*CYB* of *C*. *vettonica* and *C*. *paludica* available in GenBank were also included in the study (33 of *C*. *vettonica* and 7 of *C*. *paludica*; Table S1).

**TABLE 1:** Information on the sampling localities included in the present study

No	Species	River	Basin	Sub‐basin	Locality	Province	mtDNA	nDNA	SNPs
1	Sympatric area	Alfrividas	Tagus	Ponsul	Alfrividas	Portugal	4/1	–	–
2	*C. vettonica*	Aravil	Tagus	Aravil	Alcafozes	Portugal	1	–	–
3	*C. vettonica*	Aravil	Tagus	Aravil	Cegonhas Novas	Portugal	4	–	–
4	*C. vettonica*	Erjas	Tagus	Upper Erjas	Valverde del Fresno	Spain	12	9?	5
5	*C. vettonica*	San Martin	Tagus	Upper Erjas	San Martín de Trevejo	Spain	6	1	–
6	*C. vettonica*	Trevejana	Tagus	Upper Erjas	Cilleros	Spain	4	4	–
7	*C. vettonica*	Erjas	Tagus	Middle Erjas	Termas de Monfortinho	Portugal	6	–	–
8	*C. vettonica*	Arades	Tagus	Middle Erjas	Salvaterra do Extremo	Portugal	2	–	–
9	*C. vettonica*	Erjas	Tagus	Lower Erjas	Salvaterra do Extremo	Portugal	2	–	–
10	Sympatric area	Erjas	Tagus	Lower Erjas	Cabeça Queimado	Portugal	1/1	–	–
11	Sympatric area	Erjas	Tagus	Lower Erjas	Azenha do Roque	Portugal	4/1	–	–
12	Sympatric area	Erjas	Tagus	Lower Erjas	Serrinha	Portugal	2/1	–	–
13	*C. vettonica*	Árrago	Tagus	Western Alagón	Cadalso de Gata	Spain	15	3?	–
14	*C. vettonica*	Gata	Tagus	Western Alagón	Gata	Spain	19	5	5
15	*C. vettonica*	San Blas	Tagus	Western Alagón	Gata	Spain	1	–	–
16	*C. vettonica*	Acebo	Tagus	Western Alagón	Hoyos	Spain	3	1	–
17	*C. vettonica*	Mayas	Duero	Águeda	Descargamaría	Spain	2	–	–
18	*C. vettonica*	Mayas	Duero	Águeda	El Sahugo	Spain	20	4	10
19	*C. vettonica*	Águeda	Duero	Águeda	La Herguijuela	Spain	1	–	–
20	*C. vettonica*	Turones	Duero	Águeda	La Bouza	Spain	1	1	–
21	*C. vettonica*	Alagón	Tagus	Eastern Alagón	Santi Ibañez de la Sierra	Spain	14	9	4
22	*C. vettonica*	Francia	Tagus	Eastern Alagón	Nava de Francia	Spain	14	5	8
23	*C. vettonica*	Cuerpo de Hombre	Tagus	Eastern Alagón	Sotoserrano	Spain	19	9	10
24	*C. vettonica*	Ladrillar	Tagus	Eastern Alagón	La Rebollosa	Spain	1	–	–
25	*C. vettonica*	Hurdano	Tagus	Eastern Alagón	Vegas de Coria	Spain	1	1	–
26	*C. vettonica*	Caparro	Tagus	Eastern Alagón	Zarza de Granadilla	Spain	23	7	8
27	*C. vettonica*	Jerte	Tagus	Eastern Alagón	Navaconcejo	Spain	15	5	10
28	*C. paludica*	Alvito	Tagus	Ocreza	Monte Gordo	Portugal	1	–	–
29	*C. paludica*	Aurela	Tagus	Salor	Santiago de Alcántara	Spain	5	5	9
30	*C. paludica*	Toula	Tagus	Aravil	Alcafozes	Portugal	1	–	–
31	*C. paludica*	Aravil	Tagus	Aravil	Cegonhas Novas	Portugal	1	–	–
32	*C. paludica*	Salor	Tagus	Salor	Membrío	Spain	2	–	2
33	*C. paludica*	Tamuja	Tagus	Almonte	Trujillo	Spain	4	–	4
34	*C. paludica*	Almonte	Tagus	Almonte	Jaraicejo	Spain	5	5	5
35	*C. paludica*	Ibor	Tagus	Lower Tagus	Castañar de Ibor	Spain	3	–	3
36	*C. paludica*	Tiétar	Tagus	Tiétar	La Iglesuela del Tiétar	Spain	11	5	6
37	*C. paludica*	Yeltes	Duero	Huebra‐Yeltes	San Martín de Yeltes	Spain	5	5	8
38	*C. paludica*	Huebra	Duero	Huebra‐Yeltes	El cubo de don Sancho	Spain	9	4	9

Notes

Also detailed are the number of individuals studied for each of the marker types: mtDNA (*MT*‐*CYB*), nDNA (*RAG1*), and SNPs. Under the mtDNA column, for each sympatric area, the two numbers indicate the number of individuals analyzed for *C*. *vettonica* and *C*. *paludica*, respectively.

For each individual, DNA was extracted from ventral fin tissue using the Qiagen DNeasy^®^ Blood and Tissue Kit (Qiagen, Inc., Valencia, CA, USA), following the manufacturer's protocol. Polymerase chain reaction (PCR) was used to amplify 1140 bp of *MT*‐*CYB* from 208 individuals (164 of *C*. *vettonica* and 44 of *C*. *paludica*), and 1500 bp of *RAG1* from 90 individuals (66 of *C*. *vettonica* and 24 of *C*. *paludica*) (Table S1). Amplifications were performed following the protocol described by Doadrio and Perdices (2005) for *MT*‐*CYB*, and Corral‐Lou et al. (2021) for *RAG1*.

All sequences (the new ones and the ones downloaded from GenBank; Table S1) for *MT*‐*CYB* (248 sequences, 240 from the present study and 40 from GenBank; Table S1) and *RAG1* (90 sequences from the present study; Table S1) were aligned using MAFFT (Katoh & Standley, 2013), as implemented in Geneious 10.1.3 (https://www.geneious.com; Kearse et al., 2012), and then manually examined. Alleles of *RAG1* were separated using the PHASE algorithm (Stephens & Donnelly, 2003), as implemented in DnaSP v. 6.10.01 (Rozas et al., 2017).

### 2.2 Genotyping and SNP filtering

For the SNP study, we selected eight populations of each *C*. *vettonica* and *C*. *paludica* (Table 1) based on the structure observed in a previous study using mitochondrial data and available DNA (Doadrio et al., 2021). A total of 106 individuals (60 of *C*. *vettonica* and 46 of *C*. *paludica*; Table S1) were used to prepare libraries for double digest restriction site‐associated DNA sequencing (ddRAD‐seq) following the protocol described by Kess et al. (2016). All DNA samples were used as an input for a custom library preparation protocol. Libraries were dual‐indexed for postsequencing demultiplexing. The samples were run in a NovaSeq 6000 PE150 lane. Trimmomatic 0.36 (Bolger et al., 2014) was used to remove adapters (ILLUMINACLIP option). Using the *process_radtags* program in STACKS 2.4 (Catchen et al., 2013), all reads were truncated to the same length of 95 bp, and low‐quality reads were removed using the ‐*q* parameter according to *phred33* system. A de *novo_map* analysis was also performed in STACKS, in which different programs were run to assemble loci in each individual (*ustacks*), build a catalogue (*cstacks*), match all generic samples against the catalogue (*sstacks*), and reconstruct loci using R2 reads and identify SNPs using the metapopulation information (*gstacks*). Prior to running the STACKS modules, several tests were used to identify which parameters maximized the number of SNPs obtained in at least 80% of the individuals (r80 rule; Paris et al., 2017). Since SNPs were obtained for both species, we explored parameters in a conservative way in the *de_novo* maps module of stacks. The parameter *m* (i.e., the minimum depth of coverage required to create a stack) was set to 5 due to the depth of coverage values obtained. The *M* parameter (i.e., number of mismatches allowed between stacks within individuals) has to be carefully set. If it is set too high, paralogous or nonhomologous loci can be incorrectly merged into the same locus and if it is set too low, homologous loci can be lost. For this reason, the parameter *M* was explored between 3 and 5. Following the indications of Paris et al. (2017), the values of *n* (i.e., the number of mismatches allowed between stacks between individuals) were explored from 2 to 4 (Table S2). Finally, the selected parameters were *m* = 5, *M* = 3, and *n* = 2. The program *populations*, also in STACKS, was used to filter the SNPs using the following parameters: ‐*p* 16 ‐*r* 0.80, *–min_maf* = 0.05, *–max_obs_het* = 0.75, and *–write_random_snp*.

Two matrices for the SNPs were built. The allowed percentage of missing data per locus and per individual was 30% (Table S3). The first matrix included the populations of *C*. *vettonica* and data from 60 individuals from 8 localities and 4000 polymorphic loci. The second included populations of both *C*. *vettonica* and *C*. *paludica*, referred to as *C*. *vettonica* + *C. paludica*, and data from 106 individuals from 16 localities and 4538 polymorphic loci.

### 2.3 Phylogeny

A phylogenetic tree was constructed to assess relationships based on the collapsed *MT*‐*CYB* haplotypes matrix. The selected substitution models were SYM, HKY + I, and GTR + G for the first, second, and third position, respectively, based on the results obtained in PartitionFinder2 (Lanfear et al., 2017) using the Akaike information criterion (AIC; Akaike, 1974). The analysis was implemented in MrBayes v3.2 (Ronquist et al., 2012), with two simultaneous independent runs each with four Markov chain Monte Carlo (MCMC), which were run for 5 × 10^7^ generations. The first 25% of generations were removed as burn‐in. Posterior probability (pp) values were used to assess the reliability of the phylogenetic hypothesis. Two sequences of other species of *Cobitis* were used as outgroups: *C*. *bilineata* (EF605321.1) and *C*. *zanandreai* (AF263089.1). The genetic divergence between the lineages obtained in the *MT*‐*CYB* analysis was evaluated through uncorrected *p*‐distances with 1000 permutations using MEGA v.6.0 (Tamura et al., 2013). For the SNP data, phylogenetic relationships were evaluated using the complete SNP matrix (*C*. *vettonica* + *C. paludica*) through the Maximum Likelihood (ML) method in the RaxML program implemented in CIPRES Science Gateway v3.3. The evolutionary model selected was ASC_GTRGAMMA, as recommended in the program manual for data that contain only variable sites, and the Lewis ascertainment bias correction was used (Stamatakis, 2016). The robustness of the tree was evaluated with 1000 bootstrap (b) replicates.

### 2.4 Genetic structure

To examine the genetic structure of all studied populations of *C*. *vettonica* and *C*. *paludica*, haplotype networks for the two genes were constructed using the median‐joining algorithm (Bandelt et al., 1999) implemented in PopART (Leigh & Bryant, 2015). Analysis of molecular variance (AMOVA) was used to determine the source of the genetic variation in *MT*‐*CYB* for the populations identified as *C*. *vettonica* (excluding V4 subset, see below) using different groupings based on basin, sub‐basin, and evolutionary lineage. These analyses were implemented in Arlequin v.3.11 (Excoffier & Lischer, 2010) with 10,000 permutations. Population differentiation in terms of Φst (Hudson et al., 1992) between all population pairs (except those represented by only one individual) for the complete dataset (*C*. *vettonica* and *C*. *paludica*) was also calculated in Arlequin v.3.11. For the localities in which mitochondrial haplotypes of both *C*. *vettonica* and *C*. *paludica* were found, the *Φ*_st_ was calculated by treating the individuals of each species as separate populations.

For the SNPs data, only neutral loci were taken into account to study the population structure in order to avoid the bias that could be caused by candidate loci for selection. For this reason, we evaluated the presence of loci under selection with BayeScan v.2.0 (Foll & Gaggiotti, 2008), using the default parameters except for the prior odds (prior odds = 100; Lotterhos & Whitlock, 2014). We removed the loci under selection and constructed two new matrices for the neutral loci, keeping 3998 loci in the *C*. *vettonica* matrix and 4500 loci in the *C*. *vettonica* + *C. paludica* matrix. STRUCTURE (Pritchard et al., 2000) was used to assess the genetic structure based on the two SNP matrices. The most probable number of subpopulations (*K*) for each analysis was equal to the number of populations studied + 1 (*K* = 9 and *K* = 17, respectively). We performed 10 independent simulations for each *K* with a burn‐in length of 50,000 and 50,000 MCMC repetitions after the burn‐in. The most probable number of subpopulations for each matrix was estimated by taking into account the results of both the Δ*K* (Evanno et al., 2005) and the Puechmaille method (Puechmaille, 2016). The web server StructureSelector (Li & Liu, 2018) was used to make these estimations. We selected *K* based on both methods because a greater probability of *K* = 2 exists when the structure is analyzed with the Δ*K* method (Janes et al., 2017), and similar results for different methods indicate a clear signal (Puechmaille, 2016).

### 2.5 Genetic diversity and demography

Different genetic diversity parameters were estimated for both *MT*‐*CYB* and *RAG1* in DnaSP for each river, sub‐basin, species, evolutionary lineage, and OCUs. The studied genetic diversity parameters were haplotype or nuclear alleles number (*h* or *a* for *MT*‐*CYB* and *RAG1*, respectively), haplotype or allelic diversity (*Hd* or *a_d_* for *MT*‐*CYB* and *RAG1*, respectively), nucleotide diversity (*π*), and number of polymorphic sites (*S*). For *MT*‐*CYB*, the populations with shared haplotypes between *C*. *vettonica* and *C*. *paludica* were analyzed considering only the individuals identified as *C*. *vettonica*. For the SNP data, a different set of genetic diversity parameters was estimated using the *populations* program in STACKS 2.4 (Catchen et al., 2013). These parameters were evaluated for the two matrices independently (*C*. *vettonica* and *C*. *vettonica* + *C. paludica*); however, only the results for *C*. *vettonica* are shown. In addition, a third matrix was studied taking into account the populations of both species, except the populations of the Aurela and Salor rivers of *C*. *paludica*, due to the close relationship that these localities showed with the localities of the Erjas sub‐basin of *C*. *vettonica*.

To evaluate the demography of populations of *C*. *vettonica*, deviations from a model of mutation‐drift equilibrium for *MT*‐*CYB* were tested using Fu's Fs (Fu, 1997) and Tajima's *D* (Tajima, 1989), as implemented in Arlequin v.3.11.

### 2.6 Divergence times and niche modeling

Divergence times among the populations of *C*. *vettonica* were estimated using a relaxed lognormal clock and a coalescent model on the collapsed *MT*‐*CYB* haplotypes matrix in BEAST v 1.8.4 (Drummond et al., 2012). The molecular clock was calibrated using, as a normal prior, an evolutionary rate of 0.34% divergence per lineage per million years (Doadrio & Perdices, 2005), and the substitution model used was GTR + I.

To identify the potential niche of *C*. *vettonica* in both past and present scenarios, ecological niche models, as applied in the software MaxEnt v.3.4.1 (Phillips et al., 2017), were used to predict a mean habitat suitability value for each locality. To select the most appropriate of the 19 accessible bioclimatic variables downloaded from the WorldClim dataset (Fick & Hijmans, 2017) for the final analysis, correlation and collinearity analyses and preliminary analyses were first performed. Three variables based on the ecological characteristics of the species were chosen for the final analysis: Isothermality (BIO3), Precipitation of Driest Month (BIO14), and Precipitation Seasonality (BIO15). The data matrix on the presence of the species included only the studied populations of *C*. *vettonica* that do not have any putative genetic introgression with *C*. *paludica*. The niche modeling was made for three periods of time: Present (the years 1970–2000), Last Glacial Maximum (LGM; ~22,000 years before present, BP), and Last Interglacial (LIG; ~120,000–140,000 BP). The quality of the model was evaluated by the Area Under the Curve (AUC) derived from the Receiver Operating Characteristic (ROC).

### 2.7 Ancestral area reconstructions

The ancestral areas of *C*. *vettonica* were reconstructed using both the S‐DIVA (Yu et al., 2010) and S‐DEC (Beaulieu et al., 2013; Ree & Smith, 2008) methods implemented in RASP v.4.2 (Yu et al., 2015, 2020). The established areas were based on the current hydrographic subdivision of the Iberian Peninsula. Due to the genetic differentiation detected for *MT*‐*CYB* in the Alagón sub‐basin, this area was divided into an eastern and a western part. The code for the areas was as follows: A: Águeda, B: Western Alagón, C: Eastern Alagón, D: Aravil, E: Erjas, F: Ponsul, G: Salor, H: Ocreza, I: Aurela, J: Huebra and Yeltes, K: Tamuja and Almonte, L: Ibor, and M: Tiétar. We used the BEAST‐derived trees as the input files for RASP. We eliminated 50% of the total initial trees, and used 100 random trees for the analysis. Ancestral ranges were limited to include no more than three adjacent areas. Only the reconstructed areas for *C*. *vettonica* are shown as this lineage is the focus of the present study.

## 3 RESULTS

### 3.1 Phylogeny

**FIGURE 2:**
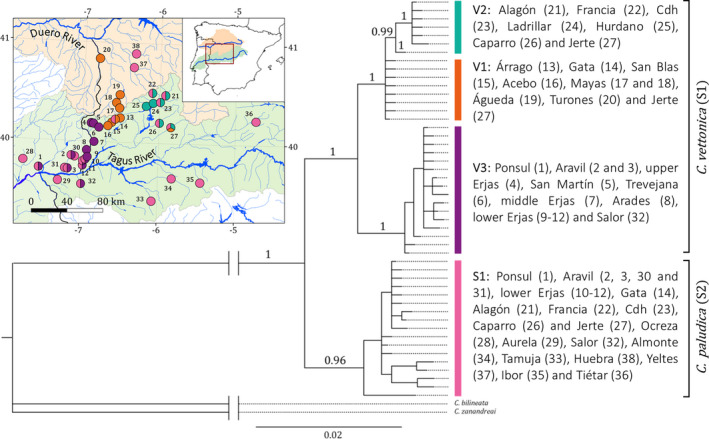
Phylogenetic tree based on Bayesian inference for the mitochondrial marker *MT*‐*CYB*. Posterior probability values are indicated above branches. S1: *Cobitis vettonica*. The vertical colored bars represent the three main lineages (V1–V3) of the species and their localities (see legend). S2: *Cobitis paludica*. Also, the map is shown indicating the sampling locations referenced in Table 1. The color of the sampling point indicates the detected lineage in each population based on the results of the phylogenetic tree

The phylogenetic analysis of *MT*‐*CYB* recovered two main lineages corresponding to the species *C*. *vettonica* (S1) and *C*. *paludica* (S2) with high support (pp = 1; Figure 2). The species *C*. *vettonica* (S1) was not monophyletic since many individuals identified morphologically as *C*. *vettonica* from the Alagón sub‐basin populations were found in both lineages (i.e., Jerte, Francia, Alagón, Caparro, Cuerpo de Hombre [Cdh], and Gata rivers). The lineage of *C*. *paludica* (S2) was also not a monophyletic group since one individual identified morphologically as *C*. *paludica* from the Salor sub‐basin was included in the *C*. *vettonica* lineage (S1). Within *C*. *vettonica* (S1), there were two well‐differentiated lineages (Figure 2). The first grouped populations from the Alagón sub‐basin in the Tagus Basin (Árrago, Gata, San Blas, Acebo, Alagón, Francia Cdh, Ladrillar, Hurdano, Caparro, and Jerte rivers) and those from the Águeda sub‐basin in the Duero Basin (Turones, Mayas, and Águeda rivers). Within this lineage, these populations grouped into two distinct lineages, V1 and V2. The populations from the eastern Alagón sub‐basin (Alagón, Francia, Cdh, Ladrillar, Hurdano, Caparro, and Jerte rivers) formed the V2 lineage (pp = 0.99), while those from the western Alagón (Árrago, Gata, San Blas, and Acebo rivers) and Águeda sub‐basins (Turones, Mayas, and Águeda rivers), and one individual from Jerte River (from eastern Alagón sub‐basin) that was not included in V2, formed the V1 lineage. The phylogenetic relationships of the sequences included in V1 were not resolved. However, within V2, we detected another highly supported monophyletic grouping (pp = 1) in which only the single individual studied from Ladrillar River was excluded. The second well‐differentiated lineage within *C*. *vettonica* (V3; pp = 1) included populations from the Ponsul, Aravil, and Erjas sub‐basins located along the western limit of the species’ distribution range, and one individual from Salor River (Figure 2).

Genetic distances among the three lineages detected in *C*. *vettonica* (V1, V2, and V3) ranged from 0.3% to 1.2% (Table 2).

**TABLE 2:** Uncorrected absolute genetic distances between the main lineages of *C*. *vettonica* (V1, V2, and V3) and *C*. *paludica* (S1). The standard deviation is shown on the upper diagonal

	V1	V2	V3	S2
V1		0.002	0.003	0.005
V2	0.003		0.003	0.004
V3	0.011	0.012		0.005
S2	0.022	0.025	0.026	

**FIGURE 3:**
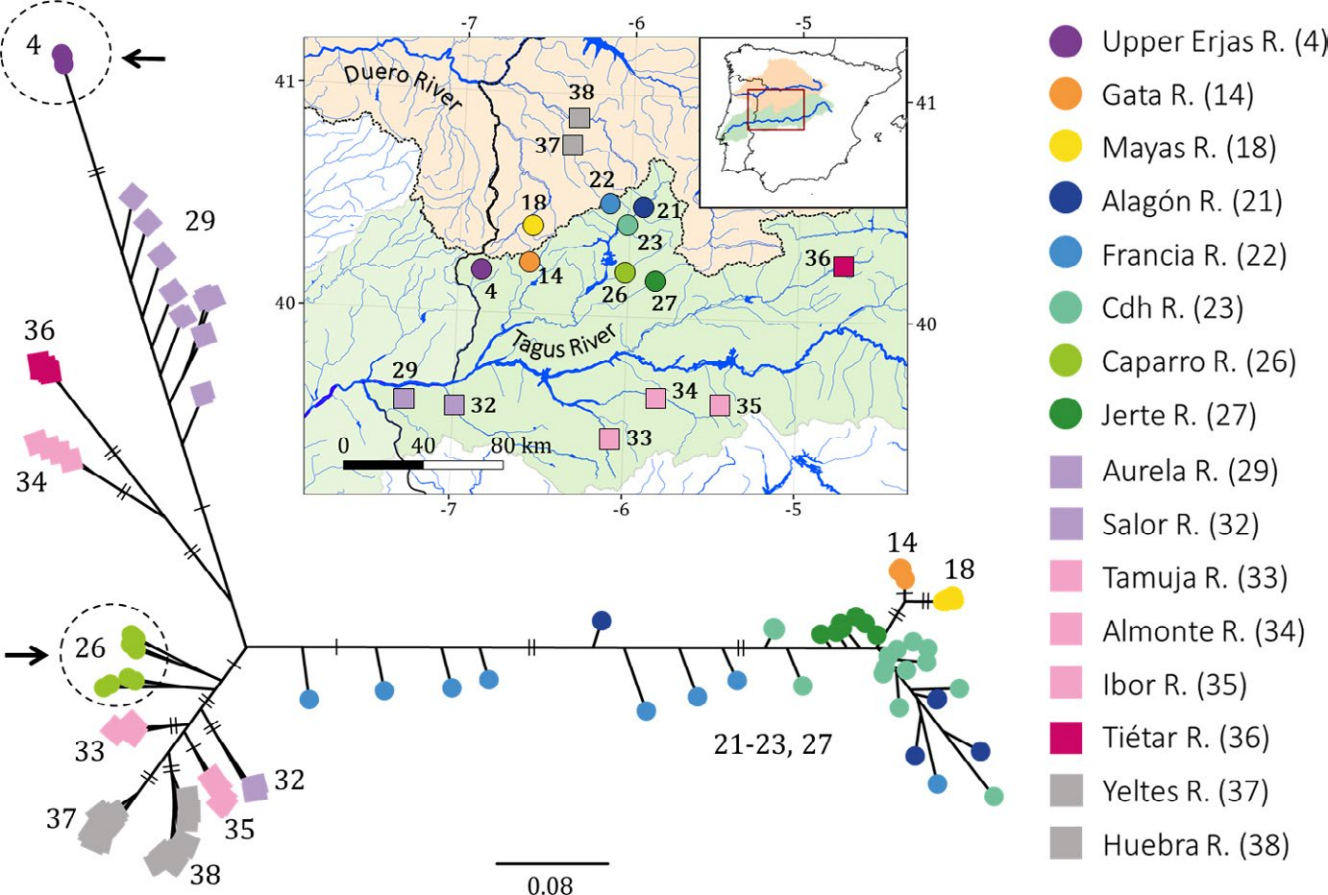
Unrooted ML phylogenetic tree based on the 4538 SNP loci. Circles and squares indicate individuals morphologically identified as *C*. *vettonica* and *C*. *paludica*, respectively. The numbers refer to those assigned to the populations in Table 1. The bootstrap values (b) are represented with a line perpendicular to the branch for 75 < b > 90, and two lines for b > 90. The arrows highlight the populations of *C*. *vettonica* from the Caparro (→) and upper Erjas (←) rivers. Abbreviation: R. for river. Also, the map is shown indicating the sampling locations referenced in Table 1

The unrooted phylogenetic tree of *C*. *vettonica* and *C*. *paludica* based on the 4538 SNPs was largely congruent with the results obtained in the phylogenetic reconstruction for *MT*‐*CYB*, although with some slight differences (Figure 3). The individuals of the populations analyzed for SNPs belonging to the V1 lineage (i.e., Mayas from Águeda sub‐basin, Duero Basin and Gata from western Alagón sub‐basin, Tagus Basin) were well differentiated not only from the rest of the individuals analyzed (b > 90) but also from each other (b > 90 and 75 < b > 90, respectively). Those belonging to the V3 lineage (Erjas River), which were separated from the rest of the populations of *C*. *vettonica*, were more related to individuals of *C*. *paludica* from Aurela River, a tributary of the Tagus Basin that is geographically close to the Erjas sub‐basin. Likewise, individuals of *C*. *vettonica* from the Caparro river population (belonging to V2 and S2) were more related to those of *C*. *paludica* from the populations in the Salor, Tamuja, Ibor, Huebra, and Yeltes rivers. The remaining populations of *C*. *vettonica* included in the SNP analysis belonging to the V2 and S2 lineages (Alagón, Cdh, Jerte, and Francia rivers) did not show a clear phylogenetic structure with several intermediate individuals.

### 3.2 Genetic structure

**FIGURE 4:**
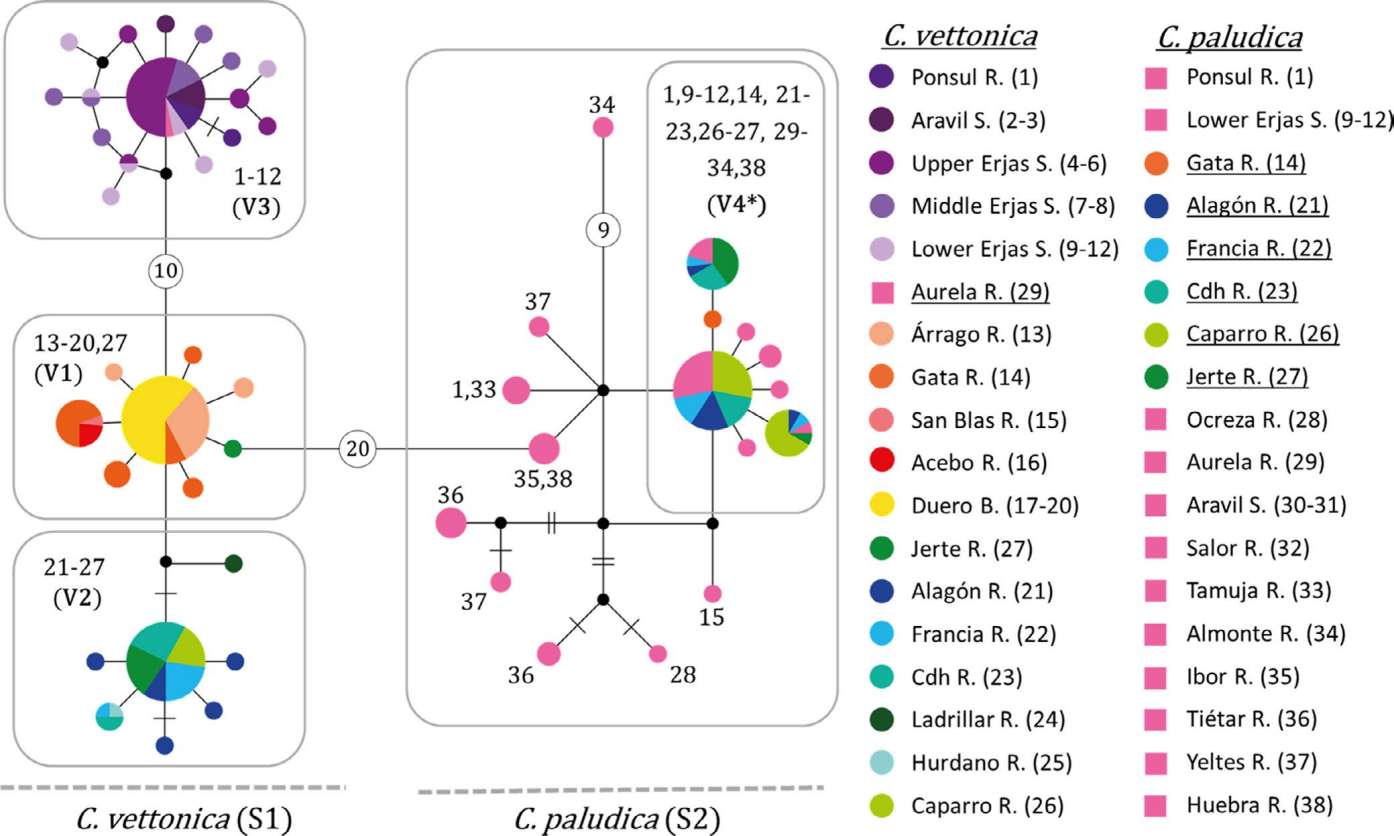
Haplotype network for the mitochondrial marker *MT*‐*CYB*. The localities are indicated by different colors and numbers (see Table 1). Mutational steps are represented as follows: one short line for two steps, two short lines for three steps, or a circle with the number of steps indicated for four or more steps. V4* refers only to those individuals identified as *C*. *vettonica* and found in the S2 lineage (individuals indicated by pink are not included in V4*). In the legend, populations identified as *C*. *vettonica* are represented by circles, and those identified as *C*. *paludica*, by squares. Populations that contain individuals with contrasting morphological and genetic identifications are underlined. Abbreviations: R. for river; S. for sub‐basin and B. for basin

**FIGURE 5:**
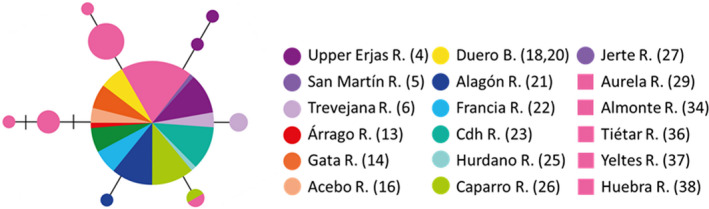
Haplotype network for the nuclear marker *RAG1*. The colors correspond to the different localities, except pink, which is used for all localities of *C*. *paludica*. Mutational steps are represented as follows: no line for one step and one short line for two steps. In the legend, the populations identified as *C*. *vettonica* are represented by circles and those identified as *C*. *paludica* are represented by squares. Numbers correspond to those used in Table 1

The haplotype network for *MT*‐*CYB* was consistent with the phylogenetic reconstruction for *MT*‐*CYB*. The network showed two main groups separated by 20 mutational steps corresponding to the species *C*. *vettonica* and *C*. *paludica* (S1 and S2, respectively; Figure 4). For *C*. *vettonica* (S1), the network was more informative than the phylogenetic reconstruction since there were three well‐differentiated haplogroups (V1, V2, and V3). The first haplogroup (V1) clustered populations from the Águeda sub‐basin in the Duero Basin and in the Tagus Basin, those from the western part of Alagón sub‐basin, plus one individual belonging to Jerte River (eastern Alagón sub‐basin) (Figure 4). These populations corresponded to the nondifferentiated lineage obtained in the phylogenetic reconstruction (i.e., V1 in Figure 4). The second haplogroup (V2) was composed of all the eastern Alagón sub‐basin populations in the Tagus Basin, corresponding to the V2 lineage obtained in the phylogenetic reconstruction. Likewise, the third haplogroup (V3), corresponding to the V3 lineage in the phylogenetic reconstruction, included populations from the western limit of the distribution range of *C*. *vettonica* in the Tagus Basin, namely those from the Ponsul, Aravil, and Erjas sub‐basins, plus one individual from Salor River. The individual from Salor River (identified as *C*. *paludica*) presented the most common haplotype of the haplogroup. Haplogroups V1 and V2 were separated by three/four mutational steps, and V3 was separated from V1 by 10 mutational steps. In the group comprising haplotypes found mainly in *C*. *paludica* (S2), a subset was composed of individuals identified as *C*. *vettonica* from the Alagón and Erjas sub‐basins (V4; referring to only those individuals identified morphologically as *C*. *vettonica* but that are genetically closer to *C*. *paludica* according to the mitochondrial data) along with other individuals of *C*. *paludica* from other Tagus and Duero tributaries. However, no evidence of a geographic structure was found within S2. The haplotype network for the nuclear marker (*RAG1*) showed a single group in which all studied populations of both species were represented and therefore was of low resolution (Figure 5).

According to the AMOVAs with the *MT*‐*CYB* data, the highest percentage of variance among groups was when the groupings were comprised of the lineages found in the previous analysis (V1, V2, V3, and V4; Table 3). The percentage of variance explained between basins (Duero and Tagus) was not significant, with most of the variation explained by differences between and within populations. Population differentiation between localities within each lineage of *C*. *vettonica* for *MT*‐*CYB* was low (*Φ*_st_ < 0.34), and no significant differences were found, except for the Acebo River, which showed a high level of differentiation with the Árrago river (*Φ*_st_ > 0.74; Table S4). High values of *Φ*_st_ were detected between populations from different lineages (*Φ*_st_ > 0.75).

**TABLE 3:** Results of the AMOVA for *MT*‐*CYB* for groups established according to basin, sub‐basin and lineage (see text for more details)

	Source of variation	*df*	Sum of squares	Variance components	Percentage of variation	Fixation indices		
						FST	FSC	FCT
Group 1: Basin	Among groups	1	49.26	−0.08	−2.29	0.91	0.91	−0.02
	Among populations within groups	21	386.16	3.12	92.92***			
	Within populations	127	39.9	0.31	9.37***			
	Total	149	491.13	3.54				
Group 2: Sub‐basin	Among groups	4	363	3.54	78.49***	0.93	0.68	0.78
	Among populations within groups	18	72.42	0.66	14.55***			
	Within populations	127	39.90	0.31	6.96***			
	Total	149	475.31	4.51				
Group 3: Lineages	Among groups	2	419.46	4.24	91.46***	0.93	0.2	0.91
	Among populations within groups	20	15.96	0.08	1.77***			
	Within populations	127	39.9	0.31	6.77***			
	Total	149	475.31	4.63				

Notes

Groupings refer to the categories shown in Table 1. Significance: *p < .05; **p < .01; ***p < .001; *df* = degrees of freedom.

**FIGURE 6:**
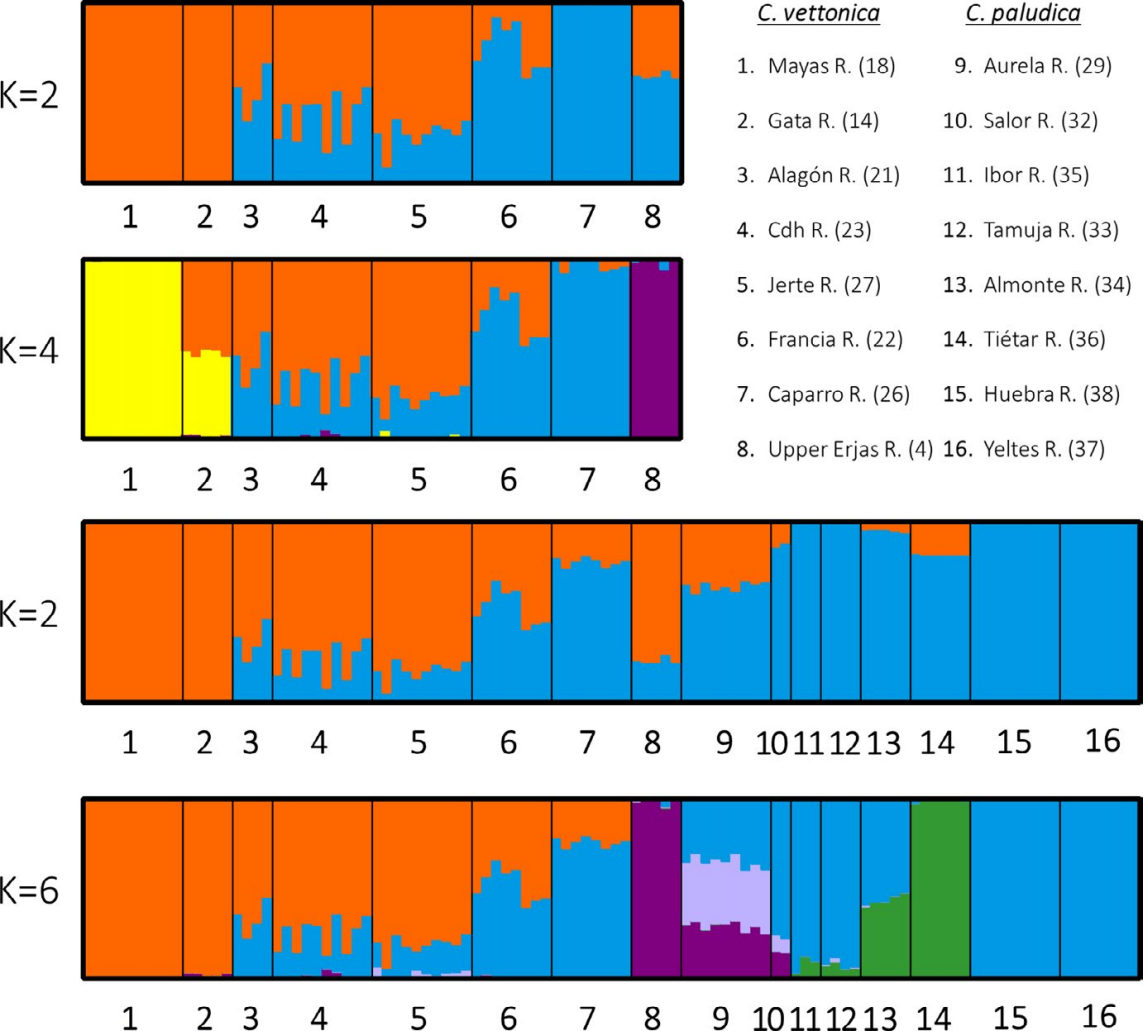
Results of the STRUCTURE analyses. The first graph shows the results obtained for *K* = 2 and *K* = 4 when only the populations of *C*. *vettonica* were analyzed (1–8). The third and fourth graphs show the results obtained when *K* = 2 and *K* = 6 when populations of both *C*. *vettonica* (1–8) and *C*. *paludica* (9–16) were analyzed. Abbreviation: R. for river

The results of the SNP structure analyses showed that the most probable number of subpopulations (genetic groups) for *C*. *vettonica* matrix was *K* = 4 and *K* = 2 for Puechmaille and Δ*K* methods, respectively (Table S5). For *K* = 4, only three populations showed very little admixture: Mayas River in the Duero Basin (first group, in yellow) and Caparro and Erjas rivers in the Tagus Basin (second and third groups, in blue and purple, respectively) (Figure 6). The other populations (Alagón, Cdh, Jerte, and Francia) showed different degrees of admixture of the second (blue) and fourth (orange) groups. Gata River had a similar level of admixture of the first (yellow) and fourth (orange) groups with a small contribution from the third (purple). The results for *K* = 2 were consistent with those obtained for *K* = 4. Mayas and Gata belonged to the first group (orange), Caparro to the second group (blue), and the rest of the populations presented a mixture of both groups. When we analyzed the populations of both species, the most probable number of subpopulations was *K* = 6 and *K* = 2 for Puechmaille and Δ*K* methods, respectively (Table S5). For *K* = 6, Mayas and Gata comprised the first group (orange), with Gata having a small contribution from the third group (purple) in all individuals (Figure 6). Alagón, Cdh, Jerte, Francia, and Caparro showed different degrees of admixture of the first (orange) and second (blue) groups, with Cdh and Francia also having a small proportion of the third group (purple) and Jerte having a small proportion of the fourth group (pink). The second group (blue) was present in all populations of *C*. *paludica*. The upper Erjas River was assigned to a single genetic group (Group 3; purple), which also comprised approximately 29% and 15% of the total composition of the Aurela and Salor rivers, respectively, with very small contributions from the second (blue) and fourth (pink) groups in some individuals. The results for *K* = 2 were consistent with those obtained for *K* = 6. Mayas and Gata belonged to the first group (orange), Ibor, Tamuja, Huebra, and Yeltes to the second group (blue), and the rest of the populations presented a mixture of both groups.

### 3.3 Genetic diversity and demography

Overall genetic diversity parameters for all individuals identified as *C*. *vettonica* based on *MT*‐*CYB* were high (Table 4). Excluding populations of *C*. *vettonica* in which only one individual was studied, *Hd* ranged from 0 to 1, and *h* ranged from 1 to 7. The populations with the lowest values of genetic diversity were Mayas, Acebo, Arades, and Caparro with only one haplotype each (Table 4). The most variable populations for all of the diversity parameters were the lower and middle Erjas, Alagón, and Gata. The sub‐basins with the highest genetic diversity values were Erjas and Alagón, specifically the western Alagón sub‐basin. The one with the lowest values was Águeda with only one haplotype.

**TABLE 4:** Genetic variability parameters for *MT*‐*CYB* and *RAG1* and the results of the neutrality tests of Tajima and Fu for the different groups (see text for details). The * indicate significance values

General	*MT‐CYB*							*RAG1*				
	*N*	*h*	*H*_d_	*π*	S	Tajima's *D*	Fu's FS	*N*	*a*	*a*_d_	*π*	S
*C. vettonica*	150	31	0.85	5.86E‐03	38	−0.18	−4.8	45	4	0.09	8.00E‐05	3

By locality	*N*	*h*	*H*_d_	*π*	S	Tajima's *D*	Fu's FS	*N*	*a*	*a*_d_	*π*	S
Alfrividas R.	4	2	0.5	9.20E‐04	2	−0.70	1.09	–	–	–	–	–
Aravil R.	5	2	0.4	3.70E‐04	1	−0.81	0.09	–	–	–	–	–
Upper Erjas R.	12	4	0.46	4.60E‐04	3	−1.62*	−2.12*	9	3	0.21	2.30E‐04	2
San Martin R.	6	2	0.33	6.10E‐04	2	−1.13	0.95	1	1	0	0	0
Trevejana R.	4	2	0.5	4.60E‐04	1	−0.61	0.17	4	2	0.43	3.00E‐04	1
Middle Erjas R.	6	6	1	1.78E‐03	5	−0.65	−4.45*	–	–	–	–	–
Lower Erjas S.	9	7	0.92	1.94E‐03	8	−1.28	−3.61*	–	–	–	–	–
Arades R.	2	1	0	0	0	–	–	–	–	–	–	–
Árrago R.	15	3	0.36	3.50E‐04	2	−1.00	−0.91	3	1	0	0	0
Gata R.	18	5	0.72	1.05E‐03	4	−0.04	−0.91	5	1	0	0	0
San Blas R.	1	1	–	–	–	–	–	–	–	–	–	–
Acebo R.	3	1	0	0	0	–	–	1	1	0	0	0
Mayas R.	22	1	0	0	0	–	–	4	1	0	0	0
Águeda R.	1	1	–	–	–	–	–	–	–	–	–	–
Turones R.	1	1	–	–	–	–	–	1	1	0	0	0
Alagón R.	7	5	0.86	1.31E‐03	5	−1.48*	−2.31*	6	1	0	0	0
Francia R.	8	2	0.25	2.30E‐04	1	−1.05	−0.18	1	1	0	0	0
Cdh R.	10	2	0.36	3.30E‐04	1	0.01	0.41	4	1	0	0	0
Ladrillar R.	1	1	–	–	–	–	–	–	–	–	–	–
Hurdano R.	1	1	–	–	–	–	–	1	1	0	0	0
Caparro R.	6	1	0	0	0	–	–	2	1	0	0	0
Jerte R.	8	2	0.25	9.20E‐04	4	−1.53*	1.94	3	1	0	0	0

By sub‐basin	*N*	*h*	*H*_d_	*π*	S	Tajima's *D*	Fu's FS	*N*	*h*	*H*_d_	*π*	*S*
Ponsul S.	4	2	0.5	9.20E‐04	2	−0.71	1.09	–	–	–	–	–
Aravil S.	5	2	0.4	3.70E‐04	1	−0.81	0.09	–	–	–	–	–
Erjas S.	39	14	0.66	1.03E‐03	12	−1.87*	−12.04*	14	4	0.29	2.60E‐04	3
Águeda S.	24	1	0	0	0	0	0	5	1	0	0	0
Alagón S.	78	15	0.781	2.12E‐03	17	−0.96	−4.34*	26	1	0	0	0
Western Alagón S.	37	7	0.71	8.60E‐04	6	−0.94	−2.62*	–	–	–	–	–
Eastern Alagón S.	41	8	0.43	7.00E‐04	11	−2.12*	−4.56*	–	–	–	–	–

By lineage (*MT*‐*CYB*)	*N*	*h*	*H*_d_	*π*	S	Tajima's *D*	Fu's FS	*N*	*h*	*H*_d_	*π*	S
V1	62	8	0.56	6.00E‐04	7	−1.42	−4.54*	15	1	0	0	0
V2	40	7	0.4	5.40E‐04	9	−2.11*	−4.36*	16	1	0	0	0
V3	48	16	0.61	9.60E‐04	15	−2.14*	−15.53*	14	4	0.29	2.60E‐04	3

By OCUs (*MT*‐*CYB*)	*N*	*h*	*H*_d_	*π*	S	Tajima's *D*	Fu's FS	*N*	*h*	*H*_d_	*π*	S
OCU I	24	1	0	0	0	–	–	5	1	0	0	0
OCU II	37	7	0.72	8.60E‐04	6	−0.94	−2.63	9	1	0	0	0
OCU III	41	8	0.42	7.00E‐04	11	−2.13*	−4.57*	17	1	0	0	0
OCU IV	48	16	0.61	9.60E‐04	15	−2.14*	−15.53*	14	4	0.29	2.60E‐04	3

Notes

The following parameters are included in the table for *MT*‐*CYB*: *N*: number of analyzed samples; *h*: haplotype number; *H*_d_: haplotype diversity; *π*: nucleotide diversity; *S*: number of polymorphic sites. The following parameters are included in the table for *RAG1*: *N*: number of analyzed samples; *a*: number of nuclear alleles; *a_d_*: allelic diversity; *π*: nucleotide diversity; *S*: number of polymorphic sites. Abbreviations: R. for river and S. for sub‐basin.

The highest number of private alleles for *C*. *vettonica*, based on the SNP data, was found in the upper Erjas (227 alleles) and Caparro (76 alleles) rivers (Table 5). However, when the complete matrix including the populations of *C*. *paludica* was taken into account, the number of private alleles decreased drastically for all rivers, except Mayas (41 vs. 40) and Gata (1 vs. 0), whose numbers remained nearly the same as when only *C*. *vettonica* was considered. In the upper Erjas and Caparro rivers, the number of private alleles decreased to eight and two, respectively (Table 5). Notably, when Aurela and Salor rivers were removed from the analysis, the number of private alleles in the Erjas population rose to 141. The lowest nucleotide diversity values were found in Mayas, Gata, and the upper Erjas rivers, and the highest, in Alagón, Cdh, Jerte, Francia, and Caparo rivers (Table 5).

**TABLE 5:** Genetic diversity parameters for the SNP data

Loc	Matrix	Pri	Pri*	*V*_s_	P	%P	*H*_o_	*H*_e_	*π*	*F*_IS_
Mayas R. (18)	V	41	–	3992	193	4.83	0.02 ± 0.011	0.017 ± 0.007	0.018 ± 0.007	−0.006 ± 0.005
	VP	40	40	4528	193	4.26	0.018 ± 0.01	0.015 ± 0.006	0.016 ± 0.007	−0.005 ± 0.004
Gata R. (14)	V	1	–	3998	502	12.56	0.047 ± 0.022	0.041 ± 0.014	0.046 ± 0.017	−0.00008 ± 0.017
	VP	0	0	4535	502	11.07	0.041 ± 0.02	0.036 ± 0.012	0.041 ± 0.015	−0.00007 ± 0.015
Alagón R. (21)	V	14	–	3999	2493	62.34	0.257 ± 0.07	0.227 ± 0.038	0.26 ± 0.05	0.003 ± 0.082
	VP	1	2	4537	2493	54.95	0.227 ± 0.068	0.2 ± 0.039	0.229 ± 0.051	0.003 ± 0.072
Cdh R. (23)	V	21	–	4000	2918	72.95	0.198 ± 0.033	0.204 ± 0.028	0.216 ± 0.031	0.046 ± 0.064
	VP	1	2	4538	2918	64.30	0.174 ± 0.033	0.18 ± 0.029	0.19 ± 0.032	0.041 ± 0.057
Jerte R. (27)	V	39	–	4000	2664	66.6	0.182 ± 0.036	0.172 ± 0.026	0.182 ± 0.029	0.0005 ± 0.048
	VP	2	2	4538	2664	58.70	0.16 ± 0.035	0.152 ± 0.026	0.16 ± 0.029	0.0004 ± 0.043
Francia R. (22)	V	28	–	4000	3042	76.05	0.309 ± 0.063	0.285 ± 0.038	0.306 ± 0.044	−0.006 ± 0.084
	VP	1	1	4538	3042	67.03	0.272 ± 0.066	0.251 ± 0.042	0.269 ± 0.048	−0.005 ± 0.074
Caparro R. (26)	V	76	–	4000	3053	76.33	0.282 ± 0.053	0.261 ± 0.033	0.279 ± 0.038	−0.007 ± 0.074
	VP	2	3	4538	3053	67.28	0.249 ± 0.055	0.23 ± 0.036	0.246 ± 0.041	−0.006 ± 0.066
Upper Erjas R. (4)	V	227	–	3960	496	12.53	0.039 ± 0.016	0.035 ± 0.011	0.039 ± 0.014	0.001 ± 0.01
	VP	8	141	4482	496	11.07	0.034 ± 0.014	0.031 ± 0.01	0.035 ± 0.012	0.001 ± 0.009
Aurela R. (29)	VP	15	–	4538	3145	69.3	0.24 ± 0.05	0.24 ± 0.04	0.25 ± 0.04	0.0265 ± 0.073
Salor R. (32)	VP	0	–	4515	1665	36.88	0.2 ± 0.1	0.15 ± 0.04	0.21 ± 0.08	0.0125 ± 0.062
Ibor R. (35)	VP	10	13	4534	1470	32.42	0.15 ± 0.07	0.12 ± 0.03	0.15 ± 0.06	0.0114 ± 0.051
Tamuja R. (33)	VP	2	5	4532	1582	34.91	0.14 ± 0.05	0.12 ± 0.03	0.15 ± 0.05	0.0163 ± 0.053
Almonte R. (34)	VP	25	40	4516	1298	28.74	0.11 ± 0.04	0.09 ± 0.03	0.11 ± 0.03	−0.0028 ± 0.038
Tiétar R. (36)	VP	146	175	4484	743	16.57	0.05 ± 0.02	0.05 ± 0.02	0.06 ± 0.02	0.0104 ± 0.023
Huebra R. (38)	VP	8	8	4538	1926	42.44	0.14 ± 0.04	0.14 ± 0.03	0.15 ± 0.04	0.0159 ± 0.042
Yeltes R. (37)	VP	7	9	4534	1042	22.98	0.09 ± 0.04	0.08 ± 0.03	0.09 ± 0.03	0.0033 ± 0.027

Notes

Two matrices were analyzed: one including only the populations of *C*. *vettonica* (V) and the other, the populations of *C*. *vettonica* and *C*. *paludica* together (VP), as indicated in the Matrix column. Pri: number of private alleles; Pri*: number of private alleles when Aurela and Salor were excluded from the analysis (only shown for VP grouping) *V*_s_: number of variant sites; P: number of polymorphic sites; %P: percentage of polymorphic sites; *H*_o_: observed heterozygosity; *H*_e_: expected heterozygosity; *π*: nucleotide diversity; *F*_IS_: inbreeding coefficient. Abbreviation: R. for river.

Significant negative values for the neutrality tests indicate a population expansion following a bottleneck. All three of the detected lineages were significantly negative for Fu's FS; V2 and V3 were also significantly negative for Tajima's *D* (Table 4).

### 3.4 Divergence time and niche modeling

**FIGURE 7:**
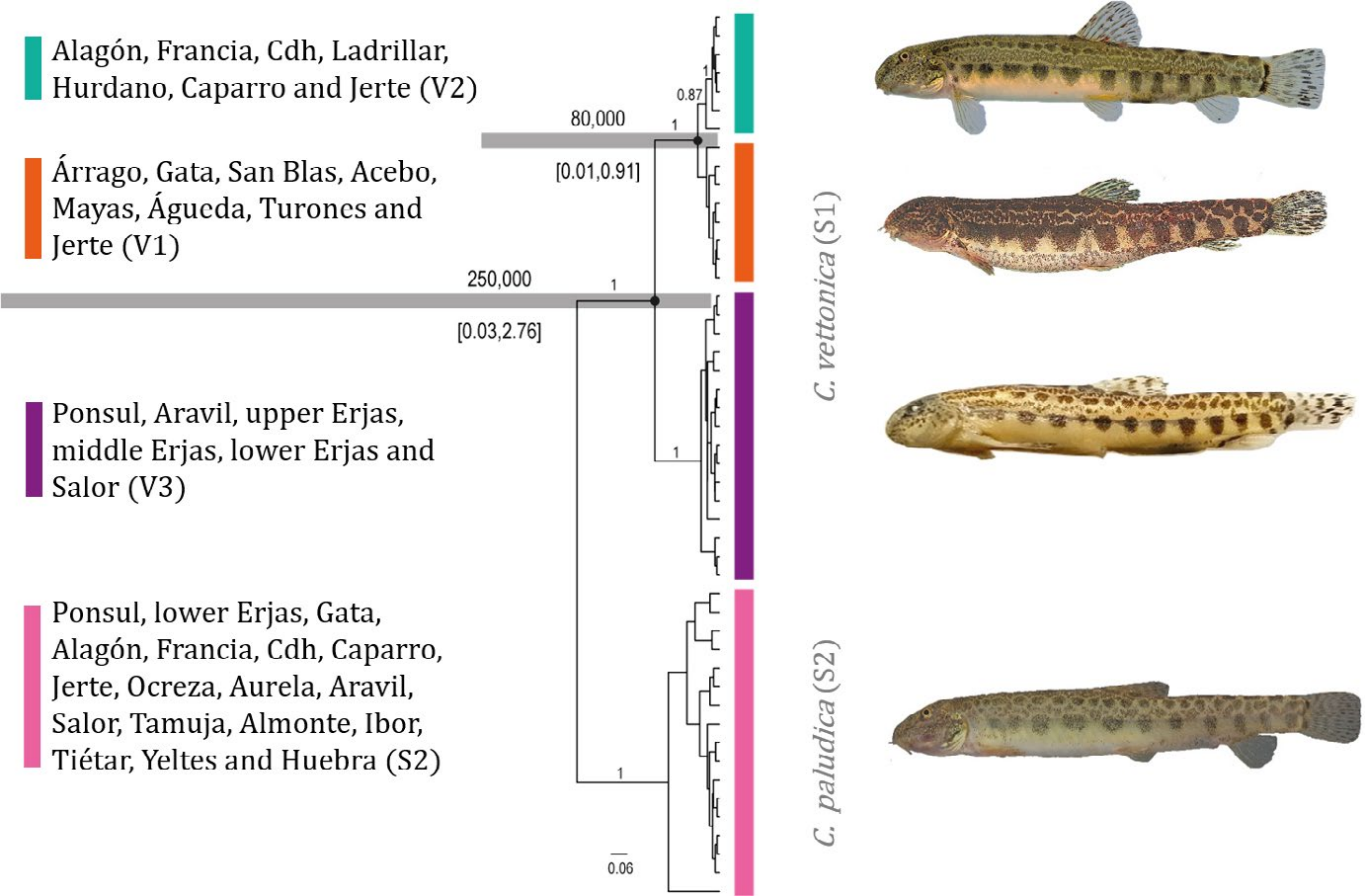
Phylogenetic relationships and divergence time estimates based on the analysis of the mitochondrial marker *MT*‐*CYB* in BEAST. The horizontal bars represent the 95% HPD intervals. Posterior probabilities of the main nodes are indicated above branches. S1: *Cobitis vettonica*. The vertical colored bars represent the three main lineages (V1–V3) of the species and their localities (see legend). S2: *Cobitis paludica*. On the right, images of representative specimens belonging to one of the localities of each lineage: (from top to bottom): Caparro, Gata, middle Erjas, and Tiétar rivers

The topology of the tree obtained by calibrating the molecular clock was identical to that of the phylogenetic Bayesian tree (Figure 7) but showed higher support for the V2 lineage (pp = 0.99 vs. pp = 0.87). The V3 lineage diverged approximately 250 Ka (HPD 95%: 30 Ka–2.76 Ma), while V1 and V2 diverged from each other approximately 80,000 years ago (HPD 95%: 10–910 Ka).

**FIGURE 8:**
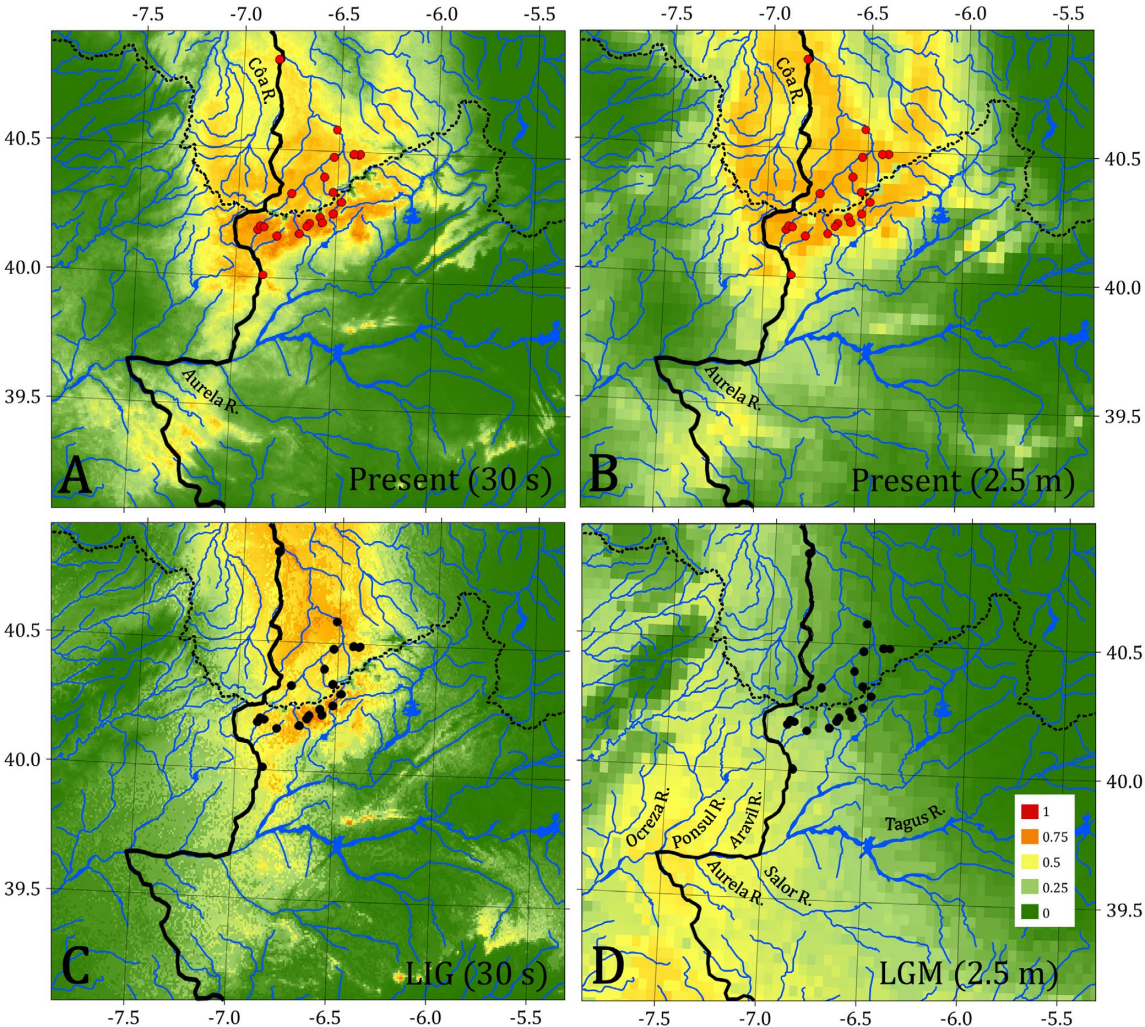
Modeling of the potential niche of *C*. *vettonica* for (A) and (B) the present (1970–2000) at a resolution of 30 s (s) and 2.5 min (m), respectively, (C) Last Interglacial (LIG; 130,000 years ago) and (D) Last Glacial Maximum (LGM; 22,000 years ago). The colors indicate the probability of the presence of the species (see legend). Some of the rivers (R.) mentioned in the text are labeled

All niche distribution models (Figure 8) had AUC values greater than 0.97. Values above 0.75 are considered useful, while those above 0.90 are very good (Elith et al., 2006; Swets, 1988). Mean habitat suitability values above 0.7 were found for the present‐day period for all localities where *C*. *vettonica* is currently present, except some areas of the eastern Alagón sub‐basin, including Jerte, Francia, Caparro, Alagón, and Cdh rivers, and the lower and middle courses of the Ponsul and Aravil sub‐basins. In addition, a potentially habitable zone outside the known current distribution area for *C*. *vettonica* was observed upstream of Aurela River, a tributary of the left margin of the Tagus Basin, and also upstream of Côa River, a tributary of the Duero Basin that is adjacent to the Águeda sub‐basin (Figure 8A,B). For the LIG period (Figure 8C), the area with the optimal conditions for the species generally overlapped with that found for the present, although the highest probability values were concentrated in the central part of the species’ current distribution (i.e., the western Alagón and Águeda sub‐basins in the Tagus and Duero basins, respectively). For the LGM period (Figure 8D), optimal conditions were located in the southwestern area of the species current distribution (the Erjas and lower Árrago sub‐basins and Salor, Aravil, and Ponsul sub‐basins). Some rivers outside the current distribution of the species in Portugal also showed optimal conditions.

### 3.5 Ancestral area reconstructions

**FIGURE 9:**
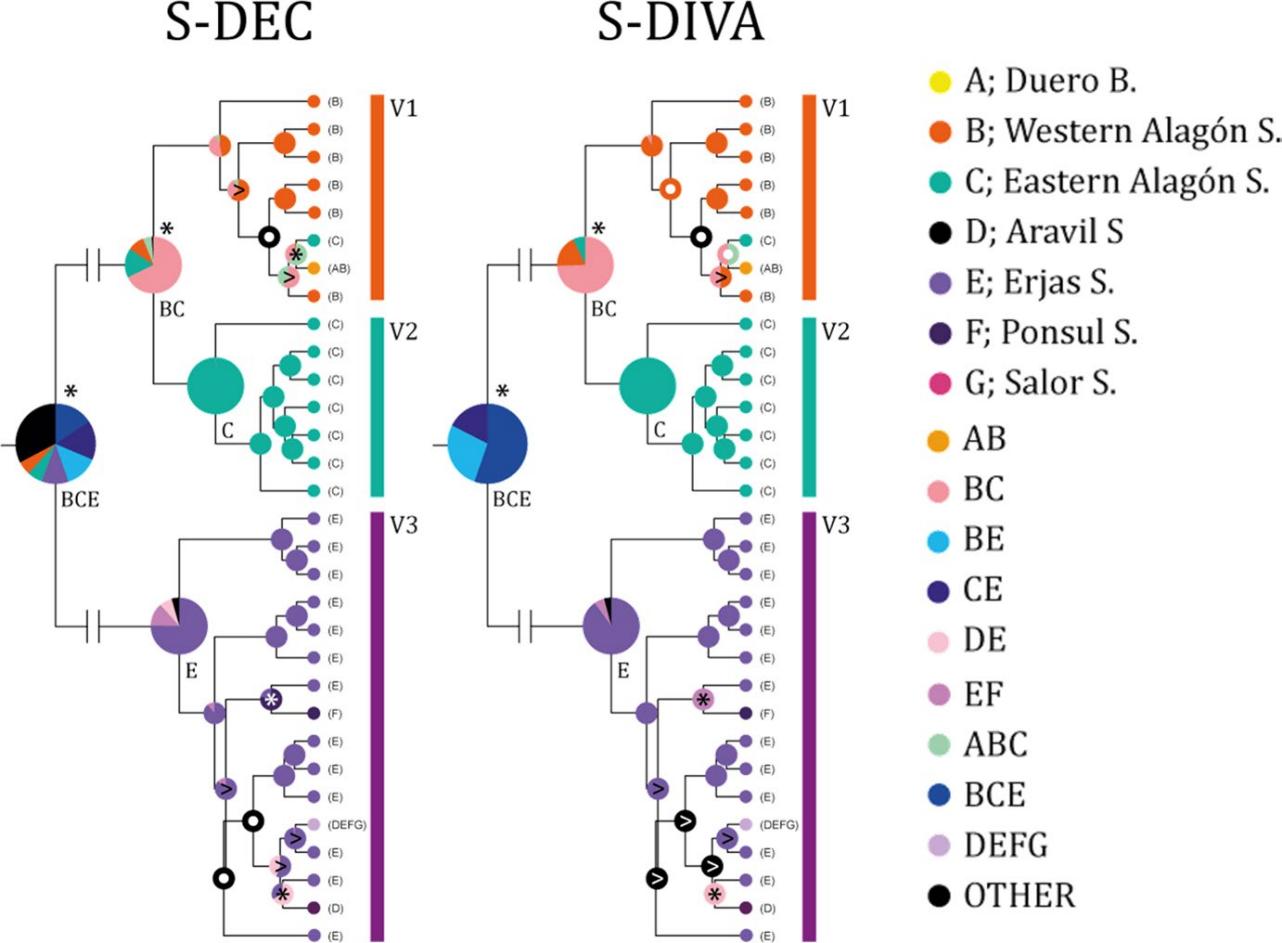
Reconstruction of the ancestral area using either S‐DIVA or S‐DEC. Only the lineage of *C*. *vettonica* is shown. The color(s) of the circles indicates the most probable ancestral area for each node (see legend for color code). Vicariant events are indicated with an *, and dispersals with a<. Abbreviations; S. for sub‐basin and B. for basin

In general, the results of the ancestral area reconstructions using either the S‐DIVA or S‐DEC method were congruent (Figure 9). Both the vicariant events between lineages and the ancestral area with the highest marginal probability were consistent between the two methods. The most probable ancestral areas were as follows: Alagón–Erjas sub‐basins for the junction node of V1‐V2‐V3, Alagón sub‐basin for V1‐V2, and Erjas sub‐basin for V3. Both analyses supported the diversification of the main evolutionary lineages due to vicariant events. Several dispersal and vicariant events were also detected within V1 and V3 (Figure 9).

## 4 DISCUSSION

### 4.1 Genetic structure

A strong population genetic structure, based on mitochondrial (*MT*‐*CYB*) and SNP data, was detected for *C*. *vettonica*, in line with previous molecular studies of *MT*‐*CYB* (Doadrio et al., 2021; Perdices & Coelho, 2020). No differences were found for nuclear *RAG1*, neither among the populations of *C*. *vettonica* nor between *C*. *vettonica* and *C*. *paludica*. Similar findings have also been reported for this gene in other Iberian species, probably as a consequence of incomplete lineage sorting (Corral‐Lou et al., 2019; Perea et al., 2021).

The population structure of *C*. *vettonica* based on the mitochondrial data differed from the main pattern observed in other European freshwater fishes, which is that gene flow within the same basin is greater than among different basins (e.g., Buj et al., 2017; Corral‐Lou et al., 2019; Marić et al., 2017, 2019; Seifertová et al., 2012; Wetjen et al., 2020). Specifically, for *C*. *vettonica*, genetic differentiation between populations from the same basin (Tagus Basin) is greater than between populations from the two main basins (Duero and Tagus basins). Similarly, greater differentiation within the same sub‐basins than between different sub‐basins has been found for *Achondrostoma salmantinum*, which has a restricted distribution area that partially overlaps with that of *C*. *vettonica* (Corral‐Lou et al., 2021). Iberian freshwater fishes with a wider distribution range that partially or completely overlaps with that of *C*. *vettonica*, such as *Squalius carolitertii*, *S*. *pyrenaicus*, *S*. *alburnoides*, or *Luciobarbus bocagei*, do not show a population structure pattern similar to that of *C*. *vettonica* (Cunha et al., 2004; Doadrio et al., 2002; Perea et al., 2021; Sousa‐Santos et al., 2007). Differences in the genetic pattern of the various species may be due to differences in size and dispersal abilities. Species of *Cobitis* are smaller benthic organisms with lower dispersal abilities (like *A*. *salmantinum*) than species of *Luciobarbus* and *Squalius* (Doadrio et al., 2011). This difference in the biology of freshwater fish species, along with different colonization times, could account for the population structure found in *C*. *vettonica* and *A*. *salmantinum* and the lack of structure in the species of *Luciobarbus* and *Squalius* (see Biogeography section).

#### 4.1.1 (V1) Western Alagón sub‐basin and Duero Basin

**FIGURE 10:**
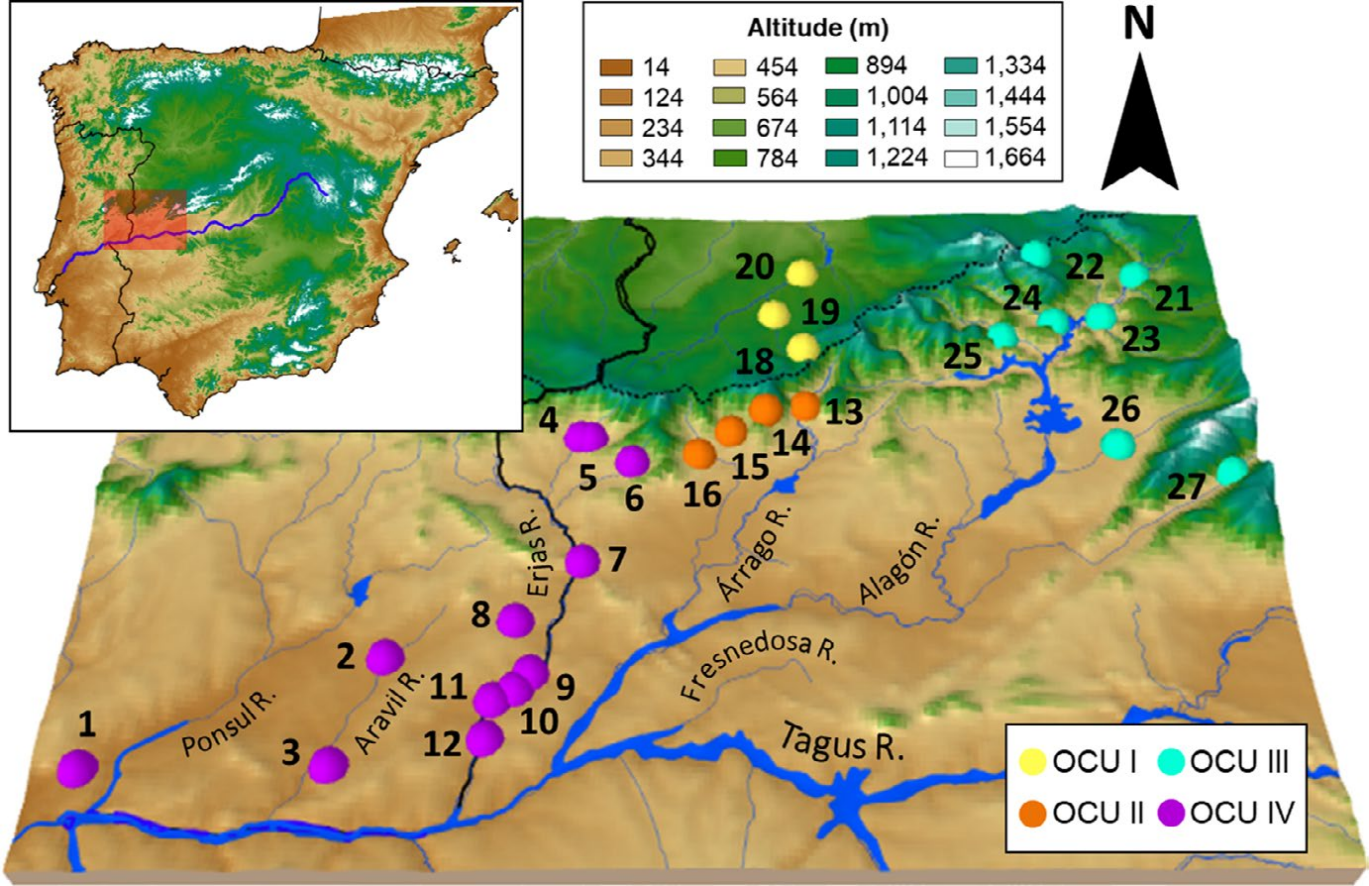
3D map indicating the sampling locations referenced in Table 1. The color of the sampling point indicates the Operational Conservation Units (OCU I to IV) to which it is assigned. Some of the main rivers sampled in the study are also indicated on the map

This mitochondrial lineage included the populations from the headwaters of the rivers in the western Alagón sub‐basin in the Tagus Basin, and those in the Duero Basin. Only one individual from Jerte River in the eastern Alagón sub‐basin had a haplotype close to the western lineage, however, it was not shared by any other locality in the western Alagón sub‐basin or the Duero Basin. Therefore, it is possible that the close relationship between this and the rest of the haplotypes is a consequence of an ancestral polymorphism; hence, we did not consider Jerte River within this lineage. The results of the SNP analyses showed slight differentiation between the Duero and Tagus populations, indicating that these isolated populations were joined in the past. These results suggest that the Duero basin and western Alagón sub‐basin populations of *C*. *vettonica*, which are currently assigned to single OCU (Doadrio et al., 2021), should be assigned to two separate OCUs (OCU I and OCU II, respectively; Figure 10).

The Duero populations of *C*. *vettonica* (OCU I) showed the lowest diversity values of all the populations. Two possible explanations may account for this low diversity: in the first case, these populations may have not yet recovered from a recent bottleneck that occurred following their separation from the western Alagón sub‐basin, and in the second, they may have not yet diversified following a founder event that occurred after the estimated dispersal of a small number of individuals from the rivers of the western Alagón sub‐basin (as suggested in our ancestral area reconstructions).

#### 4.1.2 (V2) Eastern Alagón sub‐basin

This group, composed of the populations from the eastern Alagón sub‐basin, was not monophyletic based on *MT*‐*CYB*, and in the SNP analysis, presented a high level of admixture with *C*. *paludica*. These results suggest extensive genetic introgression of *C*. *vettonica* with its sister species *C*. *paludica*, except in Hurdano and Ladrillar rivers, where only one individual from each was studied. Therefore, regardless of the different levels of genetic introgression, we propose that these populations all be assigned to the OCU that was recently established by Doadrio et al. (2021) (OCU III; Figure 10).

#### 4.1.3 (V3) Ponsul, Aravil, and Erjas sub‐basins

According to the *MT*‐*CYB* analyses, the Ponsul, Aravil, and Erjas sub‐basin populations constituted a well‐differentiated independent lineage of *C*. *vettonica*, supporting evidence from other studies that suggest the uniqueness of the Erjas populations (Doadrio et al., 2011, 2021). The upper Erjas was also an independent group, according to the SNP analyses, indicating its present‐day isolation with respect to the rest of the populations in the Alagón sub‐basin and Duero Basin. For these reasons, we include the upper Erjas, Ponsul, and Aravil sub‐basins in the OCU previously established for only the Erjas sub‐basin (Doadrio et al., 2021), thereby expanding this OCU’s area of coverage (OCU IV; Figure 10).

### 4.2 Hybridization

Hybridization due to introgression as a result of both anthropic and natural factors has been detected for other Mediterranean cyprinid and cobitid species (Almodóvar et al., 2012; Bohlen & Ráb, 2001; Choleva et al., 2014; Corral‐Lou et al., 2019; Cunha et al., 2004; Denys et al., 2013; Janko et al., 2005; Perea et al., 2016; Tancioni et al., 2013). Within the Iberian Peninsula, the majority of the natural hybrid populations originated during the Quaternary period as a consequence of hydrographical rearrangements and/or Pleistocene glaciation–deglaciation cycles (Almodóvar et al., 2012; Gante, 2009). Consistent with this, we present strong evidence confirming the introgression previously reported in the eastern Alagón populations of *C*. *vettonica* by Doadrio et al. (2011), Doadrio et al. (2021), and also postulate the genetic introgression of populations in sympatric areas (Ponsul, Aravil, and low Erjas; see below). Supporting this hypothesis is the location of these populations outside the potential range of the species based on the ecological niche models. The introgression of even a few loci due to hybridization can lead to adaptive divergence if the loci are better adapted to other niches and persist in the genome (Abbott et al., 2013).

#### 4.2.1 (V1) Western Alagón sub‐basin and Duero Basin

The Gata population within the western Alagón sub‐basin was the only one that presented a mitochondrial haplotype of *C*. *paludica*. However, in the SNP analysis, none of the individuals showed admixture with *C*. *paludica*, and they also resolved as a phylogenetically isolated group. Therefore, at present, there is no evidence to support genetic introgression with *C*. *paludica* in this area.

#### 4.2.2 (V2) Eastern Alagón sub‐basin

We found strong evidence of genetic introgression between *C*. *paludica* and *C*. *vettonica* in the eastern Alagón sub‐basin, in line with previous studies (Doadrio et al., 2011, 2021). These populations not only showed a high level of admixture with *C*. *paludica* but also high values of genetic diversity in the SNP analyses. Indeed, they had the highest values of all the populations, as would be expected for hybrids populations. The high diversity values obtained for the SNP data were not congruent with the low ones obtained for *MT*‐*CYB*. However, this observation is not surprising as hybrids typically cannot be detected by mitochondrial markers due to their maternal inheritance.

The case of the population from Caparro River is particularly interesting. It was the only eastern Alagón sub‐basin population that showed a closer phylogenetic relationship with populations of *C*. *paludica* than with those of *C*. *vettonica* and a higher degree of genetic introgression. This fact could be explained by two hypotheses. The first is that the source population was *C*. *paludica* and, through a founder effect, a few individuals of *C*. *vettonica* established themselves in this locality, giving rise to a population of *C*. *paludica* introgressed by *C*. *vettonica*. The second hypothesis is that the source population was *C*. *vettonica*, but it was displaced by *C*. *paludica* after this species’ arrival due to its better adapted genome (Abbott et al., 2013).

#### 4.2.3 (V3) Ponsul, Aravil, and Erjas sub‐basins

According to the mitochondrial analyses, the Ponsul, Aravil, and Erjas sub‐basin populations of *C*. *vettonica* were related to a population of *C*. *paludica* from Salor River whose most common haplotype was also shared by all of these populations of *C*. *vettonica*. In addition, the results of the SNP analysis revealed a strong relationship between the Erjas population (upper Erjas) with those in the Salor and Aurela sub‐basins, particularly the latter with which it was most closely related phylogenetically. The relationship between these populations was more evident when the Aurela and Salor populations were removed from the SNP genetic diversity analysis, which resulted in an increase in the number of private alleles from eight to 141. However, in the SNP structure analysis, only one individual belonging to the Erjas population had a very low contribution of the genetic group assigned to *C*. *paludica*. Altogether, the results do not support the presence of genetic introgression in the upper Erjas, where no mitochondrial haplotypes of *C*. *paludica* were found. Moreover, higher values of genetic diversity would be expected for the SNP data if there were introgressions, as occurs in the eastern Alagón sub‐basin populations.

However, we postulate genetic introgression in the Ponsul, Aravil, and lower Erjas sub‐basin, where both species are present (Perdices & Coelho, 2020). This postulation is based on their location outside the area indicated by the ecological niche models and the fact that introgression by hybridization is a common phenomenon that has been detected for not only *C*. *vettonica* and *C*. *paludica* but also other species of European *Cobitis* (Bohlen & Ráb, 2001; Janko et al., 2007; Ráb & Slavík, 1996). Furthermore, this general pattern has often been observed in the Iberian Peninsula for two sympatric species as a consequence of secondary contact after diversification (Corral‐Lou et al., 2019; Doadrio et al., 2021; Perea et al., 2016, 2021).

Within this group, hybrid localities seem to be restricted to the lower reaches of rivers, while the upper reaches are inhabited by only *C*. *vettonica* (Perdices & Coelho, 2020). The habitat preferences of the two species, *C*. *paludica* in the middle and lower reaches, and *C*. *vettonica* in the upper reaches (Doadrio et al., 2011), likely explains the restricted distribution of the hybrids.

### 4.3 Biogeography

According to our divergence time estimations, the timing of these divergences agrees with a common period of population differentiation for many Iberian freshwater fish species, which in turn coincides with the culmination of the formation of the Iberian drainage network and the Pleistocene glacial cycles (Casal‐López et al., 2017; Corral‐Lou et al., 2019, 2021; Pais et al., 2012; Perea et al., 2016). Although geological information for the study area is scarce, several tectonic and climatic events are known to have occurred during the Quaternary near the study area, which influenced the hydrogeomorphology of the aquatic network such as the fluvial capture phenomena, the presence of paleoglaciers during the last glacial cycle, and changes in the trajectory of some rivers (Benito et al., 2003; Carrasco et al., 2013, 2015; Goy et al., 2020). Therefore, we propose several hypotheses based on the biogeography of the species, which may reveal the hydrogeomorphological evolution of the study area within the Iberian Peninsula.

The earliest divergence in *C*. *vettonica* was the Ponsul‐Aravil‐Erjas lineage (V3), which split from the two Alagón sub‐basin lineages (V1‐V2) during the Pleistocene (Calabrian period, ~250,000 Mya). Based on the ancestral area reconstructions, the diversification of these lineages occurred in the area currently comprising both the Erjas and Alagón sub‐basins. This implies that the Erjas acquired its current configuration during this period of time, interrupting any gene flow that may have been established by connections between tributaries on the right bank of Erjas River and the left bank of Árrago River. Subsequently, following the isolation and diversification of the Erjas sub‐basin population from the rest of the populations, individuals from this sub‐basin dispersed to downstream areas of the Tagus Basin (reaching Ponsul, Aravil and Salor sub‐basins). These dispersal events could have taken place as a consequence of climatic changes associated with the LGM, as supported by our niche models which indicated the Ocreza, Nisa, Sever, Ponsul, Aravil, and Salor sub‐basins as more suitable habitats than the rest of the rivers in the current distribution range of the species. These events gave rise to both the colonization of new areas to the west (Ponsul and Aravil sub‐basins) and the sympatry with *C*. *paludica*, which led to introgression events.

The divergence of the two other lineages comprising the western and the eastern Alagón sub‐basin populations (V1 and V2) also occurred during the Pleistocene (Chibanian period, ~80,000 Mya). The complex geology of the Alagón sub‐basin is evident by the anomalous current trajectory of some of its rivers (Carrasco & Pedraza, 1991; Díez Herrero, 2003; Goy et al., 2020; Jiménez, 1994; Schnabel & Gutiérrez, 2014). For instance, the two banks of the Alagón sub‐basin, represented by the two mitochondrial lineages, are currently joined by the mouth of the Árrago River in the Alagón River, that is, the main river of the western and the eastern Alagón sub‐basin, respectively. The union between the two banks occurred relatively recently as a consequence of the upriver action of the Alagón River, which captured the Caparro River that eventually flowed into the Tagus through the Fresnedosa riverbank (Schnabel & Gutiérrez, 2014). Given this context, we propose two scenarios for the divergence of V1 and V2. In the first, the two lineages diverged prior to the union of the two banks and later genetic flow between populations was not possible due to the poor habitat conditions for *C*. *vettonica* in the lower Alagón sub‐basin. In the second, they diverged after the union of the banks due to climatic changes during the LGM. According to our niche models, the optimal ecological conditions for *C*. *vettonica* were located further downstream and, as predicted by phylogeography theory, the latitudinal distribution of organisms retracted southwards (Ehrich et al., 2007; Hewitt, 1996; Rodríguez et al., 2011). Once optimal conditions returned to the river headwaters, the species went back upstream toward both the east and west margins of the Alagón sub‐basin, and subsequently diversified.

After the divergence of the two banks of the Alagón, the populations in the Duero and western Alagón sub‐basin diverged. Several studies have reported on the genetic structure of freshwater fauna associated with connections between the Tagus Basin and adjacent basins (e.g., Alagón, Alberche and Lozoya rivers; Carmona et al., 2000; Casas‐Sainz & De Vicente, 2009; Doadrio, 1988; Pérez‐González, 1980; Sousa‐Santos et al., 2007). Although recent connections may be explained as a consequence of the tectonic activity in the region (Goy et al., 2020), there are no geological studies that support a recent connection between the western Alagón sub‐basin (Tagus Basin) and the Águeda sub‐basin (Duero Basin), despite the close proximity of some of their river headwaters (in some cases, as little as 150 m of linear distance). A connection, however, has been hypothesized to explain the co‐occurrence of individuals with the mitochondrial genome of *Squalius carolitertii* (distributed in the Duero Basin) and those of *S*. *pyrenaicus* (distributed in the Tagus Basin) in the Árrago River (Perea et al., 2021). The close proximity of Mayas (Duero Basin) and Árrago (Tagus Basin) rivers, whose courses are only separated by ~150 m, is particularly notable. Piracy events that occurred between these two rivers in the Pleistocene may explain the close mitochondrial relationship of the populations inhabiting them, as suggested by the dispersal event estimated in our ancestral area reconstruction analysis. Later isolation of the Duero and Tagus hydrological basins then led to the divergence of these populations, as corroborated by the SNP analysis.

### 4.4 Conservation

The habitats of *C*. *vettonica* have been and continue to be threatened by the main causes of biodiversity loss (e.g., overexploitation, water pollution, flow modification, habitat destruction and degradation, and the introduction of invasive species) (Doadrio et al., 2011; Dudgeon, 2019; Sousa‐Santos et al., 2014). Given these threats, the populations in OCU I (Águeda sub‐basin) are particularly vulnerable as they had the lowest genetic diversity values for both *MT*‐*CYB* and the SNPs, and only one *MT*‐*CYB* haplotype shared by all the populations. This low level of genetic diversity makes them more sensitive to extrinsic changes and therefore at greater risk of extinction (Frankham et al., 2002). In addition, populations in OCU I are shrinking as a result of the formation of dams along the Águeda sub‐basin, and in some localities (i.e., Turones), they have even disappeared. The species’ distribution in this area is now restricted to a few tributaries of Mayas River. Although the genetic diversity values of populations in OCU II were higher than those of OCU I, the possibility of a bottleneck followed by a population expansion cannot be rejected for these populations, despite a decrease in the number of individuals observed in recent years (Doadrio et al., 2011). Exotic species, such as *Lepomis gibbosus* and *Micropterus salmoides*, have colonized the upper parts of the rivers covered by OCU II, which may be one of the main causes for the decline in the number of *C*. *vettonica* in these rivers. Other potential causes of this decline remain unknown.

For OCU IV, there was no genetic distinction between the non‐introgressed (upper Erjas) and putatively introgressed (Ponsul, Aravil, and lower Erjas) populations. Despite this, any management plan must take into consideration this potential introgression as the indiscriminate mixing of individuals from these two groups could lead to genetic introgression with *C*. *paludica* throughout the entire area of OCU IV. *Cobitis paludica*, which is more of a generalist than *C*. *vettonica*, is widely distributed throughout the Iberian Peninsula, occupying a great variety of ecological niches (Doadrio et al., 2011). Thus, genetic introgression by *C*. *paludica* in sympatric localities would likely prove disadvantageous for *C*. *vettonica* in general, as two of the four OCUs (III and IV) would be affected by this phenomenon. It could lead to the extinction of *C*. *vettonica* in these areas or to adaptive variations, resulting in a major loss of the genetic diversity of this species.

## CONFLICT OF INTEREST

The authors declare no conflict of interest.

## AUTHOR CONTRIBUTIONS

**Andrea Corral‐Lou:** Conceptualization (lead); Data curation (lead); Formal analysis (lead); Methodology (equal); Resources (lead); Software (lead); Validation (lead); Visualization (lead); Writing – original draft (lead); Writing – review & editing (lead). **Silvia Perea:** Conceptualization (equal); Data curation (equal); Formal analysis (supporting); Methodology (supporting); Supervision (supporting); Writing – review & editing (equal). **Anabel Perdices:** Resources (supporting); Supervision (supporting); Writing – review & editing (supporting). **Ignacio Doadrio:** Conceptualization (lead); Data curation (equal); Funding acquisition (lead); Investigation (lead); Methodology (lead); Resources (equal); Supervision (lead); Validation (lead); Visualization (equal); Writing – review & editing (equal).

## Supporting information

Supplementary Material

## Data Availability

The new sequences of the mitochondrial (*MT*‐*CYTB*; OM234794‐OM235001) and nuclear (*RAG1*; OM235002‐OM235091) markers obtained from this study are available in GenBank. The SNP data have been deposited in VCF format in Figshare (https://doi.org/10.6084/m9.figshare.18778148.v1).
