# Supplementary material for: Quaternary geomorphological and climatic changes associated with the diversification of Iberian freshwater fishes: The case of the genus Cobitis (Cypriniformes, Cobitidae)
Source: Ecol Evol. 2022 Mar 1;12(3):e8635. doi: 10.1002/ece3.8635 (PMC8888266; doi:10.1002/ece3.8635)
Supplement: Supplementary file 1 — Supplementary Material [file ECE3-12-e8635-s001.docx]

**Supplementary Information for:**

**Table S1.** Information on the individuals included in the present study. The following parameters are included in the table: Identification code used in this study (ID), species, the origin of the genetic information used (Source), identification number of the DNA and Tissue Collection at the National Museum of Natural Sciences of Madrid (MNCN/DNA), the date when the sample was taken (Date), the genetic marker studied indicated with an X (for SNP, MT-CYB and RAG1), the GenBank access number for MT-CYB and RAG1 (GenBank_ID RAG1 and GenBank_ID MT-CYB respectively), the locality (Locality) and the number of the locality referenced in Table 1 (Number of the locality).

| **ID** | **Species** | **Source** | **MNCN/ADN** | **Date** | **SNPs** | ***MT-CYB*** | ***RAG1*** | **GenBank_ID (*MT-CYB*)** | **GenBank_ID (*RAG1*)** | **Locality** | **Number of  the locality** |
| --- | --- | --- | --- | --- | --- | --- | --- | --- | --- | --- | --- |
| AT15755 | *Cobitis paludica* | This study | 119350 | 04/05/2010 | X | X |  | OM234965 |  | Spain: Castañar de Ibor, Tagus Basin, lower Tagus Sub-basin, Ibor River | 38 |
| AT15756 | *Cobitis paludica* | This study | 119351 | 04/05/2010 | X | X |  | OM234966 |  | Spain: Castañar de Ibor, Tagus Basin, lower Tagus Sub-basin, Ibor River | 38 |
| AT15757 | *Cobitis paludica* | This study | 119352 | 04/05/2010 | X | X |  | OM234967 |  | Spain: Castañar de Ibor, Tagus Basin, lower Tagus Sub-basin, Ibor River | 38 |
| AT15826 | *Cobitis vettonica* | This study | 119394 | 05/05/2010 |  | X | X | OM234930 | OM235035 | Spain: Zarza de Granadilla, Tagus Basin, eastern Alagón Sub-basin, Caparro River | 38 |
| AT15827 | *Cobitis vettonica* | This study | 119395 | 05/05/2010 |  | X |  | OM234931 |  | Spain: Zarza de Granadilla, Tagus Basin, eastern Alagón Sub-basin, Caparro River | 34 |
| AT15828 | *Cobitis vettonica* | This study | 119396 | 05/05/2010 |  | X |  | OM234932 |  | Spain: Zarza de Granadilla, Tagus Basin, eastern Alagón Sub-basin, Caparro River | 34 |
| AT15829 | *Cobitis vettonica* | This study | 119397 | 05/05/2010 |  | X | X | OM234933 | OM235036 | Spain: Zarza de Granadilla, Tagus Basin, eastern Alagón Sub-basin, Caparro River | 34 |
| AT15831 | *Cobitis vettonica* | This study | 119398 | 05/05/2010 |  | X | X | OM234934 | OM235037 | Spain: Zarza de Granadilla, Tagus Basin, eastern Alagón Sub-basin, Caparro River | 34 |
| AT15832 | *Cobitis vettonica* | This study | 119399 | 05/05/2010 |  | X | X | OM234935 | OM235038 | Spain: Zarza de Granadilla, Tagus Basin, eastern Alagón Sub-basin, Caparro River | 34 |
| AT15833 | *Cobitis vettonica* | This study | 119400 | 05/05/2010 |  | X | X | OM234936 | OM235039 | Spain: Zarza de Granadilla, Tagus Basin, eastern Alagón Sub-basin, Caparro River | 37 |
| AT15834 | *Cobitis vettonica* | This study | 119401 | 05/05/2010 |  | X |  | OM234937 |  | Spain: Zarza de Granadilla, Tagus Basin, eastern Alagón Sub-basin, Caparro River | 37 |
| AT15835 | *Cobitis vettonica* | This study | 119402 | 05/05/2010 |  | X |  | OM234938 |  | Spain: Zarza de Granadilla, Tagus Basin, eastern Alagón Sub-basin, Caparro River | 37 |
| AT15836 | *Cobitis vettonica* | This study | 119403 | 05/05/2010 |  | X |  | OM234939 |  | Spain: Zarza de Granadilla, Tagus Basin, eastern Alagón Sub-basin, Caparro River | 37 |
| AT15837 | *Cobitis vettonica* | This study | 119404 | 05/05/2010 |  | X |  | OM234940 |  | Spain: Zarza de Granadilla, Tagus Basin, eastern Alagón Sub-basin, Caparro River | 37 |
| AT15838 | *Cobitis vettonica* | This study | 119405 | 05/05/2010 |  | X |  | OM234941 |  | Spain: Zarza de Granadilla, Tagus Basin, eastern Alagón Sub-basin, Caparro River | 29 |
| AT15839 | *Cobitis vettonica* | This study | 119406 | 05/05/2010 |  | X |  | OM234942 |  | Spain: Zarza de Granadilla, Tagus Basin, eastern Alagón Sub-basin, Caparro River | 29 |
| AT15866 | *Cobitis vettonica* | This study | 119407 | 05/05/2010 |  | X | X | OM234919 | OM235044 | Spain: Vegas de Coria, Tagus Basin, eastern Alagón Sub-basin, Hurdano River | 29 |
| AT15905 | *Cobitis vettonica* | This study | 119408 | 06/05/2010 |  | X | X | OM234860 | OM235058 | Spain: Valverde del Fresno, Tagus Basin, upper Erjas Sub-basin, Erjas River | 29 |
| AT15906 | *Cobitis vettonica* | This study | 119409 | 06/05/2010 |  | X | X | OM234861 | OM235059 | Spain: Valverde del Fresno, Tagus Basin, upper Erjas Sub-basin, Erjas River | 29 |
| AT15907 | *Cobitis vettonica* | This study | 119410 | 06/05/2010 |  | X | X | OM234862 | OM235060 | Spain: Valverde del Fresno, Tagus Basin, upper Erjas Sub-basin, Erjas River | 36 |
| AT15908 | *Cobitis vettonica* | This study | 119411 | 06/05/2010 |  | X | X | OM234863 | OM235061 | Spain: Valverde del Fresno, Tagus Basin, upper Erjas Sub-basin, Erjas River | 36 |
| AT15909 | *Cobitis vettonica* | This study | 119412 | 06/05/2010 |  | X |  | OM234864 |  | Spain: Valverde del Fresno, Tagus Basin, upper Erjas Sub-basin, Erjas River | 36 |
| AT15910 | *Cobitis vettonica* | This study | 119413 | 06/05/2010 |  | X |  | OM234865 |  | Spain: Valverde del Fresno, Tagus Basin, upper Erjas Sub-basin, Erjas River | 36 |
| AT15911 | *Cobitis vettonica* | This study | 119414 | 06/05/2010 |  | X |  | OM234850 |  | Spain: Hoyos, Tagus Basin, western Alagón Sub-basin, Acebo River | 36 |
| AT15919 | *Cobitis vettonica* | This study | 119415 | 06/05/2010 |  | X |  | OM234847 |  | Spain: Gata, Tagus Basin, western Alagón Sub-basin, San Blas River | 26 |
| AT15930 | *Cobitis vettonica* | This study | 119416 | 06/05/2010 |  | X |  | OM234825 |  | Spain: Gata, Tagus Basin, western Alagón Sub-basin, Gata River | 26 |
| AT15931 | *Cobitis vettonica* | This study | 119417 | 06/05/2010 |  | X |  | OM234826 |  | Spain: Gata, Tagus Basin, western Alagón Sub-basin, Gata River | 26 |
| AT15932 | *Cobitis vettonica* | This study | 119418 | 06/05/2010 |  | X |  | OM234827 |  | Spain: Gata, Tagus Basin, western Alagón Sub-basin, Gata River | 26 |
| AT15933 | *Cobitis vettonica* | This study | 119419 | 06/05/2010 |  | X |  | OM234828 |  | Spain: Gata, Tagus Basin, western Alagón Sub-basin, Gata River | 26 |
| AT15934 | *Cobitis vettonica* | This study | 119420 | 06/05/2010 |  | X |  | OM234829 |  | Spain: Gata, Tagus Basin, western Alagón Sub-basin, Gata River | 25 |
| AT15935 | *Cobitis vettonica* | This study | 119421 | 06/05/2010 | X | X | X | OM234830 | OM235007 | Spain: Gata, Tagus Basin, western Alagón Sub-basin, Gata River | 4 |
| AT15936 | *Cobitis vettonica* | This study | 119422 | 06/05/2010 | X | X | X | OM234831 | OM235008 | Spain: Gata, Tagus Basin, western Alagón Sub-basin, Gata River | 4 |
| AT15937 | *Cobitis vettonica* | This study | 119423 | 06/05/2010 | X | X | X | OM234832 | OM235009 | Spain: Gata, Tagus Basin, western Alagón Sub-basin, Gata River | 4 |
| AT15938 | *Cobitis vettonica* | This study | 119424 | 06/05/2010 | X | X | X | OM234833 | OM235010 | Spain: Gata, Tagus Basin, western Alagón Sub-basin, Gata River | 4 |
| AT15939 | *Cobitis vettonica* | This study | 119425 | 06/05/2010 | X | X | X | OM234834 | OM235011 | Spain: Gata, Tagus Basin, western Alagón Sub-basin, Gata River | 14 |
| AT15985 | *Cobitis paludica* | This study | 119353 | 13/05/2010 | X | X |  | OM234968 |  | Spain: Trujillo, Tagus Basin, Almonte Sub-basin, Tamuja River | 14 |
| AT15987 | *Cobitis paludica* | This study | 119354 | 13/05/2010 | X | X |  | OM234969 |  | Spain: Trujillo, Tagus Basin, Almonte Sub-basin, Tamuja River | 14 |
| AT16031 | *Cobitis paludica* | This study | 119355 | 13/05/2010 | X | X |  | OM234964 |  | Spain: Membrío, Tagus Basin, Salor Sub-basin, Salor River | 14 |
| AT20195 | *Cobitis vettonica* | This study | 119426 | 23/09/2010 | X | X |  | OM234950 |  | Spain: Navaconcejo, Tagus Basin, eastern Alagón Sub-basin, Jerte River | 14 |
| AT20196 | *Cobitis vettonica* | This study | 119427 | 23/09/2010 | X | X |  | OM234951 |  | Spain: Navaconcejo, Tagus Basin, eastern Alagón Sub-basin, Jerte River | 6 |
| AT20197 | *Cobitis vettonica* | This study | 119428 | 23/09/2010 | X | X |  | OM234952 |  | Spain: Navaconcejo, Tagus Basin, eastern Alagón Sub-basin, Jerte River | 6 |
| AT20198 | *Cobitis vettonica* | This study | 119572 | 23/09/2010 | X |  |  |  |  | Spain: Navaconcejo, Tagus Basin, eastern Alagón Sub-basin, Jerte River | 6 |
| AT20199 | *Cobitis vettonica* | This study | 119429 | 23/09/2010 | X | X |  | OM234953 |  | Spain: Navaconcejo, Tagus Basin, eastern Alagón Sub-basin, Jerte River | 6 |
| AT20201 | *Cobitis vettonica* | This study | 119430 | 23/09/2010 | X | X |  | OM234954 |  | Spain: Navaconcejo, Tagus Basin, eastern Alagón Sub-basin, Jerte River | 5 |
| AT20202 | *Cobitis vettonica* | This study | 119431 | 23/09/2010 | X | X |  | OM234955 |  | Spain: Navaconcejo, Tagus Basin, eastern Alagón Sub-basin, Jerte River | 13 |
| AT20203 | *Cobitis vettonica* | This study | 119432 | 23/09/2010 | X | X |  | OM234956 |  | Spain: Navaconcejo, Tagus Basin, eastern Alagón Sub-basin, Jerte River | 13 |
| AT20204 | *Cobitis vettonica* | This study | 119433 | 23/09/2010 | X | X |  | OM234957 |  | Spain: Navaconcejo, Tagus Basin, eastern Alagón Sub-basin, Jerte River | 23 |
| AT20206 | *Cobitis vettonica* | This study | 119573 | 23/09/2010 | X |  |  |  |  | Spain: Navaconcejo, Tagus Basin, eastern Alagón Sub-basin, Jerte River | 23 |
| AT20372 | *Cobitis paludica* | This study | 119356 | 24/09/2010 | X | X |  | OM234977 |  | Spain: La Iglesuela del Tiétar, Tagus Basin, Tiétar Sub-basin, Tiétar River | 23 |
| AT20373 | *Cobitis paludica* | This study | 119357 | 24/09/2010 | X | X |  | OM234978 |  | Spain: La Iglesuela del Tiétar, Tagus Basin, Tiétar Sub-basin, Tiétar River | 23 |
| AT20374 | *Cobitis paludica* | This study | 119358 | 24/09/2010 | X | X |  | OM234979 |  | Spain: La Iglesuela del Tiétar, Tagus Basin, Tiétar Sub-basin, Tiétar River | 23 |
| AT20375 | *Cobitis paludica* | This study | 119359 | 24/09/2010 | X | X |  | OM234980 |  | Spain: La Iglesuela del Tiétar, Tagus Basin, Tiétar Sub-basin, Tiétar River | 23 |
| AT20376 | *Cobitis paludica* | This study | 119360 | 24/09/2010 | X | X |  | OM234981 |  | Spain: La Iglesuela del Tiétar, Tagus Basin, Tiétar Sub-basin, Tiétar River | 23 |
| AT20377 | *Cobitis paludica* | This study | 119361 | 24/09/2010 | X | X |  | OM234982 |  | Spain: La Iglesuela del Tiétar, Tagus Basin, Tiétar Sub-basin, Tiétar River | 23 |
| AT2313 | *Cobitis vettonica* | This study | 119434 | 04/06/2004 |  | X | X | OM234851 | OM235054 | Spain: Cilleros, Tagus Basin, upper Erjas Sub-basin, Trevejana River | 23 |
| AT2314 | *Cobitis vettonica* | This study | 119435 | 04/06/2004 |  | X | X | OM234852 | OM235055 | Spain: Cilleros, Tagus Basin, upper Erjas Sub-basin, Trevejana River | 18 |
| AT2315 | *Cobitis vettonica* | This study | 119436 | 04/06/2004 |  | X | X | OM234853 | OM235056 | Spain: Cilleros, Tagus Basin, upper Erjas Sub-basin, Trevejana River | 18 |
| AT2316 | *Cobitis vettonica* | This study | 119437 | 04/06/2004 |  | X | X | OM234854 | OM235057 | Spain: Cilleros, Tagus Basin, upper Erjas Sub-basin, Trevejana River | 18 |
| AT2321 | *Cobitis vettonica* | This study | 119438 | 04/06/2004 |  | X | X | OM234866 | OM235067 | Spain: San Martín de Trevejo, Tagus Basin, upper Erjas Sub-basin, San Martin River | 18 |
| AT2322 | *Cobitis vettonica* | This study | 119439 | 04/06/2004 |  | X |  | OM234867 |  | Spain: San Martín de Trevejo, Tagus Basin, upper Erjas Sub-basin, San Martin River | 22 |
| AT2323 | *Cobitis vettonica* | This study | 119440 | 04/06/2004 |  | X |  | OM234868 |  | Spain: San Martín de Trevejo, Tagus Basin, upper Erjas Sub-basin, San Martin River | 22 |
| AT2324 | *Cobitis vettonica* | This study | 119441 | 04/06/2004 |  | X |  | OM234869 |  | Spain: San Martín de Trevejo, Tagus Basin, upper Erjas Sub-basin, San Martin River | 22 |
| AT2325 | *Cobitis vettonica* | This study | 119442 | 04/06/2004 |  | X |  | OM234870 |  | Spain: San Martín de Trevejo, Tagus Basin, upper Erjas Sub-basin, San Martin River | 22 |
| AT2326 | *Cobitis vettonica* | This study | 119443 | 04/06/2004 |  | X |  | OM234871 |  | Spain: San Martín de Trevejo, Tagus Basin, upper Erjas Sub-basin, San Martin River | 22 |
| AT2355 | *Cobitis vettonica* | This study | 119444 | 04/06/2004 |  | X |  | OM234849 |  | Spain: Hoyos, Tagus Basin, western Alagón Sub-basin, Acebo River | 21 |
| AT2359 | *Cobitis vettonica* | This study | 119445 | 04/06/2004 |  | X |  | OM234816 |  | Spain: Gata, Tagus Basin, western Alagón Sub-basin, Gata River | 21 |
| AT2360 | *Cobitis vettonica* | This study | 119446 | 04/06/2004 |  | X |  | OM234817 |  | Spain: Gata, Tagus Basin, western Alagón Sub-basin, Gata River | 21 |
| AT2361 | *Cobitis vettonica* | This study | 119447 | 04/06/2004 |  | X |  | OM234818 |  | Spain: Gata, Tagus Basin, western Alagón Sub-basin, Gata River | 21 |
| AT2362 | *Cobitis vettonica* | This study | 119448 | 04/06/2004 |  | X |  | OM234819 |  | Spain: Gata, Tagus Basin, western Alagón Sub-basin, Gata River | 21 |
| AT2372 | *Cobitis vettonica* | This study | 119449 | 04/06/2004 |  | X | X | OM234835 | OM235012 | Spain: Cadalso de Gata, Tagus Basin, western Alagón Sub-basin, Árrago River | 21 |
| AT2373 | *Cobitis vettonica* | This study | 119450 | 04/06/2004 |  | X |  | OM234836 |  | Spain: Cadalso de Gata, Tagus Basin, western Alagón Sub-basin, Árrago River | 21 |
| AT2374 | *Cobitis vettonica* | This study | 119451 | 04/06/2004 |  | X |  | OM234837 |  | Spain: Cadalso de Gata, Tagus Basin, western Alagón Sub-basin, Árrago River | 21 |
| AT2375 | *Cobitis vettonica* | This study | 119452 | 04/06/2004 |  | X | X | OM234838 | OM235013 | Spain: Cadalso de Gata, Tagus Basin, western Alagón Sub-basin, Árrago River | 21 |
| AT2376 | *Cobitis vettonica* | This study | 119453 | 04/06/2004 |  | X |  | OM234839 |  | Spain: Cadalso de Gata, Tagus Basin, western Alagón Sub-basin, Árrago River | 4 |
| AT27309 | *Cobitis vettonica* | This study | 119454 | 20/11/2015 | X | X | X | OM234900 | OM235045 | Spain: Sotoserrano, Tagus Basin, eastern Alagón Sub-basin, Cuerpo de Hombre River | 4 |
| AT27310 | *Cobitis vettonica* | This study | 119455 | 20/11/2015 | X | X | X | OM234901 | OM235046 | Spain: Sotoserrano, Tagus Basin, eastern Alagón Sub-basin, Cuerpo de Hombre River | 4 |
| AT27311 | *Cobitis vettonica* | This study | 119456 | 20/11/2015 | X | X | X | OM234902 | OM235047 | Spain: Sotoserrano, Tagus Basin, eastern Alagón Sub-basin, Cuerpo de Hombre River | 4 |
| AT27312 | *Cobitis vettonica* | This study | 119457 | 20/11/2015 | X | X | X | OM234903 | OM235048 | Spain: Sotoserrano, Tagus Basin, eastern Alagón Sub-basin, Cuerpo de Hombre River | 4 |
| AT27313 | *Cobitis vettonica* | This study | 119458 | 20/11/2015 | X | X | X | OM234904 | OM235049 | Spain: Sotoserrano, Tagus Basin, eastern Alagón Sub-basin, Cuerpo de Hombre River | 26 |
| AT27314 | *Cobitis vettonica* | This study | 119459 | 20/11/2015 | X | X |  | OM234905 |  | Spain: Sotoserrano, Tagus Basin, eastern Alagón Sub-basin, Cuerpo de Hombre River | 26 |
| AT27315 | *Cobitis vettonica* | This study | 119460 | 20/11/2015 | X | X |  | OM234906 |  | Spain: Sotoserrano, Tagus Basin, eastern Alagón Sub-basin, Cuerpo de Hombre River | 27 |
| AT27316 | *Cobitis vettonica* | This study | 119461 | 20/11/2015 | X | X |  | OM234907 |  | Spain: Sotoserrano, Tagus Basin, eastern Alagón Sub-basin, Cuerpo de Hombre River | 27 |
| AT27317 | *Cobitis vettonica* | This study | 119462 | 20/11/2015 | X | X |  | OM234908 |  | Spain: Sotoserrano, Tagus Basin, eastern Alagón Sub-basin, Cuerpo de Hombre River | 27 |
| AT27318 | *Cobitis vettonica* | This study | 119463 | 20/11/2015 | X | X |  | OM234909 |  | Spain: Sotoserrano, Tagus Basin, eastern Alagón Sub-basin, Cuerpo de Hombre River | 27 |
| AT27319 | *Cobitis vettonica* | This study | 119464 | 20/11/2015 |  | X | X | OM234910 | OM235050 | Spain: Sotoserrano, Tagus Basin, eastern Alagón Sub-basin, Cuerpo de Hombre River | 27 |
| AT27320 | *Cobitis vettonica* | This study | 119465 | 20/11/2015 |  | X |  | OM234911 |  | Spain: Sotoserrano, Tagus Basin, eastern Alagón Sub-basin, Cuerpo de Hombre River | 26 |
| AT27321 | *Cobitis vettonica* | This study | 119466 | 20/11/2015 |  | X |  | OM234912 |  | Spain: Sotoserrano, Tagus Basin, eastern Alagón Sub-basin, Cuerpo de Hombre River | 26 |
| AT27322 | *Cobitis vettonica* | This study | 119467 | 20/11/2015 |  | X | X | OM234913 | OM235051 | Spain: Sotoserrano, Tagus Basin, eastern Alagón Sub-basin, Cuerpo de Hombre River | 16 |
| AT27323 | *Cobitis vettonica* | This study | 119468 | 20/11/2015 |  | X | X | OM234914 | OM235052 | Spain: Sotoserrano, Tagus Basin, eastern Alagón Sub-basin, Cuerpo de Hombre River | 13 |
| AT27324 | *Cobitis vettonica* | This study | 119469 | 20/11/2015 |  | X | X | OM234915 | OM235053 | Spain: Sotoserrano, Tagus Basin, eastern Alagón Sub-basin, Cuerpo de Hombre River | 20 |
| AT27325 | *Cobitis vettonica* | This study | 119470 | 20/11/2015 |  | X |  | OM234916 |  | Spain: Sotoserrano, Tagus Basin, eastern Alagón Sub-basin, Cuerpo de Hombre River | 13 |
| AT27326 | *Cobitis vettonica* | This study | 119471 | 20/11/2015 |  | X |  | OM234917 |  | Spain: Sotoserrano, Tagus Basin, eastern Alagón Sub-basin, Cuerpo de Hombre River | 13 |
| AT27327 | *Cobitis vettonica* | This study | 119472 | 20/11/2015 |  | X |  | OM234918 |  | Spain: Sotoserrano, Tagus Basin, eastern Alagón Sub-basin, Cuerpo de Hombre River | 19 |
| AT27342 | *Cobitis vettonica* | This study | 119473 | 19/11/2015 |  | X |  | OM234795 |  | Spain: El Sahugo, Duero Basin, Águeda Sub-basin, Mayas River | 24 |
| AT27343 | *Cobitis vettonica* | This study | 119474 | 19/11/2015 |  | X |  | OM234796 |  | Spain: El Sahugo, Duero Basin, Águeda Sub-basin, Mayas River | 4 |
| AT27344 | *Cobitis vettonica* | This study | 119475 | 19/11/2015 |  | X |  | OM234797 |  | Spain: El Sahugo, Duero Basin, Águeda Sub-basin, Mayas River | 13 |
| AT27345 | *Cobitis vettonica* | This study | 119476 | 19/11/2015 |  | X |  | OM234798 |  | Spain: El Sahugo, Duero Basin, Águeda Sub-basin, Mayas River | 17 |
| AT27346 | *Cobitis vettonica* | This study | 119477 | 19/11/2015 |  | X |  | OM234799 |  | Spain: El Sahugo, Duero Basin, Águeda Sub-basin, Mayas River | 3 |
| AT27347 | *Cobitis vettonica* | This study | 119478 | 19/11/2015 |  | X |  | OM234800 |  | Spain: El Sahugo, Duero Basin, Águeda Sub-basin, Mayas River | 1 |
| AT27348 | *Cobitis vettonica* | This study | 119479 | 19/11/2015 |  | X |  | OM234801 |  | Spain: El Sahugo, Duero Basin, Águeda Sub-basin, Mayas River | 1 |
| AT27349 | *Cobitis vettonica* | This study | 119480 | 19/11/2015 |  | X |  | OM234802 |  | Spain: El Sahugo, Duero Basin, Águeda Sub-basin, Mayas River | 1 |
| AT27350 | *Cobitis vettonica* | This study | 119481 | 19/11/2015 |  | X |  | OM234803 |  | Spain: El Sahugo, Duero Basin, Águeda Sub-basin, Mayas River | 1 |
| AT27351 | *Cobitis vettonica* | This study | 119482 | 19/11/2015 |  | X |  | OM234804 |  | Spain: El Sahugo, Duero Basin, Águeda Sub-basin, Mayas River | 7 |
| AT27365 | *Cobitis vettonica* | This study | 119483 | 19/11/2015 | X | X |  | OM234805 |  | Spain: El Sahugo, Duero Basin, Águeda Sub-basin, Mayas River | 7 |
| AT27366 | *Cobitis vettonica* | This study | 119484 | 19/11/2015 | X | X |  | OM234806 |  | Spain: El Sahugo, Duero Basin, Águeda Sub-basin, Mayas River | 7 |
| AT27367 | *Cobitis vettonica* | This study | 119485 | 19/11/2015 | X | X |  | OM234807 |  | Spain: El Sahugo, Duero Basin, Águeda Sub-basin, Mayas River | 7 |
| AT27368 | *Cobitis vettonica* | This study | 119486 | 19/11/2015 | X | X |  | OM234808 |  | Spain: El Sahugo, Duero Basin, Águeda Sub-basin, Mayas River | 7 |
| AT27369 | *Cobitis vettonica* | This study | 119487 | 19/11/2015 | X | X |  | OM234809 |  | Spain: El Sahugo, Duero Basin, Águeda Sub-basin, Mayas River | 7 |
| AT27370 | *Cobitis vettonica* | This study | 119488 | 19/11/2015 | X | X | X | OM234810 | OM235003 | Spain: El Sahugo, Duero Basin, Águeda Sub-basin, Mayas River | 11 |
| AT27371 | *Cobitis vettonica* | This study | 119489 | 19/11/2015 | X | X | X | OM234811 | OM235004 | Spain: El Sahugo, Duero Basin, Águeda Sub-basin, Mayas River | 11 |
| AT27372 | *Cobitis vettonica* | This study | 119490 | 19/11/2015 | X | X | X | OM234812 | OM235005 | Spain: El Sahugo, Duero Basin, Águeda Sub-basin, Mayas River | 11 |
| AT27373 | *Cobitis vettonica* | This study | 119491 | 19/11/2015 | X | X | X | OM234813 | OM235006 | Spain: El Sahugo, Duero Basin, Águeda Sub-basin, Mayas River | 11 |
| AT27374 | *Cobitis vettonica* | This study | 119492 | 19/11/2015 | X | X |  | OM234814 |  | Spain: El Sahugo, Duero Basin, Águeda Sub-basin, Mayas River | 12 |
| AT27375 | *Cobitis paludica* | This study | 119562 | 19/11/2015 | X |  |  |  |  | Spain: San Martín de Yeltes, Duero Basin, Huebra-Yeltes Sub-basin, Yeltes River | 12 |
| AT27376 | *Cobitis paludica* | This study | 119563 | 19/11/2015 | X |  |  |  |  | Spain: San Martín de Yeltes, Duero Basin, Huebra-Yeltes Sub-basin, Yeltes River | 10 |
| AT27377 | *Cobitis paludica* | This study | 119564 | 19/11/2015 | X |  |  |  |  | Spain: San Martín de Yeltes, Duero Basin, Huebra-Yeltes Sub-basin, Yeltes River | 8 |
| AT27378 | *Cobitis paludica* | This study | 119565 | 19/11/2015 | X |  |  |  |  | Spain: San Martín de Yeltes, Duero Basin, Huebra-Yeltes Sub-basin, Yeltes River | 8 |
| AT27379 | *Cobitis paludica* | This study | 119566 | 19/11/2015 | X |  |  |  |  | Spain: San Martín de Yeltes, Duero Basin, Huebra-Yeltes Sub-basin, Yeltes River | 9 |
| AT27553 | *Cobitis vettonica* | This study | 119493 | 20/11/2015 | X | X | X | OM234886 | OM235021 | Spain: Nava de Francia, Tagus Basin, eastern Alagón Sub-basin, Francia River | 9 |
| AT27554 | *Cobitis vettonica* | This study | 119494 | 20/11/2015 |  | X | X | OM234887 | OM235022 | Spain: Nava de Francia, Tagus Basin, eastern Alagón Sub-basin, Francia River | 2 |
| AT27555 | *Cobitis vettonica* | This study | 119495 | 20/11/2015 | X | X | X | OM234888 | OM235023 | Spain: Nava de Francia, Tagus Basin, eastern Alagón Sub-basin, Francia River | 3 |
| AT27556 | *Cobitis vettonica* | This study | 119496 | 20/11/2015 | X | X | X | OM234889 | OM235024 | Spain: Nava de Francia, Tagus Basin, eastern Alagón Sub-basin, Francia River | 3 |
| AT27557 | *Cobitis vettonica* | This study | 119497 | 20/11/2015 | X | X | X | OM234890 | OM235025 | Spain: Nava de Francia, Tagus Basin, eastern Alagón Sub-basin, Francia River | 3 |
| AT27558 | *Cobitis vettonica* | This study | 119498 | 20/11/2015 | X | X |  | OM234891 |  | Spain: Nava de Francia, Tagus Basin, eastern Alagón Sub-basin, Francia River | 11 |
| AT27559 | *Cobitis vettonica* | This study | 119499 | 20/11/2015 |  | X |  | OM234892 |  | Spain: Nava de Francia, Tagus Basin, eastern Alagón Sub-basin, Francia River | 12 |
| AT27560 | *Cobitis vettonica* | This study | 119500 | 20/11/2015 | X | X |  | OM234893 |  | Spain: Nava de Francia, Tagus Basin, eastern Alagón Sub-basin, Francia River | 10 |
| AT27561 | *Cobitis vettonica* | This study | 119501 | 20/11/2015 | X | X |  | OM234894 |  | Spain: Nava de Francia, Tagus Basin, eastern Alagón Sub-basin, Francia River | 30 |
| AT27562 | *Cobitis vettonica* | This study | 119502 | 20/11/2015 | X | X |  | OM234895 |  | Spain: Nava de Francia, Tagus Basin, eastern Alagón Sub-basin, Francia River | 31 |
| AT27563 | *Cobitis vettonica* | This study | 119503 | 20/11/2015 |  | X |  | OM234896 |  | Spain: Nava de Francia, Tagus Basin, eastern Alagón Sub-basin, Francia River | 1 |
| AT27564 | *Cobitis vettonica* | This study | 119504 | 20/11/2015 |  | X |  | OM234897 |  | Spain: Nava de Francia, Tagus Basin, eastern Alagón Sub-basin, Francia River | 28 |
| AT27565 | *Cobitis vettonica* | This study | 119505 | 20/11/2015 |  | X |  | OM234898 |  | Spain: Nava de Francia, Tagus Basin, eastern Alagón Sub-basin, Francia River | 35 |
| AT27566 | *Cobitis vettonica* | This study | 119506 | 20/11/2015 |  | X |  | OM234899 |  | Spain: Nava de Francia, Tagus Basin, eastern Alagón Sub-basin, Francia River | 35 |
| AT27631 | *Cobitis paludica* | This study | 119362 | 22/10/2014 | X | X | X | OM234988 | OM235078 | Spain: El cubo de don Sancho, Duero Basin, Huebra-Yeltes Sub-basin, Huebra River | 35 |
| AT27632 | *Cobitis paludica* | This study | 119363 | 22/10/2014 | X | X |  | OM234989 |  | Spain: El cubo de don Sancho, Duero Basin, Huebra-Yeltes Sub-basin, Huebra River | 33 |
| AT27633 | *Cobitis paludica* | This study | 119364 | 22/10/2014 | X | X | X | OM234990 | OM235079 | Spain: El cubo de don Sancho, Duero Basin, Huebra-Yeltes Sub-basin, Huebra River | 33 |
| AT27634 | *Cobitis paludica* | This study | 119365 | 22/10/2014 | X | X | X | OM234991 | OM235080 | Spain: El cubo de don Sancho, Duero Basin, Huebra-Yeltes Sub-basin, Huebra River | 32 |
| AT27635 | *Cobitis paludica* | This study | 119366 | 22/10/2014 | X | X | X | OM234992 | OM235081 | Spain: El cubo de don Sancho, Duero Basin, Huebra-Yeltes Sub-basin, Huebra River | 36 |
| AT27636 | *Cobitis paludica* | This study | 119367 | 22/10/2014 | X | X |  | OM234993 |  | Spain: El cubo de don Sancho, Duero Basin, Huebra-Yeltes Sub-basin, Huebra River | 36 |
| AT27637 | *Cobitis paludica* | This study | 119368 | 22/10/2014 | X | X |  | OM234994 |  | Spain: El cubo de don Sancho, Duero Basin, Huebra-Yeltes Sub-basin, Huebra River | 36 |
| AT27638 | *Cobitis paludica* | This study | 119369 | 22/10/2014 | X | X |  | OM234995 |  | Spain: El cubo de don Sancho, Duero Basin, Huebra-Yeltes Sub-basin, Huebra River | 36 |
| AT27640 | *Cobitis paludica* | This study | 119370 | 22/10/2014 | X | X |  | OM234996 |  | Spain: El cubo de don Sancho, Duero Basin, Huebra-Yeltes Sub-basin, Huebra River | 36 |
| AT27661 | *Cobitis vettonica* | This study | 119507 | 22/10/2014 |  | X | X | OM234872 | OM235026 | Spain: Santi Ibañez de la Sierra, Tagus Basin, eastern Alagón Sub-basin, Alagón River | 36 |
| AT27662 | *Cobitis vettonica* | This study | 119508 | 22/10/2014 |  | X | X | OM234873 | OM235027 | Spain: Santi Ibañez de la Sierra, Tagus Basin, eastern Alagón Sub-basin, Alagón River | 38 |
| AT27663 | *Cobitis vettonica* | This study | 119509 | 22/10/2014 |  | X | X | OM234874 | OM235028 | Spain: Santi Ibañez de la Sierra, Tagus Basin, eastern Alagón Sub-basin, Alagón River | 38 |
| AT27664 | *Cobitis vettonica* | This study | 119510 | 22/10/2014 |  | X | X | OM234875 | OM235029 | Spain: Santi Ibañez de la Sierra, Tagus Basin, eastern Alagón Sub-basin, Alagón River | 38 |
| AT27665 | *Cobitis vettonica* | This study | 119511 | 22/10/2014 |  | X | X | OM234876 | OM235030 | Spain: Santi Ibañez de la Sierra, Tagus Basin, eastern Alagón Sub-basin, Alagón River | 38 |
| AT27666 | *Cobitis vettonica* | This study | 119512 | 22/10/2014 | X | X |  | OM234877 |  | Spain: Santi Ibañez de la Sierra, Tagus Basin, eastern Alagón Sub-basin, Alagón River | 38 |
| AT27667 | *Cobitis vettonica* | This study | 119513 | 22/10/2014 | X | X |  | OM234878 |  | Spain: Santi Ibañez de la Sierra, Tagus Basin, eastern Alagón Sub-basin, Alagón River | 33 |
| AT27668 | *Cobitis vettonica* | This study | 119514 | 22/10/2014 | X | X |  | OM234879 |  | Spain: Santi Ibañez de la Sierra, Tagus Basin, eastern Alagón Sub-basin, Alagón River | 33 |
| AT27669 | *Cobitis vettonica* | This study | 119515 | 22/10/2014 | X | X |  | OM234880 |  | Spain: Santi Ibañez de la Sierra, Tagus Basin, eastern Alagón Sub-basin, Alagón River | 32 |
| AT27670 | *Cobitis vettonica* | This study | 119516 | 22/10/2014 |  | X | X | OM234881 | OM235031 | Spain: Santi Ibañez de la Sierra, Tagus Basin, eastern Alagón Sub-basin, Alagón River | 26 |
| AT27671 | *Cobitis vettonica* | This study | 119517 | 22/10/2014 |  | X |  | OM234882 |  | Spain: Santi Ibañez de la Sierra, Tagus Basin, eastern Alagón Sub-basin, Alagón River | 26 |
| AT27672 | *Cobitis vettonica* | This study | 119518 | 22/10/2014 |  | X | X | OM234883 | OM235032 | Spain: Santi Ibañez de la Sierra, Tagus Basin, eastern Alagón Sub-basin, Alagón River | 26 |
| AT27673 | *Cobitis vettonica* | This study | 119519 | 22/10/2014 |  | X | X | OM234884 | OM235033 | Spain: Santi Ibañez de la Sierra, Tagus Basin, eastern Alagón Sub-basin, Alagón River | 26 |
| AT27674 | *Cobitis vettonica* | This study | 119520 | 22/10/2014 |  | X | X | OM234885 | OM235034 | Spain: Santi Ibañez de la Sierra, Tagus Basin, eastern Alagón Sub-basin, Alagón River | 26 |
| AT2874 | *Cobitis vettonica* | This study | 119521 | 26/06/2005 |  | X |  | OM234815 |  | Spain: Descargamaría, Duero Basin, Águeda Sub-basin, Malena River | 26 |
| AT6689 | *Cobitis vettonica* | This study | 119522 | 20/03/2009 | X | X | X | OM234855 | OM235062 | Spain: Valverde del Fresno, Tagus Basin, upper Erjas Sub-basin, Erjas River | 26 |
| AT6690 | *Cobitis vettonica* | This study | 119523 | 20/03/2009 | X | X | X | OM234856 | OM235063 | Spain: Valverde del Fresno, Tagus Basin, upper Erjas Sub-basin, Erjas River | 26 |
| AT6691 | *Cobitis vettonica* | This study | 119524 | 20/03/2009 | X | X | X | OM234857 | OM235064 | Spain: Valverde del Fresno, Tagus Basin, upper Erjas Sub-basin, Erjas River | 4 |
| AT6692 | *Cobitis vettonica* | This study | 119525 | 20/03/2009 | X | X | X | OM234858 | OM235065 | Spain: Valverde del Fresno, Tagus Basin, upper Erjas Sub-basin, Erjas River | 4 |
| AT6699 | *Cobitis vettonica* | This study | 119526 | 20/03/2009 | X | X | X | OM234859 | OM235066 | Spain: Valverde del Fresno, Tagus Basin, upper Erjas Sub-basin, Erjas River | 16 |
| AT6734 | *Cobitis vettonica* | This study | 119527 | 20/03/2009 |  | X |  | OM234920 |  | Spain: Zarza de Granadilla, Tagus Basin, eastern Alagón Sub-basin, Caparro River | 15 |
| AT6736 | *Cobitis vettonica* | This study | 119528 | 20/03/2009 |  | X |  | OM234921 |  | Spain: Zarza de Granadilla, Tagus Basin, eastern Alagón Sub-basin, Caparro River | 14 |
| AT6737 | *Cobitis vettonica* | This study | 119529 | 20/03/2009 |  | X | X | OM234922 | OM235040 | Spain: Zarza de Granadilla, Tagus Basin, eastern Alagón Sub-basin, Caparro River | 14 |
| AT6738 | *Cobitis vettonica* | This study | 119530 | 20/03/2009 |  | X |  | OM234923 |  | Spain: Zarza de Granadilla, Tagus Basin, eastern Alagón Sub-basin, Caparro River | 14 |
| AT6739 | *Cobitis vettonica* | This study | 119531 | 20/03/2009 |  | X |  | OM234924 |  | Spain: Zarza de Granadilla, Tagus Basin, eastern Alagón Sub-basin, Caparro River | 14 |
| AT6740 | *Cobitis vettonica* | This study | 119532 | 20/03/2009 |  | X | X | OM234925 | OM235041 | Spain: Zarza de Granadilla, Tagus Basin, eastern Alagón Sub-basin, Caparro River | 14 |
| AT6741 | *Cobitis vettonica* | This study | 119533 | 20/03/2009 |  | X |  | OM234943 |  | Spain: Navaconcejo, Tagus Basin, eastern Alagón Sub-basin, Jerte River | 27 |
| AT6743 | *Cobitis vettonica* | This study | 119534 | 20/03/2009 |  | X |  | OM234944 |  | Spain: Navaconcejo, Tagus Basin, eastern Alagón Sub-basin, Jerte River | 27 |
| AT6746 | *Cobitis vettonica* | This study | 119535 | 20/03/2009 |  | X | X | OM234945 | OM235016 | Spain: Navaconcejo, Tagus Basin, eastern Alagón Sub-basin, Jerte River | 27 |
| AT6747 | *Cobitis vettonica* | This study | 119536 | 20/03/2009 |  | X | X | OM234946 | OM235017 | Spain: Navaconcejo, Tagus Basin, eastern Alagón Sub-basin, Jerte River | 27 |
| AT6748 | *Cobitis vettonica* | This study | 119537 | 20/03/2009 |  | X | X | OM234947 | OM235018 | Spain: Navaconcejo, Tagus Basin, eastern Alagón Sub-basin, Jerte River | 27 |
| AT6749 | *Cobitis vettonica* | This study | 119538 | 20/03/2009 |  | X | X | OM234948 | OM235019 | Spain: Navaconcejo, Tagus Basin, eastern Alagón Sub-basin, Jerte River | 27 |
| AT6750 | *Cobitis vettonica* | This study | 119539 | 20/03/2009 |  | X | X | OM234949 | OM235020 | Spain: Navaconcejo, Tagus Basin, eastern Alagón Sub-basin, Jerte River | 27 |
| AT6834 | *Cobitis vettonica* | This study | 119567 | 21/03/2009 | X |  |  |  |  | Spain: Zarza de Granadilla, Tagus Basin, eastern Alagón Sub-basin, Caparro River | 27 |
| AT6835 | *Cobitis vettonica* | This study | 119540 | 21/03/2009 | X | X | X | OM234926 | OM235043 | Spain: Zarza de Granadilla, Tagus Basin, eastern Alagón Sub-basin, Caparro River | 5 |
| AT6836 | *Cobitis vettonica* | This study | 119568 | 21/03/2009 | X |  |  |  |  | Spain: Zarza de Granadilla, Tagus Basin, eastern Alagón Sub-basin, Caparro River | 5 |
| AT6837 | *Cobitis vettonica* | This study | 119541 | 21/03/2009 | X | X | X | OM234927 | OM235042 | Spain: Zarza de Granadilla, Tagus Basin, eastern Alagón Sub-basin, Caparro River | 5 |
| AT6838 | *Cobitis vettonica* | This study | 119542 | 21/03/2009 | X | X |  | OM234928 |  | Spain: Zarza de Granadilla, Tagus Basin, eastern Alagón Sub-basin, Caparro River | 5 |
| AT6839 | *Cobitis vettonica* | This study | 119569 | 21/03/2009 | X |  |  |  |  | Spain: Zarza de Granadilla, Tagus Basin, eastern Alagón Sub-basin, Caparro River | 5 |
| AT6840 | *Cobitis vettonica* | This study | 119543 | 21/03/2009 |  | X |  | OM234929 |  | Spain: Zarza de Granadilla, Tagus Basin, eastern Alagón Sub-basin, Caparro River | 16 |
| AT6841 | *Cobitis vettonica* | This study | 119570 | 21/03/2009 | X |  |  |  |  | Spain: Zarza de Granadilla, Tagus Basin, eastern Alagón Sub-basin, Caparro River | 14 |
| AT6843 | *Cobitis vettonica* | This study | 119571 | 21/03/2009 | X |  |  |  |  | Spain: Zarza de Granadilla, Tagus Basin, eastern Alagón Sub-basin, Caparro River | 14 |
| AT6865 | *Cobitis vettonica* | This study | 119544 | 22/03/2009 |  | X |  | OM234820 |  | Spain: Gata, Tagus Basin, western Alagón Sub-basin, Gata River | 14 |
| AT6866 | *Cobitis vettonica* | This study | 119545 | 22/03/2009 |  | X |  | OM234821 |  | Spain: Gata, Tagus Basin, western Alagón Sub-basin, Gata River | 14 |
| AT6867 | *Cobitis vettonica* | This study | 119546 | 22/03/2009 |  | X |  | OM234822 |  | Spain: Gata, Tagus Basin, western Alagón Sub-basin, Gata River | 13 |
| AT6868 | *Cobitis vettonica* | This study | 119547 | 22/03/2009 |  | X |  | OM234823 |  | Spain: Gata, Tagus Basin, western Alagón Sub-basin, Gata River | 13 |
| AT6869 | *Cobitis vettonica* | This study | 119548 | 22/03/2009 |  | X |  | OM234824 |  | Spain: Gata, Tagus Basin, western Alagón Sub-basin, Gata River | 13 |
| AT6892 | *Cobitis vettonica* | This study | 119549 | 22/03/2009 |  | X | X | OM234848 | OM235015 | Spain: Hoyos, Tagus Basin, western Alagón Sub-basin, Acebo River | 23 |
| AT6922 | *Cobitis vettonica* | This study | 119550 | 22/03/2009 |  | X |  | OM234840 |  | Spain: Cadalso de Gata, Tagus Basin, western Alagón Sub-basin, Árrago River | 23 |
| AT6923 | *Cobitis vettonica* | This study | 119551 | 22/03/2009 |  | X |  | OM234841 |  | Spain: Cadalso de Gata, Tagus Basin, western Alagón Sub-basin, Árrago River | 23 |
| AT6924 | *Cobitis vettonica* | This study | 119552 | 22/03/2009 |  | X |  | OM234842 |  | Spain: Cadalso de Gata, Tagus Basin, western Alagón Sub-basin, Árrago River | 23 |
| AT6925 | *Cobitis vettonica* | This study | 119553 | 22/03/2009 |  | X | X | OM234843 | OM235014 | Spain: Cadalso de Gata, Tagus Basin, western Alagón Sub-basin, Árrago River | 23 |
| AT6926 | *Cobitis vettonica* | This study | 119554 | 22/03/2009 |  | X |  | OM234844 |  | Spain: Cadalso de Gata, Tagus Basin, western Alagón Sub-basin, Árrago River | 23 |
| AT6927 | *Cobitis vettonica* | This study | 119555 | 22/03/2009 |  | X |  | OM234845 |  | Spain: Cadalso de Gata, Tagus Basin, western Alagón Sub-basin, Árrago River | 23 |
| AT7308 | *Cobitis paludica* | This study | 119371 | 18/04/2009 | X | X | X | OM234972 | OM235073 | Spain: Jaraicejo, Tagus Basin, Almonte Sub-basin, Almonte River | 23 |
| AT7309 | *Cobitis paludica* | This study | 119372 | 18/04/2009 | X | X | X | OM234973 | OM235074 | Spain: Jaraicejo, Tagus Basin, Almonte Sub-basin, Almonte River | 23 |
| AT7310 | *Cobitis paludica* | This study | 119373 | 18/04/2009 | X | X | X | OM234974 | OM235075 | Spain: Jaraicejo, Tagus Basin, Almonte Sub-basin, Almonte River | 23 |
| AT7311 | *Cobitis paludica* | This study | 119374 | 18/04/2009 | X | X | X | OM234975 | OM235076 | Spain: Jaraicejo, Tagus Basin, Almonte Sub-basin, Almonte River | 18 |
| AT7312 | *Cobitis paludica* | This study | 119375 | 18/04/2009 | X | X | X | OM234976 | OM235077 | Spain: Jaraicejo, Tagus Basin, Almonte Sub-basin, Almonte River | 18 |
| AT8964 | *Cobitis paludica* | This study | 119376 | 31/05/2009 | X | X | X | OM234997 | OM235082 | Spain: San Martín de Yeltes, Duero Basin, Huebra-Yeltes Sub-basin, Yeltes River | 18 |
| AT8965 | *Cobitis paludica* | This study | 119377 | 31/05/2009 |  | X | X | OM234998 | OM235083 | Spain: San Martín de Yeltes, Duero Basin, Huebra-Yeltes Sub-basin, Yeltes River | 18 |
| AT8966 | *Cobitis paludica* | This study | 119378 | 31/05/2009 | X | X | X | OM234999 | OM235084 | Spain: San Martín de Yeltes, Duero Basin, Huebra-Yeltes Sub-basin, Yeltes River | 18 |
| AT8967 | *Cobitis paludica* | This study | 119379 | 31/05/2009 |  | X | X | OM235000 | OM235085 | Spain: San Martín de Yeltes, Duero Basin, Huebra-Yeltes Sub-basin, Yeltes River | 18 |
| AT8968 | *Cobitis paludica* | This study | 119380 | 31/05/2009 | X | X | X | OM235001 | OM235086 | Spain: San Martín de Yeltes, Duero Basin, Huebra-Yeltes Sub-basin, Yeltes River | 18 |
| AT9091 | *Cobitis vettonica* | This study | 119556 | 01/06/2009 |  | X | X | OM234794 | OM235002 | Spain: La Bouza, Duero Basin, Águeda Sub-basin, Turones River | 18 |
| AT9296 | *Cobitis paludica* | This study | 119381 | 11/06/2009 | X | X |  | OM234970 |  | Spain: Trujillo, Tagus Basin, Almonte Sub-basin, Tamuja River | 18 |
| AT9297 | *Cobitis paludica* | This study | 119382 | 11/06/2009 | X | X |  | OM234971 |  | Spain: Trujillo, Tagus Basin, Almonte Sub-basin, Tamuja River | 18 |
| AT9346 | *Cobitis paludica* | This study | 119383 | 11/06/2009 | X | X | X | OM234958 | OM235068 | Spain: Santiago de Alcántara, Tagus Basin, Salor Sub-basin, Aurela River | 18 |
| AT9347 | *Cobitis paludica* | This study | 119384 | 11/06/2009 | X | X | X | OM234959 | OM235069 | Spain: Santiago de Alcántara, Tagus Basin, Salor Sub-basin, Aurela River | 18 |
| AT9348 | *Cobitis paludica* | This study | 119385 | 11/06/2009 | X | X | X | OM234960 | OM235070 | Spain: Santiago de Alcántara, Tagus Basin, Salor Sub-basin, Aurela River | 18 |
| AT9349 | *Cobitis paludica* | This study | 119386 | 11/06/2009 | X | X | X | OM234961 | OM235071 | Spain: Santiago de Alcántara, Tagus Basin, Salor Sub-basin, Aurela River | 18 |
| AT9350 | *Cobitis paludica* | This study | 119387 | 11/06/2009 | X | X | X | OM234962 | OM235072 | Spain: Santiago de Alcántara, Tagus Basin, Salor Sub-basin, Aurela River | 18 |
| AT9351 | *Cobitis paludica* | This study | 119558 | 11/06/2009 | X |  |  |  |  | Spain: Santiago de Alcántara, Tagus Basin, Salor Sub-basin, Aurela River | 18 |
| AT9352 | *Cobitis paludica* | This study | 119559 | 11/06/2009 | X |  |  |  |  | Spain: Santiago de Alcántara, Tagus Basin, Salor Sub-basin, Aurela River | 22 |
| AT9354 | *Cobitis paludica* | This study | 119560 | 11/06/2009 | X |  |  |  |  | Spain: Santiago de Alcántara, Tagus Basin, Salor Sub-basin, Aurela River | 22 |
| AT9355 | *Cobitis paludica* | This study | 119561 | 11/06/2009 | X |  |  |  |  | Spain: Santiago de Alcántara, Tagus Basin, Salor Sub-basin, Aurela River | 22 |
| AT9375 | *Cobitis paludica* | This study | 119388 | 11/06/2009 | X | X |  | OM234963 |  | Spain: Membrío, Tagus Basin, Salor Sub-basin, Salor River | 22 |
| CM6CC | *Cobitis vettonica* | This study | 119557 | 07/05/1995 |  | X |  | OM234846 |  | Spain: Cadalso de Gata, Tagus Basin, western Alagón Sub-basin, Árrago River | 22 |
| CP591 | *Cobitis paludica* | This study | 119389 | 07/05/1995 |  | X | X | OM234983 | OM235087 | Spain: La Iglesuela del Tiétar, Tagus Basin, Tiétar Sub-basin, Tiétar River | 22 |
| CP592 | *Cobitis paludica* | This study | 119390 | 07/05/1995 |  | X | X | OM234984 | OM235088 | Spain: La Iglesuela del Tiétar, Tagus Basin, Tiétar Sub-basin, Tiétar River | 22 |
| CP593 | *Cobitis paludica* | This study | 119391 | 07/05/1995 |  | X | X | OM234985 | OM235089 | Spain: La Iglesuela del Tiétar, Tagus Basin, Tiétar Sub-basin, Tiétar River | 22 |
| CP594 | *Cobitis paludica* | This study | 119392 | 07/05/1995 |  | X | X | OM234986 | OM235090 | Spain: La Iglesuela del Tiétar, Tagus Basin, Tiétar Sub-basin, Tiétar River | 22 |
| CP595 | *Cobitis paludica* | This study | 119393 | 07/05/1995 |  | X | X | OM234987 | OM235091 | Spain: La Iglesuela del Tiétar, Tagus Basin, Tiétar Sub-basin, Tiétar River | 21 |
| 2284PT | *Cobitis vettonica* | Perdices et al., 2018 |  |  |  | X |  | MH842916.1 |  | Portugal: Cegonhas Novas, Tagus Basin, Aravil Sub-basin, Aravil River | 21 |
| 158CP | *Cobitis vettonica* | Perdices et al., 2016 |  |  |  | X |  | KP161123.1 |  | Spain: Descargamaría, Duero Basin, Águeda Sub-basin, Malena River | 21 |
| 1ARR | *Cobitis vettonica* | Perdices & Doadrio, 2001 |  |  |  | X |  | AF263072.1 |  | Spain: Cadalso de Gata, Tagus Basin, western Alagón Sub-basin, Árrago River | 21 |
| 2ARR | *Cobitis vettonica* | Perdices & Doadrio, 2001 |  |  |  | X |  | AF263073.1 |  | Spain: Cadalso de Gata, Tagus Basin, western Alagón Sub-basin, Árrago River | 21 |
| 1945PT | *Cobitis vettonica* | Perdices & Coelho, 2020 |  |  |  | X |  | MN583187.1 |  | Portugal: Termas de Monfortinho, Tagus Basin, middle Erjas Sub-basin, Erjas River | 17 |
| 1946PT | *Cobitis vettonica* | Perdices & Coelho, 2020 |  |  |  | X |  | MN583188.1 |  | Portugal: Termas de Monfortinho, Tagus Basin, middle Erjas Sub-basin, Erjas River | 26 |
| 1982PT | *Cobitis paludica* | Perdices & Coelho, 2020 |  |  |  | X |  | MN583212.1 |  | Portugal: Azenha do Roque, Tagus Basin, lower Erjas Sub-basin, Erjas River | 26 |
| 1983PT | *Cobitis vettonica* | Perdices & Coelho, 2020 |  |  |  | X |  | MN583193.1 |  | Portugal: Azenha do Roque, Tagus Basin, lower Erjas Sub-basin, Erjas River | 26 |
| 2013PT | *Cobitis vettonica* | Perdices & Coelho, 2020 |  |  |  | X |  | MN583198.1 |  | Portugal: Serrinha, Tagus Basin, lower Erjas Sub-basin, Erjas River | 26 |
| 2037PT | *Cobitis paludica* | Perdices & Coelho, 2020 |  |  |  | X |  | MN583214.1 |  | Portugal: Cabeça Queimado, Tagus Basin, lower Erjas Sub-basin, Erjas River | 27 |
| 2039PT | *Cobitis vettonica* | Perdices & Coelho, 2020 |  |  |  | X |  | MN583200.1 |  | Portugal: Cabeça Queimado, Tagus Basin, lower Erjas Sub-basin, Erjas River | 27 |
| 2044PT | *Cobitis vettonica* | Perdices & Coelho, 2020 |  |  |  | X |  | MN583201.1 |  | Portugal: Salvaterra do Extremo, Tagus Basin, middle Erjas Sub-basin, Arades River | 26 |
| 2045PT | *Cobitis vettonica* | Perdices & Coelho, 2020 |  |  |  | X |  | MN583202.1 |  | Portugal: Salvaterra do Extremo, Tagus Basin, middle Erjas Sub-basin, Arades River | 26 |
| 2056PT | *Cobitis vettonica* | Perdices & Coelho, 2020 |  |  |  | X |  | MN583203.1 |  | Portugal: Salvaterra do Extremo, Tagus Basin, lower Erjas Sub-basin, Arades River | 14 |
| 2057PT | *Cobitis vettonica* | Perdices & Coelho, 2020 |  |  |  | X |  | MN583204.1 |  | Portugal: Salvaterra do Extremo, Tagus Basin, lower Erjas Sub-basin, Arades River | 14 |
| 2100PT | *Cobitis paludica* | Perdices & Coelho, 2020 |  |  |  | X |  | MN583216.1 |  | Portugal: Alcafozes, Tagus Basin, Aravil Sub-basin, Toula River | 14 |
| 2108PT | *Cobitis vettonica* | Perdices & Coelho, 2020 |  |  |  | X |  | MN583206.1 |  | Portugal: Alcafozes, Tagus Basin, Aravil Sub-basin, Aravil River | 14 |
| 2167PT | *Cobitis paludica* | Perdices & Coelho, 2020 |  |  |  | X |  | MN583218.1 |  | Portugal: Cegonhas Novas, Tagus Basin, Aravil Sub-basin, Aravil River | 14 |
| 2274PT | *Cobitis vettonica* | Perdices & Coelho, 2020 |  |  |  | X |  | MN583189.1 |  | Portugal: Termas de Monfortinho, Tagus Basin, middle Erjas Sub-basin, Erjas River | 13 |
| 2275PT | *Cobitis vettonica* | Perdices & Coelho, 2020 |  |  |  | X |  | MN583190.1 |  | Portugal: Termas de Monfortinho, Tagus Basin, middle Erjas Sub-basin, Erjas River | 13 |
| 2276PT | *Cobitis vettonica* | Perdices & Coelho, 2020 |  |  |  | X |  | MN583191.1 |  | Portugal: Termas de Monfortinho, Tagus Basin, middle Erjas Sub-basin, Erjas River | 13 |
| 2277PT | *Cobitis vettonica* | Perdices & Coelho, 2020 |  |  |  | X |  | MN583192.1 |  | Portugal: Termas de Monfortinho, Tagus Basin, middle Erjas Sub-basin, Erjas River | 13 |
| 2278PT | *Cobitis vettonica* | Perdices & Coelho, 2020 |  |  |  | X |  | MN583199.1 |  | Portugal: Serrinha, Tagus Basin, lower Erjas Sub-basin, Erjas River | 13 |
| 2279PT | *Cobitis paludica* | Perdices & Coelho, 2020 |  |  |  | X |  | MN583213.1 |  | Portugal: Serrinha, Tagus Basin, lower Erjas Sub-basin, Erjas River | 13 |
| 2285PT | *Cobitis vettonica* | Perdices & Coelho, 2020 |  |  |  | X |  | MN583208.1 |  | Portugal: Cegonhas Novas, Tagus Basin, Aravil Sub-basin, Aravil River | 26 |
| 2286PT | *Cobitis vettonica* | Perdices & Coelho, 2020 |  |  |  | X |  | MN583209.1 |  | Portugal: Cegonhas Novas, Tagus Basin, Aravil Sub-basin, Aravil River | 26 |
| 2287PT | *Cobitis vettonica* | Perdices & Coelho, 2020 |  |  |  | X |  | MN583210.1 |  | Portugal: Cegonhas Novas, Tagus Basin, Aravil Sub-basin, Aravil River | 26 |
| 2291PT | *Cobitis vettonica* | Perdices & Coelho, 2020 |  |  |  | X |  | MN583183.1 |  | Portugal: Alfrividas, Tagus Basin, Ponsul Sub-basin, Alfrividas River | 26 |
| 2292PT | *Cobitis vettonica* | Perdices & Coelho, 2020 |  |  |  | X |  | MN583184.1 |  | Portugal: Alfrividas, Tagus Basin, Ponsul Sub-basin, Alfrividas River | 26 |
| 2293PT | *Cobitis vettonica* | Perdices & Coelho, 2020 |  |  |  | X |  | MN583185.1 |  | Portugal: Alfrividas, Tagus Basin, Ponsul Sub-basin, Alfrividas River | 29 |
| 2294PT | *Cobitis paludica* | Perdices & Coelho, 2020 |  |  |  | X |  | MN583219.1 |  | Portugal: Alfrividas, Tagus Basin, Ponsul Sub-basin, Alfrividas River | 29 |
| 2295PT | *Cobitis vettonica* | Perdices & Coelho, 2020 |  |  |  | X |  | MN583186.1 |  | Portugal: Alfrividas, Tagus Basin, Ponsul Sub-basin, Alfrividas River | 29 |
| 2304PT | *Cobitis vettonica* | Perdices & Coelho, 2020 |  |  |  | X |  | MN583194.1 |  | Portugal: Azenha do Roque, Tagus Basin, lower Erjas Sub-basin, Erjas River | 29 |
| 2305PT | *Cobitis vettonica* | Perdices & Coelho, 2020 |  |  |  | X |  | MN583195.1 |  | Portugal: Azenha do Roque, Tagus Basin, lower Erjas Sub-basin, Erjas River | 27 |
| 2306PT | *Cobitis vettonica* | Perdices & Coelho, 2020 |  |  |  | X |  | MN583196.1 |  | Portugal: Azenha do Roque, Tagus Basin, lower Erjas Sub-basin, Erjas River | 27 |
| 3187PT | *Cobitis paludica* | Perdices & Coelho, 2020 |  |  |  | X |  | MN583220.1 |  | Portugal: Monte Gordo, Tagus Basin, Ocreza Sub-basin, Alvito River | 37 |
| 168CP | *Cobitis vettonica* | Doadrio & Perdices, 2005 |  |  |  | X |  | AY860181.1 |  | Spain: La Herguijuela, Duero Basin, Águeda Sub-basin, Águeda River | 37 |
| 268CP | *Cobitis vettonica* | Doadrio & Perdices, 2005 |  |  |  | X |  | AY860183.1 |  | Spain: Valverde del Fresno, Tagus Basin, upper Erjas Sub-basin, Erjas River | 37 |
| 575A | *Cobitis vettonica* | Doadrio & Perdices, 2005 |  |  |  | X |  | AY860182.1 |  | Spain: La Rebollosa, Tagus Basin, eastern Alagón Sub-basin, Ladrillar River | 37 |
| 5ARR | *Cobitis vettonica* | Doadrio & Perdices, 2005 |  |  |  | X |  | KP161122.1 |  | Spain: Cadalso de Gata, Tagus Basin, western Alagón Sub-basin, Árrago River | 37 |

**Table S2.** Stacks results for each analysis group.

| Ustacks parameters | Mean loci | Mean coverage | Cstacks parameters | Catalog loci | Populations loci | Variable SNPs | Fixed SNPs |
| --- | --- | --- | --- | --- | --- | --- | --- |
| *m*=5, *M*=3 | 15668 | 28.31 | n=2 | 108550 | 6684 | 4696 | 49 |
|  |  |  | n=3 | 103401 | 6691 | 4683 | 37 |
|  |  |  | n=4 | 100072 | 6695 | 4677 | 51 |

**Table S3.** Percentage of missing data by locus and by individual for SNPs data.

| ***Cobitis vettonica*** | | | | | | | | ***Cobitis vettonica + Cobitis paludica*** | | | | | | | | | |
| --- | --- | --- | --- | --- | --- | --- | --- | --- | --- | --- | --- | --- | --- | --- | --- | --- | --- |
| **Locus** | **% of  missing data** | **Locus** | **% of  missing data** | **Locus** | **% of  missing data** | **Individual** | **% of  missing data** | **Locus** | **% of  missing data** | **Locus** | **% of  missing data** | **Locus** | **% of  missing data** | **Locus** | **% of  missing data** | **Individual** | **% of  missing data** |
| 2_105 | 0.00 | 4791_89 | 8.33 | 9747_68 | 1.67 | AT27666 | 1.90 | 2_105 | 0.00 | 3564_112 | 0.94 | 7407_15 | 6.60 | 10820_43 | 2.83 | AT7308 | 3.15 |
| 6_12 | 0.00 | 4794_9 | 0.00 | 9749_150 | 6.67 | AT27667 | 0.92 | 4_103 | 3.77 | 3566_7 | 2.83 | 7408_28 | 0.00 | 10823_14 | 1.89 | AT7309 | 4.30 |
| 7_164 | 0.00 | 4795_138 | 3.33 | 9753_36 | 6.67 | AT27668 | 0.65 | 6_12 | 0.00 | 3567_19 | 0.94 | 7412_111 | 1.89 | 10824_44 | 15.09 | AT7310 | 2.95 |
| 8_97 | 0.00 | 4796_17 | 16.67 | 9754_53 | 3.33 | AT27669 | 1.08 | 7_164 | 1.89 | 3569_70 | 1.89 | 7413_65 | 0.94 | 10826_49 | 6.60 | AT7311 | 6.48 |
| 9_43 | 5.00 | 4815_12 | 6.67 | 9755_5 | 0.00 | AT6834 | 1.20 | 8_97 | 3.77 | 3574_123 | 0.00 | 7414_102 | 12.26 | 10827_78 | 3.77 | AT7312 | 2.84 |
| 10_133 | 1.67 | 4819_145 | 1.67 | 9756_23 | 0.00 | AT6835 | 5.68 | 9_43 | 10.38 | 3577_52 | 5.66 | 7422_65 | 2.83 | 10830_74 | 3.77 | AT9346 | 1.04 |
| 11_109 | 3.33 | 4820_157 | 0.00 | 9764_137 | 3.33 | AT6836 | 3.40 | 10_133 | 0.94 | 3578_93 | 2.83 | 7428_16 | 0.94 | 10832_128 | 5.66 | AT9347 | 1.10 |
| 15_48 | 0.00 | 4821_25 | 0.00 | 9766_9 | 5.00 | AT6837 | 7.85 | 11_109 | 8.49 | 3580_78 | 5.66 | 7434_184 | 0.00 | 10834_60 | 3.77 | AT9348 | 1.17 |
| 20_46 | 8.33 | 4825_152 | 1.67 | 9767_43 | 5.00 | AT6838 | 1.90 | 13_6 | 3.77 | 3582_71 | 2.83 | 7437_79 | 1.89 | 10838_91 | 9.43 | AT9349 | 1.39 |
| 21_111 | 1.67 | 4826_11 | 3.33 | 9770_145 | 5.00 | AT6839 | 1.20 | 15_48 | 0.00 | 3587_132 | 0.94 | 7439_54 | 4.72 | 10844_84 | 1.89 | AT9350 | 2.93 |
| 31_157 | 1.67 | 4829_97 | 6.67 | 9772_175 | 0.00 | AT6841 | 10.68 | 20_46 | 8.49 | 3589_105 | 8.49 | 7441_32 | 0.94 | 10845_105 | 0.94 | AT9351 | 4.28 |
| 32_24 | 5.00 | 4835_151 | 1.67 | 9774_28 | 1.67 | AT6843 | 1.43 | 21_111 | 0.94 | 3595_103 | 7.55 | 7446_50 | 1.89 | 10848_74 | 8.49 | AT9352 | 8.40 |
| 33_54 | 0.00 | 4836_39 | 3.33 | 9780_29 | 0.00 | AT27317 | 4.58 | 31_157 | 3.77 | 3598_158 | 0.94 | 7451_14 | 0.00 | 10849_65 | 13.21 | AT9354 | 6.48 |
| 39_17 | 0.00 | 4840_57 | 1.67 | 9784_102 | 1.67 | AT27318 | 1.05 | 32_24 | 5.66 | 3600_111 | 11.32 | 7452_130 | 6.60 | 10850_44 | 0.94 | AT9355 | 3.68 |
| 44_105 | 0.00 | 4853_39 | 0.00 | 9785_100 | 21.67 | AT27309 | 0.67 | 33_54 | 4.72 | 3602_146 | 5.66 | 7457_7 | 7.55 | 10851_59 | 0.94 | AT27631 | 1.92 |
| 53_103 | 0.00 | 4861_85 | 0.00 | 9786_106 | 0.00 | AT27310 | 1.18 | 39_17 | 0.00 | 3605_97 | 9.43 | 7461_119 | 12.26 | 10852_170 | 9.43 | AT27632 | 1.50 |
| 57_47 | 3.33 | 4872_152 | 13.33 | 9795_8 | 10.00 | AT27311 | 0.85 | 44_105 | 0.00 | 3611_83 | 3.77 | 7462_103 | 4.72 | 10853_9 | 0.00 | AT27633 | 1.04 |
| 60_149 | 0.00 | 4875_51 | 0.00 | 9798_49 | 3.33 | AT27312 | 3.30 | 53_103 | 0.00 | 3613_133 | 0.00 | 7465_45 | 5.66 | 10854_7 | 5.66 | AT27634 | 1.41 |
| 62_109 | 1.67 | 4877_121 | 1.67 | 9802_29 | 1.67 | AT27313 | 22.00 | 57_47 | 3.77 | 3614_37 | 0.00 | 7468_161 | 0.00 | 10857_19 | 0.94 | AT27635 | 1.67 |
| 64_20 | 3.33 | 4878_87 | 1.67 | 9807_157 | 3.33 | AT27314 | 0.72 | 60_149 | 0.00 | 3619_51 | 1.89 | 7473_36 | 0.94 | 10858_5 | 0.00 | AT27636 | 2.18 |
| 66_84 | 3.33 | 4879_137 | 0.00 | 9812_72 | 0.00 | AT27315 | 28.53 | 62_109 | 0.94 | 3622_145 | 2.83 | 7474_165 | 7.55 | 10859_33 | 11.32 | AT27637 | 1.21 |
| 69_67 | 6.67 | 4880_24 | 8.33 | 9814_57 | 0.00 | AT27316 | 3.60 | 64_20 | 2.83 | 3626_156 | 6.60 | 7476_160 | 7.55 | 10860_156 | 16.98 | AT27639 | 6.13 |
| 79_123 | 10.00 | 4881_30 | 0.00 | 9815_148 | 6.67 | AT6689 | 2.43 | 66_84 | 7.55 | 3627_90 | 0.00 | 7478_72 | 16.04 | 10861_95 | 4.72 | AT27640 | 1.17 |
| 86_65 | 0.00 | 4890_38 | 0.00 | 9816_10 | 0.00 | AT6690 | 2.15 | 69_67 | 5.66 | 3632_62 | 13.21 | 7479_128 | 5.66 | 10865_26 | 0.00 | AT15755 | 10.67 |
| 87_6 | 1.67 | 4895_82 | 1.67 | 9829_155 | 0.00 | AT6691 | 2.47 | 79_123 | 12.26 | 3634_82 | 1.89 | 7487_41 | 0.94 | 10870_11 | 2.83 | AT15756 | 7.82 |
| 101_75 | 8.33 | 4897_65 | 3.33 | 9834_139 | 3.33 | AT6692 | 2.70 | 81_184 | 2.83 | 3636_69 | 13.21 | 7496_43 | 0.94 | 10875_134 | 6.60 | AT15757 | 2.20 |
| 102_14 | 8.33 | 4898_74 | 6.67 | 9836_8 | 8.33 | AT6699 | 2.63 | 86_65 | 0.00 | 3637_138 | 1.89 | 7500_159 | 6.60 | 10879_129 | 2.83 | AT9375 | 2.86 |
| 107_113 | 8.33 | 4902_113 | 1.67 | 9837_96 | 0.00 | AT27553 | 9.43 | 87_6 | 2.83 | 3641_10 | 11.32 | 7505_23 | 2.83 | 10880_156 | 13.21 | AT16031 | 3.48 |
| 108_26 | 8.33 | 4903_92 | 10.00 | 9840_82 | 5.00 | AT27555 | 1.45 | 101_75 | 5.66 | 3648_14 | 2.83 | 7506_139 | 0.00 | 10896_73 | 0.94 | AT9296 | 3.00 |
| 109_9 | 0.00 | 4904_61 | 3.33 | 9847_44 | 0.00 | AT27556 | 2.47 | 102_14 | 7.55 | 3650_130 | 4.72 | 7507_28 | 2.83 | 10898_123 | 1.89 | AT9297 | 7.65 |
| 111_114 | 6.67 | 4909_42 | 10.00 | 9848_42 | 0.00 | AT27557 | 1.38 | 107_113 | 11.32 | 3655_42 | 0.00 | 7508_25 | 0.94 | 10899_43 | 0.00 | AT15985 | 14.43 |
| 113_61 | 0.00 | 4910_85 | 0.00 | 9849_47 | 8.33 | AT27558 | 14.08 | 108_26 | 6.60 | 3656_43 | 3.77 | 7512_131 | 0.94 | 10900_27 | 1.89 | AT15987 | 9.10 |
| 121_170 | 8.33 | 4914_30 | 1.67 | 9855_36 | 0.00 | AT27560 | 5.50 | 109_9 | 0.94 | 3657_115 | 9.43 | 7514_49 | 4.72 | 10901_90 | 6.60 | AT20372 | 17.72 |
| 124_111 | 5.00 | 4915_33 | 3.33 | 9857_82 | 0.00 | AT27561 | 3.08 | 111_114 | 12.26 | 3659_167 | 3.77 | 7515_23 | 5.66 | 10909_43 | 0.94 | AT20373 | 5.53 |
| 125_31 | 0.00 | 4917_75 | 1.67 | 9859_67 | 6.67 | AT27562 | 1.90 | 113_61 | 0.00 | 3660_36 | 1.89 | 7519_58 | 0.00 | 10915_7 | 1.89 | AT20374 | 7.98 |
| 130_149 | 3.33 | 4922_129 | 1.67 | 9861_183 | 0.00 | AT20195 | 3.13 | 121_170 | 8.49 | 3661_86 | 4.72 | 7524_87 | 0.94 | 10916_58 | 0.94 | AT20375 | 7.14 |
| 131_110 | 11.67 | 4925_60 | 0.00 | 9864_73 | 11.67 | AT20196 | 0.95 | 124_111 | 4.72 | 3662_124 | 4.72 | 7526_134 | 1.89 | 10921_195 | 1.89 | AT20376 | 11.15 |
| 133_80 | 3.33 | 4932_175 | 13.33 | 9865_61 | 0.00 | AT20197 | 1.75 | 125_31 | 1.89 | 3669_47 | 5.66 | 7527_113 | 3.77 | 10929_84 | 0.00 | AT20377 | 3.75 |
| 134_90 | 3.33 | 4936_45 | 8.33 | 9868_153 | 0.00 | AT20198 | 1.15 | 130_149 | 2.83 | 3670_156 | 0.94 | 7528_145 | 0.00 | 10933_91 | 4.72 | AT8964 | 3.02 |
| 140_121 | 0.00 | 4938_140 | 3.33 | 9869_59 | 0.00 | AT20199 | 0.88 | 131_110 | 10.38 | 3671_71 | 0.00 | 7530_158 | 8.49 | 10934_84 | 1.89 | AT8966 | 16.66 |
| 142_89 | 0.00 | 4941_122 | 1.67 | 9873_60 | 0.00 | AT20201 | 15.13 | 133_80 | 3.77 | 3674_30 | 8.49 | 7532_107 | 0.00 | 10936_158 | 2.83 | AT8968 | 3.24 |
| 146_151 | 10.00 | 4948_116 | 3.33 | 9875_73 | 18.33 | AT20202 | 0.92 | 134_90 | 2.83 | 3678_56 | 6.60 | 7533_49 | 2.83 | 10937_51 | 4.72 | AT27375 | 7.12 |
| 147_5 | 0.00 | 4949_71 | 0.00 | 9886_113 | 0.00 | AT20203 | 1.50 | 140_121 | 0.94 | 3680_34 | 5.66 | 7543_186 | 9.43 | 10946_93 | 5.66 | AT27376 | 4.39 |
| 149_54 | 6.67 | 4951_83 | 0.00 | 9888_106 | 5.00 | AT20204 | 0.85 | 142_89 | 0.94 | 3681_95 | 1.89 | 7545_137 | 3.77 | 10959_125 | 2.83 | AT27377 | 1.19 |
| 154_57 | 0.00 | 4952_82 | 8.33 | 9889_149 | 0.00 | AT20206 | 3.68 | 146_151 | 8.49 | 3682_107 | 7.55 | 7546_48 | 5.66 | 10960_68 | 13.21 | AT27378 | 1.26 |
| 156_18 | 1.67 | 4956_39 | 3.33 | 9891_122 | 18.33 | AT27365 | 0.80 | 147_5 | 1.89 | 3686_102 | 0.00 | 7553_139 | 0.94 | 10962_109 | 0.94 | AT27379 | 2.67 |
| 161_97 | 0.00 | 4957_25 | 0.00 | 9894_119 | 0.00 | AT27366 | 0.75 | 149_54 | 9.43 | 3687_91 | 12.26 | 7554_91 | 1.89 | 10963_117 | 1.89 | AT27666 | 1.87 |
| 166_102 | 1.67 | 4961_139 | 1.67 | 9896_46 | 0.00 | AT27367 | 2.60 | 154_57 | 0.94 | 3688_8 | 14.15 | 7557_62 | 11.32 | 10964_42 | 2.83 | AT27667 | 0.84 |
| 168_11 | 3.33 | 4964_14 | 18.33 | 9898_45 | 1.67 | AT27368 | 1.65 | 156_18 | 0.94 | 3692_91 | 14.15 | 7560_59 | 0.00 | 10971_73 | 0.00 | AT27668 | 0.59 |
| 184_8 | 0.00 | 4965_148 | 15.00 | 9904_71 | 1.67 | AT27369 | 0.72 | 161_97 | 0.94 | 3693_166 | 0.94 | 7565_121 | 0.94 | 10972_96 | 1.89 | AT27669 | 1.06 |
| 186_23 | 0.00 | 4976_99 | 1.67 | 9906_43 | 0.00 | AT27370 | 2.53 | 166_102 | 1.89 | 3695_66 | 0.94 | 7572_174 | 11.32 | 10989_59 | 0.94 | AT6834 | 1.17 |
| 187_105 | 6.67 | 4978_8 | 0.00 | 9911_95 | 1.67 | AT27371 | 1.63 | 168_11 | 6.60 | 3696_70 | 0.94 | 7573_13 | 9.43 | 10992_98 | 0.94 | AT6835 | 5.44 |
| 194_95 | 1.67 | 4980_185 | 0.00 | 9915_122 | 1.67 | AT27372 | 2.80 | 175_70 | 1.89 | 3699_40 | 0.94 | 7574_64 | 0.94 | 10994_43 | 7.55 | AT6836 | 3.33 |
| 200_8 | 0.00 | 4984_55 | 6.67 | 9916_148 | 10.00 | AT27373 | 1.55 | 183_126 | 9.43 | 3700_84 | 1.89 | 7577_116 | 2.83 | 10997_43 | 0.00 | AT6837 | 7.82 |
| 201_75 | 3.33 | 4985_129 | 5.00 | 9924_105 | 0.00 | AT27374 | 1.13 | 184_8 | 3.77 | 3701_80 | 0.94 | 7581_144 | 5.66 | 10998_169 | 2.83 | AT6838 | 1.76 |
| 206_58 | 0.00 | 4986_70 | 0.00 | 9925_24 | 1.67 | AT15935 | 9.30 | 186_23 | 0.00 | 3702_179 | 4.72 | 7582_119 | 4.72 | 11001_100 | 1.89 | AT6839 | 1.12 |
| 210_87 | 0.00 | 4995_83 | 1.67 | 9937_137 | 6.67 | AT15936 | 1.05 | 187_105 | 8.49 | 3704_86 | 10.38 | 7583_127 | 1.89 | 11002_55 | 2.83 | AT6841 | 10.64 |
| 213_109 | 1.67 | 5000_16 | 0.00 | 9938_98 | 21.67 | AT15937 | 2.13 | 190_126 | 0.00 | 3706_78 | 1.89 | 7584_148 | 0.94 | 11004_91 | 0.00 | AT6843 | 1.32 |
| 215_29 | 0.00 | 5002_157 | 1.67 | 9944_71 | 5.00 | AT15938 | 3.00 | 194_95 | 0.94 | 3712_45 | 1.89 | 7589_105 | 3.77 | 11007_157 | 13.21 | AT27317 | 4.54 |
| 216_110 | 16.67 | 5005_51 | 1.67 | 9947_101 | 0.00 | AT15939 | 0.78 | 200_8 | 0.94 | 3713_7 | 8.49 | 7594_176 | 8.49 | 11013_31 | 0.94 | AT27318 | 0.97 |
| 217_25 | 0.00 | 5006_26 | 0.00 | 9952_5 | 3.33 |  |  | 201_75 | 4.72 | 3717_95 | 4.72 | 7596_50 | 0.00 | 11015_165 | 3.77 | AT27309 | 0.64 |
| 218_96 | 0.00 | 5007_73 | 3.33 | 9954_44 | 0.00 |  |  | 206_58 | 0.00 | 3721_111 | 0.00 | 7597_139 | 0.00 | 11018_5 | 4.72 | AT27310 | 1.08 |
| 220_17 | 5.00 | 5009_94 | 1.67 | 9962_158 | 0.00 |  |  | 210_87 | 0.00 | 3722_9 | 1.89 | 7599_7 | 0.94 | 11025_55 | 2.83 | AT27311 | 0.77 |
| 221_158 | 6.67 | 5012_112 | 15.00 | 9969_35 | 15.00 |  |  | 213_109 | 0.94 | 3727_163 | 0.94 | 7600_6 | 10.38 | 11028_114 | 8.49 | AT27312 | 3.11 |
| 224_60 | 0.00 | 5014_19 | 0.00 | 9973_88 | 8.33 |  |  | 215_29 | 0.00 | 3728_71 | 0.00 | 7603_81 | 0.94 | 11031_24 | 3.77 | AT27313 | 22.32 |
| 237_94 | 1.67 | 5015_65 | 1.67 | 9975_117 | 0.00 |  |  | 216_110 | 15.09 | 3735_15 | 6.60 | 7605_234 | 5.66 | 11038_138 | 2.83 | AT27314 | 0.68 |
| 240_79 | 3.33 | 5024_113 | 8.33 | 9976_20 | 1.67 |  |  | 217_25 | 0.00 | 3736_38 | 2.83 | 7607_41 | 0.00 | 11046_36 | 1.89 | AT27315 | 27.88 |
| 241_90 | 0.00 | 5026_133 | 3.33 | 9977_134 | 0.00 |  |  | 218_96 | 3.77 | 3737_34 | 2.83 | 7608_36 | 11.32 | 11053_53 | 0.00 | AT27316 | 3.31 |
| 246_53 | 6.67 | 5031_41 | 13.33 | 9980_80 | 0.00 |  |  | 220_17 | 7.55 | 3745_9 | 2.83 | 7609_159 | 1.89 | 11057_160 | 0.00 | AT6689 | 2.64 |
| 248_29 | 10.00 | 5032_21 | 0.00 | 9988_117 | 16.67 |  |  | 221_158 | 7.55 | 3749_45 | 2.83 | 7611_106 | 0.00 | 11061_57 | 9.43 | AT6690 | 2.38 |
| 249_79 | 5.00 | 5045_67 | 0.00 | 9990_85 | 0.00 |  |  | 224_60 | 0.00 | 3754_53 | 1.89 | 7615_190 | 6.60 | 11064_29 | 0.00 | AT6691 | 2.67 |
| 253_5 | 5.00 | 5047_132 | 3.33 | 9991_96 | 13.33 |  |  | 227_102 | 5.66 | 3757_150 | 0.94 | 7618_90 | 10.38 | 11072_95 | 0.94 | AT6692 | 2.89 |
| 256_29 | 8.33 | 5049_32 | 5.00 | 9993_109 | 13.33 |  |  | 237_94 | 1.89 | 3766_114 | 0.94 | 7622_158 | 0.00 | 11077_27 | 13.21 | AT6699 | 2.86 |
| 257_151 | 5.00 | 5056_66 | 0.00 | 9994_155 | 1.67 |  |  | 240_79 | 1.89 | 3769_153 | 1.89 | 7624_52 | 5.66 | 11078_21 | 3.77 | AT27553 | 9.26 |
| 258_16 | 0.00 | 5058_38 | 0.00 | 9997_40 | 1.67 |  |  | 241_90 | 1.89 | 3770_98 | 3.77 | 7625_51 | 0.00 | 11085_104 | 1.89 | AT27555 | 1.32 |
| 260_72 | 0.00 | 5060_24 | 0.00 | 10002_65 | 3.33 |  |  | 246_53 | 7.55 | 3775_50 | 5.66 | 7631_28 | 0.94 | 11086_18 | 0.94 | AT27556 | 2.34 |
| 261_120 | 0.00 | 5062_114 | 0.00 | 10006_165 | 1.67 |  |  | 248_29 | 11.32 | 3778_126 | 2.83 | 7633_88 | 4.72 | 11089_96 | 3.77 | AT27557 | 1.28 |
| 262_163 | 10.00 | 5063_14 | 1.67 | 10008_105 | 5.00 |  |  | 249_79 | 4.72 | 3779_131 | 0.94 | 7635_108 | 11.32 | 11090_69 | 0.00 | AT27558 | 13.99 |
| 264_116 | 3.33 | 5064_51 | 1.67 | 10014_159 | 0.00 |  |  | 253_5 | 5.66 | 3782_106 | 6.60 | 7636_68 | 7.55 | 11100_164 | 0.94 | AT27560 | 5.33 |
| 268_14 | 1.67 | 5069_58 | 13.33 | 10015_125 | 8.33 |  |  | 254_82 | 0.00 | 3784_122 | 4.72 | 7638_86 | 11.32 | 11104_13 | 6.60 | AT27561 | 2.93 |
| 275_110 | 1.67 | 5072_61 | 1.67 | 10016_50 | 3.33 |  |  | 256_29 | 10.38 | 3785_98 | 0.00 | 7642_24 | 2.83 | 11109_55 | 3.77 | AT27562 | 1.85 |
| 276_166 | 8.33 | 5073_5 | 0.00 | 10021_74 | 10.00 |  |  | 257_151 | 11.32 | 3786_153 | 4.72 | 7646_143 | 7.55 | 11113_187 | 4.72 | AT20195 | 3.00 |
| 278_35 | 0.00 | 5077_57 | 0.00 | 10022_63 | 5.00 |  |  | 258_16 | 0.00 | 3790_92 | 3.77 | 7649_143 | 7.55 | 11118_159 | 2.83 | AT20196 | 0.88 |
| 283_40 | 0.00 | 5078_183 | 10.00 | 10023_46 | 1.67 |  |  | 260_72 | 0.00 | 3792_84 | 4.72 | 7650_155 | 3.77 | 11121_45 | 0.00 | AT20197 | 1.61 |
| 296_85 | 5.00 | 5079_117 | 8.33 | 10025_172 | 1.67 |  |  | 261_120 | 3.77 | 3793_84 | 0.00 | 7651_28 | 8.49 | 11122_29 | 0.94 | AT20198 | 1.06 |
| 300_47 | 11.67 | 5082_63 | 0.00 | 10029_132 | 0.00 |  |  | 262_163 | 15.09 | 3795_98 | 1.89 | 7653_81 | 3.77 | 11123_27 | 11.32 | AT20199 | 0.82 |
| 311_104 | 0.00 | 5085_19 | 3.33 | 10030_36 | 0.00 |  |  | 264_116 | 2.83 | 3799_75 | 1.89 | 7654_180 | 5.66 | 11125_58 | 0.00 | AT20201 | 14.92 |
| 312_120 | 1.67 | 5090_95 | 0.00 | 10036_70 | 0.00 |  |  | 268_14 | 0.94 | 3803_44 | 13.21 | 7660_123 | 10.38 | 11126_27 | 0.94 | AT20202 | 0.84 |
| 316_74 | 1.67 | 5096_194 | 5.00 | 10037_35 | 0.00 |  |  | 272_62 | 7.55 | 3807_135 | 1.89 | 7663_17 | 6.60 | 11137_152 | 0.00 | AT20203 | 1.45 |
| 323_20 | 5.00 | 5098_178 | 1.67 | 10051_34 | 10.00 |  |  | 275_110 | 5.66 | 3808_71 | 4.72 | 7664_130 | 5.66 | 11140_13 | 0.00 | AT20204 | 0.82 |
| 327_167 | 11.67 | 5099_48 | 1.67 | 10056_153 | 3.33 |  |  | 276_166 | 6.60 | 3809_60 | 10.38 | 7666_156 | 8.49 | 11141_13 | 6.60 | AT20206 | 3.46 |
| 328_143 | 1.67 | 5108_79 | 15.00 | 10058_121 | 1.67 |  |  | 278_35 | 0.00 | 3820_28 | 3.77 | 7668_87 | 2.83 | 11142_103 | 1.89 | AT27365 | 0.88 |
| 329_44 | 6.67 | 5115_37 | 3.33 | 10059_151 | 0.00 |  |  | 283_40 | 0.94 | 3826_95 | 0.00 | 7669_54 | 9.43 | 11149_124 | 8.49 | AT27366 | 0.77 |
| 332_100 | 6.67 | 5116_17 | 3.33 | 10060_31 | 0.00 |  |  | 292_42 | 2.83 | 3830_26 | 5.66 | 7672_139 | 10.38 | 11150_67 | 2.83 | AT27367 | 2.60 |
| 336_25 | 0.00 | 5117_145 | 0.00 | 10063_9 | 8.33 |  |  | 296_85 | 2.83 | 3837_61 | 3.77 | 7674_69 | 1.89 | 11154_92 | 0.94 | AT27368 | 1.72 |
| 337_106 | 0.00 | 5130_36 | 3.33 | 10064_82 | 1.67 |  |  | 300_47 | 9.43 | 3838_106 | 11.32 | 7682_89 | 1.89 | 11156_106 | 0.00 | AT27369 | 0.75 |
| 343_59 | 1.67 | 5135_47 | 0.00 | 10067_37 | 11.67 |  |  | 311_104 | 0.00 | 3843_110 | 7.55 | 7685_23 | 9.43 | 11159_91 | 11.32 | AT27370 | 2.67 |
| 344_120 | 5.00 | 5138_135 | 3.33 | 10072_152 | 11.67 |  |  | 312_120 | 2.83 | 3845_152 | 12.26 | 7686_60 | 0.00 | 11162_94 | 14.15 | AT27371 | 1.59 |
| 346_26 | 1.67 | 5139_51 | 1.67 | 10081_9 | 1.67 |  |  | 316_74 | 2.83 | 3854_7 | 0.94 | 7692_40 | 0.94 | 11168_95 | 0.00 | AT27372 | 2.91 |
| 355_111 | 5.00 | 5141_194 | 8.33 | 10085_51 | 16.67 |  |  | 322_14 | 0.94 | 3860_120 | 0.00 | 7693_152 | 3.77 | 11172_135 | 10.38 | AT27373 | 1.52 |
| 359_90 | 6.67 | 5146_98 | 0.00 | 10087_77 | 3.33 |  |  | 323_20 | 13.21 | 3861_23 | 3.77 | 7701_39 | 9.43 | 11176_50 | 0.00 | AT27374 | 1.15 |
| 366_174 | 5.00 | 5149_70 | 10.00 | 10089_158 | 0.00 |  |  | 326_56 | 0.94 | 3865_122 | 0.94 | 7705_83 | 5.66 | 11181_113 | 0.00 | AT15935 | 9.21 |
| 369_149 | 3.33 | 5152_45 | 10.00 | 10094_73 | 3.33 |  |  | 327_167 | 11.32 | 3867_138 | 15.09 | 7707_125 | 0.00 | 11185_72 | 14.15 | AT15936 | 1.17 |
| 371_12 | 0.00 | 5157_7 | 1.67 | 10095_145 | 5.00 |  |  | 328_143 | 3.77 | 3871_10 | 12.26 | 7709_148 | 0.00 | 11189_78 | 0.94 | AT15937 | 2.01 |
| 379_49 | 8.33 | 5161_91 | 5.00 | 10107_174 | 5.00 |  |  | 329_44 | 5.66 | 3877_17 | 0.94 | 7710_40 | 0.94 | 11190_37 | 1.89 | AT15938 | 3.02 |
| 382_33 | 5.00 | 5182_42 | 1.67 | 10109_97 | 0.00 |  |  | 332_100 | 6.60 | 3882_162 | 8.49 | 7712_106 | 0.00 | 11195_53 | 0.94 | AT15939 | 0.75 |
| 385_58 | 0.00 | 5185_150 | 8.33 | 10112_17 | 0.00 |  |  | 336_25 | 0.94 | 3883_168 | 6.60 | 7722_106 | 8.49 | 11197_144 | 10.38 | 107 | 7.82 |
| 392_111 | 10.00 | 5189_127 | 0.00 | 10117_49 | 3.33 |  |  | 337_106 | 0.00 | 3892_136 | 0.94 | 7724_83 | 2.83 | 11204_62 | 0.00 | 108 | 7.82 |
| 396_47 | 0.00 | 5190_40 | 1.67 | 10119_45 | 3.33 |  |  | 343_59 | 2.83 | 3894_24 | 11.32 | 7726_85 | 7.55 | 11206_45 | 0.94 | 109 | 1.76 |
| 397_67 | 0.00 | 5191_44 | 8.33 | 10120_65 | 0.00 |  |  | 344_120 | 2.83 | 3897_30 | 5.66 | 7729_96 | 0.94 | 11207_75 | 14.15 | 110 | 1.76 |
| 399_83 | 11.67 | 5203_9 | 1.67 | 10128_50 | 0.00 |  |  | 346_26 | 4.72 | 3902_28 | 5.66 | 7730_66 | 6.60 | 11208_85 | 6.60 | 111 | 1.12 |
| 401_90 | 0.00 | 5224_41 | 1.67 | 10131_123 | 1.67 |  |  | 355_111 | 8.49 | 3904_48 | 0.00 | 7733_73 | 0.00 | 11211_127 | 0.00 | 112 | 1.12 |
| 407_110 | 0.00 | 5257_155 | 1.67 | 10135_136 | 0.00 |  |  | 359_90 | 5.66 | 3905_149 | 4.72 | 7737_109 | 4.72 | 11213_47 | 1.89 | 113 | 10.64 |
| 415_83 | 1.67 | 5263_59 | 6.67 | 10138_58 | 8.33 |  |  | 360_93 | 9.43 | 3909_19 | 0.00 | 7738_73 | 2.83 | 11220_17 | 0.00 | 114 | 10.64 |
| 417_94 | 8.33 | 5273_174 | 13.33 | 10147_113 | 0.00 |  |  | 366_174 | 3.77 | 3913_31 | 10.38 | 7743_23 | 0.00 | 11225_125 | 0.00 | 115 | 1.32 |
| 418_76 | 3.33 | 5307_54 | 3.33 | 10148_9 | 3.33 |  |  | 369_149 | 1.89 | 3915_93 | 12.26 | 7746_81 | 6.60 | 11227_40 | 6.60 | 116 | 1.32 |
| 420_103 | 1.67 | 5308_74 | 0.00 | 10150_36 | 5.00 |  |  | 371_12 | 0.00 | 3916_26 | 9.43 | 7752_124 | 3.77 | 11234_35 | 1.89 | 117 | 4.54 |
| 423_78 | 0.00 | 5341_50 | 3.33 | 10154_107 | 3.33 |  |  | 379_49 | 11.32 | 3917_51 | 0.00 | 7756_111 | 7.55 | 11242_120 | 0.00 | 118 | 4.54 |
| 424_166 | 6.67 | 5353_91 | 1.67 | 10156_116 | 5.00 |  |  | 382_33 | 2.83 | 3928_117 | 0.94 | 7758_15 | 7.55 | 11243_114 | 0.94 | 119 | 0.97 |
| 425_85 | 0.00 | 5355_92 | 0.00 | 10157_55 | 0.00 |  |  | 385_58 | 0.00 | 3937_179 | 2.83 | 7761_179 | 12.26 | 11250_87 | 4.72 | 120 | 0.97 |
| 429_109 | 0.00 | 5358_50 | 6.67 | 10159_52 | 0.00 |  |  | 392_111 | 10.38 | 3939_20 | 2.83 | 7763_12 | 7.55 | 11251_31 | 0.94 | 121 | 0.64 |
| 431_127 | 0.00 | 5360_69 | 3.33 | 10165_53 | 1.67 |  |  | 396_47 | 0.00 | 3945_75 | 2.83 | 7768_66 | 3.77 | 11252_114 | 0.94 | 122 | 0.64 |
| 432_86 | 1.67 | 5362_64 | 6.67 | 10166_139 | 1.67 |  |  | 397_67 | 0.94 | 3948_7 | 8.49 | 7772_97 | 1.89 | 11254_57 | 8.49 | 123 | 1.08 |
| 439_113 | 8.33 | 5367_154 | 13.33 | 10175_43 | 1.67 |  |  | 399_83 | 9.43 | 3949_75 | 0.00 | 7776_7 | 7.55 | 11255_70 | 3.77 | 124 | 1.08 |
| 443_125 | 1.67 | 5377_103 | 1.67 | 10178_12 | 6.67 |  |  | 401_90 | 0.00 | 3951_82 | 0.94 | 7780_94 | 0.00 | 11256_7 | 8.49 | 125 | 0.77 |
| 444_84 | 6.67 | 5382_5 | 1.67 | 10182_65 | 1.67 |  |  | 407_110 | 0.00 | 3953_148 | 7.55 | 7784_51 | 0.00 | 11257_99 | 0.94 | 126 | 0.77 |
| 449_79 | 1.67 | 5394_34 | 5.00 | 10190_8 | 5.00 |  |  | 409_55 | 10.38 | 3955_10 | 3.77 | 7785_27 | 6.60 | 11259_120 | 0.00 | 127 | 3.11 |
| 459_91 | 6.67 | 5400_65 | 1.67 | 10193_21 | 1.67 |  |  | 410_78 | 0.00 | 3969_16 | 2.83 | 7787_108 | 1.89 | 11260_161 | 0.94 | 128 | 3.11 |
| 466_113 | 3.33 | 5402_52 | 0.00 | 10194_87 | 0.00 |  |  | 415_83 | 3.77 | 3971_127 | 0.00 | 7788_183 | 0.00 | 11271_73 | 1.89 | 129 | 22.32 |
| 471_54 | 5.00 | 5407_27 | 3.33 | 10196_131 | 6.67 |  |  | 417_94 | 5.66 | 3972_139 | 0.94 | 7797_56 | 3.77 | 11274_194 | 0.00 | 130 | 22.32 |
| 476_12 | 0.00 | 5418_26 | 10.00 | 10198_151 | 0.00 |  |  | 418_76 | 3.77 | 3973_80 | 0.94 | 7799_12 | 0.00 | 11278_100 | 2.83 | 131 | 0.68 |
| 477_178 | 6.67 | 5424_122 | 5.00 | 10199_88 | 6.67 |  |  | 420_103 | 1.89 | 3974_22 | 4.72 | 7804_75 | 0.94 | 11284_41 | 3.77 | 132 | 0.68 |
| 479_157 | 3.33 | 5428_120 | 1.67 | 10201_171 | 3.33 |  |  | 423_78 | 0.00 | 3976_155 | 5.66 | 7808_15 | 13.21 | 11286_91 | 3.77 | 133 | 27.88 |
| 485_93 | 3.33 | 5429_53 | 0.00 | 10206_12 | 1.67 |  |  | 424_166 | 8.49 | 3979_87 | 8.49 | 7815_139 | 5.66 | 11292_127 | 8.49 | 134 | 27.88 |
| 490_161 | 6.67 | 5430_70 | 0.00 | 10208_28 | 18.33 |  |  | 425_85 | 5.66 | 3980_159 | 0.94 | 7824_10 | 6.60 | 11293_111 | 0.00 | 135 | 3.31 |
| 494_123 | 6.67 | 5436_50 | 0.00 | 10214_98 | 3.33 |  |  | 429_109 | 0.00 | 3984_69 | 2.83 | 7825_86 | 2.83 | 11294_55 | 0.00 | 136 | 3.31 |
| 499_125 | 1.67 | 5437_73 | 1.67 | 10220_13 | 3.33 |  |  | 431_127 | 0.00 | 3986_59 | 0.94 | 7828_99 | 0.00 | 11295_9 | 1.89 | 137 | 2.64 |
| 503_94 | 6.67 | 5442_44 | 5.00 | 10223_38 | 0.00 |  |  | 432_86 | 8.49 | 3988_117 | 13.21 | 7830_57 | 12.26 | 11297_27 | 9.43 | 138 | 2.64 |
| 507_134 | 5.00 | 5447_13 | 0.00 | 10226_84 | 0.00 |  |  | 439_113 | 7.55 | 3990_68 | 0.94 | 7836_151 | 0.00 | 11298_127 | 2.83 | 139 | 2.38 |
| 516_83 | 1.67 | 5450_10 | 0.00 | 10227_131 | 11.67 |  |  | 440_161 | 5.66 | 3991_65 | 6.60 | 7837_157 | 2.83 | 11302_92 | 0.00 | 140 | 2.38 |
| 518_78 | 1.67 | 5455_85 | 0.00 | 10230_120 | 6.67 |  |  | 443_125 | 2.83 | 3992_61 | 10.38 | 7838_11 | 3.77 | 11304_10 | 1.89 | 141 | 2.67 |
| 520_108 | 10.00 | 5468_117 | 1.67 | 10232_95 | 1.67 |  |  | 444_84 | 5.66 | 3993_140 | 1.89 | 7839_134 | 2.83 | 11305_78 | 1.89 | 142 | 2.67 |
| 525_94 | 1.67 | 5478_92 | 0.00 | 10233_112 | 0.00 |  |  | 449_79 | 1.89 | 4004_14 | 10.38 | 7840_149 | 12.26 | 11307_54 | 2.83 | 143 | 2.89 |
| 526_42 | 8.33 | 5480_79 | 6.67 | 10237_34 | 1.67 |  |  | 457_44 | 2.83 | 4005_52 | 13.21 | 7844_93 | 0.94 | 11309_49 | 0.00 | 144 | 2.89 |
| 527_61 | 0.00 | 5484_63 | 3.33 | 10240_62 | 1.67 |  |  | 459_91 | 3.77 | 4006_50 | 7.55 | 7849_26 | 2.83 | 11310_67 | 1.89 | 145 | 2.86 |
| 532_131 | 1.67 | 5485_14 | 0.00 | 10243_141 | 1.67 |  |  | 466_113 | 1.89 | 4007_77 | 5.66 | 7852_67 | 0.94 | 11314_53 | 2.83 | 146 | 2.86 |
| 542_52 | 5.00 | 5489_94 | 0.00 | 10244_87 | 0.00 |  |  | 471_54 | 5.66 | 4009_93 | 13.21 | 7853_73 | 1.89 | 11316_15 | 3.77 | 147 | 9.26 |
| 547_23 | 1.67 | 5496_55 | 0.00 | 10245_80 | 0.00 |  |  | 476_12 | 0.94 | 4012_96 | 9.43 | 7854_32 | 0.00 | 11317_31 | 9.43 | 148 | 9.26 |
| 549_80 | 0.00 | 5497_6 | 1.67 | 10247_52 | 5.00 |  |  | 477_178 | 4.72 | 4013_6 | 0.00 | 7856_85 | 8.49 | 11320_142 | 10.38 | 149 | 1.32 |
| 551_153 | 1.67 | 5498_122 | 1.67 | 10248_19 | 0.00 |  |  | 478_70 | 0.00 | 4016_30 | 0.94 | 7861_35 | 0.00 | 11322_117 | 11.32 | 150 | 1.32 |
| 553_83 | 1.67 | 5501_26 | 8.33 | 10249_123 | 5.00 |  |  | 479_157 | 2.83 | 4025_11 | 6.60 | 7863_129 | 10.38 | 11329_20 | 0.00 | 151 | 2.34 |
| 554_75 | 0.00 | 5504_81 | 1.67 | 10259_8 | 1.67 |  |  | 480_83 | 2.83 | 4027_76 | 12.26 | 7864_41 | 0.94 | 11330_114 | 1.89 | 152 | 2.34 |
| 565_53 | 15.00 | 5506_134 | 8.33 | 10264_121 | 1.67 |  |  | 485_93 | 2.83 | 4030_8 | 0.94 | 7871_139 | 0.94 | 11331_35 | 8.49 | 153 | 1.28 |
| 566_36 | 1.67 | 5510_51 | 8.33 | 10265_97 | 0.00 |  |  | 490_161 | 4.72 | 4031_62 | 3.77 | 7872_14 | 0.00 | 11334_59 | 8.49 | 154 | 1.28 |
| 567_186 | 15.00 | 5514_70 | 0.00 | 10270_107 | 1.67 |  |  | 491_35 | 1.89 | 4040_27 | 2.83 | 7873_37 | 11.32 | 11335_156 | 3.77 | 155 | 13.99 |
| 568_82 | 3.33 | 5520_20 | 5.00 | 10272_125 | 10.00 |  |  | 494_123 | 4.72 | 4041_78 | 3.77 | 7876_40 | 7.55 | 11336_130 | 5.66 | 156 | 13.99 |
| 570_72 | 1.67 | 5528_119 | 1.67 | 10273_24 | 1.67 |  |  | 499_125 | 0.94 | 4049_64 | 0.00 | 7878_80 | 1.89 | 11339_120 | 13.21 | 157 | 5.33 |
| 571_82 | 0.00 | 5529_100 | 0.00 | 10277_70 | 0.00 |  |  | 503_94 | 7.55 | 4051_57 | 1.89 | 7879_75 | 0.00 | 11341_53 | 6.60 | 158 | 5.33 |
| 575_46 | 1.67 | 5530_182 | 3.33 | 10278_93 | 0.00 |  |  | 507_134 | 12.26 | 4052_93 | 0.94 | 7881_133 | 5.66 | 11342_146 | 0.94 | 159 | 2.93 |
| 576_79 | 0.00 | 5533_91 | 3.33 | 10280_75 | 0.00 |  |  | 512_104 | 1.89 | 4056_132 | 3.77 | 7883_129 | 1.89 | 11343_48 | 0.94 | 160 | 2.93 |
| 579_41 | 0.00 | 5536_78 | 1.67 | 10281_29 | 0.00 |  |  | 516_83 | 2.83 | 4059_141 | 3.77 | 7884_186 | 2.83 | 11346_47 | 0.00 | 161 | 1.85 |
| 580_32 | 3.33 | 5537_47 | 5.00 | 10283_32 | 0.00 |  |  | 518_78 | 0.94 | 4060_78 | 4.72 | 7890_89 | 4.72 | 11348_38 | 3.77 | 162 | 1.85 |
| 584_110 | 0.00 | 5541_111 | 0.00 | 10286_86 | 0.00 |  |  | 520_108 | 5.66 | 4064_123 | 0.00 | 7892_6 | 1.89 | 11350_130 | 0.00 | 163 | 3.00 |
| 586_74 | 3.33 | 5546_51 | 10.00 | 10288_103 | 5.00 |  |  | 525_94 | 3.77 | 4065_76 | 0.94 | 7895_145 | 0.00 | 11352_10 | 1.89 | 164 | 3.00 |
| 590_42 | 0.00 | 5555_24 | 6.67 | 10289_46 | 1.67 |  |  | 526_42 | 4.72 | 4066_44 | 0.00 | 7896_23 | 10.38 | 11354_56 | 2.83 | 165 | 0.88 |
| 591_120 | 1.67 | 5560_81 | 0.00 | 10292_161 | 5.00 |  |  | 527_61 | 0.94 | 4068_191 | 5.66 | 7901_27 | 0.00 | 11355_37 | 10.38 | 166 | 0.88 |
| 592_9 | 1.67 | 5564_40 | 0.00 | 10296_75 | 0.00 |  |  | 532_131 | 1.89 | 4073_16 | 1.89 | 7902_74 | 0.94 | 11362_132 | 0.00 | 167 | 1.61 |
| 597_129 | 1.67 | 5566_126 | 6.67 | 10297_38 | 1.67 |  |  | 542_52 | 7.55 | 4075_68 | 0.00 | 7905_111 | 2.83 | 11363_36 | 2.83 | 168 | 1.61 |
| 598_17 | 0.00 | 5572_62 | 1.67 | 10298_116 | 0.00 |  |  | 547_23 | 1.89 | 4078_143 | 0.94 | 7907_97 | 0.94 | 11369_38 | 3.77 | 169 | 1.06 |
| 599_103 | 0.00 | 5577_32 | 0.00 | 10299_111 | 1.67 |  |  | 549_80 | 0.00 | 4079_64 | 0.00 | 7912_93 | 0.94 | 11372_80 | 5.66 | 170 | 1.06 |
| 603_66 | 21.67 | 5579_60 | 0.00 | 10302_15 | 0.00 |  |  | 551_153 | 2.83 | 4080_141 | 10.38 | 7916_79 | 0.94 | 11374_103 | 0.00 | 171 | 0.82 |
| 607_75 | 1.67 | 5583_75 | 6.67 | 10304_24 | 6.67 |  |  | 553_83 | 6.60 | 4085_28 | 0.00 | 7919_89 | 0.00 | 11378_153 | 1.89 | 172 | 0.82 |
| 608_13 | 1.67 | 5584_26 | 1.67 | 10308_48 | 1.67 |  |  | 554_75 | 5.66 | 4086_72 | 4.72 | 7925_140 | 0.00 | 11379_39 | 0.00 | 173 | 14.92 |
| 617_129 | 0.00 | 5587_33 | 8.33 | 10309_69 | 1.67 |  |  | 559_122 | 8.49 | 4088_64 | 0.94 | 7926_43 | 0.94 | 11381_17 | 2.83 | 174 | 14.92 |
| 619_187 | 13.33 | 5591_29 | 10.00 | 10317_68 | 0.00 |  |  | 563_35 | 0.00 | 4090_70 | 6.60 | 7934_76 | 3.77 | 11385_32 | 8.49 | 175 | 0.84 |
| 623_141 | 1.67 | 5595_90 | 0.00 | 10318_57 | 0.00 |  |  | 565_53 | 15.09 | 4091_87 | 0.00 | 7935_101 | 0.00 | 11387_147 | 2.83 | 176 | 0.84 |
| 624_7 | 1.67 | 5604_21 | 0.00 | 10320_148 | 8.33 |  |  | 566_36 | 1.89 | 4092_126 | 3.77 | 7936_190 | 11.32 | 11388_120 | 9.43 | 177 | 1.45 |
| 633_5 | 16.67 | 5606_23 | 0.00 | 10322_44 | 0.00 |  |  | 567_186 | 12.26 | 4101_29 | 1.89 | 7938_79 | 0.00 | 11399_28 | 0.94 | 178 | 1.45 |
| 634_55 | 0.00 | 5612_119 | 5.00 | 10323_57 | 0.00 |  |  | 568_82 | 4.72 | 4104_100 | 12.26 | 7940_36 | 1.89 | 11400_58 | 9.43 | 179 | 0.82 |
| 636_26 | 6.67 | 5618_45 | 0.00 | 10324_138 | 3.33 |  |  | 570_72 | 1.89 | 4105_90 | 1.89 | 7947_132 | 0.00 | 11401_140 | 0.94 | 180 | 0.82 |
| 645_84 | 11.67 | 5619_155 | 1.67 | 10325_86 | 1.67 |  |  | 571_82 | 0.00 | 4106_21 | 3.77 | 7950_26 | 0.00 | 11402_13 | 3.77 | 181 | 3.46 |
| 654_161 | 10.00 | 5621_41 | 3.33 | 10326_53 | 0.00 |  |  | 573_18 | 0.00 | 4110_145 | 0.00 | 7952_117 | 1.89 | 11405_29 | 0.00 | 182 | 3.46 |
| 659_40 | 11.67 | 5622_6 | 0.00 | 10327_146 | 1.67 |  |  | 575_46 | 1.89 | 4111_144 | 6.60 | 7956_125 | 1.89 | 11407_72 | 3.77 | 183 | 0.88 |
| 666_52 | 1.67 | 5623_147 | 0.00 | 10337_158 | 10.00 |  |  | 576_79 | 0.00 | 4113_53 | 9.43 | 7957_104 | 2.83 | 11410_54 | 11.32 | 184 | 0.88 |
| 670_133 | 0.00 | 5625_72 | 1.67 | 10339_128 | 8.33 |  |  | 577_35 | 0.94 | 4116_88 | 6.60 | 7960_123 | 4.72 | 11416_33 | 1.89 | 185 | 0.77 |
| 671_118 | 1.67 | 5631_128 | 11.67 | 10341_132 | 5.00 |  |  | 578_42 | 2.83 | 4117_5 | 1.89 | 7972_29 | 0.94 | 11418_110 | 0.94 | 186 | 0.77 |
| 672_65 | 0.00 | 5632_76 | 25.00 | 10343_76 | 3.33 |  |  | 579_41 | 0.00 | 4120_85 | 0.00 | 7974_55 | 0.00 | 11421_89 | 6.60 | 187 | 2.60 |
| 674_119 | 10.00 | 5634_164 | 3.33 | 10344_7 | 5.00 |  |  | 580_32 | 7.55 | 4124_8 | 0.00 | 7977_85 | 0.00 | 11426_15 | 5.66 | 188 | 2.60 |
| 675_55 | 15.00 | 5636_44 | 0.00 | 10346_104 | 0.00 |  |  | 582_39 | 0.00 | 4128_107 | 6.60 | 7979_47 | 9.43 | 11432_24 | 1.89 | 189 | 1.72 |
| 676_130 | 1.67 | 5652_25 | 0.00 | 10349_48 | 3.33 |  |  | 584_110 | 0.00 | 4129_30 | 2.83 | 7987_70 | 0.00 | 11434_75 | 3.77 | 190 | 1.72 |
| 678_41 | 8.33 | 5653_16 | 1.67 | 10356_149 | 6.67 |  |  | 586_74 | 6.60 | 4132_109 | 10.38 | 7988_46 | 3.77 | 11440_149 | 2.83 | 191 | 0.75 |
| 683_143 | 1.67 | 5654_13 | 0.00 | 10360_119 | 3.33 |  |  | 590_42 | 0.94 | 4133_86 | 4.72 | 7989_49 | 0.00 | 11444_164 | 2.83 | 192 | 0.75 |
| 684_63 | 3.33 | 5655_87 | 1.67 | 10362_76 | 0.00 |  |  | 591_120 | 5.66 | 4135_27 | 0.00 | 7991_34 | 1.89 | 11445_81 | 0.00 | 193 | 2.67 |
| 701_68 | 1.67 | 5664_55 | 3.33 | 10367_82 | 11.67 |  |  | 592_9 | 0.94 | 4141_129 | 4.72 | 7992_84 | 9.43 | 11447_36 | 0.00 | 194 | 2.67 |
| 702_95 | 6.67 | 5667_87 | 6.67 | 10373_38 | 1.67 |  |  | 597_129 | 4.72 | 4145_23 | 7.55 | 7995_101 | 4.72 | 11451_119 | 14.15 | 195 | 1.59 |
| 707_121 | 1.67 | 5669_43 | 1.67 | 10379_92 | 1.67 |  |  | 598_17 | 0.00 | 4148_11 | 0.94 | 7997_9 | 10.38 | 11456_25 | 4.72 | 196 | 1.59 |
| 708_82 | 0.00 | 5670_128 | 8.33 | 10385_26 | 10.00 |  |  | 599_103 | 2.83 | 4150_62 | 0.00 | 7998_183 | 9.43 | 11461_193 | 8.49 | 197 | 2.91 |
| 712_127 | 3.33 | 5672_122 | 13.33 | 10386_80 | 1.67 |  |  | 603_66 | 15.09 | 4151_116 | 8.49 | 7999_48 | 5.66 | 11465_161 | 3.77 | 198 | 2.91 |
| 713_109 | 3.33 | 5676_77 | 5.00 | 10393_34 | 0.00 |  |  | 604_61 | 1.89 | 4154_153 | 0.00 | 8000_144 | 3.77 | 11467_5 | 2.83 | 199 | 1.52 |
| 714_38 | 0.00 | 5681_106 | 10.00 | 10394_65 | 0.00 |  |  | 607_75 | 0.94 | 4157_156 | 5.66 | 8001_93 | 8.49 | 11472_81 | 0.94 | 200 | 1.52 |
| 719_67 | 3.33 | 5686_74 | 3.33 | 10402_172 | 10.00 |  |  | 608_13 | 8.49 | 4165_67 | 11.32 | 8003_71 | 1.89 | 11480_11 | 2.83 | 201 | 1.15 |
| 722_183 | 3.33 | 5688_72 | 3.33 | 10407_92 | 0.00 |  |  | 617_129 | 0.00 | 4169_40 | 0.94 | 8005_131 | 9.43 | 11481_152 | 0.94 | 202 | 1.15 |
| 731_5 | 6.67 | 5689_93 | 5.00 | 10410_136 | 8.33 |  |  | 619_187 | 11.32 | 4170_96 | 8.49 | 8007_188 | 14.15 | 11484_71 | 0.00 | 203 | 9.21 |
| 734_74 | 3.33 | 5700_107 | 6.67 | 10412_182 | 8.33 |  |  | 623_141 | 1.89 | 4173_157 | 0.00 | 8009_31 | 1.89 | 11487_112 | 5.66 | 204 | 9.21 |
| 738_6 | 1.67 | 5708_149 | 1.67 | 10414_68 | 3.33 |  |  | 624_7 | 0.94 | 4174_153 | 0.94 | 8013_109 | 1.89 | 11488_150 | 2.83 | 205 | 1.17 |
| 743_87 | 0.00 | 5709_88 | 8.33 | 10416_76 | 1.67 |  |  | 629_62 | 0.00 | 4175_55 | 6.60 | 8015_53 | 0.00 | 11490_92 | 0.00 | 206 | 1.17 |
| 744_118 | 1.67 | 5710_8 | 6.67 | 10418_8 | 8.33 |  |  | 633_5 | 10.38 | 4180_172 | 10.38 | 8026_114 | 2.83 | 11493_45 | 6.60 | 207 | 2.01 |
| 747_106 | 3.33 | 5713_18 | 11.67 | 10419_13 | 0.00 |  |  | 634_55 | 0.00 | 4187_63 | 6.60 | 8030_82 | 0.00 | 11497_63 | 0.00 | 208 | 2.01 |
| 750_71 | 5.00 | 5714_152 | 8.33 | 10421_177 | 8.33 |  |  | 636_26 | 11.32 | 4189_123 | 3.77 | 8040_118 | 6.60 | 11498_10 | 0.94 | 209 | 3.02 |
| 751_195 | 11.67 | 5718_103 | 0.00 | 10425_78 | 0.00 |  |  | 645_84 | 13.21 | 4190_67 | 8.49 | 8044_26 | 0.94 | 11501_64 | 0.00 | 210 | 3.02 |
| 752_78 | 0.00 | 5719_169 | 0.00 | 10437_160 | 5.00 |  |  | 654_161 | 9.43 | 4195_138 | 0.00 | 8048_5 | 0.00 | 11505_136 | 1.89 | 211 | 0.75 |
| 753_22 | 0.00 | 5730_5 | 6.67 | 10439_56 | 1.67 |  |  | 659_40 | 10.38 | 4201_25 | 7.55 | 8055_96 | 0.94 | 11511_164 | 3.77 | 212 | 0.75 |
| 754_105 | 1.67 | 5742_88 | 8.33 | 10444_84 | 0.00 |  |  | 666_52 | 2.83 | 4204_28 | 0.00 | 8056_30 | 0.00 | 11515_8 | 0.00 |  |  |
| 758_93 | 6.67 | 5750_57 | 5.00 | 10445_82 | 5.00 |  |  | 670_133 | 0.94 | 4206_73 | 1.89 | 8059_82 | 1.89 | 11516_76 | 1.89 |  |  |
| 762_57 | 3.33 | 5754_46 | 1.67 | 10448_131 | 6.67 |  |  | 671_118 | 2.83 | 4209_11 | 5.66 | 8061_117 | 0.94 | 11518_70 | 4.72 |  |  |
| 765_13 | 6.67 | 5757_144 | 1.67 | 10449_112 | 0.00 |  |  | 672_65 | 0.94 | 4210_87 | 0.00 | 8065_19 | 0.94 | 11520_39 | 11.32 |  |  |
| 766_195 | 1.67 | 5758_111 | 0.00 | 10451_127 | 0.00 |  |  | 673_100 | 0.00 | 4214_37 | 0.00 | 8066_51 | 4.72 | 11522_138 | 7.55 |  |  |
| 771_74 | 0.00 | 5762_111 | 0.00 | 10452_126 | 1.67 |  |  | 674_119 | 10.38 | 4216_151 | 3.77 | 8067_94 | 4.72 | 11536_169 | 7.55 |  |  |
| 776_110 | 1.67 | 5772_55 | 10.00 | 10453_64 | 6.67 |  |  | 675_55 | 8.49 | 4221_30 | 10.38 | 8068_30 | 2.83 | 11540_44 | 0.00 |  |  |
| 780_160 | 6.67 | 5780_27 | 5.00 | 10454_172 | 6.67 |  |  | 676_130 | 3.77 | 4222_17 | 0.94 | 8069_130 | 0.00 | 11542_126 | 16.04 |  |  |
| 783_90 | 0.00 | 5788_69 | 0.00 | 10455_52 | 0.00 |  |  | 677_6 | 0.94 | 4225_51 | 10.38 | 8073_158 | 4.72 | 11548_131 | 2.83 |  |  |
| 784_56 | 0.00 | 5789_21 | 11.67 | 10456_67 | 8.33 |  |  | 678_41 | 6.60 | 4232_118 | 0.94 | 8074_21 | 0.94 | 11551_121 | 13.21 |  |  |
| 789_10 | 0.00 | 5790_87 | 10.00 | 10459_78 | 3.33 |  |  | 681_10 | 7.55 | 4234_94 | 8.49 | 8076_76 | 5.66 | 11557_25 | 1.89 |  |  |
| 792_182 | 0.00 | 5796_88 | 5.00 | 10460_129 | 6.67 |  |  | 683_143 | 0.94 | 4235_104 | 4.72 | 8077_29 | 11.32 | 11561_14 | 3.77 |  |  |
| 794_121 | 1.67 | 5798_110 | 1.67 | 10463_24 | 0.00 |  |  | 684_63 | 1.89 | 4239_99 | 2.83 | 8078_106 | 0.00 | 11562_157 | 0.00 |  |  |
| 806_162 | 0.00 | 5802_79 | 1.67 | 10470_153 | 0.00 |  |  | 697_50 | 0.00 | 4243_82 | 1.89 | 8081_86 | 4.72 | 11564_113 | 0.94 |  |  |
| 808_15 | 0.00 | 5811_30 | 1.67 | 10474_37 | 1.67 |  |  | 701_68 | 0.94 | 4244_100 | 11.32 | 8083_46 | 0.00 | 11566_26 | 0.00 |  |  |
| 818_98 | 3.33 | 5815_101 | 0.00 | 10485_41 | 0.00 |  |  | 702_95 | 5.66 | 4246_53 | 3.77 | 8084_67 | 9.43 | 11569_35 | 1.89 |  |  |
| 837_83 | 0.00 | 5819_104 | 1.67 | 10487_124 | 0.00 |  |  | 707_121 | 1.89 | 4247_40 | 0.00 | 8085_129 | 2.83 | 11570_129 | 12.26 |  |  |
| 838_71 | 3.33 | 5826_161 | 10.00 | 10502_19 | 0.00 |  |  | 708_82 | 0.00 | 4248_111 | 6.60 | 8090_82 | 1.89 | 11573_26 | 0.00 |  |  |
| 839_62 | 1.67 | 5830_40 | 1.67 | 10503_57 | 1.67 |  |  | 712_127 | 1.89 | 4252_81 | 0.94 | 8091_30 | 4.72 | 11574_99 | 1.89 |  |  |
| 841_105 | 0.00 | 5833_11 | 10.00 | 10505_39 | 8.33 |  |  | 713_109 | 2.83 | 4255_155 | 5.66 | 8096_86 | 0.00 | 11579_54 | 6.60 |  |  |
| 844_24 | 5.00 | 5841_70 | 0.00 | 10506_83 | 0.00 |  |  | 714_38 | 0.00 | 4257_131 | 0.94 | 8097_193 | 3.77 | 11590_143 | 4.72 |  |  |
| 852_35 | 1.67 | 5842_121 | 0.00 | 10514_29 | 3.33 |  |  | 719_67 | 2.83 | 4258_65 | 6.60 | 8101_146 | 4.72 | 11593_150 | 0.94 |  |  |
| 853_113 | 5.00 | 5843_65 | 0.00 | 10518_155 | 1.67 |  |  | 722_183 | 4.72 | 4266_177 | 6.60 | 8103_69 | 0.00 | 11595_86 | 0.94 |  |  |
| 855_75 | 8.33 | 5844_37 | 3.33 | 10521_52 | 3.33 |  |  | 728_192 | 0.00 | 4269_149 | 0.94 | 8104_67 | 6.60 | 11603_98 | 4.72 |  |  |
| 860_32 | 3.33 | 5848_48 | 0.00 | 10531_38 | 3.33 |  |  | 731_5 | 5.66 | 4271_107 | 1.89 | 8107_39 | 10.38 | 11606_73 | 2.83 |  |  |
| 871_74 | 5.00 | 5858_61 | 1.67 | 10533_74 | 5.00 |  |  | 734_74 | 6.60 | 4272_39 | 5.66 | 8111_77 | 0.00 | 11609_177 | 4.72 |  |  |
| 874_46 | 0.00 | 5860_30 | 3.33 | 10535_50 | 6.67 |  |  | 738_6 | 1.89 | 4274_186 | 10.38 | 8119_160 | 1.89 | 11611_10 | 0.94 |  |  |
| 891_34 | 0.00 | 5866_98 | 0.00 | 10536_121 | 1.67 |  |  | 743_87 | 0.94 | 4278_43 | 9.43 | 8120_79 | 0.94 | 11629_13 | 1.89 |  |  |
| 899_78 | 3.33 | 5871_113 | 10.00 | 10537_140 | 3.33 |  |  | 744_118 | 0.94 | 4280_46 | 1.89 | 8121_54 | 1.89 | 11633_110 | 0.94 |  |  |
| 900_94 | 6.67 | 5874_33 | 3.33 | 10538_32 | 0.00 |  |  | 747_106 | 1.89 | 4282_57 | 0.94 | 8124_49 | 7.55 | 11634_120 | 0.00 |  |  |
| 902_162 | 0.00 | 5880_116 | 1.67 | 10546_128 | 5.00 |  |  | 750_71 | 7.55 | 4285_99 | 7.55 | 8130_29 | 8.49 | 11636_124 | 0.00 |  |  |
| 910_85 | 0.00 | 5886_85 | 0.00 | 10554_85 | 1.67 |  |  | 751_195 | 11.32 | 4288_144 | 6.60 | 8133_82 | 10.38 | 11641_7 | 10.38 |  |  |
| 914_29 | 1.67 | 5891_9 | 3.33 | 10558_107 | 0.00 |  |  | 752_78 | 0.00 | 4298_160 | 0.94 | 8136_13 | 5.66 | 11643_61 | 6.60 |  |  |
| 923_30 | 1.67 | 5898_90 | 5.00 | 10559_66 | 8.33 |  |  | 753_22 | 2.83 | 4299_136 | 0.00 | 8139_111 | 8.49 | 11646_10 | 14.15 |  |  |
| 930_78 | 1.67 | 5899_33 | 3.33 | 10561_151 | 0.00 |  |  | 754_105 | 0.94 | 4301_130 | 10.38 | 8140_22 | 6.60 | 11657_149 | 0.94 |  |  |
| 939_173 | 1.67 | 5900_86 | 0.00 | 10564_151 | 13.33 |  |  | 758_93 | 10.38 | 4302_67 | 13.21 | 8143_190 | 4.72 | 11667_40 | 0.00 |  |  |
| 943_134 | 0.00 | 5901_48 | 0.00 | 10565_143 | 6.67 |  |  | 759_124 | 10.38 | 4306_41 | 11.32 | 8145_52 | 7.55 | 11669_27 | 4.72 |  |  |
| 952_27 | 11.67 | 5903_67 | 6.67 | 10575_114 | 0.00 |  |  | 762_57 | 1.89 | 4308_73 | 0.94 | 8148_56 | 0.00 | 11670_29 | 3.77 |  |  |
| 954_65 | 5.00 | 5904_72 | 10.00 | 10577_108 | 10.00 |  |  | 765_13 | 8.49 | 4314_84 | 4.72 | 8149_14 | 1.89 | 11686_120 | 0.94 |  |  |
| 956_98 | 0.00 | 5911_62 | 1.67 | 10582_166 | 8.33 |  |  | 766_195 | 0.94 | 4322_60 | 5.66 | 8150_188 | 6.60 | 11700_73 | 4.72 |  |  |
| 957_98 | 0.00 | 5913_122 | 3.33 | 10587_92 | 1.67 |  |  | 768_29 | 0.00 | 4326_67 | 0.94 | 8155_32 | 14.15 | 11712_110 | 5.66 |  |  |
| 961_59 | 5.00 | 5923_62 | 6.67 | 10590_134 | 5.00 |  |  | 771_74 | 0.00 | 4327_144 | 0.00 | 8158_79 | 0.94 | 11717_80 | 0.00 |  |  |
| 964_66 | 6.67 | 5927_149 | 0.00 | 10597_43 | 1.67 |  |  | 772_28 | 2.83 | 4331_73 | 5.66 | 8165_110 | 3.77 | 11718_145 | 10.38 |  |  |
| 972_158 | 3.33 | 5930_142 | 5.00 | 10616_100 | 0.00 |  |  | 776_110 | 1.89 | 4333_99 | 1.89 | 8166_56 | 5.66 | 11738_7 | 1.89 |  |  |
| 973_56 | 1.67 | 5931_24 | 0.00 | 10622_151 | 10.00 |  |  | 780_160 | 5.66 | 4334_8 | 0.00 | 8168_61 | 3.77 | 11742_116 | 6.60 |  |  |
| 978_46 | 0.00 | 5932_91 | 16.67 | 10628_32 | 0.00 |  |  | 782_187 | 1.89 | 4344_86 | 0.94 | 8170_148 | 0.00 | 11756_27 | 1.89 |  |  |
| 979_23 | 5.00 | 5936_132 | 6.67 | 10630_62 | 5.00 |  |  | 783_90 | 0.00 | 4346_164 | 5.66 | 8171_41 | 4.72 | 11768_147 | 4.72 |  |  |
| 981_51 | 1.67 | 5938_80 | 1.67 | 10640_132 | 6.67 |  |  | 784_56 | 0.00 | 4348_144 | 2.83 | 8175_74 | 2.83 | 11770_36 | 0.00 |  |  |
| 982_115 | 0.00 | 5941_110 | 3.33 | 10641_96 | 18.33 |  |  | 787_28 | 0.00 | 4349_43 | 0.00 | 8176_130 | 0.94 | 11773_72 | 2.83 |  |  |
| 983_92 | 15.00 | 5944_64 | 5.00 | 10643_20 | 6.67 |  |  | 789_10 | 2.83 | 4352_53 | 2.83 | 8177_86 | 0.00 | 11776_168 | 6.60 |  |  |
| 985_84 | 3.33 | 5948_61 | 1.67 | 10646_75 | 5.00 |  |  | 792_182 | 2.83 | 4357_6 | 0.94 | 8179_23 | 1.89 | 11783_135 | 0.94 |  |  |
| 992_124 | 0.00 | 5949_88 | 6.67 | 10647_43 | 1.67 |  |  | 794_121 | 1.89 | 4370_104 | 9.43 | 8182_24 | 4.72 | 11796_49 | 2.83 |  |  |
| 999_79 | 0.00 | 5970_21 | 15.00 | 10654_115 | 8.33 |  |  | 806_162 | 0.00 | 4372_8 | 6.60 | 8188_95 | 5.66 | 11798_62 | 0.94 |  |  |
| 1003_62 | 0.00 | 5973_74 | 0.00 | 10663_93 | 0.00 |  |  | 808_15 | 1.89 | 4373_117 | 1.89 | 8190_47 | 9.43 | 11806_56 | 12.26 |  |  |
| 1004_65 | 0.00 | 5983_44 | 8.33 | 10665_41 | 0.00 |  |  | 818_98 | 2.83 | 4384_97 | 3.77 | 8193_62 | 4.72 | 11819_135 | 10.38 |  |  |
| 1006_180 | 3.33 | 5986_86 | 3.33 | 10666_48 | 1.67 |  |  | 837_83 | 0.00 | 4389_155 | 12.26 | 8196_123 | 1.89 | 11822_72 | 5.66 |  |  |
| 1007_77 | 6.67 | 5988_66 | 1.67 | 10669_32 | 0.00 |  |  | 838_71 | 3.77 | 4390_154 | 0.00 | 8197_103 | 1.89 | 11832_40 | 1.89 |  |  |
| 1010_83 | 0.00 | 5990_17 | 0.00 | 10671_38 | 0.00 |  |  | 839_62 | 7.55 | 4392_89 | 0.94 | 8198_157 | 0.00 | 11837_120 | 2.83 |  |  |
| 1028_125 | 1.67 | 5991_30 | 8.33 | 10676_38 | 3.33 |  |  | 841_105 | 0.00 | 4394_83 | 0.00 | 8201_92 | 0.00 | 11839_114 | 11.32 |  |  |
| 1038_37 | 3.33 | 5992_151 | 5.00 | 10681_59 | 1.67 |  |  | 844_24 | 6.60 | 4398_5 | 2.83 | 8206_115 | 4.72 | 11842_121 | 0.00 |  |  |
| 1039_21 | 0.00 | 5994_56 | 0.00 | 10682_20 | 0.00 |  |  | 852_35 | 1.89 | 4405_106 | 2.83 | 8210_42 | 2.83 | 11845_121 | 0.94 |  |  |
| 1041_87 | 0.00 | 5995_67 | 3.33 | 10683_49 | 1.67 |  |  | 853_113 | 2.83 | 4407_64 | 4.72 | 8212_69 | 0.00 | 11849_7 | 4.72 |  |  |
| 1047_70 | 5.00 | 5998_33 | 0.00 | 10684_127 | 0.00 |  |  | 855_75 | 5.66 | 4408_13 | 0.00 | 8213_16 | 0.94 | 11855_82 | 6.60 |  |  |
| 1050_10 | 16.67 | 6003_18 | 1.67 | 10685_110 | 3.33 |  |  | 859_83 | 0.94 | 4418_35 | 3.77 | 8214_139 | 7.55 | 11857_125 | 1.89 |  |  |
| 1058_92 | 11.67 | 6010_162 | 1.67 | 10689_90 | 1.67 |  |  | 860_32 | 12.26 | 4425_122 | 4.72 | 8215_37 | 2.83 | 11859_77 | 5.66 |  |  |
| 1060_103 | 0.00 | 6011_103 | 0.00 | 10692_50 | 0.00 |  |  | 871_74 | 6.60 | 4426_13 | 2.83 | 8218_140 | 1.89 | 11864_64 | 1.89 |  |  |
| 1063_174 | 3.33 | 6012_37 | 0.00 | 10695_56 | 5.00 |  |  | 874_46 | 0.00 | 4427_137 | 2.83 | 8219_64 | 11.32 | 11865_30 | 10.38 |  |  |
| 1064_134 | 0.00 | 6014_47 | 3.33 | 10697_61 | 0.00 |  |  | 891_34 | 0.94 | 4428_100 | 0.94 | 8220_37 | 0.00 | 11866_119 | 1.89 |  |  |
| 1065_169 | 0.00 | 6017_90 | 1.67 | 10707_70 | 0.00 |  |  | 894_191 | 0.00 | 4431_102 | 1.89 | 8227_57 | 12.26 | 11867_90 | 0.94 |  |  |
| 1074_34 | 8.33 | 6019_14 | 0.00 | 10709_133 | 0.00 |  |  | 899_78 | 2.83 | 4432_133 | 1.89 | 8229_26 | 3.77 | 11873_51 | 7.55 |  |  |
| 1086_116 | 0.00 | 6021_6 | 5.00 | 10710_96 | 1.67 |  |  | 900_94 | 8.49 | 4434_167 | 4.72 | 8231_89 | 5.66 | 11882_150 | 13.21 |  |  |
| 1090_32 | 0.00 | 6023_62 | 6.67 | 10711_133 | 0.00 |  |  | 902_162 | 2.83 | 4437_53 | 0.00 | 8235_169 | 2.83 | 11883_90 | 5.66 |  |  |
| 1091_7 | 1.67 | 6032_70 | 3.33 | 10712_126 | 5.00 |  |  | 910_85 | 0.00 | 4439_48 | 0.94 | 8238_13 | 0.00 | 11886_194 | 13.21 |  |  |
| 1095_110 | 1.67 | 6033_57 | 16.67 | 10714_21 | 6.67 |  |  | 914_29 | 2.83 | 4441_65 | 6.60 | 8239_20 | 1.89 | 11896_137 | 9.43 |  |  |
| 1100_85 | 1.67 | 6038_9 | 0.00 | 10717_22 | 0.00 |  |  | 923_30 | 0.94 | 4443_24 | 3.77 | 8240_41 | 0.00 | 11899_31 | 0.00 |  |  |
| 1104_66 | 6.67 | 6039_68 | 0.00 | 10719_119 | 8.33 |  |  | 930_78 | 0.94 | 4450_67 | 2.83 | 8242_99 | 0.94 | 11910_32 | 4.72 |  |  |
| 1112_71 | 0.00 | 6042_75 | 5.00 | 10720_72 | 1.67 |  |  | 935_42 | 9.43 | 4452_97 | 1.89 | 8244_148 | 2.83 | 11921_112 | 4.72 |  |  |
| 1113_75 | 0.00 | 6045_47 | 3.33 | 10726_78 | 5.00 |  |  | 939_173 | 1.89 | 4454_102 | 8.49 | 8246_72 | 0.00 | 11923_112 | 3.77 |  |  |
| 1119_28 | 0.00 | 6046_46 | 3.33 | 10729_145 | 0.00 |  |  | 943_134 | 0.00 | 4456_95 | 1.89 | 8247_74 | 0.00 | 11927_135 | 0.94 |  |  |
| 1122_87 | 6.67 | 6049_74 | 6.67 | 10740_76 | 0.00 |  |  | 948_143 | 10.38 | 4457_30 | 4.72 | 8253_18 | 4.72 | 11929_40 | 1.89 |  |  |
| 1127_110 | 6.67 | 6051_107 | 3.33 | 10743_76 | 1.67 |  |  | 952_27 | 10.38 | 4467_99 | 0.94 | 8255_132 | 3.77 | 11930_125 | 4.72 |  |  |
| 1130_64 | 25.00 | 6055_168 | 1.67 | 10744_91 | 8.33 |  |  | 954_65 | 8.49 | 4471_20 | 6.60 | 8259_17 | 0.94 | 11944_149 | 4.72 |  |  |
| 1137_166 | 8.33 | 6056_142 | 0.00 | 10745_86 | 1.67 |  |  | 956_98 | 0.00 | 4474_6 | 0.94 | 8260_99 | 3.77 | 11952_157 | 6.60 |  |  |
| 1139_7 | 5.00 | 6062_6 | 0.00 | 10752_100 | 1.67 |  |  | 957_98 | 0.94 | 4475_11 | 0.00 | 8261_141 | 4.72 | 11968_190 | 1.89 |  |  |
| 1140_103 | 0.00 | 6069_131 | 1.67 | 10753_117 | 1.67 |  |  | 961_59 | 4.72 | 4477_73 | 0.00 | 8264_100 | 0.00 | 11969_85 | 0.00 |  |  |
| 1142_125 | 10.00 | 6075_39 | 3.33 | 10754_100 | 1.67 |  |  | 964_66 | 11.32 | 4479_68 | 0.00 | 8265_56 | 5.66 | 11975_21 | 0.00 |  |  |
| 1149_20 | 20.00 | 6081_17 | 0.00 | 10755_42 | 0.00 |  |  | 972_158 | 1.89 | 4486_9 | 0.00 | 8267_179 | 7.55 | 11978_89 | 10.38 |  |  |
| 1150_184 | 13.33 | 6085_187 | 5.00 | 10758_74 | 8.33 |  |  | 973_56 | 0.94 | 4488_60 | 6.60 | 8273_51 | 0.00 | 11990_187 | 10.38 |  |  |
| 1151_136 | 0.00 | 6087_72 | 5.00 | 10765_71 | 8.33 |  |  | 978_46 | 0.94 | 4490_185 | 5.66 | 8276_35 | 2.83 | 11998_76 | 3.77 |  |  |
| 1154_33 | 6.67 | 6094_83 | 8.33 | 10766_149 | 10.00 |  |  | 979_23 | 8.49 | 4491_71 | 0.00 | 8277_83 | 0.00 | 12017_107 | 5.66 |  |  |
| 1159_26 | 20.00 | 6096_28 | 6.67 | 10770_62 | 1.67 |  |  | 981_51 | 3.77 | 4501_72 | 9.43 | 8279_43 | 8.49 | 12030_9 | 14.15 |  |  |
| 1161_63 | 1.67 | 6097_106 | 0.00 | 10771_128 | 8.33 |  |  | 982_115 | 0.00 | 4502_18 | 0.00 | 8281_37 | 0.00 | 12042_191 | 5.66 |  |  |
| 1164_84 | 5.00 | 6102_93 | 1.67 | 10773_13 | 8.33 |  |  | 983_92 | 12.26 | 4503_79 | 9.43 | 8288_10 | 3.77 | 12056_23 | 0.94 |  |  |
| 1174_6 | 1.67 | 6103_98 | 1.67 | 10775_52 | 0.00 |  |  | 984_101 | 1.89 | 4506_22 | 1.89 | 8290_159 | 14.15 | 12061_166 | 6.60 |  |  |
| 1176_107 | 6.67 | 6104_129 | 3.33 | 10778_15 | 1.67 |  |  | 985_84 | 3.77 | 4507_109 | 0.00 | 8291_125 | 0.00 | 12065_79 | 8.49 |  |  |
| 1182_140 | 0.00 | 6108_108 | 5.00 | 10780_99 | 3.33 |  |  | 992_124 | 0.94 | 4509_72 | 0.00 | 8296_96 | 0.94 | 12073_154 | 6.60 |  |  |
| 1184_112 | 0.00 | 6112_91 | 1.67 | 10783_165 | 3.33 |  |  | 999_79 | 4.72 | 4511_95 | 5.66 | 8297_102 | 0.00 | 12080_34 | 0.00 |  |  |
| 1185_60 | 3.33 | 6113_46 | 3.33 | 10793_67 | 0.00 |  |  | 1003_62 | 0.94 | 4515_39 | 12.26 | 8301_187 | 5.66 | 12082_122 | 1.89 |  |  |
| 1188_19 | 1.67 | 6117_18 | 1.67 | 10804_49 | 0.00 |  |  | 1004_65 | 0.00 | 4520_44 | 14.15 | 8303_54 | 0.00 | 12083_104 | 0.94 |  |  |
| 1190_152 | 0.00 | 6118_33 | 1.67 | 10805_17 | 5.00 |  |  | 1006_180 | 5.66 | 4521_183 | 9.43 | 8312_36 | 2.83 | 12092_152 | 5.66 |  |  |
| 1191_91 | 21.67 | 6120_143 | 0.00 | 10811_55 | 3.33 |  |  | 1007_77 | 5.66 | 4528_171 | 1.89 | 8313_111 | 0.94 | 12098_49 | 4.72 |  |  |
| 1193_24 | 5.00 | 6125_18 | 3.33 | 10814_166 | 0.00 |  |  | 1010_83 | 0.00 | 4534_92 | 0.00 | 8317_116 | 7.55 | 12102_37 | 7.55 |  |  |
| 1197_121 | 5.00 | 6127_41 | 1.67 | 10820_43 | 1.67 |  |  | 1020_59 | 0.94 | 4536_176 | 0.94 | 8318_55 | 1.89 | 12116_36 | 3.77 |  |  |
| 1199_92 | 0.00 | 6128_86 | 8.33 | 10823_14 | 0.00 |  |  | 1028_125 | 0.94 | 4539_50 | 0.94 | 8322_119 | 2.83 | 12117_190 | 6.60 |  |  |
| 1200_129 | 1.67 | 6129_111 | 10.00 | 10824_44 | 26.67 |  |  | 1038_37 | 1.89 | 4540_158 | 3.77 | 8324_64 | 11.32 | 12119_23 | 2.83 |  |  |
| 1201_20 | 5.00 | 6150_27 | 8.33 | 10826_49 | 3.33 |  |  | 1039_21 | 0.00 | 4541_137 | 2.83 | 8329_177 | 5.66 | 12123_23 | 0.00 |  |  |
| 1206_164 | 1.67 | 6151_111 | 1.67 | 10827_78 | 5.00 |  |  | 1041_87 | 0.00 | 4544_91 | 0.00 | 8335_6 | 0.94 | 12126_36 | 7.55 |  |  |
| 1219_17 | 1.67 | 6155_114 | 0.00 | 10832_128 | 1.67 |  |  | 1046_11 | 4.72 | 4545_128 | 11.32 | 8337_54 | 4.72 | 12129_130 | 1.89 |  |  |
| 1224_91 | 3.33 | 6158_46 | 0.00 | 10834_60 | 5.00 |  |  | 1047_70 | 2.83 | 4548_120 | 4.72 | 8338_37 | 3.77 | 12131_40 | 0.00 |  |  |
| 1225_73 | 3.33 | 6159_160 | 5.00 | 10838_91 | 11.67 |  |  | 1050_10 | 12.26 | 4559_160 | 6.60 | 8339_98 | 0.00 | 12132_154 | 0.94 |  |  |
| 1229_21 | 1.67 | 6160_121 | 13.33 | 10844_84 | 1.67 |  |  | 1056_62 | 7.55 | 4563_55 | 1.89 | 8344_42 | 2.83 | 12140_17 | 4.72 |  |  |
| 1230_72 | 3.33 | 6162_116 | 1.67 | 10845_105 | 1.67 |  |  | 1058_92 | 10.38 | 4566_85 | 0.00 | 8345_120 | 3.77 | 12144_20 | 0.94 |  |  |
| 1231_30 | 1.67 | 6166_23 | 0.00 | 10848_74 | 5.00 |  |  | 1060_103 | 0.00 | 4574_129 | 0.00 | 8348_136 | 0.00 | 12147_93 | 12.26 |  |  |
| 1233_183 | 16.67 | 6168_139 | 6.67 | 10849_65 | 8.33 |  |  | 1063_174 | 2.83 | 4581_60 | 0.00 | 8352_107 | 1.89 | 12151_11 | 0.00 |  |  |
| 1234_71 | 6.67 | 6170_75 | 0.00 | 10851_59 | 1.67 |  |  | 1064_134 | 0.00 | 4583_109 | 0.00 | 8353_119 | 1.89 | 12162_35 | 1.89 |  |  |
| 1239_142 | 1.67 | 6171_65 | 5.00 | 10852_170 | 11.67 |  |  | 1065_169 | 0.00 | 4585_158 | 9.43 | 8354_110 | 0.00 | 12163_12 | 2.83 |  |  |
| 1241_129 | 1.67 | 6172_178 | 1.67 | 10853_9 | 0.00 |  |  | 1074_34 | 9.43 | 4596_177 | 6.60 | 8357_72 | 0.00 | 12164_82 | 4.72 |  |  |
| 1243_46 | 0.00 | 6177_35 | 6.67 | 10854_7 | 6.67 |  |  | 1080_51 | 2.83 | 4598_35 | 0.00 | 8358_67 | 0.00 | 12173_127 | 2.83 |  |  |
| 1245_191 | 0.00 | 6179_12 | 0.00 | 10858_5 | 0.00 |  |  | 1086_116 | 0.00 | 4600_37 | 5.66 | 8365_30 | 0.00 | 12180_99 | 2.83 |  |  |
| 1248_17 | 11.67 | 6180_128 | 0.00 | 10859_33 | 11.67 |  |  | 1090_32 | 0.00 | 4603_122 | 1.89 | 8366_68 | 2.83 | 12181_86 | 3.77 |  |  |
| 1250_42 | 6.67 | 6187_92 | 1.67 | 10860_156 | 5.00 |  |  | 1091_7 | 4.72 | 4604_10 | 3.77 | 8377_54 | 0.00 | 12183_11 | 3.77 |  |  |
| 1254_131 | 11.67 | 6191_190 | 8.33 | 10861_95 | 1.67 |  |  | 1095_110 | 0.94 | 4611_31 | 6.60 | 8380_107 | 0.00 | 12199_134 | 0.00 |  |  |
| 1258_77 | 11.67 | 6202_38 | 0.00 | 10865_26 | 0.00 |  |  | 1099_10 | 0.00 | 4613_104 | 7.55 | 8382_22 | 3.77 | 12200_58 | 2.83 |  |  |
| 1262_60 | 0.00 | 6209_48 | 16.67 | 10870_11 | 1.67 |  |  | 1100_85 | 0.94 | 4622_128 | 0.00 | 8383_94 | 0.00 | 12204_107 | 11.32 |  |  |
| 1263_174 | 3.33 | 6217_87 | 6.67 | 10875_134 | 6.67 |  |  | 1104_66 | 5.66 | 4623_56 | 0.94 | 8384_108 | 4.72 | 12207_142 | 7.55 |  |  |
| 1264_57 | 1.67 | 6220_25 | 0.00 | 10879_129 | 3.33 |  |  | 1112_71 | 0.00 | 4624_34 | 10.38 | 8385_64 | 0.00 | 12209_125 | 10.38 |  |  |
| 1267_33 | 1.67 | 6221_55 | 10.00 | 10880_156 | 13.33 |  |  | 1113_75 | 0.00 | 4627_186 | 5.66 | 8392_31 | 7.55 | 12214_69 | 0.00 |  |  |
| 1268_183 | 8.33 | 6225_133 | 0.00 | 10896_73 | 1.67 |  |  | 1119_28 | 0.94 | 4629_101 | 0.94 | 8394_26 | 0.00 | 12230_75 | 0.00 |  |  |
| 1270_19 | 0.00 | 6230_32 | 5.00 | 10898_123 | 3.33 |  |  | 1122_87 | 12.26 | 4631_152 | 6.60 | 8396_28 | 0.00 | 12232_33 | 0.94 |  |  |
| 1272_70 | 3.33 | 6240_103 | 0.00 | 10899_43 | 0.00 |  |  | 1127_110 | 6.60 | 4633_207 | 12.26 | 8399_44 | 4.72 | 12239_121 | 3.77 |  |  |
| 1281_93 | 1.67 | 6242_46 | 1.67 | 10900_27 | 0.00 |  |  | 1130_64 | 14.15 | 4639_130 | 2.83 | 8401_83 | 0.00 | 12242_188 | 6.60 |  |  |
| 1284_154 | 8.33 | 6245_82 | 1.67 | 10901_90 | 5.00 |  |  | 1137_166 | 11.32 | 4642_83 | 12.26 | 8402_5 | 1.89 | 12246_74 | 0.94 |  |  |
| 1288_106 | 8.33 | 6246_127 | 3.33 | 10909_43 | 1.67 |  |  | 1139_7 | 3.77 | 4646_79 | 12.26 | 8403_51 | 1.89 | 12247_38 | 0.94 |  |  |
| 1296_129 | 1.67 | 6247_40 | 6.67 | 10915_7 | 0.00 |  |  | 1140_103 | 0.00 | 4650_134 | 3.77 | 8408_139 | 11.32 | 12251_109 | 8.49 |  |  |
| 1305_101 | 0.00 | 6251_164 | 5.00 | 10916_58 | 1.67 |  |  | 1142_125 | 11.32 | 4655_25 | 4.72 | 8412_152 | 2.83 | 12257_112 | 5.66 |  |  |
| 1308_134 | 1.67 | 6260_72 | 6.67 | 10921_195 | 3.33 |  |  | 1143_82 | 0.00 | 4657_15 | 1.89 | 8417_47 | 9.43 | 12258_33 | 5.66 |  |  |
| 1313_122 | 8.33 | 6261_15 | 3.33 | 10929_84 | 0.00 |  |  | 1149_20 | 12.26 | 4668_88 | 0.94 | 8418_102 | 4.72 | 12272_79 | 0.94 |  |  |
| 1314_91 | 1.67 | 6276_109 | 1.67 | 10934_84 | 3.33 |  |  | 1150_184 | 10.38 | 4669_46 | 4.72 | 8427_95 | 1.89 | 12273_194 | 12.26 |  |  |
| 1319_192 | 1.67 | 6280_72 | 3.33 | 10936_158 | 3.33 |  |  | 1151_136 | 0.00 | 4677_96 | 10.38 | 8429_63 | 8.49 | 12276_128 | 3.77 |  |  |
| 1321_86 | 0.00 | 6295_108 | 1.67 | 10937_51 | 0.00 |  |  | 1154_33 | 9.43 | 4681_112 | 0.94 | 8430_37 | 2.83 | 12278_87 | 1.89 |  |  |
| 1323_58 | 1.67 | 6300_134 | 0.00 | 10946_93 | 10.00 |  |  | 1159_26 | 13.21 | 4682_51 | 0.00 | 8435_8 | 3.77 | 12287_198 | 8.49 |  |  |
| 1326_18 | 0.00 | 6303_76 | 0.00 | 10959_125 | 3.33 |  |  | 1161_63 | 3.77 | 4683_77 | 0.00 | 8440_136 | 8.49 | 12294_56 | 6.60 |  |  |
| 1328_44 | 1.67 | 6311_5 | 0.00 | 10960_68 | 10.00 |  |  | 1164_84 | 5.66 | 4690_98 | 10.38 | 8452_172 | 0.94 | 12295_31 | 8.49 |  |  |
| 1337_125 | 0.00 | 6312_25 | 0.00 | 10962_109 | 1.67 |  |  | 1174_6 | 1.89 | 4693_5 | 0.94 | 8460_13 | 4.72 | 12296_61 | 0.94 |  |  |
| 1339_52 | 0.00 | 6314_63 | 0.00 | 10964_42 | 1.67 |  |  | 1176_107 | 4.72 | 4701_46 | 0.00 | 8461_52 | 2.83 | 12297_175 | 5.66 |  |  |
| 1340_66 | 1.67 | 6316_48 | 6.67 | 10971_73 | 0.00 |  |  | 1177_9 | 0.94 | 4705_41 | 5.66 | 8462_42 | 0.94 | 12300_5 | 1.89 |  |  |
| 1348_115 | 10.00 | 6318_107 | 8.33 | 10992_98 | 0.00 |  |  | 1180_55 | 1.89 | 4711_95 | 2.83 | 8467_9 | 0.00 | 12303_64 | 12.26 |  |  |
| 1357_147 | 0.00 | 6330_5 | 5.00 | 10994_43 | 6.67 |  |  | 1182_140 | 2.83 | 4717_38 | 2.83 | 8468_116 | 0.94 | 12312_124 | 0.00 |  |  |
| 1361_121 | 8.33 | 6350_105 | 1.67 | 10997_43 | 0.00 |  |  | 1184_112 | 0.00 | 4723_36 | 2.83 | 8470_105 | 0.00 | 12325_147 | 0.00 |  |  |
| 1365_96 | 3.33 | 6351_20 | 5.00 | 10998_169 | 5.00 |  |  | 1185_60 | 3.77 | 4728_114 | 7.55 | 8479_139 | 0.00 | 12333_112 | 3.77 |  |  |
| 1368_59 | 3.33 | 6353_13 | 10.00 | 11001_100 | 3.33 |  |  | 1188_19 | 3.77 | 4729_170 | 0.94 | 8483_105 | 11.32 | 12346_92 | 10.38 |  |  |
| 1370_134 | 5.00 | 6355_44 | 5.00 | 11002_55 | 5.00 |  |  | 1190_152 | 0.00 | 4730_45 | 11.32 | 8485_37 | 6.60 | 12352_36 | 0.94 |  |  |
| 1371_90 | 6.67 | 6359_17 | 6.67 | 11004_91 | 0.00 |  |  | 1191_91 | 14.15 | 4732_98 | 1.89 | 8486_78 | 14.15 | 12355_48 | 5.66 |  |  |
| 1374_62 | 1.67 | 6362_50 | 1.67 | 11007_157 | 18.33 |  |  | 1193_24 | 7.55 | 4733_66 | 10.38 | 8488_85 | 0.00 | 12365_121 | 7.55 |  |  |
| 1378_155 | 0.00 | 6367_11 | 11.67 | 11013_31 | 1.67 |  |  | 1197_121 | 4.72 | 4735_20 | 0.00 | 8490_96 | 2.83 | 12369_165 | 11.32 |  |  |
| 1379_85 | 0.00 | 6368_53 | 0.00 | 11015_165 | 5.00 |  |  | 1199_92 | 0.00 | 4737_10 | 11.32 | 8493_142 | 5.66 | 12372_42 | 3.77 |  |  |
| 1380_118 | 5.00 | 6369_14 | 10.00 | 11018_5 | 6.67 |  |  | 1200_129 | 1.89 | 4741_74 | 0.00 | 8495_20 | 7.55 | 12377_121 | 0.00 |  |  |
| 1381_116 | 1.67 | 6371_78 | 3.33 | 11025_55 | 3.33 |  |  | 1201_20 | 4.72 | 4746_96 | 1.89 | 8499_168 | 2.83 | 12378_189 | 11.32 |  |  |
| 1382_160 | 3.33 | 6373_74 | 1.67 | 11028_114 | 3.33 |  |  | 1206_164 | 3.77 | 4750_57 | 0.94 | 8501_63 | 3.77 | 12382_83 | 7.55 |  |  |
| 1384_78 | 3.33 | 6375_32 | 0.00 | 11031_24 | 5.00 |  |  | 1212_45 | 2.83 | 4752_89 | 0.00 | 8504_38 | 1.89 | 12384_10 | 10.38 |  |  |
| 1386_55 | 1.67 | 6376_59 | 3.33 | 11038_138 | 3.33 |  |  | 1219_17 | 0.94 | 4755_86 | 5.66 | 8505_56 | 3.77 | 12389_49 | 4.72 |  |  |
| 1387_54 | 0.00 | 6378_5 | 0.00 | 11053_53 | 0.00 |  |  | 1224_91 | 1.89 | 4758_190 | 2.83 | 8507_73 | 0.00 | 12394_93 | 0.94 |  |  |
| 1392_129 | 0.00 | 6380_175 | 8.33 | 11064_29 | 0.00 |  |  | 1225_73 | 2.83 | 4764_170 | 10.38 | 8511_135 | 0.94 | 12403_128 | 0.00 |  |  |
| 1393_41 | 0.00 | 6382_68 | 1.67 | 11072_95 | 1.67 |  |  | 1229_21 | 1.89 | 4765_102 | 0.00 | 8512_71 | 2.83 | 12410_98 | 11.32 |  |  |
| 1394_32 | 6.67 | 6384_67 | 1.67 | 11077_27 | 15.00 |  |  | 1230_72 | 3.77 | 4766_102 | 4.72 | 8517_34 | 5.66 | 12411_146 | 4.72 |  |  |
| 1399_50 | 0.00 | 6386_44 | 5.00 | 11078_21 | 5.00 |  |  | 1231_30 | 0.94 | 4768_14 | 12.26 | 8520_22 | 5.66 | 12412_57 | 0.00 |  |  |
| 1405_92 | 0.00 | 6387_114 | 8.33 | 11085_104 | 1.67 |  |  | 1233_183 | 10.38 | 4770_62 | 0.94 | 8522_8 | 1.89 | 12415_14 | 0.00 |  |  |
| 1407_36 | 1.67 | 6389_103 | 10.00 | 11086_18 | 1.67 |  |  | 1234_71 | 4.72 | 4771_79 | 3.77 | 8523_99 | 0.00 | 12418_84 | 4.72 |  |  |
| 1409_24 | 8.33 | 6393_130 | 5.00 | 11089_96 | 3.33 |  |  | 1239_142 | 0.94 | 4772_35 | 11.32 | 8524_12 | 9.43 | 12420_58 | 11.32 |  |  |
| 1414_133 | 0.00 | 6397_119 | 1.67 | 11100_164 | 0.00 |  |  | 1241_129 | 1.89 | 4773_56 | 0.94 | 8525_139 | 7.55 | 12424_106 | 11.32 |  |  |
| 1416_66 | 15.00 | 6398_143 | 1.67 | 11104_13 | 3.33 |  |  | 1243_46 | 0.94 | 4774_42 | 0.00 | 8529_75 | 1.89 | 12426_103 | 0.00 |  |  |
| 1419_162 | 1.67 | 6406_171 | 11.67 | 11109_55 | 3.33 |  |  | 1245_191 | 0.00 | 4778_84 | 0.00 | 8531_32 | 11.32 | 12428_169 | 8.49 |  |  |
| 1420_83 | 8.33 | 6407_154 | 5.00 | 11118_159 | 0.00 |  |  | 1246_32 | 6.60 | 4779_63 | 2.83 | 8533_31 | 0.00 | 12429_130 | 10.38 |  |  |
| 1423_69 | 11.67 | 6408_52 | 0.00 | 11121_45 | 0.00 |  |  | 1248_17 | 15.09 | 4786_103 | 0.94 | 8534_98 | 1.89 | 12430_85 | 7.55 |  |  |
| 1424_30 | 0.00 | 6412_22 | 0.00 | 11122_29 | 1.67 |  |  | 1250_42 | 3.77 | 4787_12 | 3.77 | 8538_173 | 9.43 | 12431_87 | 6.60 |  |  |
| 1426_71 | 8.33 | 6413_116 | 0.00 | 11123_27 | 8.33 |  |  | 1254_131 | 11.32 | 4789_32 | 0.00 | 8541_62 | 0.00 | 12436_110 | 0.94 |  |  |
| 1431_82 | 1.67 | 6415_120 | 0.00 | 11125_58 | 0.00 |  |  | 1258_77 | 10.38 | 4791_89 | 6.60 | 8542_177 | 2.83 | 12437_58 | 6.60 |  |  |
| 1440_100 | 0.00 | 6417_103 | 15.00 | 11126_27 | 0.00 |  |  | 1262_60 | 0.00 | 4794_9 | 0.00 | 8547_92 | 1.89 | 12438_72 | 2.83 |  |  |
| 1443_72 | 5.00 | 6423_20 | 10.00 | 11137_152 | 0.00 |  |  | 1263_174 | 2.83 | 4795_138 | 2.83 | 8556_78 | 0.94 | 12447_82 | 0.00 |  |  |
| 1444_102 | 1.67 | 6431_95 | 0.00 | 11140_13 | 0.00 |  |  | 1264_57 | 4.72 | 4796_17 | 11.32 | 8561_66 | 0.00 | 12461_69 | 4.72 |  |  |
| 1445_144 | 11.67 | 6433_35 | 0.00 | 11141_13 | 6.67 |  |  | 1267_33 | 0.94 | 4812_83 | 10.38 | 8563_41 | 0.00 | 12473_118 | 6.60 |  |  |
| 1449_23 | 0.00 | 6435_74 | 1.67 | 11142_103 | 3.33 |  |  | 1268_183 | 14.15 | 4813_142 | 5.66 | 8567_30 | 0.00 | 12475_107 | 0.94 |  |  |
| 1453_122 | 0.00 | 6436_57 | 1.67 | 11149_124 | 5.00 |  |  | 1270_19 | 0.00 | 4815_12 | 6.60 | 8570_19 | 5.66 | 12493_104 | 7.55 |  |  |
| 1456_84 | 0.00 | 6444_60 | 5.00 | 11150_67 | 3.33 |  |  | 1272_70 | 12.26 | 4817_82 | 7.55 | 8572_9 | 2.83 | 12504_81 | 11.32 |  |  |
| 1458_43 | 1.67 | 6446_13 | 5.00 | 11154_92 | 1.67 |  |  | 1281_93 | 0.94 | 4819_145 | 0.94 | 8574_44 | 0.94 | 12508_49 | 0.94 |  |  |
| 1468_82 | 3.33 | 6452_40 | 0.00 | 11156_106 | 0.00 |  |  | 1284_154 | 8.49 | 4820_157 | 0.00 | 8575_68 | 6.60 | 12515_84 | 7.55 |  |  |
| 1470_154 | 0.00 | 6455_62 | 3.33 | 11159_91 | 8.33 |  |  | 1288_106 | 9.43 | 4821_25 | 0.94 | 8580_12 | 0.00 | 12521_75 | 0.00 |  |  |
| 1481_90 | 1.67 | 6456_7 | 1.67 | 11162_94 | 25.00 |  |  | 1296_129 | 2.83 | 4825_152 | 1.89 | 8581_32 | 0.00 | 12525_104 | 0.00 |  |  |
| 1484_36 | 3.33 | 6457_90 | 10.00 | 11168_95 | 0.00 |  |  | 1305_101 | 0.00 | 4826_11 | 1.89 | 8585_75 | 0.00 | 12528_93 | 7.55 |  |  |
| 1487_27 | 0.00 | 6463_90 | 1.67 | 11172_135 | 11.67 |  |  | 1308_134 | 1.89 | 4829_97 | 9.43 | 8592_89 | 14.15 | 12538_71 | 0.94 |  |  |
| 1493_107 | 6.67 | 6467_103 | 0.00 | 11176_50 | 0.00 |  |  | 1313_122 | 4.72 | 4835_151 | 1.89 | 8593_15 | 6.60 | 12541_84 | 0.00 |  |  |
| 1494_10 | 3.33 | 6470_51 | 13.33 | 11181_113 | 0.00 |  |  | 1314_91 | 1.89 | 4836_39 | 4.72 | 8597_158 | 8.49 | 12543_50 | 4.72 |  |  |
| 1495_91 | 0.00 | 6477_74 | 1.67 | 11185_72 | 8.33 |  |  | 1319_192 | 0.94 | 4840_57 | 0.94 | 8602_38 | 0.00 | 12544_35 | 6.60 |  |  |
| 1496_110 | 0.00 | 6480_20 | 3.33 | 11189_78 | 0.00 |  |  | 1321_86 | 0.00 | 4853_39 | 0.94 | 8606_70 | 0.00 | 12547_89 | 2.83 |  |  |
| 1501_103 | 5.00 | 6482_72 | 1.67 | 11190_37 | 3.33 |  |  | 1323_58 | 0.94 | 4861_85 | 0.00 | 8611_115 | 0.00 | 12553_24 | 0.00 |  |  |
| 1502_27 | 1.67 | 6485_86 | 5.00 | 11195_53 | 0.00 |  |  | 1326_18 | 0.00 | 4872_152 | 8.49 | 8616_48 | 0.94 | 12557_35 | 0.94 |  |  |
| 1503_16 | 3.33 | 6489_79 | 3.33 | 11197_144 | 5.00 |  |  | 1328_44 | 0.94 | 4874_8 | 13.21 | 8618_140 | 0.00 | 12568_61 | 1.89 |  |  |
| 1507_69 | 0.00 | 6491_29 | 11.67 | 11204_62 | 0.00 |  |  | 1330_13 | 0.00 | 4875_51 | 0.00 | 8619_96 | 0.94 | 12575_245 | 16.04 |  |  |
| 1517_47 | 0.00 | 6492_6 | 0.00 | 11206_45 | 1.67 |  |  | 1337_125 | 0.00 | 4877_121 | 2.83 | 8621_33 | 6.60 | 12577_89 | 7.55 |  |  |
| 1521_111 | 1.67 | 6496_7 | 3.33 | 11207_75 | 5.00 |  |  | 1339_52 | 0.00 | 4878_87 | 0.94 | 8624_15 | 1.89 | 12578_15 | 12.26 |  |  |
| 1523_39 | 0.00 | 6500_22 | 3.33 | 11208_85 | 5.00 |  |  | 1340_66 | 1.89 | 4879_137 | 0.00 | 8626_13 | 0.00 | 12580_13 | 12.26 |  |  |
| 1524_124 | 0.00 | 6501_63 | 10.00 | 11211_127 | 0.00 |  |  | 1348_115 | 10.38 | 4880_24 | 8.49 | 8628_98 | 0.00 | 12581_26 | 2.83 |  |  |
| 1526_83 | 0.00 | 6508_96 | 10.00 | 11213_47 | 1.67 |  |  | 1351_8 | 3.77 | 4881_30 | 0.00 | 8633_74 | 6.60 | 12584_29 | 0.94 |  |  |
| 1527_102 | 0.00 | 6512_107 | 3.33 | 11220_17 | 0.00 |  |  | 1357_147 | 0.00 | 4890_38 | 0.00 | 8637_72 | 0.00 | 12597_21 | 5.66 |  |  |
| 1528_41 | 0.00 | 6518_33 | 5.00 | 11225_125 | 0.00 |  |  | 1361_121 | 6.60 | 4895_82 | 1.89 | 8639_18 | 0.00 | 12601_48 | 4.72 |  |  |
| 1535_68 | 0.00 | 6521_31 | 0.00 | 11227_40 | 5.00 |  |  | 1362_7 | 0.00 | 4897_65 | 1.89 | 8640_151 | 0.00 | 12605_118 | 0.94 |  |  |
| 1538_25 | 0.00 | 6528_98 | 0.00 | 11234_35 | 0.00 |  |  | 1365_96 | 2.83 | 4898_74 | 6.60 | 8641_101 | 1.89 | 12610_157 | 1.89 |  |  |
| 1540_84 | 0.00 | 6533_90 | 6.67 | 11250_87 | 6.67 |  |  | 1368_59 | 10.38 | 4902_113 | 4.72 | 8642_86 | 0.94 | 12611_16 | 6.60 |  |  |
| 1542_42 | 0.00 | 6534_44 | 0.00 | 11251_31 | 0.00 |  |  | 1370_134 | 3.77 | 4903_92 | 9.43 | 8643_83 | 8.49 | 12612_31 | 0.00 |  |  |
| 1547_53 | 0.00 | 6536_86 | 0.00 | 11252_114 | 1.67 |  |  | 1371_90 | 6.60 | 4904_61 | 6.60 | 8648_78 | 0.94 | 12616_12 | 4.72 |  |  |
| 1551_13 | 0.00 | 6538_18 | 0.00 | 11254_57 | 8.33 |  |  | 1374_62 | 1.89 | 4909_42 | 8.49 | 8651_88 | 5.66 | 12617_19 | 7.55 |  |  |
| 1557_90 | 3.33 | 6543_117 | 0.00 | 11255_70 | 3.33 |  |  | 1378_155 | 4.72 | 4910_85 | 0.00 | 8652_35 | 0.94 | 12618_175 | 0.94 |  |  |
| 1570_101 | 5.00 | 6548_8 | 10.00 | 11256_7 | 11.67 |  |  | 1379_85 | 3.77 | 4911_110 | 1.89 | 8654_81 | 0.00 | 12624_172 | 5.66 |  |  |
| 1573_89 | 0.00 | 6550_194 | 10.00 | 11257_99 | 1.67 |  |  | 1380_118 | 2.83 | 4914_30 | 0.94 | 8663_72 | 0.00 | 12644_171 | 10.38 |  |  |
| 1574_96 | 3.33 | 6555_121 | 5.00 | 11259_120 | 0.00 |  |  | 1381_116 | 0.94 | 4915_33 | 1.89 | 8674_151 | 1.89 | 12650_60 | 8.49 |  |  |
| 1579_30 | 1.67 | 6565_57 | 1.67 | 11260_161 | 0.00 |  |  | 1382_160 | 1.89 | 4917_75 | 2.83 | 8679_49 | 0.94 | 12654_90 | 0.94 |  |  |
| 1582_78 | 15.00 | 6567_63 | 8.33 | 11271_73 | 3.33 |  |  | 1384_78 | 3.77 | 4922_129 | 0.94 | 8680_136 | 12.26 | 12658_185 | 8.49 |  |  |
| 1584_44 | 0.00 | 6568_55 | 0.00 | 11274_194 | 0.00 |  |  | 1386_55 | 5.66 | 4925_60 | 0.00 | 8686_90 | 10.38 | 12659_102 | 5.66 |  |  |
| 1586_98 | 0.00 | 6581_29 | 1.67 | 11278_100 | 1.67 |  |  | 1387_54 | 0.00 | 4932_175 | 14.15 | 8688_28 | 0.00 | 12670_166 | 4.72 |  |  |
| 1589_51 | 5.00 | 6582_5 | 5.00 | 11284_41 | 5.00 |  |  | 1392_129 | 0.00 | 4934_45 | 7.55 | 8694_150 | 0.00 | 12673_29 | 0.94 |  |  |
| 1590_136 | 0.00 | 6589_63 | 1.67 | 11286_91 | 1.67 |  |  | 1393_41 | 0.00 | 4936_45 | 10.38 | 8697_7 | 0.00 | 12674_91 | 0.00 |  |  |
| 1593_19 | 1.67 | 6595_136 | 8.33 | 11292_127 | 5.00 |  |  | 1394_32 | 8.49 | 4938_140 | 1.89 | 8702_128 | 0.00 | 12678_126 | 0.94 |  |  |
| 1595_150 | 1.67 | 6598_118 | 0.00 | 11293_111 | 0.00 |  |  | 1399_50 | 0.00 | 4941_122 | 1.89 | 8704_18 | 10.38 | 12680_32 | 14.15 |  |  |
| 1596_141 | 1.67 | 6603_123 | 18.33 | 11294_55 | 0.00 |  |  | 1403_8 | 0.94 | 4948_116 | 1.89 | 8706_22 | 1.89 | 12685_106 | 4.72 |  |  |
| 1600_24 | 0.00 | 6620_45 | 3.33 | 11295_9 | 1.67 |  |  | 1405_92 | 0.00 | 4949_71 | 1.89 | 8715_88 | 0.00 | 12692_104 | 1.89 |  |  |
| 1603_37 | 0.00 | 6621_90 | 3.33 | 11297_27 | 15.00 |  |  | 1407_36 | 0.94 | 4951_83 | 10.38 | 8719_97 | 1.89 | 12695_9 | 13.21 |  |  |
| 1606_97 | 6.67 | 6623_62 | 13.33 | 11298_127 | 0.00 |  |  | 1409_24 | 10.38 | 4952_82 | 11.32 | 8721_57 | 12.26 | 12700_84 | 1.89 |  |  |
| 1609_53 | 11.67 | 6625_120 | 1.67 | 11302_92 | 0.00 |  |  | 1414_133 | 0.00 | 4956_39 | 1.89 | 8723_133 | 10.38 | 12703_86 | 0.00 |  |  |
| 1610_51 | 3.33 | 6626_33 | 3.33 | 11304_10 | 0.00 |  |  | 1416_66 | 12.26 | 4957_25 | 0.00 | 8727_182 | 2.83 | 12721_13 | 10.38 |  |  |
| 1612_6 | 10.00 | 6628_104 | 0.00 | 11305_78 | 3.33 |  |  | 1419_162 | 0.94 | 4958_152 | 2.83 | 8729_65 | 0.00 | 12722_62 | 0.94 |  |  |
| 1615_155 | 0.00 | 6629_85 | 0.00 | 11307_54 | 3.33 |  |  | 1420_83 | 8.49 | 4961_139 | 1.89 | 8730_67 | 10.38 | 12724_83 | 10.38 |  |  |
| 1616_165 | 5.00 | 6636_160 | 0.00 | 11309_49 | 0.00 |  |  | 1423_69 | 11.32 | 4963_27 | 7.55 | 8731_77 | 0.00 | 12727_102 | 2.83 |  |  |
| 1618_120 | 0.00 | 6637_73 | 10.00 | 11310_67 | 1.67 |  |  | 1424_30 | 4.72 | 4964_14 | 15.09 | 8739_177 | 4.72 | 12731_57 | 6.60 |  |  |
| 1623_133 | 6.67 | 6639_46 | 1.67 | 11314_53 | 5.00 |  |  | 1426_71 | 10.38 | 4965_148 | 10.38 | 8746_40 | 3.77 | 12732_123 | 5.66 |  |  |
| 1625_66 | 0.00 | 6641_78 | 0.00 | 11320_142 | 11.67 |  |  | 1431_82 | 2.83 | 4976_99 | 0.94 | 8751_127 | 2.83 | 12743_93 | 0.00 |  |  |
| 1637_30 | 1.67 | 6642_39 | 8.33 | 11322_117 | 5.00 |  |  | 1438_92 | 3.77 | 4978_8 | 0.00 | 8753_33 | 0.94 | 12747_59 | 0.00 |  |  |
| 1642_16 | 3.33 | 6643_73 | 5.00 | 11329_20 | 0.00 |  |  | 1440_100 | 0.00 | 4980_185 | 0.00 | 8757_175 | 0.94 | 12748_130 | 1.89 |  |  |
| 1643_59 | 0.00 | 6654_73 | 1.67 | 11330_114 | 1.67 |  |  | 1441_54 | 9.43 | 4984_55 | 3.77 | 8759_177 | 3.77 | 12751_134 | 0.94 |  |  |
| 1644_53 | 0.00 | 6661_151 | 5.00 | 11331_35 | 6.67 |  |  | 1443_72 | 3.77 | 4985_129 | 2.83 | 8763_173 | 8.49 | 12753_133 | 10.38 |  |  |
| 1646_45 | 3.33 | 6662_130 | 0.00 | 11334_59 | 8.33 |  |  | 1444_102 | 0.94 | 4986_70 | 0.00 | 8767_81 | 0.00 | 12757_136 | 1.89 |  |  |
| 1649_26 | 0.00 | 6663_52 | 1.67 | 11335_156 | 5.00 |  |  | 1445_144 | 9.43 | 4990_77 | 7.55 | 8769_102 | 0.00 | 12763_69 | 3.77 |  |  |
| 1664_72 | 13.33 | 6664_83 | 0.00 | 11336_130 | 5.00 |  |  | 1449_23 | 0.00 | 4995_83 | 3.77 | 8770_11 | 0.00 | 12765_82 | 0.94 |  |  |
| 1668_46 | 0.00 | 6665_42 | 8.33 | 11339_120 | 18.33 |  |  | 1453_122 | 0.00 | 5000_16 | 1.89 | 8772_134 | 6.60 | 12769_14 | 6.60 |  |  |
| 1669_31 | 0.00 | 6666_151 | 8.33 | 11341_53 | 5.00 |  |  | 1456_84 | 0.00 | 5002_157 | 4.72 | 8777_29 | 5.66 | 12772_166 | 16.04 |  |  |
| 1673_57 | 0.00 | 6669_192 | 3.33 | 11342_146 | 1.67 |  |  | 1457_109 | 1.89 | 5005_51 | 0.94 | 8782_79 | 1.89 | 12774_138 | 2.83 |  |  |
| 1676_123 | 1.67 | 6684_104 | 0.00 | 11346_47 | 0.00 |  |  | 1458_43 | 0.94 | 5006_26 | 0.00 | 8785_5 | 0.94 | 12777_108 | 4.72 |  |  |
| 1680_122 | 1.67 | 6699_185 | 11.67 | 11348_38 | 3.33 |  |  | 1465_63 | 2.83 | 5007_73 | 4.72 | 8786_59 | 0.94 | 12784_44 | 6.60 |  |  |
| 1684_40 | 6.67 | 6701_10 | 0.00 | 11350_130 | 0.00 |  |  | 1468_82 | 2.83 | 5009_94 | 1.89 | 8790_46 | 6.60 | 12785_150 | 4.72 |  |  |
| 1688_93 | 0.00 | 6702_128 | 0.00 | 11352_10 | 1.67 |  |  | 1470_154 | 0.00 | 5012_112 | 12.26 | 8791_52 | 2.83 | 12791_104 | 5.66 |  |  |
| 1691_164 | 5.00 | 6704_16 | 0.00 | 11354_56 | 5.00 |  |  | 1481_90 | 0.94 | 5014_19 | 0.00 | 8803_46 | 8.49 | 12793_155 | 1.89 |  |  |
| 1692_31 | 10.00 | 6707_14 | 1.67 | 11355_37 | 5.00 |  |  | 1484_36 | 5.66 | 5015_65 | 1.89 | 8805_153 | 2.83 | 12796_5 | 1.89 |  |  |
| 1694_102 | 0.00 | 6708_76 | 1.67 | 11362_132 | 0.00 |  |  | 1487_27 | 0.00 | 5024_113 | 8.49 | 8809_38 | 1.89 | 12798_109 | 7.55 |  |  |
| 1697_92 | 0.00 | 6710_19 | 1.67 | 11363_36 | 1.67 |  |  | 1488_121 | 1.89 | 5026_133 | 4.72 | 8810_29 | 9.43 | 12804_19 | 0.94 |  |  |
| 1700_151 | 11.67 | 6713_132 | 0.00 | 11369_38 | 3.33 |  |  | 1493_107 | 6.60 | 5031_41 | 7.55 | 8812_38 | 15.09 | 12810_23 | 2.83 |  |  |
| 1701_165 | 1.67 | 6715_9 | 0.00 | 11372_80 | 5.00 |  |  | 1494_10 | 6.60 | 5032_21 | 2.83 | 8813_107 | 0.00 | 12811_156 | 0.00 |  |  |
| 1702_195 | 0.00 | 6717_35 | 1.67 | 11374_103 | 0.00 |  |  | 1495_91 | 0.00 | 5033_175 | 0.94 | 8814_101 | 3.77 | 12812_127 | 0.94 |  |  |
| 1706_23 | 0.00 | 6721_193 | 16.67 | 11378_153 | 3.33 |  |  | 1496_110 | 0.00 | 5037_121 | 3.77 | 8816_79 | 0.00 | 12815_72 | 10.38 |  |  |
| 1713_13 | 1.67 | 6722_6 | 0.00 | 11379_39 | 0.00 |  |  | 1501_103 | 8.49 | 5042_47 | 4.72 | 8817_7 | 2.83 | 12819_132 | 8.49 |  |  |
| 1714_56 | 0.00 | 6730_101 | 3.33 | 11381_17 | 1.67 |  |  | 1502_27 | 2.83 | 5045_67 | 0.00 | 8820_106 | 7.55 | 12833_175 | 0.94 |  |  |
| 1719_64 | 0.00 | 6733_17 | 10.00 | 11385_32 | 5.00 |  |  | 1503_16 | 1.89 | 5047_132 | 1.89 | 8821_33 | 0.00 | 12835_56 | 11.32 |  |  |
| 1721_140 | 1.67 | 6736_143 | 0.00 | 11387_147 | 0.00 |  |  | 1504_125 | 5.66 | 5049_32 | 7.55 | 8826_37 | 0.00 | 12846_60 | 8.49 |  |  |
| 1725_90 | 1.67 | 6742_106 | 1.67 | 11388_120 | 6.67 |  |  | 1507_69 | 0.00 | 5056_66 | 0.00 | 8829_46 | 0.00 | 12852_169 | 0.00 |  |  |
| 1727_99 | 0.00 | 6743_119 | 10.00 | 11399_28 | 0.00 |  |  | 1517_47 | 0.00 | 5057_63 | 14.15 | 8830_123 | 0.00 | 12856_59 | 0.94 |  |  |
| 1730_84 | 3.33 | 6749_87 | 13.33 | 11400_58 | 3.33 |  |  | 1521_111 | 0.94 | 5058_38 | 5.66 | 8832_116 | 0.00 | 12861_133 | 4.72 |  |  |
| 1734_82 | 0.00 | 6753_28 | 0.00 | 11401_140 | 1.67 |  |  | 1523_39 | 0.00 | 5060_24 | 4.72 | 8837_180 | 5.66 | 12863_147 | 3.77 |  |  |
| 1736_120 | 0.00 | 6754_23 | 1.67 | 11402_13 | 3.33 |  |  | 1524_124 | 2.83 | 5062_114 | 0.00 | 8838_125 | 10.38 | 12873_150 | 0.00 |  |  |
| 1742_113 | 6.67 | 6756_26 | 8.33 | 11405_29 | 0.00 |  |  | 1526_83 | 1.89 | 5063_14 | 0.94 | 8842_121 | 2.83 | 12885_65 | 5.66 |  |  |
| 1744_84 | 0.00 | 6767_79 | 0.00 | 11407_72 | 3.33 |  |  | 1527_102 | 0.00 | 5064_51 | 0.94 | 8848_67 | 0.00 | 12889_60 | 0.00 |  |  |
| 1749_101 | 10.00 | 6772_86 | 0.00 | 11410_54 | 10.00 |  |  | 1528_41 | 4.72 | 5069_58 | 14.15 | 8855_40 | 0.00 | 12892_64 | 3.77 |  |  |
| 1755_110 | 1.67 | 6775_53 | 10.00 | 11416_33 | 1.67 |  |  | 1535_68 | 0.00 | 5072_61 | 1.89 | 8857_42 | 1.89 | 12900_121 | 0.00 |  |  |
| 1759_72 | 3.33 | 6778_121 | 0.00 | 11418_110 | 1.67 |  |  | 1538_25 | 0.00 | 5073_5 | 0.00 | 8858_164 | 4.72 | 12908_22 | 5.66 |  |  |
| 1760_9 | 1.67 | 6779_70 | 5.00 | 11421_89 | 5.00 |  |  | 1540_84 | 0.00 | 5077_57 | 0.00 | 8868_44 | 7.55 | 12913_52 | 0.00 |  |  |
| 1767_47 | 3.33 | 6780_168 | 1.67 | 11426_15 | 10.00 |  |  | 1542_42 | 0.94 | 5078_183 | 9.43 | 8871_93 | 0.00 | 12918_26 | 3.77 |  |  |
| 1768_44 | 1.67 | 6790_62 | 0.00 | 11432_24 | 1.67 |  |  | 1547_53 | 0.00 | 5079_117 | 8.49 | 8872_82 | 0.00 | 12924_134 | 3.77 |  |  |
| 1776_68 | 0.00 | 6793_22 | 3.33 | 11434_75 | 3.33 |  |  | 1551_13 | 0.00 | 5082_63 | 0.00 | 8883_81 | 6.60 | 12927_192 | 8.49 |  |  |
| 1779_18 | 5.00 | 6798_109 | 0.00 | 11440_149 | 0.00 |  |  | 1556_141 | 4.72 | 5083_84 | 2.83 | 8887_101 | 0.00 | 12930_69 | 12.26 |  |  |
| 1780_59 | 1.67 | 6801_114 | 3.33 | 11444_164 | 3.33 |  |  | 1557_90 | 1.89 | 5085_19 | 2.83 | 8889_74 | 5.66 | 12935_76 | 0.00 |  |  |
| 1785_39 | 3.33 | 6809_44 | 1.67 | 11445_81 | 0.00 |  |  | 1559_38 | 0.00 | 5090_95 | 0.00 | 8890_66 | 0.00 | 12938_65 | 2.83 |  |  |
| 1786_158 | 6.67 | 6810_130 | 0.00 | 11447_36 | 0.00 |  |  | 1570_101 | 14.15 | 5096_194 | 4.72 | 8893_73 | 0.00 | 12941_118 | 0.94 |  |  |
| 1793_59 | 0.00 | 6811_100 | 1.67 | 11451_119 | 10.00 |  |  | 1573_89 | 0.00 | 5098_178 | 6.60 | 8894_138 | 0.00 | 12946_81 | 8.49 |  |  |
| 1794_8 | 10.00 | 6817_30 | 0.00 | 11456_25 | 5.00 |  |  | 1574_96 | 5.66 | 5099_48 | 4.72 | 8897_117 | 0.00 | 12947_142 | 0.94 |  |  |
| 1796_132 | 1.67 | 6818_13 | 0.00 | 11461_193 | 15.00 |  |  | 1579_30 | 0.94 | 5105_52 | 0.00 | 8898_22 | 9.43 | 12955_120 | 6.60 |  |  |
| 1797_28 | 0.00 | 6823_80 | 1.67 | 11465_161 | 3.33 |  |  | 1582_78 | 10.38 | 5108_79 | 12.26 | 8900_192 | 9.43 | 12969_122 | 6.60 |  |  |
| 1802_160 | 6.67 | 6825_60 | 3.33 | 11467_5 | 1.67 |  |  | 1583_138 | 0.00 | 5115_37 | 2.83 | 8901_40 | 0.00 | 12973_31 | 0.94 |  |  |
| 1806_92 | 5.00 | 6829_72 | 5.00 | 11472_81 | 0.00 |  |  | 1584_44 | 0.00 | 5116_17 | 1.89 | 8907_148 | 1.89 | 12981_5 | 0.94 |  |  |
| 1808_87 | 0.00 | 6832_120 | 1.67 | 11480_11 | 3.33 |  |  | 1586_98 | 0.00 | 5117_145 | 1.89 | 8908_169 | 12.26 | 12983_124 | 0.00 |  |  |
| 1809_38 | 3.33 | 6837_68 | 0.00 | 11484_71 | 0.00 |  |  | 1589_51 | 2.83 | 5130_36 | 1.89 | 8912_60 | 3.77 | 12988_71 | 8.49 |  |  |
| 1810_152 | 3.33 | 6839_89 | 0.00 | 11487_112 | 1.67 |  |  | 1590_136 | 0.00 | 5135_47 | 0.94 | 8915_90 | 0.94 | 12992_175 | 10.38 |  |  |
| 1813_59 | 0.00 | 6841_45 | 0.00 | 11488_150 | 1.67 |  |  | 1593_19 | 1.89 | 5138_135 | 5.66 | 8919_79 | 11.32 | 12994_15 | 0.00 |  |  |
| 1817_11 | 0.00 | 6844_41 | 0.00 | 11490_92 | 0.00 |  |  | 1595_150 | 1.89 | 5139_51 | 6.60 | 8923_57 | 0.94 | 12995_58 | 14.15 |  |  |
| 1822_97 | 1.67 | 6848_154 | 3.33 | 11493_45 | 1.67 |  |  | 1596_141 | 1.89 | 5141_194 | 8.49 | 8924_21 | 0.94 | 12999_124 | 0.94 |  |  |
| 1825_107 | 13.33 | 6866_75 | 5.00 | 11498_10 | 0.00 |  |  | 1599_105 | 8.49 | 5146_98 | 0.00 | 8925_106 | 7.55 | 13002_30 | 6.60 |  |  |
| 1827_54 | 0.00 | 6867_123 | 5.00 | 11501_64 | 0.00 |  |  | 1600_24 | 0.00 | 5149_70 | 6.60 | 8926_38 | 0.00 | 13004_8 | 6.60 |  |  |
| 1834_136 | 5.00 | 6877_17 | 8.33 | 11505_136 | 1.67 |  |  | 1603_37 | 0.00 | 5152_45 | 10.38 | 8929_103 | 5.66 | 13012_173 | 10.38 |  |  |
| 1846_75 | 5.00 | 6884_54 | 3.33 | 11511_164 | 5.00 |  |  | 1605_16 | 1.89 | 5153_43 | 0.00 | 8931_109 | 0.94 | 13018_75 | 0.00 |  |  |
| 1857_153 | 3.33 | 6885_178 | 5.00 | 11515_8 | 0.00 |  |  | 1606_97 | 11.32 | 5157_7 | 0.94 | 8932_168 | 1.89 | 13020_177 | 11.32 |  |  |
| 1858_60 | 5.00 | 6889_43 | 0.00 | 11516_76 | 0.00 |  |  | 1609_53 | 10.38 | 5161_91 | 8.49 | 8933_66 | 0.94 | 13024_115 | 4.72 |  |  |
| 1861_38 | 6.67 | 6897_84 | 3.33 | 11518_70 | 0.00 |  |  | 1610_51 | 5.66 | 5164_23 | 4.72 | 8936_65 | 9.43 | 13028_95 | 2.83 |  |  |
| 1862_8 | 5.00 | 6901_142 | 3.33 | 11520_39 | 10.00 |  |  | 1612_6 | 6.60 | 5182_42 | 0.94 | 8938_150 | 0.00 | 13034_36 | 0.00 |  |  |
| 1863_100 | 0.00 | 6908_115 | 11.67 | 11522_138 | 11.67 |  |  | 1615_155 | 0.00 | 5185_150 | 7.55 | 8943_80 | 0.94 | 13037_130 | 13.21 |  |  |
| 1865_90 | 6.67 | 6909_122 | 3.33 | 11536_169 | 8.33 |  |  | 1616_165 | 7.55 | 5188_13 | 3.77 | 8945_140 | 10.38 | 13039_142 | 13.21 |  |  |
| 1868_129 | 0.00 | 6914_153 | 13.33 | 11540_44 | 0.00 |  |  | 1618_120 | 0.94 | 5189_127 | 0.00 | 8946_48 | 6.60 | 13041_84 | 9.43 |  |  |
| 1870_89 | 0.00 | 6915_63 | 10.00 | 11542_126 | 20.00 |  |  | 1620_72 | 5.66 | 5190_40 | 2.83 | 8948_110 | 0.94 | 13044_43 | 5.66 |  |  |
| 1875_49 | 8.33 | 6920_187 | 1.67 | 11548_131 | 1.67 |  |  | 1623_133 | 3.77 | 5191_44 | 4.72 | 8952_109 | 9.43 | 13055_30 | 0.00 |  |  |
| 1878_51 | 8.33 | 6921_28 | 1.67 | 11551_121 | 10.00 |  |  | 1625_66 | 0.00 | 5203_9 | 5.66 | 8953_97 | 3.77 | 13057_145 | 2.83 |  |  |
| 1882_19 | 0.00 | 6923_46 | 0.00 | 11557_25 | 3.33 |  |  | 1637_30 | 1.89 | 5224_41 | 3.77 | 8955_116 | 1.89 | 13059_98 | 6.60 |  |  |
| 1883_70 | 6.67 | 6927_112 | 1.67 | 11561_14 | 6.67 |  |  | 1638_116 | 0.00 | 5257_155 | 6.60 | 8956_76 | 7.55 | 13060_30 | 12.26 |  |  |
| 1884_59 | 8.33 | 6936_6 | 0.00 | 11562_157 | 0.00 |  |  | 1642_16 | 1.89 | 5263_59 | 4.72 | 8959_39 | 7.55 | 13066_67 | 7.55 |  |  |
| 1887_67 | 8.33 | 6940_33 | 0.00 | 11564_113 | 1.67 |  |  | 1643_59 | 0.94 | 5273_174 | 7.55 | 8962_50 | 0.00 | 13068_194 | 10.38 |  |  |
| 1888_149 | 13.33 | 6942_80 | 0.00 | 11566_26 | 0.00 |  |  | 1644_53 | 0.94 | 5276_146 | 7.55 | 8967_42 | 12.26 | 13072_34 | 0.94 |  |  |
| 1892_85 | 10.00 | 6943_105 | 0.00 | 11569_35 | 1.67 |  |  | 1646_45 | 9.43 | 5307_54 | 2.83 | 8968_67 | 1.89 | 13085_192 | 2.83 |  |  |
| 1898_25 | 1.67 | 6944_55 | 1.67 | 11570_129 | 11.67 |  |  | 1649_26 | 0.00 | 5308_74 | 1.89 | 8970_16 | 9.43 | 13094_105 | 9.43 |  |  |
| 1900_78 | 0.00 | 6947_85 | 0.00 | 11573_26 | 0.00 |  |  | 1664_72 | 9.43 | 5341_50 | 1.89 | 8973_172 | 5.66 | 13096_126 | 0.00 |  |  |
| 1901_140 | 0.00 | 6951_65 | 1.67 | 11574_99 | 1.67 |  |  | 1668_46 | 0.00 | 5353_91 | 0.94 | 8976_18 | 0.00 | 13099_40 | 0.00 |  |  |
| 1902_103 | 0.00 | 6955_23 | 5.00 | 11579_54 | 3.33 |  |  | 1669_31 | 0.00 | 5355_92 | 0.00 | 8977_110 | 2.83 | 13101_41 | 10.38 |  |  |
| 1908_81 | 0.00 | 6956_56 | 1.67 | 11590_143 | 8.33 |  |  | 1673_57 | 0.00 | 5358_50 | 7.55 | 8978_159 | 10.38 | 13105_40 | 11.32 |  |  |
| 1909_38 | 0.00 | 6958_133 | 0.00 | 11593_150 | 1.67 |  |  | 1676_123 | 0.94 | 5360_69 | 4.72 | 8979_130 | 0.94 | 13106_128 | 11.32 |  |  |
| 1913_131 | 1.67 | 6961_73 | 0.00 | 11595_86 | 1.67 |  |  | 1678_44 | 13.21 | 5362_64 | 4.72 | 8982_139 | 6.60 | 13110_126 | 4.72 |  |  |
| 1924_10 | 11.67 | 6965_25 | 0.00 | 11603_98 | 3.33 |  |  | 1680_122 | 0.94 | 5367_154 | 12.26 | 8985_51 | 0.00 | 13111_107 | 4.72 |  |  |
| 1936_106 | 5.00 | 6967_124 | 1.67 | 11606_73 | 1.67 |  |  | 1684_40 | 7.55 | 5377_103 | 6.60 | 8987_103 | 1.89 | 13116_192 | 0.00 |  |  |
| 1939_85 | 0.00 | 6972_136 | 3.33 | 11611_10 | 1.67 |  |  | 1685_142 | 1.89 | 5382_5 | 0.94 | 8992_106 | 0.00 | 13119_46 | 2.83 |  |  |
| 1941_8 | 3.33 | 6981_95 | 0.00 | 11629_13 | 3.33 |  |  | 1688_93 | 1.89 | 5394_34 | 5.66 | 8995_110 | 10.38 | 13120_75 | 0.94 |  |  |
| 1942_168 | 8.33 | 6984_18 | 5.00 | 11633_110 | 1.67 |  |  | 1691_164 | 2.83 | 5400_65 | 6.60 | 8996_14 | 5.66 | 13128_19 | 0.00 |  |  |
| 1945_91 | 3.33 | 6990_142 | 1.67 | 11634_120 | 0.00 |  |  | 1692_31 | 11.32 | 5402_52 | 0.00 | 8998_130 | 0.00 | 13129_47 | 5.66 |  |  |
| 1951_157 | 1.67 | 6994_24 | 8.33 | 11636_124 | 0.00 |  |  | 1694_102 | 0.00 | 5407_27 | 4.72 | 9000_22 | 5.66 | 13133_131 | 6.60 |  |  |
| 1954_193 | 8.33 | 6995_152 | 0.00 | 11641_7 | 8.33 |  |  | 1697_92 | 0.00 | 5418_26 | 8.49 | 9003_150 | 1.89 | 13138_131 | 3.77 |  |  |
| 1960_15 | 10.00 | 6999_36 | 1.67 | 11643_61 | 1.67 |  |  | 1700_151 | 13.21 | 5424_122 | 5.66 | 9006_105 | 0.00 | 13144_78 | 4.72 |  |  |
| 1968_192 | 5.00 | 7000_56 | 5.00 | 11646_10 | 11.67 |  |  | 1701_165 | 7.55 | 5428_120 | 0.94 | 9008_10 | 0.00 | 13151_160 | 0.00 |  |  |
| 1976_101 | 0.00 | 7002_163 | 1.67 | 11657_149 | 0.00 |  |  | 1702_195 | 0.00 | 5429_53 | 0.00 | 9010_65 | 3.77 | 13152_7 | 0.00 |  |  |
| 1981_16 | 1.67 | 7012_98 | 1.67 | 11667_40 | 0.00 |  |  | 1706_23 | 0.00 | 5430_70 | 0.00 | 9013_22 | 9.43 | 13153_15 | 3.77 |  |  |
| 1983_110 | 0.00 | 7015_24 | 0.00 | 11669_27 | 3.33 |  |  | 1708_59 | 4.72 | 5436_50 | 0.00 | 9015_115 | 1.89 | 13159_101 | 4.72 |  |  |
| 1988_93 | 0.00 | 7016_163 | 0.00 | 11670_29 | 3.33 |  |  | 1713_13 | 0.94 | 5437_73 | 0.94 | 9016_22 | 7.55 | 13161_36 | 0.00 |  |  |
| 1990_50 | 3.33 | 7018_56 | 0.00 | 11686_120 | 1.67 |  |  | 1714_56 | 1.89 | 5442_44 | 7.55 | 9024_73 | 0.94 | 13162_133 | 0.94 |  |  |
| 1991_77 | 1.67 | 7019_59 | 1.67 | 11712_110 | 6.67 |  |  | 1719_64 | 0.00 | 5447_13 | 2.83 | 9033_83 | 0.94 | 13169_15 | 0.00 |  |  |
| 1993_36 | 0.00 | 7027_66 | 8.33 | 11717_80 | 0.00 |  |  | 1721_140 | 0.94 | 5450_10 | 0.00 | 9037_100 | 15.09 | 13170_22 | 0.00 |  |  |
| 1994_106 | 1.67 | 7028_16 | 6.67 | 11718_145 | 11.67 |  |  | 1725_90 | 1.89 | 5455_85 | 0.00 | 9040_127 | 4.72 | 13171_97 | 8.49 |  |  |
| 2002_146 | 5.00 | 7029_65 | 6.67 | 11738_7 | 3.33 |  |  | 1727_99 | 0.00 | 5468_117 | 0.94 | 9041_21 | 12.26 | 13178_5 | 0.00 |  |  |
| 2004_135 | 5.00 | 7033_97 | 6.67 | 11742_116 | 3.33 |  |  | 1730_84 | 5.66 | 5469_141 | 3.77 | 9043_41 | 0.00 | 13185_64 | 0.94 |  |  |
| 2006_72 | 1.67 | 7036_80 | 0.00 | 11768_147 | 1.67 |  |  | 1734_82 | 0.00 | 5478_92 | 0.94 | 9049_62 | 0.00 | 13188_109 | 0.00 |  |  |
| 2009_31 | 5.00 | 7037_105 | 3.33 | 11770_36 | 0.00 |  |  | 1736_120 | 0.00 | 5480_79 | 4.72 | 9058_81 | 1.89 | 13194_15 | 2.83 |  |  |
| 2014_97 | 5.00 | 7040_30 | 0.00 | 11773_72 | 1.67 |  |  | 1742_113 | 14.15 | 5484_63 | 1.89 | 9059_82 | 0.94 | 13195_25 | 0.00 |  |  |
| 2016_94 | 3.33 | 7043_58 | 1.67 | 11776_168 | 3.33 |  |  | 1744_84 | 4.72 | 5485_14 | 1.89 | 9061_95 | 0.00 | 13198_142 | 2.83 |  |  |
| 2019_7 | 0.00 | 7045_92 | 6.67 | 11783_135 | 1.67 |  |  | 1749_101 | 10.38 | 5489_94 | 0.00 | 9062_29 | 3.77 | 13202_73 | 4.72 |  |  |
| 2021_7 | 0.00 | 7047_56 | 0.00 | 11796_49 | 1.67 |  |  | 1755_110 | 0.94 | 5494_123 | 0.00 | 9065_115 | 0.00 | 13203_47 | 2.83 |  |  |
| 2023_46 | 10.00 | 7052_68 | 1.67 | 11798_62 | 1.67 |  |  | 1759_72 | 7.55 | 5496_55 | 0.94 | 9071_51 | 0.94 | 13204_97 | 0.94 |  |  |
| 2026_14 | 3.33 | 7055_26 | 5.00 | 11806_56 | 6.67 |  |  | 1760_9 | 0.94 | 5497_6 | 0.94 | 9072_126 | 11.32 | 13206_120 | 0.00 |  |  |
| 2028_58 | 0.00 | 7058_75 | 1.67 | 11819_135 | 13.33 |  |  | 1766_71 | 0.94 | 5498_122 | 0.94 | 9080_37 | 7.55 | 13221_92 | 0.94 |  |  |
| 2029_141 | 5.00 | 7062_180 | 0.00 | 11822_72 | 1.67 |  |  | 1767_47 | 2.83 | 5501_26 | 8.49 | 9083_132 | 3.77 | 13223_46 | 1.89 |  |  |
| 2031_38 | 1.67 | 7063_18 | 1.67 | 11832_40 | 3.33 |  |  | 1768_44 | 0.94 | 5504_81 | 3.77 | 9084_146 | 0.00 | 13224_89 | 0.00 |  |  |
| 2035_33 | 3.33 | 7070_50 | 10.00 | 11837_120 | 5.00 |  |  | 1776_68 | 0.00 | 5506_134 | 6.60 | 9091_69 | 0.00 | 13229_113 | 0.00 |  |  |
| 2036_9 | 0.00 | 7072_172 | 0.00 | 11839_114 | 1.67 |  |  | 1779_18 | 4.72 | 5510_51 | 13.21 | 9094_9 | 4.72 | 13248_46 | 12.26 |  |  |
| 2041_37 | 6.67 | 7077_111 | 0.00 | 11842_121 | 0.00 |  |  | 1780_59 | 1.89 | 5514_70 | 0.00 | 9095_54 | 0.00 | 13249_62 | 2.83 |  |  |
| 2045_105 | 1.67 | 7080_92 | 6.67 | 11845_121 | 1.67 |  |  | 1785_39 | 2.83 | 5520_20 | 3.77 | 9097_58 | 13.21 | 13254_76 | 8.49 |  |  |
| 2047_19 | 0.00 | 7090_75 | 0.00 | 11849_7 | 0.00 |  |  | 1786_158 | 3.77 | 5528_119 | 0.94 | 9108_125 | 0.00 | 13259_177 | 2.83 |  |  |
| 2051_39 | 0.00 | 7093_73 | 1.67 | 11855_82 | 0.00 |  |  | 1793_59 | 7.55 | 5529_100 | 0.00 | 9109_18 | 0.00 | 13265_103 | 0.94 |  |  |
| 2053_128 | 0.00 | 7096_163 | 1.67 | 11857_125 | 1.67 |  |  | 1794_8 | 6.60 | 5530_182 | 8.49 | 9116_76 | 0.94 | 13271_33 | 2.83 |  |  |
| 2056_73 | 0.00 | 7100_149 | 1.67 | 11859_77 | 5.00 |  |  | 1796_132 | 6.60 | 5533_91 | 2.83 | 9119_96 | 2.83 | 13281_193 | 4.72 |  |  |
| 2057_10 | 0.00 | 7103_67 | 1.67 | 11864_64 | 1.67 |  |  | 1797_28 | 5.66 | 5536_78 | 4.72 | 9121_117 | 1.89 | 13282_116 | 3.77 |  |  |
| 2061_70 | 5.00 | 7110_148 | 0.00 | 11865_30 | 10.00 |  |  | 1802_160 | 4.72 | 5537_47 | 7.55 | 9124_180 | 19.81 | 13284_20 | 0.00 |  |  |
| 2064_166 | 5.00 | 7112_78 | 0.00 | 11866_119 | 3.33 |  |  | 1806_92 | 3.77 | 5541_111 | 0.00 | 9131_46 | 0.94 | 13287_101 | 1.89 |  |  |
| 2065_61 | 1.67 | 7116_116 | 0.00 | 11867_90 | 1.67 |  |  | 1808_87 | 0.00 | 5546_51 | 11.32 | 9134_69 | 7.55 | 13292_87 | 4.72 |  |  |
| 2070_122 | 1.67 | 7124_132 | 0.00 | 11873_51 | 3.33 |  |  | 1809_38 | 2.83 | 5555_24 | 8.49 | 9136_5 | 2.83 | 13295_164 | 5.66 |  |  |
| 2078_190 | 6.67 | 7126_23 | 8.33 | 11882_150 | 16.67 |  |  | 1810_152 | 1.89 | 5556_90 | 1.89 | 9139_47 | 5.66 | 13296_152 | 4.72 |  |  |
| 2081_114 | 0.00 | 7127_6 | 1.67 | 11883_90 | 3.33 |  |  | 1813_59 | 0.00 | 5560_81 | 0.00 | 9147_10 | 0.94 | 13303_113 | 3.77 |  |  |
| 2082_185 | 1.67 | 7139_59 | 3.33 | 11886_194 | 18.33 |  |  | 1815_14 | 0.94 | 5563_91 | 0.00 | 9150_22 | 0.00 | 13308_34 | 15.09 |  |  |
| 2086_127 | 11.67 | 7142_100 | 1.67 | 11896_137 | 10.00 |  |  | 1817_11 | 0.00 | 5564_40 | 0.94 | 9151_139 | 0.00 | 13312_112 | 1.89 |  |  |
| 2087_70 | 0.00 | 7145_141 | 3.33 | 11899_31 | 0.00 |  |  | 1819_184 | 6.60 | 5566_126 | 10.38 | 9152_8 | 0.00 | 13320_93 | 5.66 |  |  |
| 2090_22 | 0.00 | 7148_112 | 0.00 | 11910_32 | 5.00 |  |  | 1822_97 | 2.83 | 5572_62 | 2.83 | 9154_44 | 0.94 | 13322_172 | 2.83 |  |  |
| 2097_139 | 6.67 | 7149_29 | 11.67 | 11921_112 | 0.00 |  |  | 1825_107 | 9.43 | 5577_32 | 0.94 | 9158_115 | 0.94 | 13323_193 | 4.72 |  |  |
| 2099_101 | 1.67 | 7150_117 | 1.67 | 11923_112 | 6.67 |  |  | 1827_54 | 0.00 | 5579_60 | 3.77 | 9163_100 | 0.00 | 13328_140 | 0.94 |  |  |
| 2111_102 | 0.00 | 7151_60 | 0.00 | 11927_135 | 1.67 |  |  | 1833_106 | 0.94 | 5583_75 | 6.60 | 9169_111 | 12.26 | 13329_177 | 5.66 |  |  |
| 2116_131 | 0.00 | 7160_27 | 6.67 | 11929_40 | 1.67 |  |  | 1834_136 | 2.83 | 5584_26 | 0.94 | 9174_168 | 4.72 | 13336_59 | 1.89 |  |  |
| 2117_26 | 1.67 | 7163_168 | 0.00 | 11944_149 | 3.33 |  |  | 1842_25 | 0.00 | 5587_33 | 11.32 | 9176_13 | 5.66 | 13337_99 | 2.83 |  |  |
| 2124_55 | 1.67 | 7169_80 | 0.00 | 11952_157 | 6.67 |  |  | 1846_75 | 9.43 | 5591_29 | 9.43 | 9177_59 | 4.72 | 13342_141 | 3.77 |  |  |
| 2127_113 | 6.67 | 7170_28 | 1.67 | 11968_190 | 0.00 |  |  | 1855_67 | 0.94 | 5595_90 | 1.89 | 9179_68 | 7.55 | 13346_163 | 5.66 |  |  |
| 2131_84 | 0.00 | 7171_71 | 0.00 | 11969_85 | 0.00 |  |  | 1857_153 | 10.38 | 5604_21 | 0.00 | 9180_16 | 0.94 | 13348_60 | 0.00 |  |  |
| 2132_154 | 1.67 | 7173_102 | 0.00 | 11975_21 | 0.00 |  |  | 1858_60 | 5.66 | 5606_23 | 0.94 | 9183_22 | 1.89 | 13350_27 | 1.89 |  |  |
| 2139_67 | 0.00 | 7175_27 | 0.00 | 11978_89 | 8.33 |  |  | 1861_38 | 9.43 | 5612_119 | 5.66 | 9189_127 | 15.09 | 13352_57 | 6.60 |  |  |
| 2142_168 | 6.67 | 7188_106 | 1.67 | 11998_76 | 1.67 |  |  | 1862_8 | 4.72 | 5613_149 | 0.94 | 9194_122 | 1.89 | 13353_44 | 5.66 |  |  |
| 2144_80 | 23.33 | 7190_79 | 0.00 | 12017_107 | 1.67 |  |  | 1863_100 | 1.89 | 5618_45 | 0.00 | 9200_11 | 2.83 | 13363_64 | 9.43 |  |  |
| 2145_85 | 0.00 | 7194_54 | 3.33 | 12030_9 | 8.33 |  |  | 1865_90 | 9.43 | 5619_155 | 0.94 | 9201_41 | 0.94 | 13380_25 | 4.72 |  |  |
| 2148_10 | 1.67 | 7199_139 | 1.67 | 12056_23 | 1.67 |  |  | 1868_129 | 0.00 | 5621_41 | 5.66 | 9202_107 | 0.00 | 13382_13 | 5.66 |  |  |
| 2151_17 | 0.00 | 7204_43 | 0.00 | 12061_166 | 5.00 |  |  | 1870_89 | 7.55 | 5622_6 | 0.00 | 9204_133 | 3.77 | 13384_46 | 4.72 |  |  |
| 2153_67 | 0.00 | 7206_144 | 0.00 | 12065_79 | 11.67 |  |  | 1875_49 | 6.60 | 5623_147 | 3.77 | 9207_158 | 0.94 | 13386_34 | 4.72 |  |  |
| 2159_71 | 1.67 | 7215_150 | 0.00 | 12073_154 | 3.33 |  |  | 1878_51 | 9.43 | 5625_72 | 15.09 | 9210_78 | 11.32 | 13387_98 | 4.72 |  |  |
| 2163_67 | 0.00 | 7218_73 | 1.67 | 12080_34 | 0.00 |  |  | 1881_56 | 0.00 | 5631_128 | 11.32 | 9212_122 | 0.94 | 13388_121 | 5.66 |  |  |
| 2169_116 | 0.00 | 7221_157 | 3.33 | 12082_122 | 1.67 |  |  | 1882_19 | 0.00 | 5632_76 | 14.15 | 9216_120 | 0.00 | 13389_135 | 0.94 |  |  |
| 2170_123 | 0.00 | 7224_11 | 0.00 | 12092_152 | 3.33 |  |  | 1883_70 | 5.66 | 5634_164 | 8.49 | 9219_113 | 0.94 | 13395_5 | 12.26 |  |  |
| 2172_51 | 5.00 | 7226_83 | 0.00 | 12098_49 | 3.33 |  |  | 1884_59 | 10.38 | 5635_35 | 0.94 | 9222_19 | 0.94 | 13405_61 | 3.77 |  |  |
| 2181_19 | 1.67 | 7227_137 | 0.00 | 12102_37 | 6.67 |  |  | 1886_116 | 13.21 | 5636_44 | 0.00 | 9224_64 | 10.38 | 13409_6 | 7.55 |  |  |
| 2188_63 | 1.67 | 7229_82 | 3.33 | 12116_36 | 1.67 |  |  | 1887_67 | 8.49 | 5642_112 | 0.00 | 9225_95 | 5.66 | 13418_97 | 1.89 |  |  |
| 2192_166 | 3.33 | 7231_43 | 1.67 | 12117_190 | 8.33 |  |  | 1888_149 | 13.21 | 5648_142 | 4.72 | 9226_57 | 4.72 | 13419_92 | 1.89 |  |  |
| 2194_38 | 1.67 | 7233_166 | 11.67 | 12123_23 | 0.00 |  |  | 1892_85 | 6.60 | 5652_25 | 0.94 | 9228_145 | 2.83 | 13422_46 | 0.00 |  |  |
| 2195_59 | 5.00 | 7234_56 | 1.67 | 12129_130 | 1.67 |  |  | 1898_25 | 1.89 | 5653_16 | 5.66 | 9237_66 | 0.00 | 13430_8 | 12.26 |  |  |
| 2197_59 | 0.00 | 7236_71 | 1.67 | 12131_40 | 0.00 |  |  | 1900_78 | 2.83 | 5654_13 | 0.00 | 9241_76 | 2.83 | 13432_55 | 0.00 |  |  |
| 2198_185 | 6.67 | 7241_17 | 8.33 | 12132_154 | 1.67 |  |  | 1901_140 | 0.00 | 5655_87 | 2.83 | 9242_26 | 4.72 | 13435_75 | 0.00 |  |  |
| 2199_26 | 13.33 | 7243_107 | 0.00 | 12140_17 | 3.33 |  |  | 1902_103 | 0.94 | 5658_131 | 1.89 | 9253_48 | 0.94 | 13437_39 | 8.49 |  |  |
| 2201_166 | 15.00 | 7251_167 | 6.67 | 12144_20 | 1.67 |  |  | 1907_102 | 0.00 | 5664_55 | 4.72 | 9264_103 | 0.00 | 13442_150 | 13.21 |  |  |
| 2205_109 | 1.67 | 7253_115 | 0.00 | 12151_11 | 0.00 |  |  | 1908_81 | 0.00 | 5667_87 | 9.43 | 9265_15 | 16.98 | 13444_44 | 5.66 |  |  |
| 2206_37 | 10.00 | 7255_145 | 3.33 | 12162_35 | 3.33 |  |  | 1909_38 | 0.00 | 5669_43 | 1.89 | 9266_38 | 0.94 | 13452_110 | 6.60 |  |  |
| 2209_99 | 0.00 | 7260_32 | 1.67 | 12163_12 | 5.00 |  |  | 1913_131 | 0.94 | 5670_128 | 11.32 | 9267_95 | 9.43 | 13453_137 | 0.94 |  |  |
| 2211_17 | 0.00 | 7261_60 | 1.67 | 12164_82 | 0.00 |  |  | 1924_10 | 12.26 | 5672_122 | 12.26 | 9269_105 | 3.77 | 13465_47 | 5.66 |  |  |
| 2214_22 | 3.33 | 7265_58 | 0.00 | 12173_127 | 3.33 |  |  | 1934_122 | 0.00 | 5676_77 | 4.72 | 9276_162 | 7.55 | 13478_63 | 6.60 |  |  |
| 2217_62 | 11.67 | 7271_138 | 0.00 | 12180_99 | 3.33 |  |  | 1936_106 | 6.60 | 5681_106 | 9.43 | 9277_11 | 2.83 | 13479_18 | 10.38 |  |  |
| 2219_41 | 1.67 | 7275_109 | 6.67 | 12181_86 | 3.33 |  |  | 1937_81 | 9.43 | 5686_74 | 1.89 | 9278_17 | 10.38 | 13481_93 | 11.32 |  |  |
| 2223_84 | 8.33 | 7281_103 | 3.33 | 12183_11 | 5.00 |  |  | 1939_85 | 0.00 | 5688_72 | 4.72 | 9279_112 | 0.00 | 13490_123 | 5.66 |  |  |
| 2224_56 | 16.67 | 7292_128 | 0.00 | 12200_58 | 3.33 |  |  | 1941_8 | 1.89 | 5689_93 | 8.49 | 9288_64 | 1.89 | 13495_37 | 0.00 |  |  |
| 2232_14 | 3.33 | 7293_88 | 0.00 | 12204_107 | 10.00 |  |  | 1942_168 | 7.55 | 5700_107 | 5.66 | 9289_80 | 5.66 | 13496_141 | 2.83 |  |  |
| 2233_37 | 0.00 | 7294_192 | 1.67 | 12207_142 | 6.67 |  |  | 1945_91 | 3.77 | 5703_24 | 0.94 | 9290_62 | 0.00 | 13502_127 | 1.89 |  |  |
| 2236_53 | 1.67 | 7297_60 | 0.00 | 12209_125 | 11.67 |  |  | 1951_157 | 0.94 | 5708_149 | 0.94 | 9291_68 | 4.72 | 13506_10 | 4.72 |  |  |
| 2245_80 | 10.00 | 7302_47 | 1.67 | 12214_69 | 0.00 |  |  | 1954_193 | 7.55 | 5709_88 | 7.55 | 9293_113 | 15.09 | 13512_5 | 6.60 |  |  |
| 2247_74 | 1.67 | 7309_21 | 1.67 | 12230_75 | 0.00 |  |  | 1960_15 | 12.26 | 5710_8 | 10.38 | 9296_136 | 0.00 | 13513_44 | 1.89 |  |  |
| 2253_6 | 1.67 | 7310_162 | 0.00 | 12232_33 | 0.00 |  |  | 1968_192 | 7.55 | 5713_18 | 10.38 | 9298_8 | 6.60 | 13516_54 | 5.66 |  |  |
| 2254_114 | 0.00 | 7318_81 | 1.67 | 12239_121 | 5.00 |  |  | 1969_117 | 6.60 | 5714_152 | 11.32 | 9300_93 | 0.94 | 13520_161 | 0.94 |  |  |
| 2257_123 | 3.33 | 7320_28 | 0.00 | 12242_188 | 8.33 |  |  | 1976_101 | 0.00 | 5718_103 | 0.00 | 9301_125 | 0.00 | 13534_11 | 11.32 |  |  |
| 2259_41 | 13.33 | 7322_116 | 11.67 | 12246_74 | 1.67 |  |  | 1981_16 | 0.94 | 5719_169 | 0.00 | 9305_25 | 7.55 | 13537_18 | 5.66 |  |  |
| 2260_68 | 0.00 | 7325_50 | 0.00 | 12247_38 | 1.67 |  |  | 1983_110 | 0.00 | 5730_5 | 3.77 | 9308_95 | 0.00 | 13541_36 | 9.43 |  |  |
| 2263_181 | 6.67 | 7331_32 | 5.00 | 12251_109 | 1.67 |  |  | 1988_93 | 11.32 | 5739_104 | 2.83 | 9309_170 | 9.43 | 13549_7 | 1.89 |  |  |
| 2264_86 | 11.67 | 7338_157 | 1.67 | 12257_112 | 6.67 |  |  | 1990_50 | 8.49 | 5742_88 | 7.55 | 9310_18 | 12.26 | 13561_129 | 10.38 |  |  |
| 2266_33 | 6.67 | 7342_77 | 1.67 | 12258_33 | 5.00 |  |  | 1991_77 | 1.89 | 5748_24 | 1.89 | 9319_148 | 0.00 | 13563_82 | 0.00 |  |  |
| 2267_62 | 3.33 | 7343_79 | 5.00 | 12272_79 | 1.67 |  |  | 1993_36 | 0.00 | 5750_57 | 3.77 | 9324_62 | 3.77 | 13564_110 | 0.00 |  |  |
| 2270_83 | 0.00 | 7344_10 | 3.33 | 12273_194 | 6.67 |  |  | 1994_106 | 0.94 | 5754_46 | 3.77 | 9325_109 | 0.94 | 13573_161 | 1.89 |  |  |
| 2272_128 | 0.00 | 7345_124 | 1.67 | 12276_128 | 1.67 |  |  | 1996_129 | 0.00 | 5757_144 | 0.94 | 9326_125 | 2.83 | 13581_108 | 6.60 |  |  |
| 2274_80 | 0.00 | 7351_52 | 0.00 | 12278_87 | 1.67 |  |  | 2002_146 | 8.49 | 5758_111 | 0.00 | 9328_128 | 0.00 | 13584_100 | 7.55 |  |  |
| 2275_79 | 3.33 | 7356_75 | 10.00 | 12287_198 | 10.00 |  |  | 2004_135 | 4.72 | 5762_111 | 0.00 | 9331_59 | 0.00 | 13591_10 | 10.38 |  |  |
| 2277_126 | 0.00 | 7359_9 | 1.67 | 12294_56 | 5.00 |  |  | 2006_72 | 2.83 | 5770_105 | 7.55 | 9336_56 | 0.00 | 13592_32 | 10.38 |  |  |
| 2279_167 | 1.67 | 7360_143 | 1.67 | 12295_31 | 6.67 |  |  | 2009_31 | 4.72 | 5772_55 | 8.49 | 9338_14 | 6.60 | 13593_86 | 1.89 |  |  |
| 2281_78 | 5.00 | 7365_41 | 5.00 | 12297_175 | 8.33 |  |  | 2014_97 | 4.72 | 5777_21 | 3.77 | 9340_172 | 1.89 | 13596_41 | 0.94 |  |  |
| 2286_15 | 5.00 | 7366_135 | 0.00 | 12300_5 | 1.67 |  |  | 2016_94 | 2.83 | 5780_27 | 7.55 | 9342_46 | 19.81 | 13601_103 | 5.66 |  |  |
| 2295_61 | 1.67 | 7370_9 | 0.00 | 12303_64 | 18.33 |  |  | 2019_7 | 0.94 | 5788_69 | 0.00 | 9352_37 | 5.66 | 13602_151 | 10.38 |  |  |
| 2302_152 | 0.00 | 7372_79 | 0.00 | 12312_124 | 0.00 |  |  | 2021_7 | 0.00 | 5789_21 | 8.49 | 9357_99 | 3.77 | 13607_10 | 0.00 |  |  |
| 2303_14 | 3.33 | 7379_67 | 15.00 | 12325_147 | 0.00 |  |  | 2023_46 | 12.26 | 5790_87 | 6.60 | 9359_28 | 3.77 | 13608_63 | 0.00 |  |  |
| 2316_100 | 0.00 | 7384_33 | 0.00 | 12333_112 | 1.67 |  |  | 2026_14 | 2.83 | 5795_46 | 0.94 | 9361_75 | 0.00 | 13612_115 | 1.89 |  |  |
| 2319_79 | 1.67 | 7385_145 | 5.00 | 12346_92 | 13.33 |  |  | 2028_58 | 0.00 | 5796_88 | 8.49 | 9362_121 | 5.66 | 13618_60 | 4.72 |  |  |
| 2322_61 | 5.00 | 7386_31 | 0.00 | 12352_36 | 1.67 |  |  | 2029_141 | 3.77 | 5798_110 | 0.94 | 9363_145 | 8.49 | 13626_95 | 0.94 |  |  |
| 2323_102 | 1.67 | 7390_14 | 1.67 | 12355_48 | 3.33 |  |  | 2031_38 | 1.89 | 5802_79 | 0.94 | 9365_75 | 0.94 | 13627_85 | 3.77 |  |  |
| 2328_76 | 1.67 | 7396_44 | 0.00 | 12365_121 | 5.00 |  |  | 2035_33 | 1.89 | 5811_30 | 0.94 | 9372_9 | 0.94 | 13630_52 | 0.94 |  |  |
| 2331_47 | 1.67 | 7397_94 | 10.00 | 12369_165 | 6.67 |  |  | 2036_9 | 5.66 | 5815_101 | 0.00 | 9377_15 | 0.00 | 13635_78 | 3.77 |  |  |
| 2338_109 | 3.33 | 7398_68 | 0.00 | 12372_42 | 3.33 |  |  | 2038_97 | 1.89 | 5819_104 | 1.89 | 9382_23 | 0.94 | 13637_154 | 11.32 |  |  |
| 2341_150 | 5.00 | 7403_58 | 1.67 | 12377_121 | 0.00 |  |  | 2041_37 | 5.66 | 5826_161 | 7.55 | 9383_51 | 8.49 | 13646_41 | 4.72 |  |  |
| 2342_138 | 1.67 | 7406_107 | 3.33 | 12378_189 | 6.67 |  |  | 2045_105 | 2.83 | 5830_40 | 0.94 | 9392_114 | 1.89 | 13650_143 | 0.00 |  |  |
| 2347_189 | 8.33 | 7407_15 | 0.00 | 12382_83 | 5.00 |  |  | 2047_19 | 0.00 | 5833_11 | 10.38 | 9400_97 | 11.32 | 13653_135 | 1.89 |  |  |
| 2360_38 | 3.33 | 7408_28 | 0.00 | 12389_49 | 5.00 |  |  | 2051_39 | 3.77 | 5841_70 | 6.60 | 9404_177 | 6.60 | 13673_176 | 5.66 |  |  |
| 2364_126 | 11.67 | 7412_111 | 3.33 | 12394_93 | 1.67 |  |  | 2053_128 | 6.60 | 5842_121 | 0.94 | 9405_77 | 4.72 | 13677_26 | 11.32 |  |  |
| 2368_61 | 5.00 | 7413_65 | 1.67 | 12410_98 | 8.33 |  |  | 2056_73 | 0.00 | 5843_65 | 0.94 | 9409_74 | 3.77 | 13680_22 | 1.89 |  |  |
| 2377_137 | 8.33 | 7414_102 | 18.33 | 12411_146 | 6.67 |  |  | 2057_10 | 0.94 | 5844_37 | 2.83 | 9410_7 | 9.43 | 13681_100 | 0.94 |  |  |
| 2378_38 | 1.67 | 7428_16 | 1.67 | 12412_57 | 0.00 |  |  | 2061_70 | 4.72 | 5848_48 | 0.00 | 9414_57 | 7.55 | 13683_99 | 0.00 |  |  |
| 2380_100 | 0.00 | 7434_184 | 0.00 | 12415_14 | 0.00 |  |  | 2064_166 | 9.43 | 5858_61 | 2.83 | 9418_51 | 0.94 | 13684_64 | 9.43 |  |  |
| 2382_90 | 0.00 | 7437_79 | 0.00 | 12418_84 | 5.00 |  |  | 2065_61 | 0.94 | 5860_30 | 5.66 | 9419_180 | 9.43 | 13690_18 | 0.00 |  |  |
| 2385_14 | 0.00 | 7439_54 | 6.67 | 12420_58 | 10.00 |  |  | 2070_122 | 0.94 | 5866_98 | 0.00 | 9422_114 | 3.77 | 13696_48 | 2.83 |  |  |
| 2388_38 | 8.33 | 7441_32 | 0.00 | 12424_106 | 15.00 |  |  | 2072_49 | 8.49 | 5871_113 | 5.66 | 9425_7 | 4.72 | 13700_130 | 9.43 |  |  |
| 2400_156 | 1.67 | 7452_130 | 8.33 | 12428_169 | 1.67 |  |  | 2077_139 | 9.43 | 5873_90 | 0.00 | 9427_57 | 10.38 | 13702_109 | 4.72 |  |  |
| 2401_35 | 1.67 | 7457_7 | 8.33 | 12429_130 | 10.00 |  |  | 2078_190 | 4.72 | 5874_33 | 2.83 | 9430_10 | 0.00 | 13705_33 | 0.00 |  |  |
| 2402_68 | 0.00 | 7462_103 | 5.00 | 12430_85 | 8.33 |  |  | 2081_114 | 5.66 | 5880_116 | 3.77 | 9438_88 | 9.43 | 13708_14 | 0.00 |  |  |
| 2409_139 | 3.33 | 7465_45 | 8.33 | 12431_87 | 5.00 |  |  | 2082_185 | 0.94 | 5886_85 | 0.00 | 9442_86 | 3.77 | 13713_140 | 4.72 |  |  |
| 2416_46 | 0.00 | 7473_36 | 0.00 | 12436_110 | 0.00 |  |  | 2086_127 | 16.98 | 5891_9 | 2.83 | 9453_24 | 1.89 | 13714_129 | 1.89 |  |  |
| 2418_164 | 1.67 | 7474_165 | 5.00 | 12437_58 | 5.00 |  |  | 2087_70 | 0.00 | 5898_90 | 6.60 | 9456_50 | 2.83 | 13715_55 | 4.72 |  |  |
| 2422_78 | 0.00 | 7476_160 | 10.00 | 12438_72 | 3.33 |  |  | 2090_22 | 0.00 | 5899_33 | 2.83 | 9460_19 | 0.00 | 13718_116 | 6.60 |  |  |
| 2424_168 | 3.33 | 7478_72 | 15.00 | 12447_82 | 0.00 |  |  | 2097_139 | 4.72 | 5900_86 | 1.89 | 9461_41 | 1.89 | 13722_92 | 11.32 |  |  |
| 2425_176 | 13.33 | 7479_128 | 3.33 | 12461_69 | 1.67 |  |  | 2099_101 | 0.94 | 5901_48 | 0.94 | 9462_124 | 5.66 | 13726_21 | 10.38 |  |  |
| 2428_109 | 3.33 | 7487_41 | 0.00 | 12473_118 | 8.33 |  |  | 2111_102 | 0.94 | 5903_67 | 7.55 | 9470_146 | 2.83 | 13730_104 | 7.55 |  |  |
| 2429_30 | 0.00 | 7496_43 | 1.67 | 12475_107 | 1.67 |  |  | 2116_131 | 0.00 | 5904_72 | 11.32 | 9471_8 | 1.89 | 13735_89 | 1.89 |  |  |
| 2439_11 | 18.33 | 7500_159 | 8.33 | 12493_104 | 1.67 |  |  | 2117_26 | 1.89 | 5906_131 | 5.66 | 9472_35 | 7.55 | 13736_54 | 4.72 |  |  |
| 2445_74 | 0.00 | 7505_23 | 3.33 | 12504_81 | 11.67 |  |  | 2119_27 | 2.83 | 5907_54 | 1.89 | 9473_57 | 4.72 | 13737_84 | 5.66 |  |  |
| 2449_138 | 1.67 | 7506_139 | 0.00 | 12508_49 | 1.67 |  |  | 2124_55 | 0.94 | 5908_149 | 9.43 | 9476_10 | 0.94 | 13740_106 | 0.94 |  |  |
| 2453_61 | 15.00 | 7508_25 | 0.00 | 12515_84 | 6.67 |  |  | 2125_81 | 0.00 | 5911_62 | 0.94 | 9479_66 | 2.83 | 13745_110 | 4.72 |  |  |
| 2455_89 | 0.00 | 7512_131 | 1.67 | 12521_75 | 0.00 |  |  | 2127_113 | 8.49 | 5913_122 | 1.89 | 9481_115 | 1.89 | 13746_36 | 1.89 |  |  |
| 2458_12 | 3.33 | 7514_49 | 3.33 | 12525_104 | 0.00 |  |  | 2131_84 | 0.00 | 5923_62 | 6.60 | 9484_54 | 9.43 | 13748_73 | 0.00 |  |  |
| 2459_8 | 0.00 | 7515_23 | 3.33 | 12528_93 | 5.00 |  |  | 2132_154 | 0.94 | 5927_149 | 1.89 | 9489_165 | 1.89 | 13756_106 | 5.66 |  |  |
| 2465_104 | 3.33 | 7519_58 | 0.00 | 12538_71 | 1.67 |  |  | 2138_115 | 3.77 | 5930_142 | 8.49 | 9495_31 | 5.66 | 13757_54 | 10.38 |  |  |
| 2466_30 | 1.67 | 7524_87 | 1.67 | 12541_84 | 0.00 |  |  | 2139_67 | 0.94 | 5931_24 | 0.00 | 9496_43 | 0.94 | 13770_82 | 6.60 |  |  |
| 2470_41 | 0.00 | 7526_134 | 3.33 | 12544_35 | 6.67 |  |  | 2142_168 | 9.43 | 5932_91 | 13.21 | 9498_57 | 10.38 | 13784_202 | 6.60 |  |  |
| 2472_85 | 1.67 | 7528_145 | 0.00 | 12547_89 | 0.00 |  |  | 2144_80 | 14.15 | 5936_132 | 10.38 | 9501_107 | 0.94 | 13785_26 | 0.94 |  |  |
| 2475_132 | 11.67 | 7530_158 | 5.00 | 12553_24 | 0.00 |  |  | 2145_85 | 0.00 | 5938_80 | 1.89 | 9505_126 | 7.55 | 13786_27 | 4.72 |  |  |
| 2476_32 | 0.00 | 7532_107 | 0.00 | 12557_35 | 1.67 |  |  | 2148_10 | 2.83 | 5941_110 | 2.83 | 9506_55 | 5.66 | 13788_90 | 7.55 |  |  |
| 2477_47 | 13.33 | 7533_49 | 0.00 | 12568_61 | 1.67 |  |  | 2151_17 | 0.00 | 5944_64 | 7.55 | 9507_23 | 1.89 | 13789_71 | 3.77 |  |  |
| 2479_143 | 3.33 | 7545_137 | 5.00 | 12575_245 | 10.00 |  |  | 2153_67 | 0.00 | 5948_61 | 0.94 | 9508_133 | 0.94 | 13793_10 | 9.43 |  |  |
| 2482_56 | 0.00 | 7546_48 | 1.67 | 12577_89 | 6.67 |  |  | 2159_71 | 0.94 | 5949_88 | 4.72 | 9509_105 | 0.00 | 13797_153 | 1.89 |  |  |
| 2483_85 | 1.67 | 7553_139 | 0.00 | 12578_15 | 8.33 |  |  | 2162_108 | 4.72 | 5970_21 | 12.26 | 9510_87 | 4.72 | 13801_163 | 1.89 |  |  |
| 2485_46 | 5.00 | 7554_91 | 1.67 | 12580_13 | 15.00 |  |  | 2163_67 | 0.94 | 5973_74 | 4.72 | 9511_68 | 3.77 | 13802_42 | 0.00 |  |  |
| 2486_137 | 0.00 | 7557_62 | 15.00 | 12581_26 | 5.00 |  |  | 2169_116 | 0.00 | 5983_44 | 12.26 | 9512_126 | 2.83 | 13812_21 | 1.89 |  |  |
| 2487_106 | 0.00 | 7560_59 | 0.00 | 12584_29 | 0.00 |  |  | 2170_123 | 3.77 | 5986_86 | 13.21 | 9517_30 | 2.83 | 13815_91 | 6.60 |  |  |
| 2488_113 | 0.00 | 7565_121 | 1.67 | 12597_21 | 8.33 |  |  | 2172_51 | 7.55 | 5988_66 | 1.89 | 9519_75 | 0.00 | 13817_125 | 8.49 |  |  |
| 2490_59 | 8.33 | 7572_174 | 10.00 | 12601_48 | 6.67 |  |  | 2181_19 | 2.83 | 5990_17 | 0.00 | 9525_45 | 0.00 | 13823_83 | 1.89 |  |  |
| 2491_26 | 0.00 | 7573_13 | 8.33 | 12610_157 | 0.00 |  |  | 2188_63 | 3.77 | 5991_30 | 10.38 | 9526_105 | 2.83 | 13826_94 | 0.94 |  |  |
| 2493_20 | 0.00 | 7577_116 | 1.67 | 12611_16 | 8.33 |  |  | 2192_166 | 3.77 | 5992_151 | 5.66 | 9531_53 | 0.94 | 13828_111 | 4.72 |  |  |
| 2494_37 | 10.00 | 7581_144 | 3.33 | 12612_31 | 0.00 |  |  | 2194_38 | 2.83 | 5994_56 | 0.94 | 9536_95 | 0.94 | 13835_131 | 0.00 |  |  |
| 2502_20 | 11.67 | 7582_119 | 1.67 | 12617_19 | 6.67 |  |  | 2195_59 | 4.72 | 5995_67 | 1.89 | 9539_142 | 0.00 | 13837_110 | 12.26 |  |  |
| 2508_89 | 6.67 | 7583_127 | 3.33 | 12618_175 | 1.67 |  |  | 2197_59 | 0.00 | 5998_33 | 0.00 | 9541_11 | 1.89 | 13842_149 | 2.83 |  |  |
| 2515_47 | 0.00 | 7584_148 | 1.67 | 12624_172 | 6.67 |  |  | 2198_185 | 6.60 | 5999_26 | 3.77 | 9542_131 | 0.94 | 13849_8 | 2.83 |  |  |
| 2518_102 | 8.33 | 7589_105 | 1.67 | 12644_171 | 6.67 |  |  | 2199_26 | 10.38 | 6003_18 | 1.89 | 9544_7 | 7.55 | 13853_136 | 8.49 |  |  |
| 2523_97 | 0.00 | 7594_176 | 8.33 | 12650_60 | 8.33 |  |  | 2201_166 | 8.49 | 6010_162 | 0.94 | 9547_112 | 8.49 | 13855_87 | 0.00 |  |  |
| 2524_44 | 0.00 | 7596_50 | 0.00 | 12654_90 | 1.67 |  |  | 2205_109 | 1.89 | 6011_103 | 0.00 | 9550_163 | 10.38 | 13857_153 | 4.72 |  |  |
| 2525_64 | 6.67 | 7597_139 | 0.00 | 12658_185 | 3.33 |  |  | 2206_37 | 11.32 | 6012_37 | 0.00 | 9552_55 | 0.94 | 13871_63 | 4.72 |  |  |
| 2527_184 | 1.67 | 7599_7 | 1.67 | 12659_102 | 3.33 |  |  | 2207_54 | 0.00 | 6014_47 | 6.60 | 9555_169 | 0.94 | 13872_116 | 0.94 |  |  |
| 2534_107 | 1.67 | 7600_6 | 15.00 | 12670_166 | 6.67 |  |  | 2209_99 | 0.00 | 6017_90 | 3.77 | 9567_71 | 0.00 | 13877_114 | 0.94 |  |  |
| 2537_109 | 1.67 | 7603_81 | 0.00 | 12673_29 | 1.67 |  |  | 2211_17 | 0.00 | 6019_14 | 0.00 | 9568_95 | 3.77 | 13881_117 | 0.94 |  |  |
| 2542_58 | 1.67 | 7605_234 | 3.33 | 12674_91 | 0.00 |  |  | 2214_22 | 1.89 | 6021_6 | 4.72 | 9573_83 | 2.83 | 13884_96 | 1.89 |  |  |
| 2551_98 | 3.33 | 7607_41 | 0.00 | 12678_126 | 0.00 |  |  | 2217_62 | 6.60 | 6023_62 | 5.66 | 9576_12 | 10.38 | 13888_52 | 1.89 |  |  |
| 2553_63 | 0.00 | 7608_36 | 10.00 | 12680_32 | 25.00 |  |  | 2219_41 | 1.89 | 6025_33 | 3.77 | 9578_100 | 0.94 | 13892_42 | 9.43 |  |  |
| 2558_114 | 6.67 | 7609_159 | 1.67 | 12685_106 | 5.00 |  |  | 2223_84 | 4.72 | 6032_70 | 7.55 | 9581_138 | 8.49 | 13893_118 | 2.83 |  |  |
| 2560_108 | 1.67 | 7611_106 | 0.00 | 12692_104 | 0.00 |  |  | 2224_56 | 13.21 | 6033_57 | 9.43 | 9584_43 | 0.00 | 13918_19 | 0.00 |  |  |
| 2561_96 | 5.00 | 7615_190 | 1.67 | 12695_9 | 11.67 |  |  | 2231_84 | 2.83 | 6038_9 | 0.00 | 9585_28 | 6.60 | 13922_13 | 6.60 |  |  |
| 2563_18 | 1.67 | 7618_90 | 15.00 | 12700_84 | 1.67 |  |  | 2232_14 | 6.60 | 6039_68 | 0.00 | 9597_31 | 2.83 | 13930_59 | 1.89 |  |  |
| 2564_59 | 10.00 | 7622_158 | 0.00 | 12703_86 | 0.00 |  |  | 2233_37 | 0.94 | 6042_75 | 4.72 | 9598_99 | 0.00 | 13933_168 | 9.43 |  |  |
| 2569_132 | 3.33 | 7624_52 | 3.33 | 12721_13 | 10.00 |  |  | 2236_53 | 1.89 | 6043_25 | 0.00 | 9599_23 | 8.49 | 13936_43 | 3.77 |  |  |
| 2570_179 | 8.33 | 7625_51 | 0.00 | 12722_62 | 1.67 |  |  | 2237_68 | 2.83 | 6045_47 | 6.60 | 9600_66 | 0.00 | 13941_92 | 11.32 |  |  |
| 2572_47 | 1.67 | 7631_28 | 1.67 | 12727_102 | 3.33 |  |  | 2243_7 | 0.00 | 6046_46 | 7.55 | 9604_107 | 3.77 | 13944_48 | 0.00 |  |  |
| 2573_99 | 0.00 | 7633_88 | 1.67 | 12731_57 | 6.67 |  |  | 2245_80 | 8.49 | 6047_87 | 6.60 | 9609_70 | 3.77 | 13955_13 | 2.83 |  |  |
| 2574_129 | 0.00 | 7635_108 | 15.00 | 12732_123 | 5.00 |  |  | 2247_74 | 2.83 | 6049_74 | 5.66 | 9611_59 | 1.89 | 13959_111 | 0.00 |  |  |
| 2575_76 | 11.67 | 7636_68 | 10.00 | 12743_93 | 0.00 |  |  | 2253_6 | 0.94 | 6051_107 | 2.83 | 9615_63 | 8.49 | 13960_152 | 2.83 |  |  |
| 2577_125 | 0.00 | 7638_86 | 11.67 | 12747_59 | 0.00 |  |  | 2254_114 | 0.00 | 6055_168 | 0.94 | 9617_73 | 5.66 | 13965_34 | 14.15 |  |  |
| 2578_156 | 0.00 | 7642_24 | 1.67 | 12751_134 | 1.67 |  |  | 2257_123 | 3.77 | 6056_142 | 0.00 | 9619_71 | 5.66 | 13970_71 | 12.26 |  |  |
| 2580_81 | 0.00 | 7649_143 | 3.33 | 12757_136 | 1.67 |  |  | 2259_41 | 10.38 | 6058_83 | 5.66 | 9621_66 | 2.83 | 13973_50 | 0.00 |  |  |
| 2581_39 | 6.67 | 7650_155 | 5.00 | 12763_69 | 3.33 |  |  | 2260_68 | 0.00 | 6062_6 | 0.94 | 9623_61 | 2.83 | 13981_184 | 5.66 |  |  |
| 2584_122 | 1.67 | 7651_28 | 8.33 | 12772_166 | 16.67 |  |  | 2263_181 | 5.66 | 6069_131 | 0.94 | 9624_195 | 7.55 | 13988_14 | 11.32 |  |  |
| 2585_66 | 1.67 | 7653_81 | 1.67 | 12774_138 | 1.67 |  |  | 2264_86 | 13.21 | 6075_39 | 2.83 | 9625_14 | 0.00 | 13992_10 | 8.49 |  |  |
| 2590_73 | 6.67 | 7654_180 | 5.00 | 12777_108 | 0.00 |  |  | 2266_33 | 6.60 | 6081_17 | 0.00 | 9633_95 | 7.55 | 13993_27 | 0.94 |  |  |
| 2593_134 | 3.33 | 7660_123 | 3.33 | 12784_44 | 3.33 |  |  | 2267_62 | 4.72 | 6084_92 | 2.83 | 9635_100 | 11.32 | 13995_19 | 0.94 |  |  |
| 2596_169 | 10.00 | 7663_17 | 3.33 | 12793_155 | 3.33 |  |  | 2270_83 | 0.00 | 6085_187 | 3.77 | 9636_88 | 0.94 | 14003_10 | 8.49 |  |  |
| 2599_17 | 0.00 | 7664_130 | 6.67 | 12798_109 | 6.67 |  |  | 2272_128 | 0.00 | 6087_72 | 7.55 | 9640_59 | 3.77 | 14018_97 | 9.43 |  |  |
| 2604_98 | 1.67 | 7666_156 | 11.67 | 12811_156 | 0.00 |  |  | 2274_80 | 0.94 | 6089_83 | 10.38 | 9641_106 | 0.00 | 14023_100 | 4.72 |  |  |
| 2608_85 | 0.00 | 7668_87 | 1.67 | 12812_127 | 1.67 |  |  | 2275_79 | 3.77 | 6094_83 | 9.43 | 9645_45 | 7.55 | 14030_65 | 7.55 |  |  |
| 2612_38 | 0.00 | 7669_54 | 6.67 | 12815_72 | 15.00 |  |  | 2277_126 | 0.00 | 6096_28 | 14.15 | 9647_65 | 1.89 | 14033_129 | 2.83 |  |  |
| 2616_23 | 1.67 | 7672_139 | 6.67 | 12819_132 | 5.00 |  |  | 2279_167 | 0.94 | 6097_106 | 0.00 | 9648_22 | 2.83 | 14042_34 | 6.60 |  |  |
| 2621_18 | 1.67 | 7674_69 | 1.67 | 12833_175 | 1.67 |  |  | 2281_78 | 8.49 | 6102_93 | 3.77 | 9651_136 | 5.66 | 14044_134 | 13.21 |  |  |
| 2623_78 | 0.00 | 7682_89 | 1.67 | 12835_56 | 11.67 |  |  | 2283_145 | 6.60 | 6103_98 | 13.21 | 9652_109 | 0.00 | 14046_84 | 6.60 |  |  |
| 2626_56 | 0.00 | 7685_23 | 8.33 | 12846_60 | 5.00 |  |  | 2286_15 | 4.72 | 6104_129 | 5.66 | 9654_75 | 2.83 | 14054_146 | 13.21 |  |  |
| 2631_90 | 0.00 | 7692_40 | 1.67 | 12852_169 | 0.00 |  |  | 2289_32 | 6.60 | 6108_108 | 3.77 | 9656_80 | 0.94 | 14059_89 | 14.15 |  |  |
| 2641_88 | 1.67 | 7693_152 | 1.67 | 12856_59 | 1.67 |  |  | 2295_61 | 4.72 | 6112_91 | 1.89 | 9662_11 | 0.00 | 14060_104 | 5.66 |  |  |
| 2645_53 | 0.00 | 7701_39 | 10.00 | 12861_133 | 5.00 |  |  | 2296_34 | 5.66 | 6113_46 | 1.89 | 9664_165 | 2.83 | 14061_193 | 11.32 |  |  |
| 2664_15 | 0.00 | 7705_83 | 1.67 | 12863_147 | 5.00 |  |  | 2302_152 | 0.00 | 6117_18 | 1.89 | 9668_9 | 5.66 | 14066_100 | 9.43 |  |  |
| 2666_15 | 10.00 | 7709_148 | 0.00 | 12873_150 | 0.00 |  |  | 2303_14 | 6.60 | 6118_33 | 1.89 | 9669_10 | 17.92 | 14068_34 | 0.00 |  |  |
| 2678_50 | 3.33 | 7710_40 | 1.67 | 12885_65 | 1.67 |  |  | 2311_157 | 2.83 | 6120_143 | 0.00 | 9672_158 | 0.00 | 14076_143 | 0.00 |  |  |
| 2680_58 | 0.00 | 7722_106 | 5.00 | 12889_60 | 0.00 |  |  | 2316_100 | 0.00 | 6125_18 | 3.77 | 9676_100 | 9.43 | 14077_57 | 6.60 |  |  |
| 2681_73 | 1.67 | 7724_83 | 3.33 | 12892_64 | 3.33 |  |  | 2319_79 | 0.94 | 6127_41 | 5.66 | 9679_104 | 0.94 | 14080_113 | 0.00 |  |  |
| 2683_90 | 10.00 | 7726_85 | 11.67 | 12900_121 | 0.00 |  |  | 2322_61 | 2.83 | 6128_86 | 4.72 | 9682_84 | 0.94 | 14082_24 | 3.77 |  |  |
| 2684_103 | 1.67 | 7729_96 | 1.67 | 12908_22 | 5.00 |  |  | 2323_102 | 0.94 | 6129_111 | 11.32 | 9683_27 | 1.89 | 14098_14 | 9.43 |  |  |
| 2689_70 | 1.67 | 7730_66 | 6.67 | 12913_52 | 0.00 |  |  | 2324_17 | 1.89 | 6135_134 | 0.00 | 9684_97 | 6.60 | 14101_36 | 2.83 |  |  |
| 2691_88 | 8.33 | 7733_73 | 0.00 | 12918_26 | 3.33 |  |  | 2328_76 | 0.94 | 6136_120 | 0.94 | 9687_96 | 0.94 | 14105_19 | 5.66 |  |  |
| 2692_58 | 6.67 | 7737_109 | 8.33 | 12924_134 | 3.33 |  |  | 2329_37 | 0.94 | 6150_27 | 5.66 | 9695_123 | 10.38 | 14113_42 | 2.83 |  |  |
| 2693_120 | 0.00 | 7743_23 | 0.00 | 12927_192 | 8.33 |  |  | 2331_47 | 2.83 | 6151_111 | 1.89 | 9696_65 | 0.94 | 14118_120 | 7.55 |  |  |
| 2695_38 | 0.00 | 7746_81 | 6.67 | 12930_69 | 13.33 |  |  | 2338_109 | 6.60 | 6155_114 | 0.94 | 9701_39 | 13.21 | 14122_180 | 12.26 |  |  |
| 2697_164 | 0.00 | 7752_124 | 0.00 | 12935_76 | 0.00 |  |  | 2341_150 | 11.32 | 6158_46 | 0.00 | 9702_113 | 5.66 | 14123_74 | 5.66 |  |  |
| 2699_113 | 0.00 | 7756_111 | 0.00 | 12938_65 | 1.67 |  |  | 2342_138 | 2.83 | 6159_160 | 6.60 | 9703_38 | 3.77 | 14128_151 | 0.94 |  |  |
| 2703_89 | 5.00 | 7758_15 | 8.33 | 12941_118 | 1.67 |  |  | 2347_189 | 8.49 | 6160_121 | 12.26 | 9704_10 | 3.77 | 14133_121 | 0.00 |  |  |
| 2704_86 | 1.67 | 7761_179 | 3.33 | 12946_81 | 6.67 |  |  | 2351_67 | 10.38 | 6162_116 | 3.77 | 9705_127 | 2.83 | 14138_10 | 8.49 |  |  |
| 2707_118 | 0.00 | 7768_66 | 5.00 | 12947_142 | 0.00 |  |  | 2360_38 | 5.66 | 6166_23 | 1.89 | 9707_88 | 0.00 | 14140_160 | 0.94 |  |  |
| 2718_62 | 6.67 | 7772_97 | 1.67 | 12955_120 | 6.67 |  |  | 2364_126 | 13.21 | 6168_139 | 5.66 | 9709_149 | 12.26 | 14144_175 | 1.89 |  |  |
| 2723_168 | 6.67 | 7776_7 | 11.67 | 12969_122 | 5.00 |  |  | 2368_61 | 5.66 | 6170_75 | 0.00 | 9712_56 | 1.89 | 14146_138 | 0.00 |  |  |
| 2724_134 | 3.33 | 7780_94 | 0.00 | 12973_31 | 1.67 |  |  | 2370_14 | 12.26 | 6171_65 | 4.72 | 9716_107 | 14.15 | 14150_109 | 4.72 |  |  |
| 2727_37 | 3.33 | 7784_51 | 0.00 | 12981_5 | 0.00 |  |  | 2374_145 | 12.26 | 6172_178 | 0.94 | 9718_23 | 0.94 | 14152_88 | 3.77 |  |  |
| 2730_104 | 5.00 | 7785_27 | 3.33 | 12983_124 | 0.00 |  |  | 2377_137 | 11.32 | 6177_35 | 5.66 | 9729_183 | 3.77 | 14158_32 | 6.60 |  |  |
| 2731_183 | 21.67 | 7787_108 | 1.67 | 12988_71 | 6.67 |  |  | 2378_38 | 4.72 | 6179_12 | 0.00 | 9734_119 | 0.94 | 14165_192 | 9.43 |  |  |
| 2750_63 | 6.67 | 7788_183 | 0.00 | 12992_175 | 6.67 |  |  | 2380_100 | 0.00 | 6180_128 | 0.00 | 9736_138 | 2.83 | 14174_58 | 3.77 |  |  |
| 2754_36 | 15.00 | 7797_56 | 0.00 | 12994_15 | 0.00 |  |  | 2382_90 | 0.00 | 6187_92 | 1.89 | 9740_36 | 1.89 | 14175_165 | 3.77 |  |  |
| 2756_15 | 11.67 | 7799_12 | 0.00 | 12995_58 | 10.00 |  |  | 2385_14 | 0.00 | 6191_190 | 13.21 | 9744_18 | 5.66 | 14188_108 | 11.32 |  |  |
| 2759_106 | 8.33 | 7804_75 | 1.67 | 12999_124 | 1.67 |  |  | 2388_38 | 12.26 | 6202_38 | 0.00 | 9745_55 | 15.09 | 14204_41 | 0.00 |  |  |
| 2760_16 | 0.00 | 7808_15 | 5.00 | 13002_30 | 0.00 |  |  | 2392_67 | 15.09 | 6206_57 | 0.00 | 9746_119 | 4.72 | 14209_69 | 2.83 |  |  |
| 2766_164 | 1.67 | 7815_139 | 3.33 | 13004_8 | 3.33 |  |  | 2400_156 | 0.94 | 6209_48 | 9.43 | 9747_68 | 0.94 | 14210_123 | 0.00 |  |  |
| 2767_47 | 1.67 | 7824_10 | 8.33 | 13020_177 | 6.67 |  |  | 2401_35 | 2.83 | 6217_87 | 8.49 | 9749_150 | 10.38 | 14216_29 | 13.21 |  |  |
| 2769_76 | 1.67 | 7825_86 | 3.33 | 13024_115 | 1.67 |  |  | 2402_68 | 1.89 | 6220_25 | 0.00 | 9752_60 | 0.94 | 14224_172 | 0.94 |  |  |
| 2771_115 | 8.33 | 7830_57 | 13.33 | 13028_95 | 0.00 |  |  | 2405_103 | 4.72 | 6221_55 | 7.55 | 9753_36 | 8.49 | 14229_59 | 1.89 |  |  |
| 2772_125 | 8.33 | 7836_151 | 0.00 | 13034_36 | 0.00 |  |  | 2409_139 | 5.66 | 6225_133 | 0.00 | 9754_53 | 3.77 | 14234_18 | 0.94 |  |  |
| 2777_21 | 1.67 | 7837_157 | 3.33 | 13037_130 | 3.33 |  |  | 2416_46 | 1.89 | 6230_32 | 8.49 | 9755_5 | 0.00 | 14238_41 | 11.32 |  |  |
| 2786_14 | 5.00 | 7839_134 | 0.00 | 13039_142 | 13.33 |  |  | 2418_164 | 1.89 | 6240_103 | 0.00 | 9756_23 | 0.94 | 14241_58 | 3.77 |  |  |
| 2790_86 | 3.33 | 7840_149 | 15.00 | 13041_84 | 8.33 |  |  | 2419_68 | 0.94 | 6242_46 | 1.89 | 9764_137 | 3.77 | 14244_36 | 11.32 |  |  |
| 2798_91 | 0.00 | 7844_93 | 0.00 | 13044_43 | 1.67 |  |  | 2422_78 | 0.00 | 6245_82 | 3.77 | 9766_9 | 4.72 | 14250_37 | 10.38 |  |  |
| 2807_18 | 0.00 | 7849_26 | 3.33 | 13055_30 | 0.00 |  |  | 2424_168 | 2.83 | 6246_127 | 1.89 | 9767_43 | 2.83 | 14257_24 | 12.26 |  |  |
| 2813_13 | 3.33 | 7852_67 | 1.67 | 13057_145 | 1.67 |  |  | 2425_176 | 7.55 | 6247_40 | 5.66 | 9770_145 | 3.77 | 14260_94 | 15.09 |  |  |
| 2815_74 | 1.67 | 7856_85 | 6.67 | 13059_98 | 6.67 |  |  | 2428_109 | 1.89 | 6251_164 | 5.66 | 9772_175 | 0.00 | 14264_29 | 6.60 |  |  |
| 2817_30 | 10.00 | 7861_35 | 0.00 | 13060_30 | 16.67 |  |  | 2429_30 | 0.00 | 6260_72 | 9.43 | 9774_28 | 0.94 | 14268_78 | 14.15 |  |  |
| 2818_22 | 0.00 | 7864_41 | 1.67 | 13066_67 | 6.67 |  |  | 2439_11 | 10.38 | 6261_15 | 3.77 | 9777_14 | 5.66 | 14269_86 | 8.49 |  |  |
| 2819_53 | 5.00 | 7871_139 | 1.67 | 13068_194 | 8.33 |  |  | 2445_74 | 0.94 | 6264_74 | 6.60 | 9780_29 | 0.00 | 14270_8 | 0.00 |  |  |
| 2821_64 | 0.00 | 7872_14 | 0.00 | 13072_34 | 0.00 |  |  | 2449_138 | 2.83 | 6276_109 | 1.89 | 9784_102 | 2.83 | 14279_45 | 3.77 |  |  |
| 2822_133 | 0.00 | 7873_37 | 11.67 | 13085_192 | 1.67 |  |  | 2453_61 | 8.49 | 6280_72 | 2.83 | 9785_100 | 15.09 | 14288_79 | 6.60 |  |  |
| 2828_123 | 0.00 | 7878_80 | 0.00 | 13094_105 | 13.33 |  |  | 2455_89 | 0.00 | 6295_108 | 4.72 | 9786_106 | 0.00 | 14289_124 | 0.00 |  |  |
| 2831_56 | 1.67 | 7879_75 | 0.00 | 13096_126 | 0.00 |  |  | 2458_12 | 1.89 | 6300_134 | 0.00 | 9795_8 | 14.15 | 14295_187 | 6.60 |  |  |
| 2834_117 | 0.00 | 7881_133 | 0.00 | 13099_40 | 0.00 |  |  | 2459_8 | 0.00 | 6303_76 | 0.00 | 9798_49 | 12.26 | 14297_59 | 1.89 |  |  |
| 2839_138 | 1.67 | 7883_129 | 1.67 | 13101_41 | 13.33 |  |  | 2465_104 | 5.66 | 6311_5 | 0.94 | 9802_29 | 0.94 | 14307_53 | 7.55 |  |  |
| 2841_15 | 0.00 | 7884_186 | 3.33 | 13105_40 | 8.33 |  |  | 2466_30 | 2.83 | 6312_25 | 0.94 | 9807_157 | 4.72 | 14313_71 | 5.66 |  |  |
| 2859_113 | 3.33 | 7890_89 | 3.33 | 13110_126 | 0.00 |  |  | 2470_41 | 0.00 | 6314_63 | 0.94 | 9812_72 | 0.94 | 14320_17 | 0.94 |  |  |
| 2866_45 | 0.00 | 7892_6 | 3.33 | 13111_107 | 5.00 |  |  | 2472_85 | 1.89 | 6316_48 | 4.72 | 9814_57 | 0.94 | 14330_78 | 0.00 |  |  |
| 2873_95 | 1.67 | 7895_145 | 0.00 | 13116_192 | 0.00 |  |  | 2474_20 | 0.94 | 6318_107 | 11.32 | 9815_148 | 3.77 | 14334_56 | 0.00 |  |  |
| 2882_115 | 5.00 | 7896_23 | 13.33 | 13120_75 | 1.67 |  |  | 2475_132 | 10.38 | 6321_96 | 6.60 | 9816_10 | 0.00 | 14337_126 | 0.94 |  |  |
| 2884_172 | 1.67 | 7901_27 | 0.00 | 13128_19 | 0.00 |  |  | 2476_32 | 0.00 | 6322_30 | 9.43 | 9819_49 | 0.00 | 14338_94 | 0.00 |  |  |
| 2888_14 | 1.67 | 7902_74 | 1.67 | 13129_47 | 3.33 |  |  | 2477_47 | 12.26 | 6330_5 | 5.66 | 9829_155 | 0.00 | 14340_122 | 7.55 |  |  |
| 2889_53 | 3.33 | 7905_111 | 3.33 | 13133_131 | 6.67 |  |  | 2479_143 | 4.72 | 6350_105 | 0.94 | 9831_138 | 0.00 | 14346_100 | 12.26 |  |  |
| 2908_68 | 0.00 | 7907_97 | 0.00 | 13138_131 | 1.67 |  |  | 2482_56 | 0.00 | 6351_20 | 3.77 | 9834_139 | 5.66 | 14350_62 | 10.38 |  |  |
| 2911_151 | 1.67 | 7912_93 | 1.67 | 13144_78 | 8.33 |  |  | 2483_85 | 2.83 | 6353_13 | 11.32 | 9836_8 | 9.43 | 14354_57 | 0.00 |  |  |
| 2913_128 | 3.33 | 7916_79 | 0.00 | 13151_160 | 0.00 |  |  | 2485_46 | 2.83 | 6355_44 | 7.55 | 9837_96 | 0.00 | 14355_92 | 0.94 |  |  |
| 2917_145 | 8.33 | 7919_89 | 0.00 | 13152_7 | 0.00 |  |  | 2486_137 | 0.94 | 6358_20 | 0.94 | 9840_82 | 7.55 | 14356_162 | 9.43 |  |  |
| 2918_148 | 1.67 | 7925_140 | 0.00 | 13153_15 | 5.00 |  |  | 2487_106 | 0.00 | 6359_17 | 3.77 | 9847_44 | 0.00 | 14359_122 | 0.94 |  |  |
| 2922_50 | 1.67 | 7935_101 | 0.00 | 13159_101 | 5.00 |  |  | 2488_113 | 0.00 | 6362_50 | 1.89 | 9848_42 | 6.60 | 14362_127 | 0.00 |  |  |
| 2924_128 | 0.00 | 7936_190 | 8.33 | 13161_36 | 0.00 |  |  | 2490_59 | 6.60 | 6367_11 | 8.49 | 9849_47 | 6.60 | 14363_97 | 3.77 |  |  |
| 2925_8 | 0.00 | 7938_79 | 0.00 | 13162_133 | 0.00 |  |  | 2491_26 | 0.00 | 6368_53 | 0.00 | 9855_36 | 0.00 | 14366_65 | 3.77 |  |  |
| 2928_31 | 0.00 | 7940_36 | 1.67 | 13169_15 | 0.00 |  |  | 2493_20 | 0.94 | 6369_14 | 6.60 | 9857_82 | 0.94 | 14372_10 | 0.94 |  |  |
| 2940_125 | 5.00 | 7947_132 | 0.00 | 13171_97 | 15.00 |  |  | 2494_37 | 9.43 | 6371_78 | 3.77 | 9859_67 | 10.38 | 14373_80 | 1.89 |  |  |
| 2943_5 | 1.67 | 7950_26 | 0.00 | 13178_5 | 0.00 |  |  | 2502_20 | 12.26 | 6373_74 | 1.89 | 9861_183 | 1.89 | 14379_117 | 0.00 |  |  |
| 2944_103 | 0.00 | 7952_117 | 1.67 | 13185_64 | 1.67 |  |  | 2508_89 | 3.77 | 6375_32 | 0.00 | 9864_73 | 12.26 | 14387_12 | 9.43 |  |  |
| 2948_164 | 3.33 | 7956_125 | 1.67 | 13188_109 | 0.00 |  |  | 2510_34 | 5.66 | 6376_59 | 1.89 | 9865_61 | 0.94 | 14390_36 | 7.55 |  |  |
| 2951_57 | 6.67 | 7957_104 | 3.33 | 13194_15 | 1.67 |  |  | 2515_47 | 0.00 | 6378_5 | 1.89 | 9868_153 | 3.77 | 14393_83 | 0.94 |  |  |
| 2957_76 | 3.33 | 7960_123 | 3.33 | 13195_25 | 0.00 |  |  | 2518_102 | 8.49 | 6380_175 | 7.55 | 9869_59 | 3.77 | 14395_178 | 1.89 |  |  |
| 2959_122 | 15.00 | 7972_29 | 0.00 | 13198_142 | 1.67 |  |  | 2523_97 | 8.49 | 6382_68 | 1.89 | 9873_60 | 0.00 | 14400_80 | 9.43 |  |  |
| 2963_22 | 3.33 | 7974_55 | 0.00 | 13202_73 | 3.33 |  |  | 2524_44 | 0.00 | 6384_67 | 0.94 | 9874_91 | 0.00 | 14413_103 | 0.00 |  |  |
| 2965_85 | 0.00 | 7977_85 | 0.00 | 13203_47 | 1.67 |  |  | 2525_64 | 6.60 | 6386_44 | 3.77 | 9875_73 | 14.15 | 14419_170 | 8.49 |  |  |
| 2970_143 | 13.33 | 7979_47 | 11.67 | 13204_97 | 1.67 |  |  | 2527_184 | 5.66 | 6387_114 | 11.32 | 9886_113 | 0.00 | 14421_39 | 0.94 |  |  |
| 2971_81 | 0.00 | 7988_46 | 3.33 | 13206_120 | 0.00 |  |  | 2534_107 | 0.94 | 6389_103 | 7.55 | 9888_106 | 4.72 | 14443_24 | 8.49 |  |  |
| 2972_60 | 6.67 | 7989_49 | 0.00 | 13221_92 | 1.67 |  |  | 2537_109 | 0.94 | 6393_130 | 9.43 | 9889_149 | 0.00 | 14447_76 | 12.26 |  |  |
| 2973_20 | 0.00 | 7991_34 | 1.67 | 13229_113 | 0.00 |  |  | 2542_58 | 1.89 | 6395_88 | 0.94 | 9891_122 | 13.21 | 14451_9 | 8.49 |  |  |
| 2977_38 | 10.00 | 7992_84 | 5.00 | 13248_46 | 11.67 |  |  | 2551_98 | 3.77 | 6397_119 | 1.89 | 9894_119 | 0.00 | 14452_35 | 0.94 |  |  |
| 2987_122 | 5.00 | 7995_101 | 6.67 | 13249_62 | 3.33 |  |  | 2553_63 | 0.00 | 6398_143 | 0.94 | 9896_46 | 0.00 | 14467_106 | 0.94 |  |  |
| 2995_102 | 1.67 | 7997_9 | 5.00 | 13254_76 | 3.33 |  |  | 2556_73 | 3.77 | 6406_171 | 12.26 | 9898_45 | 0.94 | 14470_73 | 5.66 |  |  |
| 3001_139 | 1.67 | 7998_183 | 8.33 | 13265_103 | 1.67 |  |  | 2558_114 | 3.77 | 6407_154 | 2.83 | 9900_24 | 2.83 | 14471_72 | 1.89 |  |  |
| 3004_8 | 0.00 | 7999_48 | 5.00 | 13271_33 | 3.33 |  |  | 2560_108 | 0.94 | 6408_52 | 0.00 | 9904_71 | 1.89 | 14483_133 | 2.83 |  |  |
| 3005_55 | 1.67 | 8000_144 | 0.00 | 13282_116 | 1.67 |  |  | 2561_96 | 12.26 | 6412_22 | 0.00 | 9906_43 | 0.94 | 14484_117 | 12.26 |  |  |
| 3008_92 | 0.00 | 8001_93 | 6.67 | 13284_20 | 0.00 |  |  | 2563_18 | 0.94 | 6413_116 | 3.77 | 9911_95 | 2.83 | 14503_38 | 0.94 |  |  |
| 3009_61 | 3.33 | 8003_71 | 1.67 | 13287_101 | 3.33 |  |  | 2564_59 | 6.60 | 6415_120 | 0.00 | 9915_122 | 9.43 | 14512_104 | 1.89 |  |  |
| 3011_61 | 8.33 | 8007_188 | 8.33 | 13292_87 | 3.33 |  |  | 2565_185 | 0.94 | 6417_103 | 14.15 | 9916_148 | 9.43 | 14516_171 | 12.26 |  |  |
| 3012_18 | 5.00 | 8013_109 | 1.67 | 13295_164 | 8.33 |  |  | 2567_87 | 3.77 | 6423_20 | 9.43 | 9924_105 | 0.00 | 14525_77 | 5.66 |  |  |
| 3022_90 | 5.00 | 8015_53 | 0.00 | 13303_113 | 5.00 |  |  | 2569_132 | 6.60 | 6426_27 | 4.72 | 9925_24 | 0.94 | 14530_58 | 0.00 |  |  |
| 3024_43 | 0.00 | 8026_114 | 0.00 | 13308_34 | 15.00 |  |  | 2570_179 | 6.60 | 6431_95 | 0.00 | 9937_137 | 6.60 | 14544_41 | 0.00 |  |  |
| 3029_6 | 1.67 | 8030_82 | 0.00 | 13312_112 | 3.33 |  |  | 2572_47 | 1.89 | 6433_35 | 0.00 | 9938_98 | 15.09 | 14548_10 | 2.83 |  |  |
| 3037_86 | 11.67 | 8040_118 | 0.00 | 13320_93 | 3.33 |  |  | 2573_99 | 3.77 | 6435_74 | 0.94 | 9944_71 | 6.60 | 14552_29 | 7.55 |  |  |
| 3038_70 | 11.67 | 8044_26 | 1.67 | 13322_172 | 1.67 |  |  | 2574_129 | 0.00 | 6436_57 | 2.83 | 9947_101 | 2.83 | 14555_33 | 0.94 |  |  |
| 3046_86 | 13.33 | 8048_5 | 0.00 | 13323_193 | 1.67 |  |  | 2575_76 | 9.43 | 6444_60 | 2.83 | 9951_173 | 5.66 | 14556_29 | 2.83 |  |  |
| 3047_181 | 6.67 | 8055_96 | 0.00 | 13328_140 | 1.67 |  |  | 2577_125 | 0.94 | 6446_13 | 6.60 | 9952_5 | 5.66 | 14558_43 | 9.43 |  |  |
| 3051_117 | 5.00 | 8056_30 | 0.00 | 13329_177 | 5.00 |  |  | 2578_156 | 0.00 | 6447_106 | 0.00 | 9954_44 | 1.89 | 14563_181 | 5.66 |  |  |
| 3053_36 | 1.67 | 8059_82 | 3.33 | 13336_59 | 1.67 |  |  | 2580_81 | 0.94 | 6452_40 | 5.66 | 9962_158 | 0.00 | 14566_42 | 7.55 |  |  |
| 3055_121 | 0.00 | 8061_117 | 1.67 | 13337_99 | 3.33 |  |  | 2581_39 | 12.26 | 6455_62 | 2.83 | 9963_125 | 1.89 | 14573_92 | 5.66 |  |  |
| 3057_80 | 3.33 | 8065_19 | 1.67 | 13342_141 | 0.00 |  |  | 2584_122 | 0.94 | 6456_7 | 0.94 | 9967_68 | 2.83 | 14587_55 | 13.21 |  |  |
| 3058_117 | 6.67 | 8066_51 | 8.33 | 13346_163 | 5.00 |  |  | 2585_66 | 7.55 | 6457_90 | 6.60 | 9969_35 | 13.21 | 14593_12 | 12.26 |  |  |
| 3072_84 | 0.00 | 8067_94 | 6.67 | 13350_27 | 3.33 |  |  | 2590_73 | 14.15 | 6463_90 | 0.94 | 9972_85 | 4.72 | 14596_38 | 5.66 |  |  |
| 3075_93 | 0.00 | 8068_30 | 5.00 | 13352_57 | 6.67 |  |  | 2591_87 | 8.49 | 6467_103 | 0.00 | 9973_88 | 5.66 | 14598_126 | 0.00 |  |  |
| 3077_124 | 5.00 | 8069_130 | 0.00 | 13353_44 | 6.67 |  |  | 2593_134 | 5.66 | 6470_51 | 10.38 | 9975_117 | 0.00 | 14599_179 | 6.60 |  |  |
| 3085_159 | 1.67 | 8073_158 | 5.00 | 13363_64 | 8.33 |  |  | 2596_169 | 9.43 | 6477_74 | 0.94 | 9976_20 | 0.94 | 14603_100 | 0.94 |  |  |
| 3088_50 | 6.67 | 8074_21 | 1.67 | 13380_25 | 3.33 |  |  | 2599_17 | 0.00 | 6480_20 | 2.83 | 9977_134 | 0.00 | 14607_90 | 0.94 |  |  |
| 3093_51 | 1.67 | 8076_76 | 5.00 | 13382_13 | 0.00 |  |  | 2604_98 | 0.94 | 6482_72 | 0.94 | 9980_80 | 0.00 | 14610_62 | 0.00 |  |  |
| 3100_52 | 5.00 | 8077_29 | 10.00 | 13384_46 | 5.00 |  |  | 2608_85 | 0.00 | 6485_86 | 3.77 | 9988_117 | 11.32 | 14613_152 | 0.94 |  |  |
| 3102_40 | 0.00 | 8078_106 | 0.00 | 13386_34 | 3.33 |  |  | 2612_38 | 0.00 | 6489_79 | 4.72 | 9990_85 | 0.00 | 14619_112 | 11.32 |  |  |
| 3109_23 | 1.67 | 8081_86 | 6.67 | 13388_121 | 5.00 |  |  | 2616_23 | 4.72 | 6491_29 | 13.21 | 9991_96 | 13.21 | 14621_118 | 7.55 |  |  |
| 3110_79 | 1.67 | 8083_46 | 0.00 | 13389_135 | 0.00 |  |  | 2621_18 | 0.94 | 6492_6 | 0.00 | 9992_151 | 0.00 | 14624_52 | 12.26 |  |  |
| 3111_29 | 5.00 | 8084_67 | 10.00 | 13395_5 | 13.33 |  |  | 2623_78 | 0.94 | 6494_117 | 0.94 | 9993_109 | 10.38 | 14628_103 | 6.60 |  |  |
| 3116_128 | 5.00 | 8085_129 | 5.00 | 13405_61 | 3.33 |  |  | 2626_56 | 0.00 | 6496_7 | 10.38 | 9994_155 | 0.94 | 14633_138 | 0.94 |  |  |
| 3118_152 | 11.67 | 8090_82 | 0.00 | 13409_6 | 1.67 |  |  | 2631_90 | 0.00 | 6500_22 | 2.83 | 9997_40 | 2.83 | 14639_143 | 9.43 |  |  |
| 3121_128 | 8.33 | 8091_30 | 6.67 | 13418_97 | 1.67 |  |  | 2641_88 | 2.83 | 6501_63 | 10.38 | 10002_65 | 2.83 | 14640_175 | 0.00 |  |  |
| 3127_91 | 3.33 | 8096_86 | 0.00 | 13419_92 | 1.67 |  |  | 2645_53 | 0.00 | 6508_96 | 10.38 | 10006_165 | 2.83 | 14641_109 | 4.72 |  |  |
| 3128_53 | 8.33 | 8097_193 | 3.33 | 13422_46 | 0.00 |  |  | 2664_15 | 0.00 | 6510_120 | 5.66 | 10008_105 | 4.72 | 14648_140 | 4.72 |  |  |
| 3129_39 | 11.67 | 8101_146 | 1.67 | 13432_55 | 0.00 |  |  | 2666_15 | 13.21 | 6512_107 | 4.72 | 10012_72 | 3.77 | 14653_90 | 9.43 |  |  |
| 3130_67 | 0.00 | 8103_69 | 0.00 | 13435_75 | 0.00 |  |  | 2678_50 | 6.60 | 6518_33 | 7.55 | 10014_159 | 0.00 | 14658_112 | 7.55 |  |  |
| 3131_134 | 8.33 | 8107_39 | 13.33 | 13442_150 | 15.00 |  |  | 2680_58 | 0.00 | 6521_31 | 0.00 | 10015_125 | 12.26 | 14659_108 | 6.60 |  |  |
| 3137_74 | 3.33 | 8111_77 | 0.00 | 13444_44 | 6.67 |  |  | 2681_73 | 0.94 | 6528_98 | 0.00 | 10016_50 | 1.89 | 14663_90 | 2.83 |  |  |
| 3138_91 | 1.67 | 8119_160 | 3.33 | 13452_110 | 5.00 |  |  | 2683_90 | 10.38 | 6533_90 | 4.72 | 10021_74 | 11.32 | 14667_107 | 0.00 |  |  |
| 3140_88 | 0.00 | 8120_79 | 1.67 | 13453_137 | 1.67 |  |  | 2684_103 | 0.94 | 6534_44 | 0.00 | 10022_63 | 2.83 | 14676_32 | 3.77 |  |  |
| 3141_59 | 1.67 | 8121_54 | 3.33 | 13465_47 | 0.00 |  |  | 2685_49 | 1.89 | 6536_86 | 0.00 | 10023_46 | 7.55 | 14681_101 | 4.72 |  |  |
| 3145_60 | 1.67 | 8130_29 | 5.00 | 13478_63 | 6.67 |  |  | 2689_70 | 0.94 | 6538_18 | 0.00 | 10025_172 | 2.83 | 14688_43 | 4.72 |  |  |
| 3146_113 | 8.33 | 8133_82 | 6.67 | 13479_18 | 6.67 |  |  | 2690_79 | 0.94 | 6543_117 | 1.89 | 10027_127 | 4.72 | 14693_88 | 0.00 |  |  |
| 3147_143 | 1.67 | 8136_13 | 3.33 | 13481_93 | 8.33 |  |  | 2691_88 | 9.43 | 6548_8 | 5.66 | 10029_132 | 4.72 | 14695_151 | 4.72 |  |  |
| 3149_125 | 1.67 | 8139_111 | 8.33 | 13490_123 | 5.00 |  |  | 2692_58 | 7.55 | 6550_194 | 9.43 | 10030_36 | 5.66 | 14698_16 | 8.49 |  |  |
| 3150_58 | 5.00 | 8140_22 | 3.33 | 13495_37 | 0.00 |  |  | 2693_120 | 0.00 | 6552_137 | 0.94 | 10036_70 | 0.00 | 14701_88 | 4.72 |  |  |
| 3152_86 | 1.67 | 8143_190 | 1.67 | 13496_141 | 1.67 |  |  | 2695_38 | 0.94 | 6555_121 | 3.77 | 10037_35 | 0.00 | 14703_132 | 0.00 |  |  |
| 3153_62 | 1.67 | 8145_52 | 0.00 | 13502_127 | 3.33 |  |  | 2696_87 | 6.60 | 6564_105 | 0.00 | 10038_29 | 11.32 | 14704_39 | 10.38 |  |  |
| 3157_140 | 8.33 | 8149_14 | 3.33 | 13506_10 | 0.00 |  |  | 2697_164 | 0.00 | 6565_57 | 0.94 | 10051_34 | 11.32 | 14713_64 | 0.00 |  |  |
| 3159_146 | 6.67 | 8150_188 | 3.33 | 13512_5 | 5.00 |  |  | 2698_169 | 11.32 | 6567_63 | 9.43 | 10056_153 | 2.83 | 14720_55 | 8.49 |  |  |
| 3168_75 | 1.67 | 8155_32 | 15.00 | 13513_44 | 1.67 |  |  | 2699_113 | 0.00 | 6568_55 | 7.55 | 10058_121 | 0.94 | 14722_129 | 1.89 |  |  |
| 3174_18 | 10.00 | 8158_79 | 1.67 | 13516_54 | 6.67 |  |  | 2703_89 | 3.77 | 6572_117 | 0.00 | 10059_151 | 0.00 | 14729_61 | 1.89 |  |  |
| 3175_144 | 0.00 | 8165_110 | 3.33 | 13520_161 | 1.67 |  |  | 2704_86 | 0.94 | 6581_29 | 6.60 | 10060_31 | 2.83 | 14734_159 | 9.43 |  |  |
| 3177_51 | 8.33 | 8166_56 | 10.00 | 13534_11 | 10.00 |  |  | 2707_118 | 0.00 | 6582_5 | 9.43 | 10063_9 | 10.38 | 14738_43 | 9.43 |  |  |
| 3181_103 | 0.00 | 8168_61 | 1.67 | 13537_18 | 1.67 |  |  | 2718_62 | 13.21 | 6589_63 | 0.94 | 10064_82 | 2.83 | 14739_76 | 3.77 |  |  |
| 3182_111 | 6.67 | 8170_148 | 0.00 | 13541_36 | 8.33 |  |  | 2723_168 | 6.60 | 6591_36 | 6.60 | 10067_37 | 8.49 | 14747_69 | 6.60 |  |  |
| 3186_101 | 8.33 | 8171_41 | 1.67 | 13549_7 | 0.00 |  |  | 2724_134 | 5.66 | 6595_136 | 7.55 | 10070_36 | 5.66 | 14756_85 | 4.72 |  |  |
| 3187_100 | 0.00 | 8175_74 | 3.33 | 13561_129 | 6.67 |  |  | 2727_37 | 3.77 | 6598_118 | 0.00 | 10072_152 | 11.32 | 14757_131 | 2.83 |  |  |
| 3188_138 | 5.00 | 8176_130 | 1.67 | 13563_82 | 0.00 |  |  | 2730_104 | 2.83 | 6599_76 | 2.83 | 10075_111 | 0.00 | 14769_67 | 10.38 |  |  |
| 3193_53 | 1.67 | 8177_86 | 0.00 | 13564_110 | 0.00 |  |  | 2731_183 | 12.26 | 6603_123 | 12.26 | 10081_9 | 0.94 | 14773_113 | 4.72 |  |  |
| 3194_118 | 6.67 | 8179_23 | 1.67 | 13581_108 | 5.00 |  |  | 2732_7 | 0.94 | 6620_45 | 5.66 | 10085_51 | 10.38 | 14776_75 | 0.00 |  |  |
| 3200_13 | 0.00 | 8182_24 | 3.33 | 13584_100 | 5.00 |  |  | 2744_91 | 3.77 | 6621_90 | 2.83 | 10087_77 | 5.66 | 14783_107 | 0.94 |  |  |
| 3201_172 | 1.67 | 8188_95 | 3.33 | 13591_10 | 16.67 |  |  | 2748_25 | 2.83 | 6623_62 | 7.55 | 10089_158 | 0.94 | 14787_36 | 2.83 |  |  |
| 3203_86 | 1.67 | 8190_47 | 11.67 | 13592_32 | 3.33 |  |  | 2750_63 | 6.60 | 6625_120 | 3.77 | 10094_73 | 2.83 | 14795_64 | 3.77 |  |  |
| 3209_143 | 10.00 | 8193_62 | 1.67 | 13593_86 | 1.67 |  |  | 2754_36 | 10.38 | 6626_33 | 3.77 | 10095_145 | 5.66 | 14797_99 | 0.94 |  |  |
| 3212_67 | 0.00 | 8196_123 | 1.67 | 13601_103 | 5.00 |  |  | 2756_15 | 12.26 | 6628_104 | 0.94 | 10101_23 | 6.60 | 14798_88 | 0.94 |  |  |
| 3214_20 | 0.00 | 8197_103 | 3.33 | 13602_151 | 11.67 |  |  | 2759_106 | 9.43 | 6629_85 | 0.00 | 10107_174 | 3.77 | 14799_82 | 0.94 |  |  |
| 3216_73 | 0.00 | 8198_157 | 0.00 | 13607_10 | 0.00 |  |  | 2760_16 | 5.66 | 6635_41 | 3.77 | 10109_97 | 0.00 | 14800_124 | 10.38 |  |  |
| 3228_108 | 0.00 | 8201_92 | 0.00 | 13608_63 | 0.00 |  |  | 2766_164 | 0.94 | 6636_160 | 0.00 | 10112_17 | 0.00 | 14808_120 | 0.00 |  |  |
| 3231_10 | 11.67 | 8206_115 | 1.67 | 13612_115 | 3.33 |  |  | 2767_47 | 0.94 | 6637_73 | 12.26 | 10117_49 | 4.72 | 14809_71 | 9.43 |  |  |
| 3233_76 | 0.00 | 8212_69 | 0.00 | 13618_60 | 5.00 |  |  | 2769_76 | 2.83 | 6639_46 | 0.94 | 10119_45 | 5.66 | 14812_112 | 12.26 |  |  |
| 3238_81 | 10.00 | 8213_16 | 0.00 | 13626_95 | 1.67 |  |  | 2771_115 | 8.49 | 6640_9 | 12.26 | 10120_65 | 0.00 | 14813_170 | 7.55 |  |  |
| 3239_21 | 1.67 | 8214_139 | 1.67 | 13627_85 | 1.67 |  |  | 2772_125 | 10.38 | 6641_78 | 0.00 | 10128_50 | 3.77 | 14826_115 | 0.00 |  |  |
| 3241_54 | 1.67 | 8215_37 | 3.33 | 13630_52 | 1.67 |  |  | 2777_21 | 1.89 | 6642_39 | 7.55 | 10131_123 | 0.94 | 14828_112 | 0.00 |  |  |
| 3243_110 | 6.67 | 8218_140 | 0.00 | 13635_78 | 3.33 |  |  | 2786_14 | 8.49 | 6643_73 | 4.72 | 10134_52 | 6.60 | 14838_143 | 1.89 |  |  |
| 3248_9 | 0.00 | 8219_64 | 5.00 | 13637_154 | 13.33 |  |  | 2790_86 | 5.66 | 6654_73 | 2.83 | 10135_136 | 6.60 | 14840_118 | 2.83 |  |  |
| 3251_112 | 0.00 | 8220_37 | 0.00 | 13646_41 | 5.00 |  |  | 2792_11 | 14.15 | 6661_151 | 4.72 | 10138_58 | 5.66 | 14841_92 | 3.77 |  |  |
| 3252_166 | 0.00 | 8227_57 | 8.33 | 13653_135 | 0.00 |  |  | 2796_51 | 4.72 | 6662_130 | 0.00 | 10144_61 | 4.72 | 14844_158 | 4.72 |  |  |
| 3254_151 | 8.33 | 8229_26 | 5.00 | 13673_176 | 5.00 |  |  | 2798_91 | 0.94 | 6663_52 | 1.89 | 10147_113 | 0.00 | 14845_101 | 6.60 |  |  |
| 3259_81 | 5.00 | 8238_13 | 0.00 | 13677_26 | 16.67 |  |  | 2807_18 | 4.72 | 6664_83 | 0.00 | 10148_9 | 3.77 | 14853_51 | 0.94 |  |  |
| 3263_9 | 0.00 | 8239_20 | 1.67 | 13680_22 | 0.00 |  |  | 2813_13 | 4.72 | 6665_42 | 6.60 | 10150_36 | 5.66 | 14856_81 | 2.83 |  |  |
| 3265_49 | 0.00 | 8240_41 | 0.00 | 13681_100 | 1.67 |  |  | 2815_74 | 1.89 | 6666_151 | 11.32 | 10153_71 | 0.94 | 14864_113 | 2.83 |  |  |
| 3266_172 | 3.33 | 8242_99 | 1.67 | 13683_99 | 0.00 |  |  | 2817_30 | 6.60 | 6669_192 | 7.55 | 10154_107 | 7.55 | 14866_28 | 0.94 |  |  |
| 3273_50 | 5.00 | 8244_148 | 1.67 | 13684_64 | 6.67 |  |  | 2818_22 | 3.77 | 6677_93 | 0.00 | 10156_116 | 4.72 | 14872_115 | 6.60 |  |  |
| 3275_14 | 3.33 | 8246_72 | 0.00 | 13690_18 | 0.00 |  |  | 2819_53 | 4.72 | 6684_104 | 0.00 | 10157_55 | 0.00 | 14876_64 | 3.77 |  |  |
| 3280_85 | 1.67 | 8247_74 | 0.00 | 13696_48 | 0.00 |  |  | 2821_64 | 0.00 | 6691_98 | 6.60 | 10159_52 | 0.00 | 14878_138 | 10.38 |  |  |
| 3285_109 | 3.33 | 8255_132 | 3.33 | 13700_130 | 8.33 |  |  | 2822_133 | 0.00 | 6695_82 | 4.72 | 10165_53 | 1.89 | 14886_49 | 4.72 |  |  |
| 3287_63 | 1.67 | 8259_17 | 1.67 | 13702_109 | 6.67 |  |  | 2826_194 | 1.89 | 6699_185 | 11.32 | 10166_139 | 1.89 | 14897_127 | 7.55 |  |  |
| 3291_13 | 5.00 | 8260_99 | 5.00 | 13705_33 | 0.00 |  |  | 2828_123 | 0.00 | 6701_10 | 9.43 | 10175_43 | 1.89 | 14898_60 | 0.00 |  |  |
| 3299_18 | 6.67 | 8261_141 | 6.67 | 13708_14 | 0.00 |  |  | 2831_56 | 0.94 | 6702_128 | 1.89 | 10178_12 | 6.60 | 14905_27 | 0.00 |  |  |
| 3304_98 | 1.67 | 8264_100 | 0.00 | 13713_140 | 1.67 |  |  | 2834_117 | 0.00 | 6704_16 | 0.00 | 10182_65 | 5.66 | 14909_89 | 11.32 |  |  |
| 3307_58 | 5.00 | 8267_179 | 3.33 | 13714_129 | 3.33 |  |  | 2839_138 | 1.89 | 6705_112 | 4.72 | 10190_8 | 2.83 | 14921_190 | 8.49 |  |  |
| 3308_52 | 3.33 | 8273_51 | 0.00 | 13715_55 | 1.67 |  |  | 2841_15 | 0.00 | 6707_14 | 6.60 | 10193_21 | 0.94 | 14928_10 | 2.83 |  |  |
| 3311_86 | 10.00 | 8276_35 | 1.67 | 13718_116 | 8.33 |  |  | 2854_93 | 4.72 | 6708_76 | 0.94 | 10194_87 | 0.00 | 14929_10 | 3.77 |  |  |
| 3314_116 | 5.00 | 8277_83 | 0.00 | 13722_92 | 8.33 |  |  | 2859_113 | 1.89 | 6710_19 | 1.89 | 10196_131 | 6.60 | 14939_141 | 0.94 |  |  |
| 3321_25 | 6.67 | 8279_43 | 0.00 | 13726_21 | 5.00 |  |  | 2866_45 | 0.00 | 6713_132 | 0.00 | 10198_151 | 0.00 | 14941_116 | 7.55 |  |  |
| 3324_171 | 3.33 | 8288_10 | 3.33 | 13730_104 | 3.33 |  |  | 2873_95 | 9.43 | 6715_9 | 0.94 | 10199_88 | 8.49 | 14958_158 | 7.55 |  |  |
| 3325_132 | 1.67 | 8290_159 | 13.33 | 13735_89 | 3.33 |  |  | 2882_115 | 5.66 | 6717_35 | 0.94 | 10201_171 | 4.72 | 14972_5 | 1.89 |  |  |
| 3329_24 | 0.00 | 8291_125 | 0.00 | 13736_54 | 5.00 |  |  | 2884_172 | 1.89 | 6721_193 | 14.15 | 10206_12 | 0.94 | 14979_156 | 10.38 |  |  |
| 3331_12 | 0.00 | 8296_96 | 1.67 | 13737_84 | 1.67 |  |  | 2887_109 | 3.77 | 6722_6 | 0.00 | 10208_28 | 13.21 | 14984_9 | 2.83 |  |  |
| 3336_18 | 0.00 | 8297_102 | 0.00 | 13740_106 | 1.67 |  |  | 2888_14 | 0.94 | 6729_107 | 1.89 | 10214_98 | 4.72 | 14985_111 | 0.00 |  |  |
| 3339_40 | 3.33 | 8301_187 | 0.00 | 13745_110 | 3.33 |  |  | 2889_53 | 3.77 | 6730_101 | 5.66 | 10215_10 | 6.60 | 14989_117 | 9.43 |  |  |
| 3340_58 | 3.33 | 8303_54 | 0.00 | 13746_36 | 3.33 |  |  | 2908_68 | 0.00 | 6733_17 | 12.26 | 10220_13 | 7.55 | 14991_46 | 6.60 |  |  |
| 3341_38 | 6.67 | 8312_36 | 1.67 | 13756_106 | 5.00 |  |  | 2911_151 | 3.77 | 6736_143 | 0.00 | 10223_38 | 0.00 | 15001_133 | 0.00 |  |  |
| 3342_55 | 1.67 | 8313_111 | 0.00 | 13770_82 | 6.67 |  |  | 2913_128 | 1.89 | 6742_106 | 1.89 | 10226_84 | 0.94 | 15006_10 | 5.66 |  |  |
| 3350_102 | 3.33 | 8317_116 | 6.67 | 13785_26 | 1.67 |  |  | 2917_145 | 7.55 | 6743_119 | 6.60 | 10227_131 | 11.32 | 15008_50 | 0.00 |  |  |
| 3357_17 | 3.33 | 8318_55 | 1.67 | 13786_27 | 1.67 |  |  | 2918_148 | 0.94 | 6749_87 | 8.49 | 10229_59 | 7.55 | 15021_5 | 0.94 |  |  |
| 3362_85 | 0.00 | 8322_119 | 3.33 | 13788_90 | 8.33 |  |  | 2922_50 | 0.94 | 6753_28 | 0.94 | 10230_120 | 9.43 | 15025_28 | 11.32 |  |  |
| 3365_39 | 1.67 | 8324_64 | 11.67 | 13789_71 | 6.67 |  |  | 2924_128 | 0.00 | 6754_23 | 5.66 | 10231_72 | 7.55 | 15029_99 | 9.43 |  |  |
| 3366_130 | 3.33 | 8329_177 | 5.00 | 13793_10 | 3.33 |  |  | 2925_8 | 0.00 | 6756_26 | 7.55 | 10232_95 | 0.94 | 15040_142 | 9.43 |  |  |
| 3369_128 | 0.00 | 8335_6 | 0.00 | 13797_153 | 0.00 |  |  | 2928_31 | 0.00 | 6767_79 | 0.00 | 10233_112 | 0.00 | 15046_103 | 3.77 |  |  |
| 3370_117 | 3.33 | 8338_37 | 1.67 | 13801_163 | 0.00 |  |  | 2940_125 | 4.72 | 6772_86 | 0.00 | 10237_34 | 0.94 | 15052_119 | 1.89 |  |  |
| 3371_88 | 0.00 | 8339_98 | 0.00 | 13802_42 | 0.00 |  |  | 2943_5 | 0.94 | 6774_158 | 3.77 | 10240_62 | 0.94 | 15053_38 | 1.89 |  |  |
| 3372_183 | 11.67 | 8344_42 | 0.00 | 13812_21 | 3.33 |  |  | 2944_103 | 0.00 | 6775_53 | 12.26 | 10243_141 | 0.94 | 15060_58 | 0.00 |  |  |
| 3381_91 | 10.00 | 8345_120 | 1.67 | 13815_91 | 8.33 |  |  | 2948_164 | 4.72 | 6778_121 | 0.00 | 10244_87 | 0.00 | 15068_153 | 5.66 |  |  |
| 3382_159 | 0.00 | 8348_136 | 0.00 | 13823_83 | 3.33 |  |  | 2951_57 | 4.72 | 6779_70 | 10.38 | 10245_80 | 0.00 | 15075_33 | 11.32 |  |  |
| 3383_110 | 5.00 | 8353_119 | 0.00 | 13826_94 | 0.00 |  |  | 2953_99 | 0.94 | 6780_168 | 1.89 | 10247_52 | 2.83 | 15086_18 | 5.66 |  |  |
| 3385_37 | 1.67 | 8354_110 | 0.00 | 13828_111 | 1.67 |  |  | 2957_76 | 2.83 | 6790_62 | 0.00 | 10248_19 | 5.66 | 15090_186 | 5.66 |  |  |
| 3389_34 | 0.00 | 8366_68 | 3.33 | 13835_131 | 0.00 |  |  | 2959_122 | 13.21 | 6793_22 | 1.89 | 10249_123 | 3.77 | 15095_49 | 1.89 |  |  |
| 3401_89 | 0.00 | 8377_54 | 0.00 | 13837_110 | 11.67 |  |  | 2963_22 | 7.55 | 6795_144 | 0.00 | 10251_155 | 7.55 | 15114_56 | 6.60 |  |  |
| 3405_21 | 0.00 | 8380_107 | 0.00 | 13842_149 | 3.33 |  |  | 2965_85 | 0.00 | 6796_112 | 0.00 | 10257_46 | 0.00 | 15115_137 | 0.94 |  |  |
| 3409_86 | 1.67 | 8382_22 | 3.33 | 13849_8 | 0.00 |  |  | 2970_143 | 9.43 | 6798_109 | 0.00 | 10259_8 | 0.94 | 15120_7 | 3.77 |  |  |
| 3412_159 | 13.33 | 8383_94 | 0.00 | 13853_136 | 8.33 |  |  | 2971_81 | 1.89 | 6801_114 | 1.89 | 10264_121 | 1.89 | 15123_61 | 0.00 |  |  |
| 3413_10 | 0.00 | 8384_108 | 8.33 | 13855_87 | 0.00 |  |  | 2972_60 | 7.55 | 6806_96 | 9.43 | 10265_97 | 0.00 | 15135_178 | 4.72 |  |  |
| 3415_52 | 3.33 | 8385_64 | 0.00 | 13857_153 | 6.67 |  |  | 2973_20 | 0.00 | 6809_44 | 0.94 | 10270_107 | 0.94 | 15158_87 | 0.94 |  |  |
| 3416_27 | 6.67 | 8392_31 | 8.33 | 13871_63 | 1.67 |  |  | 2977_38 | 5.66 | 6810_130 | 2.83 | 10272_125 | 8.49 | 15160_133 | 4.72 |  |  |
| 3417_40 | 0.00 | 8394_26 | 0.00 | 13872_116 | 1.67 |  |  | 2983_132 | 11.32 | 6811_100 | 0.94 | 10273_24 | 1.89 | 15166_34 | 0.00 |  |  |
| 3420_21 | 0.00 | 8396_28 | 0.00 | 13881_117 | 1.67 |  |  | 2987_122 | 8.49 | 6817_30 | 0.94 | 10277_70 | 1.89 | 15170_90 | 5.66 |  |  |
| 3427_14 | 0.00 | 8399_44 | 5.00 | 13884_96 | 3.33 |  |  | 2995_102 | 0.94 | 6818_13 | 0.00 | 10278_93 | 0.00 | 15172_142 | 3.77 |  |  |
| 3432_85 | 10.00 | 8401_83 | 0.00 | 13888_52 | 1.67 |  |  | 3001_139 | 7.55 | 6823_80 | 3.77 | 10280_75 | 0.00 | 15174_80 | 7.55 |  |  |
| 3439_66 | 3.33 | 8402_5 | 0.00 | 13892_42 | 11.67 |  |  | 3004_8 | 0.00 | 6825_60 | 4.72 | 10281_29 | 0.94 | 15179_91 | 6.60 |  |  |
| 3446_38 | 3.33 | 8403_51 | 3.33 | 13893_118 | 5.00 |  |  | 3005_55 | 0.94 | 6829_72 | 2.83 | 10283_32 | 0.00 | 15180_107 | 3.77 |  |  |
| 3447_55 | 6.67 | 8408_139 | 6.67 | 13918_19 | 0.00 |  |  | 3008_92 | 0.00 | 6830_76 | 0.00 | 10286_86 | 0.00 | 15185_107 | 0.00 |  |  |
| 3449_15 | 3.33 | 8412_152 | 1.67 | 13922_13 | 5.00 |  |  | 3009_61 | 6.60 | 6832_120 | 1.89 | 10288_103 | 4.72 | 15206_48 | 1.89 |  |  |
| 3453_61 | 5.00 | 8417_47 | 10.00 | 13930_59 | 0.00 |  |  | 3011_61 | 4.72 | 6834_168 | 2.83 | 10289_46 | 2.83 | 15207_31 | 0.00 |  |  |
| 3456_82 | 3.33 | 8427_95 | 1.67 | 13933_168 | 1.67 |  |  | 3012_18 | 14.15 | 6837_68 | 0.00 | 10292_161 | 3.77 | 15209_157 | 5.66 |  |  |
| 3459_129 | 16.67 | 8435_8 | 5.00 | 13936_43 | 1.67 |  |  | 3022_90 | 14.15 | 6839_89 | 0.00 | 10296_75 | 1.89 | 15212_95 | 0.94 |  |  |
| 3460_156 | 3.33 | 8440_136 | 8.33 | 13941_92 | 13.33 |  |  | 3023_77 | 11.32 | 6841_45 | 1.89 | 10297_38 | 12.26 | 15217_137 | 7.55 |  |  |
| 3465_53 | 0.00 | 8452_172 | 1.67 | 13944_48 | 0.00 |  |  | 3024_43 | 0.00 | 6844_41 | 0.00 | 10298_116 | 0.00 | 15219_149 | 7.55 |  |  |
| 3468_45 | 1.67 | 8460_13 | 3.33 | 13955_13 | 3.33 |  |  | 3025_41 | 7.55 | 6848_154 | 7.55 | 10299_111 | 10.38 | 15222_62 | 2.83 |  |  |
| 3486_133 | 0.00 | 8461_52 | 1.67 | 13959_111 | 0.00 |  |  | 3029_6 | 0.94 | 6866_75 | 5.66 | 10302_15 | 0.00 | 15226_101 | 3.77 |  |  |
| 3498_126 | 0.00 | 8462_42 | 0.00 | 13960_152 | 1.67 |  |  | 3032_64 | 5.66 | 6867_123 | 7.55 | 10304_24 | 5.66 | 15228_164 | 7.55 |  |  |
| 3504_100 | 5.00 | 8467_9 | 0.00 | 13965_34 | 5.00 |  |  | 3037_86 | 10.38 | 6877_17 | 6.60 | 10308_48 | 2.83 | 15229_186 | 7.55 |  |  |
| 3510_66 | 0.00 | 8468_116 | 1.67 | 13973_50 | 0.00 |  |  | 3038_70 | 10.38 | 6884_54 | 1.89 | 10309_69 | 1.89 | 15231_95 | 4.72 |  |  |
| 3512_155 | 13.33 | 8470_105 | 0.00 | 13981_184 | 8.33 |  |  | 3046_86 | 9.43 | 6885_178 | 2.83 | 10317_68 | 0.00 | 15233_66 | 2.83 |  |  |
| 3522_16 | 0.00 | 8479_139 | 0.00 | 13988_14 | 8.33 |  |  | 3047_181 | 10.38 | 6889_43 | 2.83 | 10318_57 | 0.00 | 15259_163 | 4.72 |  |  |
| 3523_98 | 16.67 | 8483_105 | 5.00 | 13992_10 | 0.00 |  |  | 3051_117 | 7.55 | 6897_84 | 3.77 | 10320_148 | 9.43 | 15261_146 | 5.66 |  |  |
| 3525_82 | 5.00 | 8486_78 | 18.33 | 13993_27 | 1.67 |  |  | 3052_45 | 9.43 | 6900_65 | 0.00 | 10322_44 | 0.00 | 15262_23 | 12.26 |  |  |
| 3530_61 | 11.67 | 8488_85 | 0.00 | 13995_19 | 1.67 |  |  | 3053_36 | 0.94 | 6901_142 | 3.77 | 10323_57 | 0.00 | 15266_130 | 10.38 |  |  |
| 3534_91 | 8.33 | 8490_96 | 0.00 | 14003_10 | 0.00 |  |  | 3055_121 | 0.00 | 6908_115 | 7.55 | 10324_138 | 7.55 | 15275_29 | 0.00 |  |  |
| 3536_72 | 3.33 | 8493_142 | 6.67 | 14018_97 | 8.33 |  |  | 3057_80 | 1.89 | 6909_122 | 3.77 | 10325_86 | 0.94 | 15276_22 | 8.49 |  |  |
| 3543_46 | 1.67 | 8495_20 | 1.67 | 14023_100 | 5.00 |  |  | 3058_117 | 4.72 | 6914_153 | 9.43 | 10326_53 | 0.00 | 15278_63 | 1.89 |  |  |
| 3544_22 | 1.67 | 8499_168 | 0.00 | 14030_65 | 5.00 |  |  | 3072_84 | 1.89 | 6915_63 | 15.09 | 10327_146 | 0.94 | 15282_146 | 1.89 |  |  |
| 3545_55 | 3.33 | 8501_63 | 0.00 | 14033_129 | 3.33 |  |  | 3074_74 | 0.94 | 6920_187 | 4.72 | 10329_89 | 0.00 | 15288_66 | 0.94 |  |  |
| 3546_71 | 3.33 | 8504_38 | 0.00 | 14042_34 | 0.00 |  |  | 3075_93 | 0.94 | 6921_28 | 1.89 | 10337_158 | 13.21 | 15296_117 | 0.00 |  |  |
| 3550_174 | 5.00 | 8505_56 | 6.67 | 14046_84 | 6.67 |  |  | 3077_124 | 2.83 | 6923_46 | 0.94 | 10339_128 | 7.55 | 15299_35 | 6.60 |  |  |
| 3554_88 | 13.33 | 8507_73 | 0.00 | 14054_146 | 13.33 |  |  | 3083_18 | 0.94 | 6927_112 | 0.94 | 10341_132 | 6.60 | 15302_66 | 0.00 |  |  |
| 3555_31 | 0.00 | 8511_135 | 0.00 | 14059_89 | 11.67 |  |  | 3085_159 | 1.89 | 6936_6 | 0.00 | 10343_76 | 1.89 | 15306_41 | 8.49 |  |  |
| 3560_151 | 6.67 | 8512_71 | 3.33 | 14060_104 | 8.33 |  |  | 3088_50 | 4.72 | 6940_33 | 0.00 | 10344_7 | 5.66 | 15313_71 | 12.26 |  |  |
| 3562_110 | 0.00 | 8517_34 | 10.00 | 14061_193 | 11.67 |  |  | 3093_51 | 4.72 | 6942_80 | 0.00 | 10346_104 | 0.00 | 15319_34 | 7.55 |  |  |
| 3564_112 | 1.67 | 8520_22 | 8.33 | 14066_100 | 8.33 |  |  | 3100_52 | 7.55 | 6943_105 | 1.89 | 10349_48 | 12.26 | 15322_109 | 0.00 |  |  |
| 3566_7 | 1.67 | 8523_99 | 0.00 | 14068_34 | 0.00 |  |  | 3102_40 | 0.00 | 6944_55 | 0.94 | 10356_149 | 5.66 | 15323_10 | 12.26 |  |  |
| 3567_19 | 0.00 | 8524_12 | 11.67 | 14076_143 | 0.00 |  |  | 3109_23 | 8.49 | 6947_85 | 0.00 | 10360_119 | 3.77 | 15324_19 | 0.00 |  |  |
| 3569_70 | 0.00 | 8525_139 | 8.33 | 14077_57 | 10.00 |  |  | 3110_79 | 0.94 | 6951_65 | 1.89 | 10362_76 | 2.83 | 15335_21 | 3.77 |  |  |
| 3574_123 | 0.00 | 8533_31 | 0.00 | 14080_113 | 0.00 |  |  | 3111_29 | 10.38 | 6955_23 | 6.60 | 10367_82 | 12.26 | 15340_105 | 4.72 |  |  |
| 3577_52 | 3.33 | 8538_173 | 6.67 | 14082_24 | 3.33 |  |  | 3114_24 | 0.00 | 6956_56 | 2.83 | 10370_111 | 1.89 | 15341_124 | 0.94 |  |  |
| 3578_93 | 5.00 | 8541_62 | 0.00 | 14098_14 | 6.67 |  |  | 3116_128 | 4.72 | 6958_133 | 0.00 | 10373_38 | 1.89 | 15344_108 | 3.77 |  |  |
| 3582_71 | 1.67 | 8542_177 | 0.00 | 14101_36 | 5.00 |  |  | 3118_152 | 11.32 | 6961_73 | 0.00 | 10379_92 | 6.60 | 15349_185 | 2.83 |  |  |
| 3587_132 | 1.67 | 8547_92 | 1.67 | 14105_19 | 3.33 |  |  | 3121_128 | 8.49 | 6964_134 | 1.89 | 10383_72 | 0.00 | 15358_33 | 2.83 |  |  |
| 3595_103 | 6.67 | 8556_78 | 1.67 | 14113_42 | 3.33 |  |  | 3127_91 | 1.89 | 6965_25 | 3.77 | 10385_26 | 11.32 | 15361_38 | 12.26 |  |  |
| 3598_158 | 1.67 | 8563_41 | 0.00 | 14118_120 | 1.67 |  |  | 3128_53 | 8.49 | 6967_124 | 0.94 | 10386_80 | 3.77 | 15362_53 | 0.00 |  |  |
| 3600_111 | 11.67 | 8567_30 | 0.00 | 14122_180 | 10.00 |  |  | 3129_39 | 8.49 | 6972_136 | 5.66 | 10388_106 | 0.00 | 15369_100 | 0.94 |  |  |
| 3605_97 | 8.33 | 8570_19 | 6.67 | 14123_74 | 3.33 |  |  | 3130_67 | 4.72 | 6981_95 | 0.00 | 10390_122 | 0.00 | 15382_43 | 0.94 |  |  |
| 3611_83 | 1.67 | 8572_9 | 1.67 | 14128_151 | 0.00 |  |  | 3131_134 | 6.60 | 6984_18 | 4.72 | 10393_34 | 12.26 | 15383_102 | 1.89 |  |  |
| 3613_133 | 0.00 | 8574_44 | 0.00 | 14133_121 | 0.00 |  |  | 3137_74 | 1.89 | 6990_142 | 4.72 | 10394_65 | 0.00 | 15384_170 | 0.00 |  |  |
| 3614_37 | 0.00 | 8575_68 | 8.33 | 14138_10 | 8.33 |  |  | 3138_91 | 1.89 | 6994_24 | 8.49 | 10402_172 | 10.38 | 15386_124 | 3.77 |  |  |
| 3619_51 | 1.67 | 8580_12 | 0.00 | 14140_160 | 1.67 |  |  | 3140_88 | 0.94 | 6995_152 | 1.89 | 10407_92 | 5.66 | 15389_45 | 4.72 |  |  |
| 3622_145 | 3.33 | 8585_75 | 0.00 | 14144_175 | 1.67 |  |  | 3141_59 | 0.94 | 6999_36 | 0.94 | 10410_136 | 12.26 | 15392_185 | 8.49 |  |  |
| 3626_156 | 3.33 | 8592_89 | 8.33 | 14146_138 | 0.00 |  |  | 3145_60 | 0.94 | 7000_56 | 8.49 | 10412_182 | 7.55 | 15393_85 | 10.38 |  |  |
| 3627_90 | 0.00 | 8597_158 | 11.67 | 14150_109 | 0.00 |  |  | 3146_113 | 10.38 | 7002_163 | 4.72 | 10414_68 | 1.89 | 15407_159 | 8.49 |  |  |
| 3632_62 | 8.33 | 8602_38 | 0.00 | 14152_88 | 3.33 |  |  | 3147_143 | 0.94 | 7012_98 | 1.89 | 10416_76 | 0.94 | 15409_65 | 2.83 |  |  |
| 3634_82 | 1.67 | 8611_115 | 0.00 | 14165_192 | 6.67 |  |  | 3149_125 | 0.94 | 7015_24 | 1.89 | 10418_8 | 8.49 | 15419_97 | 5.66 |  |  |
| 3636_69 | 3.33 | 8616_48 | 0.00 | 14174_58 | 6.67 |  |  | 3150_58 | 4.72 | 7016_163 | 0.00 | 10419_13 | 2.83 | 15434_168 | 11.32 |  |  |
| 3637_138 | 3.33 | 8619_96 | 1.67 | 14175_165 | 0.00 |  |  | 3152_86 | 0.94 | 7018_56 | 0.00 | 10421_177 | 8.49 | 15436_136 | 14.15 |  |  |
| 3641_10 | 10.00 | 8621_33 | 1.67 | 14188_108 | 13.33 |  |  | 3153_62 | 3.77 | 7019_59 | 1.89 | 10425_78 | 0.00 | 15438_25 | 9.43 |  |  |
| 3648_14 | 3.33 | 8624_15 | 1.67 | 14209_69 | 1.67 |  |  | 3157_140 | 11.32 | 7023_16 | 5.66 | 10428_30 | 1.89 | 15447_97 | 0.94 |  |  |
| 3650_130 | 6.67 | 8628_98 | 0.00 | 14210_123 | 0.00 |  |  | 3159_146 | 5.66 | 7027_66 | 8.49 | 10437_160 | 10.38 | 15449_53 | 4.72 |  |  |
| 3655_42 | 0.00 | 8633_74 | 5.00 | 14216_29 | 13.33 |  |  | 3168_75 | 0.94 | 7028_16 | 10.38 | 10439_56 | 2.83 | 15457_60 | 0.94 |  |  |
| 3656_43 | 5.00 | 8639_18 | 0.00 | 14224_172 | 0.00 |  |  | 3174_18 | 9.43 | 7029_65 | 5.66 | 10444_84 | 0.00 | 15465_154 | 8.49 |  |  |
| 3657_115 | 10.00 | 8641_101 | 1.67 | 14229_59 | 1.67 |  |  | 3175_144 | 3.77 | 7033_97 | 8.49 | 10445_82 | 3.77 | 15475_96 | 7.55 |  |  |
| 3659_167 | 5.00 | 8642_86 | 1.67 | 14234_18 | 1.67 |  |  | 3177_51 | 6.60 | 7036_80 | 0.00 | 10448_131 | 6.60 | 15481_74 | 15.09 |  |  |
| 3660_36 | 3.33 | 8643_83 | 6.67 | 14238_41 | 11.67 |  |  | 3181_103 | 0.00 | 7037_105 | 3.77 | 10449_112 | 0.00 | 15497_47 | 3.77 |  |  |
| 3661_86 | 0.00 | 8648_78 | 1.67 | 14241_58 | 5.00 |  |  | 3182_111 | 8.49 | 7040_30 | 1.89 | 10451_127 | 0.00 | 15507_127 | 0.00 |  |  |
| 3662_124 | 1.67 | 8651_88 | 3.33 | 14244_36 | 11.67 |  |  | 3186_101 | 5.66 | 7043_58 | 2.83 | 10452_126 | 1.89 | 15516_123 | 8.49 |  |  |
| 3669_47 | 3.33 | 8652_35 | 1.67 | 14257_24 | 8.33 |  |  | 3187_100 | 0.00 | 7045_92 | 9.43 | 10453_64 | 5.66 | 15519_116 | 0.94 |  |  |
| 3670_156 | 0.00 | 8654_81 | 0.00 | 14260_94 | 11.67 |  |  | 3188_138 | 9.43 | 7047_56 | 0.94 | 10454_172 | 8.49 | 15523_112 | 0.00 |  |  |
| 3671_71 | 0.00 | 8663_72 | 0.00 | 14264_29 | 5.00 |  |  | 3193_53 | 6.60 | 7050_81 | 2.83 | 10455_52 | 0.94 | 15525_49 | 0.94 |  |  |
| 3680_34 | 5.00 | 8674_151 | 3.33 | 14268_78 | 15.00 |  |  | 3194_118 | 3.77 | 7052_68 | 1.89 | 10456_67 | 8.49 | 15526_114 | 2.83 |  |  |
| 3681_95 | 3.33 | 8679_49 | 0.00 | 14269_86 | 5.00 |  |  | 3200_13 | 1.89 | 7055_26 | 6.60 | 10459_78 | 2.83 | 15532_11 | 4.72 |  |  |
| 3686_102 | 0.00 | 8680_136 | 11.67 | 14270_8 | 0.00 |  |  | 3201_172 | 1.89 | 7058_75 | 7.55 | 10460_129 | 9.43 | 15545_40 | 15.09 |  |  |
| 3687_91 | 11.67 | 8686_90 | 11.67 | 14279_45 | 0.00 |  |  | 3203_86 | 1.89 | 7062_180 | 1.89 | 10463_24 | 5.66 | 15548_109 | 0.94 |  |  |
| 3688_8 | 11.67 | 8688_28 | 0.00 | 14288_79 | 6.67 |  |  | 3209_143 | 11.32 | 7063_18 | 1.89 | 10469_188 | 10.38 | 15551_76 | 0.00 |  |  |
| 3692_91 | 6.67 | 8694_150 | 0.00 | 14289_124 | 0.00 |  |  | 3212_67 | 0.00 | 7064_30 | 0.00 | 10470_153 | 0.00 | 15557_58 | 1.89 |  |  |
| 3693_166 | 0.00 | 8697_7 | 0.00 | 14295_187 | 6.67 |  |  | 3214_20 | 0.00 | 7070_50 | 6.60 | 10474_37 | 7.55 | 15562_94 | 0.94 |  |  |
| 3696_70 | 0.00 | 8702_128 | 0.00 | 14297_59 | 0.00 |  |  | 3216_73 | 0.94 | 7071_111 | 0.94 | 10476_137 | 1.89 | 15563_71 | 0.94 |  |  |
| 3699_40 | 0.00 | 8704_18 | 6.67 | 14307_53 | 10.00 |  |  | 3228_108 | 0.00 | 7072_172 | 0.00 | 10479_30 | 6.60 | 15565_70 | 0.00 |  |  |
| 3700_84 | 1.67 | 8706_22 | 0.00 | 14320_17 | 0.00 |  |  | 3231_10 | 11.32 | 7074_159 | 0.00 | 10484_15 | 6.60 | 15568_175 | 6.60 |  |  |
| 3701_80 | 1.67 | 8715_88 | 0.00 | 14337_126 | 0.00 |  |  | 3233_76 | 0.00 | 7077_111 | 0.00 | 10485_41 | 0.00 | 15569_44 | 0.94 |  |  |
| 3702_179 | 5.00 | 8719_97 | 3.33 | 14338_94 | 0.00 |  |  | 3238_81 | 6.60 | 7080_92 | 6.60 | 10487_124 | 0.00 | 15575_48 | 4.72 |  |  |
| 3704_86 | 5.00 | 8721_57 | 13.33 | 14340_122 | 1.67 |  |  | 3239_21 | 3.77 | 7085_50 | 0.00 | 10496_89 | 1.89 | 15583_108 | 0.00 |  |  |
| 3706_78 | 1.67 | 8723_133 | 8.33 | 14346_100 | 0.00 |  |  | 3241_54 | 2.83 | 7090_75 | 0.00 | 10502_19 | 1.89 | 15585_43 | 4.72 |  |  |
| 3712_45 | 1.67 | 8730_67 | 10.00 | 14350_62 | 11.67 |  |  | 3243_110 | 3.77 | 7093_73 | 1.89 | 10503_57 | 1.89 | 15588_149 | 0.94 |  |  |
| 3717_95 | 3.33 | 8731_77 | 0.00 | 14354_57 | 0.00 |  |  | 3244_75 | 13.21 | 7096_163 | 5.66 | 10505_39 | 10.38 | 15595_133 | 14.15 |  |  |
| 3721_111 | 0.00 | 8739_177 | 3.33 | 14355_92 | 1.67 |  |  | 3246_118 | 1.89 | 7099_76 | 6.60 | 10506_83 | 0.00 | 15601_144 | 6.60 |  |  |
| 3727_163 | 1.67 | 8746_40 | 3.33 | 14356_162 | 5.00 |  |  | 3248_9 | 0.94 | 7100_149 | 0.94 | 10511_83 | 4.72 | 15602_74 | 4.72 |  |  |
| 3728_71 | 0.00 | 8751_127 | 1.67 | 14359_122 | 1.67 |  |  | 3251_112 | 0.00 | 7103_67 | 13.21 | 10514_29 | 1.89 | 15603_73 | 0.00 |  |  |
| 3735_15 | 5.00 | 8753_33 | 0.00 | 14362_127 | 0.00 |  |  | 3252_166 | 0.00 | 7110_148 | 0.00 | 10518_155 | 0.94 | 15613_184 | 0.00 |  |  |
| 3736_38 | 1.67 | 8757_175 | 0.00 | 14363_97 | 3.33 |  |  | 3254_151 | 11.32 | 7112_78 | 0.00 | 10519_41 | 2.83 | 15617_184 | 14.15 |  |  |
| 3737_34 | 1.67 | 8763_173 | 8.33 | 14366_65 | 1.67 |  |  | 3258_64 | 0.00 | 7116_116 | 0.00 | 10521_52 | 2.83 | 15622_63 | 0.94 |  |  |
| 3745_9 | 1.67 | 8767_81 | 0.00 | 14372_10 | 0.00 |  |  | 3259_81 | 3.77 | 7124_132 | 0.00 | 10531_38 | 7.55 | 15624_37 | 0.00 |  |  |
| 3754_53 | 0.00 | 8769_102 | 0.00 | 14373_80 | 3.33 |  |  | 3263_9 | 0.00 | 7126_23 | 6.60 | 10533_74 | 4.72 | 15628_126 | 0.00 |  |  |
| 3757_150 | 0.00 | 8770_11 | 0.00 | 14379_117 | 0.00 |  |  | 3265_49 | 2.83 | 7127_6 | 4.72 | 10535_50 | 9.43 | 15637_94 | 0.00 |  |  |
| 3766_114 | 0.00 | 8772_134 | 10.00 | 14390_36 | 5.00 |  |  | 3266_172 | 2.83 | 7139_59 | 5.66 | 10536_121 | 6.60 | 15639_171 | 0.00 |  |  |
| 3769_153 | 0.00 | 8777_29 | 6.67 | 14393_83 | 0.00 |  |  | 3268_40 | 0.94 | 7142_100 | 0.94 | 10537_140 | 4.72 | 15640_83 | 9.43 |  |  |
| 3770_98 | 3.33 | 8782_79 | 1.67 | 14400_80 | 5.00 |  |  | 3273_50 | 2.83 | 7145_141 | 2.83 | 10538_32 | 0.00 | 15666_12 | 6.60 |  |  |
| 3775_50 | 3.33 | 8785_5 | 1.67 | 14413_103 | 0.00 |  |  | 3275_14 | 4.72 | 7148_112 | 0.94 | 10546_128 | 8.49 | 15690_72 | 8.49 |  |  |
| 3778_126 | 1.67 | 8786_59 | 1.67 | 14421_39 | 1.67 |  |  | 3280_85 | 3.77 | 7149_29 | 10.38 | 10548_37 | 2.83 | 15771_32 | 11.32 |  |  |
| 3779_131 | 1.67 | 8791_52 | 3.33 | 14443_24 | 8.33 |  |  | 3285_109 | 4.72 | 7150_117 | 4.72 | 10551_30 | 0.94 | 15779_14 | 13.21 |  |  |
| 3782_106 | 0.00 | 8805_153 | 1.67 | 14447_76 | 10.00 |  |  | 3287_63 | 2.83 | 7151_60 | 0.00 | 10554_85 | 0.94 | 15790_86 | 3.77 |  |  |
| 3784_122 | 0.00 | 8809_38 | 1.67 | 14451_9 | 1.67 |  |  | 3291_13 | 6.60 | 7160_27 | 5.66 | 10556_14 | 0.00 | 15838_88 | 8.49 |  |  |
| 3785_98 | 0.00 | 8810_29 | 11.67 | 14467_106 | 1.67 |  |  | 3299_18 | 7.55 | 7163_168 | 0.00 | 10558_107 | 0.00 | 15855_95 | 14.15 |  |  |
| 3786_153 | 5.00 | 8812_38 | 11.67 | 14470_73 | 5.00 |  |  | 3304_98 | 5.66 | 7169_80 | 0.94 | 10559_66 | 5.66 | 16005_99 | 10.38 |  |  |
| 3790_92 | 1.67 | 8813_107 | 0.00 | 14471_72 | 1.67 |  |  | 3307_58 | 11.32 | 7170_28 | 0.94 | 10561_151 | 4.72 | 16109_98 | 9.43 |  |  |
| 3792_84 | 1.67 | 8816_79 | 0.00 | 14483_133 | 3.33 |  |  | 3308_52 | 9.43 | 7171_71 | 0.00 | 10564_151 | 12.26 | 16180_160 | 6.60 |  |  |
| 3793_84 | 0.00 | 8817_7 | 1.67 | 14484_117 | 10.00 |  |  | 3311_86 | 5.66 | 7173_102 | 0.00 | 10565_143 | 10.38 | 16199_80 | 9.43 |  |  |
| 3795_98 | 1.67 | 8820_106 | 6.67 | 14503_38 | 1.67 |  |  | 3314_116 | 5.66 | 7175_27 | 0.00 | 10568_108 | 6.60 | 16224_17 | 12.26 |  |  |
| 3799_75 | 1.67 | 8826_37 | 0.00 | 14512_104 | 0.00 |  |  | 3321_25 | 5.66 | 7181_27 | 0.94 | 10575_114 | 0.00 | 16233_81 | 6.60 |  |  |
| 3803_44 | 15.00 | 8829_46 | 0.00 | 14516_171 | 10.00 |  |  | 3324_171 | 2.83 | 7188_106 | 0.94 | 10577_108 | 9.43 | 16252_70 | 9.43 |  |  |
| 3807_135 | 1.67 | 8830_123 | 0.00 | 14525_77 | 6.67 |  |  | 3325_132 | 0.94 | 7190_79 | 0.00 | 10579_170 | 2.83 | 16304_88 | 8.49 |  |  |
| 3808_71 | 5.00 | 8832_116 | 0.00 | 14530_58 | 0.00 |  |  | 3329_24 | 0.00 | 7194_54 | 1.89 | 10582_166 | 5.66 | 16359_148 | 12.26 |  |  |
| 3809_60 | 5.00 | 8837_180 | 5.00 | 14544_41 | 0.00 |  |  | 3331_12 | 0.94 | 7199_139 | 0.94 | 10587_92 | 0.94 | 16361_145 | 12.26 |  |  |
| 3820_28 | 0.00 | 8838_125 | 6.67 | 14548_10 | 1.67 |  |  | 3336_18 | 0.00 | 7202_72 | 0.00 | 10590_134 | 5.66 | 16430_7 | 8.49 |  |  |
| 3826_95 | 0.00 | 8842_121 | 5.00 | 14555_33 | 1.67 |  |  | 3339_40 | 1.89 | 7204_43 | 0.00 | 10597_43 | 1.89 | 16459_59 | 11.32 |  |  |
| 3830_26 | 1.67 | 8848_67 | 0.00 | 14556_29 | 3.33 |  |  | 3340_58 | 6.60 | 7206_144 | 0.00 | 10609_34 | 0.00 | 16505_99 | 9.43 |  |  |
| 3838_106 | 13.33 | 8855_40 | 0.00 | 14558_43 | 8.33 |  |  | 3341_38 | 7.55 | 7215_150 | 0.00 | 10611_73 | 10.38 | 16515_65 | 14.15 |  |  |
| 3843_110 | 3.33 | 8857_42 | 1.67 | 14563_181 | 3.33 |  |  | 3342_55 | 0.94 | 7218_73 | 3.77 | 10616_100 | 0.00 | 16617_36 | 11.32 |  |  |
| 3854_7 | 1.67 | 8858_164 | 3.33 | 14566_42 | 6.67 |  |  | 3350_102 | 7.55 | 7221_157 | 1.89 | 10621_115 | 0.00 | 16702_18 | 11.32 |  |  |
| 3860_120 | 0.00 | 8868_44 | 10.00 | 14573_92 | 5.00 |  |  | 3357_17 | 1.89 | 7223_92 | 0.00 | 10622_151 | 9.43 | 16731_118 | 5.66 |  |  |
| 3861_23 | 1.67 | 8871_93 | 0.00 | 14587_55 | 13.33 |  |  | 3362_85 | 0.00 | 7224_11 | 1.89 | 10628_32 | 0.94 | 16814_120 | 14.15 |  |  |
| 3865_122 | 0.00 | 8872_82 | 0.00 | 14593_12 | 10.00 |  |  | 3363_7 | 0.94 | 7226_83 | 0.00 | 10630_62 | 2.83 | 16884_13 | 13.21 |  |  |
| 3867_138 | 11.67 | 8887_101 | 0.00 | 14598_126 | 0.00 |  |  | 3365_39 | 1.89 | 7227_137 | 0.00 | 10640_132 | 7.55 | 16900_70 | 11.32 |  |  |
| 3871_10 | 21.67 | 8889_74 | 5.00 | 14599_179 | 5.00 |  |  | 3366_130 | 1.89 | 7229_82 | 6.60 | 10641_96 | 13.21 | 16915_31 | 8.49 |  |  |
| 3877_17 | 1.67 | 8890_66 | 0.00 | 14603_100 | 1.67 |  |  | 3369_128 | 1.89 | 7231_43 | 5.66 | 10643_20 | 10.38 | 16988_64 | 15.09 |  |  |
| 3892_136 | 1.67 | 8893_73 | 0.00 | 14607_90 | 0.00 |  |  | 3370_117 | 1.89 | 7233_166 | 15.09 | 10646_75 | 4.72 | 17020_46 | 13.21 |  |  |
| 3897_30 | 6.67 | 8894_138 | 0.00 | 14613_152 | 0.00 |  |  | 3371_88 | 0.00 | 7234_56 | 1.89 | 10647_43 | 1.89 | 17035_125 | 1.89 |  |  |
| 3902_28 | 3.33 | 8897_117 | 0.00 | 14619_112 | 10.00 |  |  | 3372_183 | 12.26 | 7236_71 | 2.83 | 10654_115 | 9.43 | 17041_111 | 7.55 |  |  |
| 3904_48 | 0.00 | 8898_22 | 8.33 | 14621_118 | 3.33 |  |  | 3379_69 | 10.38 | 7241_17 | 8.49 | 10663_93 | 0.94 | 17052_106 | 10.38 |  |  |
| 3905_149 | 8.33 | 8900_192 | 11.67 | 14624_52 | 6.67 |  |  | 3381_91 | 11.32 | 7243_107 | 0.00 | 10665_41 | 2.83 | 17063_39 | 11.32 |  |  |
| 3909_19 | 0.00 | 8901_40 | 0.00 | 14628_103 | 6.67 |  |  | 3382_159 | 0.00 | 7251_167 | 5.66 | 10666_48 | 3.77 | 17074_72 | 5.66 |  |  |
| 3913_31 | 11.67 | 8907_148 | 1.67 | 14633_138 | 0.00 |  |  | 3383_110 | 5.66 | 7253_115 | 3.77 | 10669_32 | 0.00 | 17083_54 | 14.15 |  |  |
| 3915_93 | 3.33 | 8908_169 | 8.33 | 14639_143 | 8.33 |  |  | 3385_37 | 0.94 | 7255_145 | 7.55 | 10671_38 | 0.00 | 17085_63 | 2.83 |  |  |
| 3916_26 | 1.67 | 8915_90 | 1.67 | 14640_175 | 0.00 |  |  | 3389_34 | 0.00 | 7260_32 | 1.89 | 10676_38 | 4.72 | 17088_59 | 13.21 |  |  |
| 3917_51 | 0.00 | 8919_79 | 13.33 | 14641_109 | 0.00 |  |  | 3392_162 | 5.66 | 7261_60 | 0.94 | 10681_59 | 1.89 | 17131_67 | 11.32 |  |  |
| 3928_117 | 1.67 | 8923_57 | 1.67 | 14648_140 | 5.00 |  |  | 3401_89 | 0.00 | 7265_58 | 0.00 | 10682_20 | 0.00 | 17204_106 | 9.43 |  |  |
| 3937_179 | 1.67 | 8924_21 | 1.67 | 14653_90 | 6.67 |  |  | 3405_21 | 1.89 | 7271_138 | 0.00 | 10683_49 | 4.72 | 17282_67 | 14.15 |  |  |
| 3939_20 | 0.00 | 8925_106 | 10.00 | 14658_112 | 10.00 |  |  | 3409_86 | 0.94 | 7275_109 | 8.49 | 10684_127 | 2.83 | 17358_192 | 11.32 |  |  |
| 3945_75 | 0.00 | 8926_38 | 0.00 | 14659_108 | 3.33 |  |  | 3412_159 | 11.32 | 7279_87 | 2.83 | 10685_110 | 5.66 | 17440_85 | 11.32 |  |  |
| 3949_75 | 0.00 | 8929_103 | 10.00 | 14663_90 | 0.00 |  |  | 3413_10 | 1.89 | 7281_103 | 1.89 | 10689_90 | 4.72 | 17458_50 | 2.83 |  |  |
| 3951_82 | 0.00 | 8931_109 | 1.67 | 14667_107 | 0.00 |  |  | 3415_52 | 1.89 | 7290_139 | 0.94 | 10692_50 | 0.94 | 17529_43 | 12.26 |  |  |
| 3953_148 | 3.33 | 8932_168 | 1.67 | 14676_32 | 3.33 |  |  | 3416_27 | 9.43 | 7292_128 | 0.00 | 10695_56 | 3.77 | 17586_77 | 13.21 |  |  |
| 3955_10 | 1.67 | 8936_65 | 3.33 | 14688_43 | 0.00 |  |  | 3417_40 | 0.00 | 7293_88 | 0.00 | 10697_61 | 0.00 | 17623_112 | 12.26 |  |  |
| 3969_16 | 0.00 | 8938_150 | 0.00 | 14693_88 | 0.00 |  |  | 3420_21 | 0.94 | 7294_192 | 0.94 | 10702_15 | 0.00 | 17638_119 | 8.49 |  |  |
| 3971_127 | 0.00 | 8945_140 | 13.33 | 14695_151 | 1.67 |  |  | 3427_14 | 0.00 | 7297_60 | 0.00 | 10706_203 | 6.60 | 17659_147 | 8.49 |  |  |
| 3972_139 | 0.00 | 8946_48 | 3.33 | 14698_16 | 6.67 |  |  | 3432_85 | 13.21 | 7302_47 | 0.94 | 10707_70 | 0.94 | 17734_50 | 8.49 |  |  |
| 3973_80 | 0.00 | 8948_110 | 1.67 | 14701_88 | 5.00 |  |  | 3439_66 | 2.83 | 7307_122 | 0.94 | 10709_133 | 0.00 | 17789_64 | 10.38 |  |  |
| 3974_22 | 5.00 | 8952_109 | 3.33 | 14704_39 | 8.33 |  |  | 3440_96 | 3.77 | 7309_21 | 1.89 | 10710_96 | 2.83 | 17795_136 | 15.09 |  |  |
| 3976_155 | 3.33 | 8953_97 | 0.00 | 14713_64 | 0.00 |  |  | 3446_38 | 2.83 | 7310_162 | 0.00 | 10711_133 | 0.00 | 17799_89 | 13.21 |  |  |
| 3979_87 | 8.33 | 8955_116 | 1.67 | 14720_55 | 10.00 |  |  | 3447_55 | 7.55 | 7318_81 | 3.77 | 10712_126 | 7.55 | 17808_119 | 12.26 |  |  |
| 3980_159 | 1.67 | 8956_76 | 3.33 | 14722_129 | 3.33 |  |  | 3449_15 | 3.77 | 7320_28 | 0.00 | 10714_21 | 9.43 | 17855_85 | 4.72 |  |  |
| 3984_69 | 3.33 | 8959_39 | 8.33 | 14729_61 | 0.00 |  |  | 3453_61 | 5.66 | 7322_116 | 10.38 | 10717_22 | 5.66 | 17868_108 | 13.21 |  |  |
| 3986_59 | 1.67 | 8962_50 | 0.00 | 14734_159 | 6.67 |  |  | 3456_82 | 1.89 | 7325_50 | 0.94 | 10719_119 | 6.60 | 17870_91 | 11.32 |  |  |
| 3988_117 | 21.67 | 8967_42 | 13.33 | 14738_43 | 3.33 |  |  | 3459_129 | 13.21 | 7331_32 | 3.77 | 10720_72 | 0.94 | 17873_113 | 11.32 |  |  |
| 3992_61 | 13.33 | 8968_67 | 3.33 | 14739_76 | 3.33 |  |  | 3460_156 | 4.72 | 7338_157 | 1.89 | 10726_78 | 5.66 | 17908_9 | 15.09 |  |  |
| 3993_140 | 1.67 | 8970_16 | 1.67 | 14747_69 | 5.00 |  |  | 3465_53 | 0.94 | 7342_77 | 1.89 | 10729_145 | 0.94 | 18002_205 | 14.15 |  |  |
| 4004_14 | 8.33 | 8973_172 | 5.00 | 14756_85 | 3.33 |  |  | 3468_45 | 1.89 | 7343_79 | 5.66 | 10740_76 | 0.00 | 18461_90 | 10.38 |  |  |
| 4005_52 | 6.67 | 8976_18 | 0.00 | 14757_131 | 1.67 |  |  | 3486_133 | 0.00 | 7344_10 | 2.83 | 10743_76 | 0.94 | 18785_110 | 9.43 |  |  |
| 4006_50 | 13.33 | 8977_110 | 0.00 | 14769_67 | 6.67 |  |  | 3498_126 | 0.00 | 7345_124 | 3.77 | 10744_91 | 7.55 | 19004_30 | 12.26 |  |  |
| 4009_93 | 16.67 | 8978_159 | 10.00 | 14773_113 | 1.67 |  |  | 3504_100 | 7.55 | 7351_52 | 0.00 | 10745_86 | 3.77 | 27864_114 | 0.00 |  |  |
| 4012_96 | 8.33 | 8979_130 | 1.67 | 14776_75 | 0.00 |  |  | 3510_66 | 0.94 | 7356_75 | 8.49 | 10752_100 | 1.89 | 46788_60 | 3.77 |  |  |
| 4013_6 | 0.00 | 8982_139 | 5.00 | 14783_107 | 1.67 |  |  | 3512_155 | 9.43 | 7359_9 | 0.94 | 10753_117 | 1.89 | 47019_76 | 3.77 |  |  |
| 4016_30 | 0.00 | 8985_51 | 0.00 | 14787_36 | 1.67 |  |  | 3522_16 | 0.00 | 7360_143 | 6.60 | 10754_100 | 10.38 | 48066_67 | 1.89 |  |  |
| 4025_11 | 6.67 | 8987_103 | 1.67 | 14795_64 | 3.33 |  |  | 3523_98 | 9.43 | 7365_41 | 4.72 | 10755_42 | 0.00 | 48165_156 | 8.49 |  |  |
| 4027_76 | 6.67 | 8992_106 | 0.00 | 14797_99 | 1.67 |  |  | 3524_78 | 8.49 | 7366_135 | 0.00 | 10758_74 | 9.43 | 52488_83 | 7.55 |  |  |
| 4030_8 | 1.67 | 8995_110 | 5.00 | 14798_88 | 0.00 |  |  | 3525_82 | 5.66 | 7367_89 | 0.94 | 10765_71 | 4.72 | 52584_92 | 2.83 |  |  |
| 4031_62 | 1.67 | 8996_14 | 8.33 | 14799_82 | 1.67 |  |  | 3530_61 | 6.60 | 7370_9 | 2.83 | 10766_149 | 10.38 | 56414_95 | 16.04 |  |  |
| 4040_27 | 1.67 | 8998_130 | 0.00 | 14800_124 | 5.00 |  |  | 3534_91 | 10.38 | 7372_79 | 0.00 | 10770_62 | 0.94 | 57688_42 | 6.60 |  |  |
| 4041_78 | 5.00 | 9000_22 | 3.33 | 14809_71 | 6.67 |  |  | 3535_45 | 0.00 | 7376_88 | 0.00 | 10771_128 | 10.38 | 59595_15 | 12.26 |  |  |
| 4049_64 | 0.00 | 9003_150 | 1.67 | 14812_112 | 6.67 |  |  | 3536_72 | 1.89 | 7379_67 | 13.21 | 10773_13 | 6.60 | 59857_36 | 6.60 |  |  |
| 4051_57 | 0.00 | 9006_105 | 0.00 | 14813_170 | 6.67 |  |  | 3539_77 | 2.83 | 7382_35 | 0.00 | 10775_52 | 0.00 | 64248_101 | 0.94 |  |  |
| 4052_93 | 0.00 | 9008_10 | 0.00 | 14826_115 | 0.00 |  |  | 3540_8 | 4.72 | 7384_33 | 0.00 | 10777_42 | 10.38 | 65447_157 | 0.00 |  |  |
| 4056_132 | 5.00 | 9010_65 | 1.67 | 14838_143 | 3.33 |  |  | 3543_46 | 0.94 | 7385_145 | 2.83 | 10778_15 | 0.94 | 67800_109 | 9.43 |  |  |
| 4059_141 | 3.33 | 9015_115 | 1.67 | 14840_118 | 1.67 |  |  | 3544_22 | 7.55 | 7386_31 | 0.00 | 10780_99 | 8.49 | 72819_229 | 5.66 |  |  |
| 4060_78 | 1.67 | 9016_22 | 6.67 | 14841_92 | 1.67 |  |  | 3545_55 | 2.83 | 7390_14 | 0.94 | 10783_165 | 1.89 | 83869_25 | 3.77 |  |  |
| 4064_123 | 0.00 | 9024_73 | 1.67 | 14844_158 | 3.33 |  |  | 3546_71 | 2.83 | 7396_44 | 0.00 | 10789_31 | 1.89 | 85489_48 | 12.26 |  |  |
| 4065_76 | 0.00 | 9033_83 | 1.67 | 14845_101 | 8.33 |  |  | 3550_174 | 8.49 | 7397_94 | 12.26 | 10793_67 | 0.00 | 97241_126 | 8.49 |  |  |
| 4066_44 | 0.00 | 9037_100 | 15.00 | 14853_51 | 0.00 |  |  | 3554_88 | 12.26 | 7398_68 | 0.00 | 10804_49 | 0.00 | 100159_34 | 12.26 |  |  |
| 4068_191 | 6.67 | 9040_127 | 6.67 | 14864_113 | 3.33 |  |  | 3555_31 | 1.89 | 7403_58 | 3.77 | 10805_17 | 8.49 | 104333_82 | 4.72 |  |  |
| 4073_16 | 1.67 | 9041_21 | 5.00 | 14872_115 | 10.00 |  |  | 3560_151 | 4.72 | 7405_95 | 1.89 | 10811_55 | 3.77 |  |  |  |  |
| 4075_68 | 0.00 | 9049_62 | 0.00 | 14876_64 | 5.00 |  |  | 3562_110 | 0.00 | 7406_107 | 1.89 | 10814_166 | 0.00 |  |  |  |  |
| 4078_143 | 0.00 | 9058_81 | 3.33 | 14878_138 | 5.00 |  |  |  |  |  |  |  |  |  |  |  |  |
| 4079_64 | 0.00 | 9059_82 | 1.67 | 14886_49 | 0.00 |  |  |  |  |  |  |  |  |  |  |  |  |
| 4080_141 | 6.67 | 9061_95 | 0.00 | 14897_127 | 5.00 |  |  |  |  |  |  |  |  |  |  |  |  |
| 4085_28 | 0.00 | 9062_29 | 5.00 | 14898_60 | 0.00 |  |  |  |  |  |  |  |  |  |  |  |  |
| 4086_72 | 5.00 | 9071_51 | 1.67 | 14905_27 | 0.00 |  |  |  |  |  |  |  |  |  |  |  |  |
| 4088_64 | 1.67 | 9072_126 | 10.00 | 14909_89 | 11.67 |  |  |  |  |  |  |  |  |  |  |  |  |
| 4090_70 | 5.00 | 9080_37 | 6.67 | 14921_190 | 8.33 |  |  |  |  |  |  |  |  |  |  |  |  |
| 4091_87 | 0.00 | 9083_132 | 3.33 | 14928_10 | 1.67 |  |  |  |  |  |  |  |  |  |  |  |  |
| 4101_29 | 3.33 | 9084_146 | 0.00 | 14929_10 | 5.00 |  |  |  |  |  |  |  |  |  |  |  |  |
| 4104_100 | 13.33 | 9091_69 | 0.00 | 14939_141 | 0.00 |  |  |  |  |  |  |  |  |  |  |  |  |
| 4105_90 | 3.33 | 9094_9 | 1.67 | 14941_116 | 6.67 |  |  |  |  |  |  |  |  |  |  |  |  |
| 4106_21 | 5.00 | 9095_54 | 0.00 | 14958_158 | 10.00 |  |  |  |  |  |  |  |  |  |  |  |  |
| 4110_145 | 0.00 | 9097_58 | 13.33 | 14972_5 | 1.67 |  |  |  |  |  |  |  |  |  |  |  |  |
| 4111_144 | 8.33 | 9108_125 | 0.00 | 14979_156 | 11.67 |  |  |  |  |  |  |  |  |  |  |  |  |
| 4116_88 | 8.33 | 9109_18 | 0.00 | 14984_9 | 1.67 |  |  |  |  |  |  |  |  |  |  |  |  |
| 4117_5 | 1.67 | 9116_76 | 0.00 | 14985_111 | 0.00 |  |  |  |  |  |  |  |  |  |  |  |  |
| 4120_85 | 0.00 | 9119_96 | 3.33 | 14989_117 | 6.67 |  |  |  |  |  |  |  |  |  |  |  |  |
| 4124_8 | 0.00 | 9124_180 | 30.00 | 14991_46 | 5.00 |  |  |  |  |  |  |  |  |  |  |  |  |
| 4128_107 | 5.00 | 9131_46 | 0.00 | 15001_133 | 0.00 |  |  |  |  |  |  |  |  |  |  |  |  |
| 4129_30 | 1.67 | 9134_69 | 10.00 | 15006_10 | 8.33 |  |  |  |  |  |  |  |  |  |  |  |  |
| 4132_109 | 10.00 | 9136_5 | 0.00 | 15021_5 | 1.67 |  |  |  |  |  |  |  |  |  |  |  |  |
| 4133_86 | 0.00 | 9139_47 | 3.33 | 15025_28 | 10.00 |  |  |  |  |  |  |  |  |  |  |  |  |
| 4135_27 | 0.00 | 9147_10 | 1.67 | 15029_99 | 5.00 |  |  |  |  |  |  |  |  |  |  |  |  |
| 4141_129 | 0.00 | 9150_22 | 0.00 | 15046_103 | 1.67 |  |  |  |  |  |  |  |  |  |  |  |  |
| 4145_23 | 1.67 | 9151_139 | 0.00 | 15052_119 | 1.67 |  |  |  |  |  |  |  |  |  |  |  |  |
| 4148_11 | 1.67 | 9152_8 | 0.00 | 15053_38 | 1.67 |  |  |  |  |  |  |  |  |  |  |  |  |
| 4150_62 | 0.00 | 9154_44 | 0.00 | 15060_58 | 0.00 |  |  |  |  |  |  |  |  |  |  |  |  |
| 4151_116 | 8.33 | 9158_115 | 1.67 | 15068_153 | 1.67 |  |  |  |  |  |  |  |  |  |  |  |  |
| 4154_153 | 0.00 | 9163_100 | 0.00 | 15075_33 | 8.33 |  |  |  |  |  |  |  |  |  |  |  |  |
| 4157_156 | 1.67 | 9169_111 | 10.00 | 15090_186 | 6.67 |  |  |  |  |  |  |  |  |  |  |  |  |
| 4165_67 | 10.00 | 9174_168 | 3.33 | 15095_49 | 3.33 |  |  |  |  |  |  |  |  |  |  |  |  |
| 4169_40 | 1.67 | 9177_59 | 5.00 | 15114_56 | 3.33 |  |  |  |  |  |  |  |  |  |  |  |  |
| 4170_96 | 15.00 | 9179_68 | 5.00 | 15120_7 | 3.33 |  |  |  |  |  |  |  |  |  |  |  |  |
| 4174_153 | 0.00 | 9180_16 | 1.67 | 15123_61 | 0.00 |  |  |  |  |  |  |  |  |  |  |  |  |
| 4175_55 | 6.67 | 9183_22 | 1.67 | 15135_178 | 5.00 |  |  |  |  |  |  |  |  |  |  |  |  |
| 4180_172 | 11.67 | 9189_127 | 25.00 | 15158_87 | 1.67 |  |  |  |  |  |  |  |  |  |  |  |  |
| 4187_63 | 6.67 | 9194_122 | 0.00 | 15160_133 | 1.67 |  |  |  |  |  |  |  |  |  |  |  |  |
| 4195_138 | 0.00 | 9200_11 | 3.33 | 15166_34 | 0.00 |  |  |  |  |  |  |  |  |  |  |  |  |
| 4201_25 | 13.33 | 9201_41 | 0.00 | 15170_90 | 3.33 |  |  |  |  |  |  |  |  |  |  |  |  |
| 4204_28 | 0.00 | 9202_107 | 0.00 | 15172_142 | 1.67 |  |  |  |  |  |  |  |  |  |  |  |  |
| 4206_73 | 0.00 | 9204_133 | 3.33 | 15174_80 | 8.33 |  |  |  |  |  |  |  |  |  |  |  |  |
| 4214_37 | 0.00 | 9207_158 | 0.00 | 15180_107 | 1.67 |  |  |  |  |  |  |  |  |  |  |  |  |
| 4216_151 | 1.67 | 9210_78 | 16.67 | 15185_107 | 0.00 |  |  |  |  |  |  |  |  |  |  |  |  |
| 4221_30 | 5.00 | 9212_122 | 1.67 | 15206_48 | 3.33 |  |  |  |  |  |  |  |  |  |  |  |  |
| 4222_17 | 1.67 | 9216_120 | 0.00 | 15207_31 | 0.00 |  |  |  |  |  |  |  |  |  |  |  |  |
| 4232_118 | 1.67 | 9219_113 | 1.67 | 15209_157 | 6.67 |  |  |  |  |  |  |  |  |  |  |  |  |
| 4239_99 | 3.33 | 9222_19 | 1.67 | 15212_95 | 0.00 |  |  |  |  |  |  |  |  |  |  |  |  |
| 4243_82 | 1.67 | 9224_64 | 11.67 | 15219_149 | 1.67 |  |  |  |  |  |  |  |  |  |  |  |  |
| 4244_100 | 13.33 | 9225_95 | 5.00 | 15222_62 | 1.67 |  |  |  |  |  |  |  |  |  |  |  |  |
| 4246_53 | 3.33 | 9226_57 | 5.00 | 15226_101 | 1.67 |  |  |  |  |  |  |  |  |  |  |  |  |
| 4247_40 | 0.00 | 9228_145 | 1.67 | 15229_186 | 10.00 |  |  |  |  |  |  |  |  |  |  |  |  |
| 4248_111 | 5.00 | 9237_66 | 0.00 | 15231_95 | 5.00 |  |  |  |  |  |  |  |  |  |  |  |  |
| 4252_81 | 1.67 | 9241_76 | 3.33 | 15233_66 | 0.00 |  |  |  |  |  |  |  |  |  |  |  |  |
| 4255_155 | 6.67 | 9242_26 | 0.00 | 15259_163 | 6.67 |  |  |  |  |  |  |  |  |  |  |  |  |
| 4257_131 | 0.00 | 9253_48 | 1.67 | 15261_146 | 5.00 |  |  |  |  |  |  |  |  |  |  |  |  |
| 4258_65 | 0.00 | 9264_103 | 0.00 | 15262_23 | 8.33 |  |  |  |  |  |  |  |  |  |  |  |  |
| 4266_177 | 5.00 | 9267_95 | 5.00 | 15266_130 | 11.67 |  |  |  |  |  |  |  |  |  |  |  |  |
| 4269_149 | 0.00 | 9269_105 | 0.00 | 15275_29 | 0.00 |  |  |  |  |  |  |  |  |  |  |  |  |
| 4271_107 | 0.00 | 9276_162 | 8.33 | 15276_22 | 11.67 |  |  |  |  |  |  |  |  |  |  |  |  |
| 4272_39 | 1.67 | 9277_11 | 5.00 | 15278_63 | 1.67 |  |  |  |  |  |  |  |  |  |  |  |  |
| 4274_186 | 11.67 | 9278_17 | 11.67 | 15282_146 | 0.00 |  |  |  |  |  |  |  |  |  |  |  |  |
| 4278_43 | 11.67 | 9279_112 | 0.00 | 15296_117 | 0.00 |  |  |  |  |  |  |  |  |  |  |  |  |
| 4280_46 | 0.00 | 9288_64 | 0.00 | 15299_35 | 3.33 |  |  |  |  |  |  |  |  |  |  |  |  |
| 4285_99 | 6.67 | 9289_80 | 8.33 | 15302_66 | 0.00 |  |  |  |  |  |  |  |  |  |  |  |  |
| 4288_144 | 11.67 | 9290_62 | 0.00 | 15306_41 | 6.67 |  |  |  |  |  |  |  |  |  |  |  |  |
| 4298_160 | 0.00 | 9291_68 | 3.33 | 15313_71 | 16.67 |  |  |  |  |  |  |  |  |  |  |  |  |
| 4299_136 | 0.00 | 9293_113 | 25.00 | 15319_34 | 8.33 |  |  |  |  |  |  |  |  |  |  |  |  |
| 4301_130 | 10.00 | 9296_136 | 0.00 | 15322_109 | 0.00 |  |  |  |  |  |  |  |  |  |  |  |  |
| 4302_67 | 3.33 | 9298_8 | 3.33 | 15323_10 | 11.67 |  |  |  |  |  |  |  |  |  |  |  |  |
| 4306_41 | 8.33 | 9300_93 | 1.67 | 15324_19 | 0.00 |  |  |  |  |  |  |  |  |  |  |  |  |
| 4308_73 | 1.67 | 9301_125 | 0.00 | 15335_21 | 6.67 |  |  |  |  |  |  |  |  |  |  |  |  |
| 4314_84 | 5.00 | 9305_25 | 5.00 | 15340_105 | 3.33 |  |  |  |  |  |  |  |  |  |  |  |  |
| 4322_60 | 5.00 | 9308_95 | 0.00 | 15341_124 | 1.67 |  |  |  |  |  |  |  |  |  |  |  |  |
| 4326_67 | 1.67 | 9309_170 | 10.00 | 15344_108 | 1.67 |  |  |  |  |  |  |  |  |  |  |  |  |
| 4327_144 | 0.00 | 9310_18 | 8.33 | 15349_185 | 3.33 |  |  |  |  |  |  |  |  |  |  |  |  |
| 4331_73 | 5.00 | 9319_148 | 0.00 | 15358_33 | 0.00 |  |  |  |  |  |  |  |  |  |  |  |  |
| 4333_99 | 3.33 | 9324_62 | 3.33 | 15361_38 | 10.00 |  |  |  |  |  |  |  |  |  |  |  |  |
| 4334_8 | 0.00 | 9326_125 | 3.33 | 15362_53 | 0.00 |  |  |  |  |  |  |  |  |  |  |  |  |
| 4344_86 | 1.67 | 9328_128 | 0.00 | 15369_100 | 0.00 |  |  |  |  |  |  |  |  |  |  |  |  |
| 4346_164 | 5.00 | 9331_59 | 0.00 | 15382_43 | 0.00 |  |  |  |  |  |  |  |  |  |  |  |  |
| 4348_144 | 5.00 | 9336_56 | 0.00 | 15383_102 | 1.67 |  |  |  |  |  |  |  |  |  |  |  |  |
| 4349_43 | 0.00 | 9338_14 | 8.33 | 15384_170 | 0.00 |  |  |  |  |  |  |  |  |  |  |  |  |
| 4352_53 | 3.33 | 9340_172 | 0.00 | 15386_124 | 1.67 |  |  |  |  |  |  |  |  |  |  |  |  |
| 4357_6 | 0.00 | 9342_46 | 18.33 | 15389_45 | 6.67 |  |  |  |  |  |  |  |  |  |  |  |  |
| 4370_104 | 8.33 | 9352_37 | 5.00 | 15392_185 | 0.00 |  |  |  |  |  |  |  |  |  |  |  |  |
| 4372_8 | 3.33 | 9357_99 | 1.67 | 15407_159 | 3.33 |  |  |  |  |  |  |  |  |  |  |  |  |
| 4373_117 | 0.00 | 9362_121 | 6.67 | 15409_65 | 3.33 |  |  |  |  |  |  |  |  |  |  |  |  |
| 4384_97 | 1.67 | 9363_145 | 8.33 | 15419_97 | 5.00 |  |  |  |  |  |  |  |  |  |  |  |  |
| 4389_155 | 13.33 | 9365_75 | 1.67 | 15434_168 | 6.67 |  |  |  |  |  |  |  |  |  |  |  |  |
| 4390_154 | 0.00 | 9372_9 | 0.00 | 15436_136 | 18.33 |  |  |  |  |  |  |  |  |  |  |  |  |
| 4392_89 | 1.67 | 9377_15 | 0.00 | 15438_25 | 5.00 |  |  |  |  |  |  |  |  |  |  |  |  |
| 4394_83 | 0.00 | 9382_23 | 0.00 | 15447_97 | 1.67 |  |  |  |  |  |  |  |  |  |  |  |  |
| 4398_5 | 1.67 | 9383_51 | 8.33 | 15457_60 | 1.67 |  |  |  |  |  |  |  |  |  |  |  |  |
| 4405_106 | 0.00 | 9400_97 | 6.67 | 15465_154 | 6.67 |  |  |  |  |  |  |  |  |  |  |  |  |
| 4407_64 | 5.00 | 9404_177 | 10.00 | 15475_96 | 8.33 |  |  |  |  |  |  |  |  |  |  |  |  |
| 4408_13 | 0.00 | 9405_77 | 3.33 | 15481_74 | 5.00 |  |  |  |  |  |  |  |  |  |  |  |  |
| 4418_35 | 3.33 | 9409_74 | 0.00 | 15497_47 | 5.00 |  |  |  |  |  |  |  |  |  |  |  |  |
| 4425_122 | 1.67 | 9410_7 | 16.67 | 15507_127 | 0.00 |  |  |  |  |  |  |  |  |  |  |  |  |
| 4427_137 | 0.00 | 9414_57 | 5.00 | 15516_123 | 3.33 |  |  |  |  |  |  |  |  |  |  |  |  |
| 4428_100 | 1.67 | 9418_51 | 0.00 | 15519_116 | 0.00 |  |  |  |  |  |  |  |  |  |  |  |  |
| 4431_102 | 3.33 | 9419_180 | 10.00 | 15523_112 | 0.00 |  |  |  |  |  |  |  |  |  |  |  |  |
| 4432_133 | 0.00 | 9422_114 | 0.00 | 15525_49 | 1.67 |  |  |  |  |  |  |  |  |  |  |  |  |
| 4434_167 | 1.67 | 9425_7 | 3.33 | 15526_114 | 1.67 |  |  |  |  |  |  |  |  |  |  |  |  |
| 4437_53 | 0.00 | 9427_57 | 8.33 | 15532_11 | 3.33 |  |  |  |  |  |  |  |  |  |  |  |  |
| 4439_48 | 1.67 | 9430_10 | 0.00 | 15545_40 | 21.67 |  |  |  |  |  |  |  |  |  |  |  |  |
| 4441_65 | 5.00 | 9438_88 | 11.67 | 15548_109 | 1.67 |  |  |  |  |  |  |  |  |  |  |  |  |
| 4443_24 | 1.67 | 9442_86 | 1.67 | 15551_76 | 0.00 |  |  |  |  |  |  |  |  |  |  |  |  |
| 4450_67 | 1.67 | 9453_24 | 0.00 | 15557_58 | 1.67 |  |  |  |  |  |  |  |  |  |  |  |  |
| 4452_97 | 0.00 | 9460_19 | 0.00 | 15562_94 | 0.00 |  |  |  |  |  |  |  |  |  |  |  |  |
| 4456_95 | 0.00 | 9461_41 | 0.00 | 15563_71 | 0.00 |  |  |  |  |  |  |  |  |  |  |  |  |
| 4457_30 | 0.00 | 9470_146 | 5.00 | 15565_70 | 0.00 |  |  |  |  |  |  |  |  |  |  |  |  |
| 4467_99 | 1.67 | 9471_8 | 1.67 | 15568_175 | 6.67 |  |  |  |  |  |  |  |  |  |  |  |  |
| 4471_20 | 5.00 | 9472_35 | 8.33 | 15569_44 | 0.00 |  |  |  |  |  |  |  |  |  |  |  |  |
| 4474_6 | 1.67 | 9473_57 | 1.67 | 15575_48 | 6.67 |  |  |  |  |  |  |  |  |  |  |  |  |
| 4475_11 | 0.00 | 9476_10 | 1.67 | 15583_108 | 0.00 |  |  |  |  |  |  |  |  |  |  |  |  |
| 4479_68 | 0.00 | 9479_66 | 0.00 | 15588_149 | 1.67 |  |  |  |  |  |  |  |  |  |  |  |  |
| 4486_9 | 0.00 | 9484_54 | 11.67 | 15595_133 | 6.67 |  |  |  |  |  |  |  |  |  |  |  |  |
| 4490_185 | 3.33 | 9489_165 | 3.33 | 15601_144 | 6.67 |  |  |  |  |  |  |  |  |  |  |  |  |
| 4491_71 | 0.00 | 9495_31 | 3.33 | 15602_74 | 8.33 |  |  |  |  |  |  |  |  |  |  |  |  |
| 4501_72 | 10.00 | 9496_43 | 0.00 | 15613_184 | 0.00 |  |  |  |  |  |  |  |  |  |  |  |  |
| 4502_18 | 0.00 | 9498_57 | 6.67 | 15617_184 | 13.33 |  |  |  |  |  |  |  |  |  |  |  |  |
| 4503_79 | 10.00 | 9501_107 | 1.67 | 15624_37 | 0.00 |  |  |  |  |  |  |  |  |  |  |  |  |
| 4507_109 | 0.00 | 9505_126 | 5.00 | 15628_126 | 0.00 |  |  |  |  |  |  |  |  |  |  |  |  |
| 4511_95 | 8.33 | 9507_23 | 1.67 | 15637_94 | 0.00 |  |  |  |  |  |  |  |  |  |  |  |  |
| 4515_39 | 18.33 | 9508_133 | 1.67 | 15639_171 | 0.00 |  |  |  |  |  |  |  |  |  |  |  |  |
| 4520_44 | 10.00 | 9509_105 | 0.00 | 15640_83 | 6.67 |  |  |  |  |  |  |  |  |  |  |  |  |
| 4521_183 | 8.33 | 9510_87 | 5.00 | 15666_12 | 3.33 |  |  |  |  |  |  |  |  |  |  |  |  |
| 4528_171 | 0.00 | 9511_68 | 0.00 | 15690_72 | 6.67 |  |  |  |  |  |  |  |  |  |  |  |  |
| 4534_92 | 0.00 | 9512_126 | 5.00 | 15771_32 | 8.33 |  |  |  |  |  |  |  |  |  |  |  |  |
| 4536_176 | 1.67 | 9517_30 | 5.00 | 15790_86 | 5.00 |  |  |  |  |  |  |  |  |  |  |  |  |
| 4539_50 | 0.00 | 9519_75 | 0.00 | 15838_88 | 3.33 |  |  |  |  |  |  |  |  |  |  |  |  |
| 4540_158 | 3.33 | 9525_45 | 0.00 | 16005_99 | 6.67 |  |  |  |  |  |  |  |  |  |  |  |  |
| 4541_137 | 1.67 | 9526_105 | 1.67 | 16109_98 | 8.33 |  |  |  |  |  |  |  |  |  |  |  |  |
| 4544_91 | 0.00 | 9531_53 | 1.67 | 16180_160 | 6.67 |  |  |  |  |  |  |  |  |  |  |  |  |
| 4545_128 | 6.67 | 9536_95 | 0.00 | 16199_80 | 5.00 |  |  |  |  |  |  |  |  |  |  |  |  |
| 4548_120 | 0.00 | 9539_142 | 0.00 | 16224_17 | 10.00 |  |  |  |  |  |  |  |  |  |  |  |  |
| 4559_160 | 6.67 | 9541_11 | 0.00 | 16233_81 | 5.00 |  |  |  |  |  |  |  |  |  |  |  |  |
| 4563_55 | 3.33 | 9542_131 | 0.00 | 16252_70 | 8.33 |  |  |  |  |  |  |  |  |  |  |  |  |
| 4566_85 | 0.00 | 9544_7 | 3.33 | 16304_88 | 1.67 |  |  |  |  |  |  |  |  |  |  |  |  |
| 4574_129 | 0.00 | 9550_163 | 5.00 | 16359_148 | 11.67 |  |  |  |  |  |  |  |  |  |  |  |  |
| 4581_60 | 0.00 | 9552_55 | 1.67 | 16430_7 | 5.00 |  |  |  |  |  |  |  |  |  |  |  |  |
| 4583_109 | 0.00 | 9555_169 | 1.67 | 16459_59 | 10.00 |  |  |  |  |  |  |  |  |  |  |  |  |
| 4596_177 | 5.00 | 9567_71 | 0.00 | 16505_99 | 15.00 |  |  |  |  |  |  |  |  |  |  |  |  |
| 4598_35 | 0.00 | 9568_95 | 1.67 | 16515_65 | 8.33 |  |  |  |  |  |  |  |  |  |  |  |  |
| 4600_37 | 1.67 | 9573_83 | 3.33 | 16702_18 | 6.67 |  |  |  |  |  |  |  |  |  |  |  |  |
| 4603_122 | 1.67 | 9576_12 | 6.67 | 16731_118 | 8.33 |  |  |  |  |  |  |  |  |  |  |  |  |
| 4604_10 | 6.67 | 9578_100 | 1.67 | 16814_120 | 10.00 |  |  |  |  |  |  |  |  |  |  |  |  |
| 4611_31 | 8.33 | 9581_138 | 8.33 | 16884_13 | 23.33 |  |  |  |  |  |  |  |  |  |  |  |  |
| 4613_104 | 8.33 | 9584_43 | 0.00 | 16900_70 | 11.67 |  |  |  |  |  |  |  |  |  |  |  |  |
| 4622_128 | 0.00 | 9585_28 | 8.33 | 16915_31 | 11.67 |  |  |  |  |  |  |  |  |  |  |  |  |
| 4623_56 | 0.00 | 9597_31 | 5.00 | 16988_64 | 13.33 |  |  |  |  |  |  |  |  |  |  |  |  |
| 4624_34 | 5.00 | 9598_99 | 0.00 | 17020_46 | 18.33 |  |  |  |  |  |  |  |  |  |  |  |  |
| 4627_186 | 6.67 | 9599_23 | 5.00 | 17035_125 | 3.33 |  |  |  |  |  |  |  |  |  |  |  |  |
| 4629_101 | 0.00 | 9604_107 | 3.33 | 17052_106 | 13.33 |  |  |  |  |  |  |  |  |  |  |  |  |
| 4631_152 | 10.00 | 9609_70 | 3.33 | 17063_39 | 10.00 |  |  |  |  |  |  |  |  |  |  |  |  |
| 4633_207 | 10.00 | 9611_59 | 1.67 | 17074_72 | 3.33 |  |  |  |  |  |  |  |  |  |  |  |  |
| 4639_130 | 0.00 | 9617_73 | 6.67 | 17083_54 | 15.00 |  |  |  |  |  |  |  |  |  |  |  |  |
| 4642_83 | 11.67 | 9619_71 | 3.33 | 17085_63 | 5.00 |  |  |  |  |  |  |  |  |  |  |  |  |
| 4646_79 | 3.33 | 9621_66 | 5.00 | 17088_59 | 13.33 |  |  |  |  |  |  |  |  |  |  |  |  |
| 4650_134 | 6.67 | 9623_61 | 5.00 | 17131_67 | 6.67 |  |  |  |  |  |  |  |  |  |  |  |  |
| 4655_25 | 8.33 | 9624_195 | 5.00 | 17204_106 | 8.33 |  |  |  |  |  |  |  |  |  |  |  |  |
| 4668_88 | 1.67 | 9633_95 | 3.33 | 17282_67 | 13.33 |  |  |  |  |  |  |  |  |  |  |  |  |
| 4669_46 | 1.67 | 9635_100 | 6.67 | 17358_192 | 16.67 |  |  |  |  |  |  |  |  |  |  |  |  |
| 4677_96 | 8.33 | 9636_88 | 0.00 | 17440_85 | 10.00 |  |  |  |  |  |  |  |  |  |  |  |  |
| 4681_112 | 1.67 | 9640_59 | 3.33 | 17458_50 | 3.33 |  |  |  |  |  |  |  |  |  |  |  |  |
| 4682_51 | 0.00 | 9645_45 | 3.33 | 17529_43 | 11.67 |  |  |  |  |  |  |  |  |  |  |  |  |
| 4683_77 | 0.00 | 9647_65 | 1.67 | 17623_112 | 8.33 |  |  |  |  |  |  |  |  |  |  |  |  |
| 4690_98 | 6.67 | 9648_22 | 5.00 | 17638_119 | 10.00 |  |  |  |  |  |  |  |  |  |  |  |  |
| 4693_5 | 1.67 | 9651_136 | 5.00 | 17659_147 | 8.33 |  |  |  |  |  |  |  |  |  |  |  |  |
| 4701_46 | 0.00 | 9652_109 | 0.00 | 17789_64 | 18.33 |  |  |  |  |  |  |  |  |  |  |  |  |
| 4705_41 | 6.67 | 9654_75 | 1.67 | 17795_136 | 8.33 |  |  |  |  |  |  |  |  |  |  |  |  |
| 4711_95 | 3.33 | 9662_11 | 0.00 | 17799_89 | 11.67 |  |  |  |  |  |  |  |  |  |  |  |  |
| 4717_38 | 3.33 | 9664_165 | 0.00 | 17808_119 | 18.33 |  |  |  |  |  |  |  |  |  |  |  |  |
| 4723_36 | 3.33 | 9668_9 | 3.33 | 17855_85 | 6.67 |  |  |  |  |  |  |  |  |  |  |  |  |
| 4729_170 | 0.00 | 9669_10 | 15.00 | 17868_108 | 8.33 |  |  |  |  |  |  |  |  |  |  |  |  |
| 4730_45 | 13.33 | 9672_158 | 0.00 | 17870_91 | 8.33 |  |  |  |  |  |  |  |  |  |  |  |  |
| 4733_66 | 5.00 | 9676_100 | 1.67 | 17873_113 | 11.67 |  |  |  |  |  |  |  |  |  |  |  |  |
| 4735_20 | 0.00 | 9682_84 | 1.67 | 18002_205 | 15.00 |  |  |  |  |  |  |  |  |  |  |  |  |
| 4737_10 | 6.67 | 9683_27 | 1.67 | 18461_90 | 10.00 |  |  |  |  |  |  |  |  |  |  |  |  |
| 4741_74 | 0.00 | 9684_97 | 3.33 | 18785_110 | 11.67 |  |  |  |  |  |  |  |  |  |  |  |  |
| 4746_96 | 1.67 | 9687_96 | 0.00 | 19004_30 | 8.33 |  |  |  |  |  |  |  |  |  |  |  |  |
| 4750_57 | 0.00 | 9695_123 | 10.00 | 27864_114 | 0.00 |  |  |  |  |  |  |  |  |  |  |  |  |
| 4752_89 | 0.00 | 9696_65 | 1.67 | 46788_60 | 1.67 |  |  |  |  |  |  |  |  |  |  |  |  |
| 4755_86 | 5.00 | 9701_39 | 10.00 | 48066_67 | 1.67 |  |  |  |  |  |  |  |  |  |  |  |  |
| 4758_190 | 1.67 | 9702_113 | 8.33 | 48165_156 | 3.33 |  |  |  |  |  |  |  |  |  |  |  |  |
| 4764_170 | 15.00 | 9703_38 | 3.33 | 52488_83 | 6.67 |  |  |  |  |  |  |  |  |  |  |  |  |
| 4765_102 | 0.00 | 9705_127 | 1.67 | 52584_92 | 3.33 |  |  |  |  |  |  |  |  |  |  |  |  |
| 4766_102 | 6.67 | 9707_88 | 0.00 | 56414_95 | 20.00 |  |  |  |  |  |  |  |  |  |  |  |  |
| 4768_14 | 5.00 | 9709_149 | 11.67 | 57688_42 | 8.33 |  |  |  |  |  |  |  |  |  |  |  |  |
| 4770_62 | 0.00 | 9712_56 | 3.33 | 59595_15 | 8.33 |  |  |  |  |  |  |  |  |  |  |  |  |
| 4771_79 | 1.67 | 9716_107 | 13.33 | 59857_36 | 5.00 |  |  |  |  |  |  |  |  |  |  |  |  |
| 4772_35 | 13.33 | 9718_23 | 1.67 | 64248_101 | 1.67 |  |  |  |  |  |  |  |  |  |  |  |  |
| 4773_56 | 1.67 | 9729_183 | 5.00 | 65447_157 | 0.00 |  |  |  |  |  |  |  |  |  |  |  |  |
| 4774_42 | 0.00 | 9734_119 | 1.67 | 72819_229 | 10.00 |  |  |  |  |  |  |  |  |  |  |  |  |
| 4778_84 | 0.00 | 9736_138 | 3.33 | 83869_25 | 3.33 |  |  |  |  |  |  |  |  |  |  |  |  |
| 4779_63 | 1.67 | 9744_18 | 0.00 | 85489_48 | 16.67 |  |  |  |  |  |  |  |  |  |  |  |  |
| 4786_103 | 1.67 | 9745_55 | 10.00 | 97241_126 | 3.33 |  |  |  |  |  |  |  |  |  |  |  |  |
| 4789_32 | 0.00 | 9746_119 | 6.67 | 100159_34 | 10.00 |  |  |  |  |  |  |  |  |  |  |  |  |
|  |  |  |  | 104333_82 | 3.33 |  |  |  |  |  |  |  |  |  |  |  |  |

**Table S4.** Pairwise values of Φst for *MT-CYB* between the different localities of *C. paludica* and *C. vettonica*. Only significant values are shown (*p-value* < 0.05). Red cells: Φst>0.75; Orange: 0.5>Φst <0.75; Yellow: 0.25>Φst<0.5; Green: Φst<0.25. The localities where mitochondrial haplotypes of *C. vettonica* and *C. paludica* were detected, they were studied for each species separately (_V and _P respectively).

|  | Ponsul | Aravil_V | High Erjas | SanMartin | Trevejana | Middle Erjas | Lower Erjas_V | Arades | Arrago | Gata | Acebo | Mayas | Alagon_V | Alagon_P | Francia_V |
| --- | --- | --- | --- | --- | --- | --- | --- | --- | --- | --- | --- | --- | --- | --- | --- |
| Ponsul |  |  |  |  |  |  |  |  |  |  |  |  |  |  |  |
| Aravil_V |  |  |  |  |  |  |  |  |  |  |  |  |  |  |  |
| High Erjas |  |  |  |  |  |  |  |  |  |  |  |  |  |  |  |
| San Martin |  |  |  |  |  |  |  |  |  |  |  |  |  |  |  |
| Trevejana |  |  |  |  |  |  |  |  |  |  |  |  |  |  |  |
| Middle Erjas |  |  | 0.2 |  |  |  |  |  |  |  |  |  |  |  |  |
| Lower Erjas_V |  |  |  |  |  |  |  |  |  |  |  |  |  |  |  |
| Arades |  |  |  |  |  |  |  |  |  |  |  |  |  |  |  |
| Árrago | 0.96 | 0.97 | 0.96 | 0.96 | 0.96 | 0.93 | 0.92 | 0.97 |  |  |  |  |  |  |  |
| Gata | 0.91 | 0.92 | 0.93 | 0.92 | 0.91 | 0.9 | 0.88 | 0.91 | 0.25 |  |  |  |  |  |  |
| Acebo | 0.95 | 0.98 | 0.97 | 0.96 | 0.98 | 0.9 | 0.87 |  | 0.74 |  |  |  |  |  |  |
| Mayas | 0.99 | 0.99 | 0.98 | 0.99 | 0.99 | 0.97 | 0.95 | 1 |  | 0.34 | 1 |  |  |  |  |
| Alagon_V | 0.9 | 0.92 | 0.94 | 0.92 | 0.91 | 0.88 | 0.87 | 0.91 | 0.81 | 0.73 | 0.78 | 0.91 |  |  |  |
| Alagon_P | 0.97 | 0.98 | 0.98 | 0.97 | 0.97 | 0.95 | 0.94 | 0.97 | 0.98 | 0.96 | 0.97 | 0.99 | 0.96 |  |  |
| Francia_V | 0.96 | 0.98 | 0.97 | 0.97 | 0.97 | 0.93 | 0.9 | 0.98 | 0.9 | 0.79 | 0.95 | 0.98 |  | 0.98 |  |
| Francia_P | 0.96 | 0.97 | 0.98 | 0.97 | 0.97 | 0.95 | 0.94 | 0.97 | 0.98 | 0.95 | 0.97 | 0.99 | 0.95 |  | 0.98 |
| Cdh_V | 0.96 | 0.97 | 0.96 | 0.96 | 0.97 | 0.93 | 0.91 | 0.97 | 0.89 | 0.79 | 0.93 | 0.97 |  | 0.98 |  |
| Cdh_P | 0.96 | 0.97 | 0.97 | 0.97 | 0.97 | 0.95 | 0.94 | 0.96 | 0.97 | 0.95 | 0.96 | 0.99 | 0.95 |  | 0.97 |
| Caparro_V | 0.97 | 0.99 | 0.97 | 0.97 | 0.98 | 0.93 | 0.9 | 1 | 0.91 | 0.78 | 1 | 1 |  | 0.98 |  |
| Caparro_P | 0.98 | 0.98 | 0.98 | 0.98 | 0.98 | 0.97 | 0.96 | 0.98 | 0.98 | 0.97 | 0.98 | 0.99 | 0.97 |  | 0.98 |
| Jerte_V | 0.92 | 0.94 | 0.94 | 0.93 | 0.93 | 0.89 | 0.88 | 0.93 | 0.8 | 0.69 | 0.8 | 0.9 |  | 0.96 |  |
| Jerte_P | 0.97 | 0.98 | 0.98 | 0.97 | 0.97 | 0.95 | 0.95 | 0.97 | 0.98 | 0.96 | 0.98 | 0.99 | 0.96 | 0.51 | 0.98 |
| Lower Erjas_P | 0.95 | 0.97 | 0.97 | 0.96 | 0.96 | 0.93 | 0.93 |  | 0.98 | 0.95 |  | 0.99 | 0.94 |  | 0.98 |
| Aurela | 0.94 | 0.96 | 0.97 | 0.96 | 0.95 | 0.93 | 0.93 | 0.94 | 0.97 | 0.95 | 0.95 | 0.99 | 0.94 |  | 0.97 |
| Aravil_P |  | 0.97 | 0.98 | 0.97 |  | 0.93 | 0.93 |  | 0.98 | 0.95 |  | 1 | 0.94 |  | 0.98 |
| Salor |  |  |  |  |  |  |  |  | 0.82 | 0.79 |  | 0.89 | 0.68 |  | 0.75 |
| Tamuja | 0.94 | 0.95 | 0.97 | 0.95 | 0.95 | 0.93 | 0.92 |  | 0.97 | 0.95 | 0.94 | 0.99 | 0.94 |  | 0.97 |
| Almonte | 0.85 | 0.88 | 0.92 | 0.88 | 0.86 | 0.86 | 0.87 | 0.82 | 0.93 | 0.91 | 0.84 | 0.96 | 0.87 | 0.32 | 0.9 |
| Ibor | 0.91 | 0.94 | 0.96 | 0.94 | 0.93 | 0.91 | 0.91 |  | 0.96 | 0.94 |  | 0.98 | 0.92 | 0.65 | 0.95 |
| Tietar | 0.88 | 0.89 | 0.92 | 0.9 | 0.89 | 0.89 | 0.89 | 0.87 | 0.92 | 0.91 | 0.86 | 0.94 | 0.89 | 0.55 | 0.9 |
| Yeltes | 0.98 | 0.99 | 0.99 | 0.99 | 0.99 | 0.96 | 0.95 | 1 | 0.99 | 0.96 | 1 | 1 | 0.97 | 0.79 | 0.99 |
| Huebra | 0.96 | 0.97 | 0.97 | 0.96 | 0.96 | 0.95 | 0.94 | 0.96 | 0.97 | 0.95 | 0.96 | 0.99 | 0.95 | 0.35 | 0.97 |

|  | Francia_P | Cdh_V | Cdh_P | Caparro_V | Caparro_P | Jerte_V | Jerte_P | Lower Erjas_P | Aurela | Aravil_P | Salor | Tamuja | Almonte | Ibor | Tietar | Yeltes |
| --- | --- | --- | --- | --- | --- | --- | --- | --- | --- | --- | --- | --- | --- | --- | --- | --- |
| Ponsul |  |  |  |  |  |  |  |  |  |  |  |  |  |  |  |  |
| Aravil_V |  |  |  |  |  |  |  |  |  |  |  |  |  |  |  |  |
| High Erjas |  |  |  |  |  |  |  |  |  |  |  |  |  |  |  |  |
| San Martin |  |  |  |  |  |  |  |  |  |  |  |  |  |  |  |  |
| Trevejana |  |  |  |  |  |  |  |  |  |  |  |  |  |  |  |  |
| Middle Erjas |  |  |  |  |  |  |  |  |  |  |  |  |  |  |  |  |
| Lower Erjas_V |  |  |  |  |  |  |  |  |  |  |  |  |  |  |  |  |
| Arades |  |  |  |  |  |  |  |  |  |  |  |  |  |  |  |  |
| Árrago |  |  |  |  |  |  |  |  |  |  |  |  |  |  |  |  |
| Gata |  |  |  |  |  |  |  |  |  |  |  |  |  |  |  |  |
| Acebo |  |  |  |  |  |  |  |  |  |  |  |  |  |  |  |  |
| Mayas |  |  |  |  |  |  |  |  |  |  |  |  |  |  |  |  |
| Alagon_V |  |  |  |  |  |  |  |  |  |  |  |  |  |  |  |  |
| Alagon_P |  |  |  |  |  |  |  |  |  |  |  |  |  |  |  |  |
| Francia_V |  |  |  |  |  |  |  |  |  |  |  |  |  |  |  |  |
| Francia_P |  |  |  |  |  |  |  |  |  |  |  |  |  |  |  |  |
| Cdh_V | 0.98 |  |  |  |  |  |  |  |  |  |  |  |  |  |  |  |
| Cdh_P |  | 0.97 |  |  |  |  |  |  |  |  |  |  |  |  |  |  |
| Caparro_V | 0.98 |  | 0.97 |  |  |  |  |  |  |  |  |  |  |  |  |  |
| Caparro_P |  | 0.98 | 0.43 | 0.98 |  |  |  |  |  |  |  |  |  |  |  |  |
| Jerte_V | 0.96 |  | 0.96 |  | 0.97 |  |  |  |  |  |  |  |  |  |  |  |
| Jerte_P | 0.47 | 0.98 |  | 0.98 | 0.71 | 0.97 |  |  |  |  |  |  |  |  |  |  |
| Lower Erjas_P |  | 0.98 |  | 0.98 | 0.38 | 0.95 |  |  |  |  |  |  |  |  |  |  |
| Aurela |  | 0.97 |  | 0.97 | 0.28 | 0.95 |  |  |  |  |  |  |  |  |  |  |
| Aravil_P |  | 0.98 |  | 0.99 | 0.47 | 0.96 |  |  |  |  |  |  |  |  |  |  |
| Salor |  | 0.79 |  | 0.7 |  | 0.71 |  |  |  |  |  |  |  |  |  |  |
| Tamuja |  | 0.97 | 0.34 | 0.97 | 0.37 | 0.95 | 0.57 |  |  |  |  |  |  |  |  |  |
| Almonte | 0.29 | 0.91 | 0.39 | 0.89 | 0.53 | 0.89 | 0.48 |  |  |  |  |  |  |  |  |  |
| Ibor | 0.62 | 0.96 | 0.67 | 0.95 | 0.79 | 0.93 | 0.73 |  | 0.5 |  |  | 0.43 |  |  |  |  |
| Tietar | 0.54 | 0.91 | 0.59 | 0.9 | 0.68 | 0.89 | 0.63 | 0.47 | 0.48 |  | 0.55 | 0.44 | 0.48 |  |  |  |
| Yeltes | 0.78 | 0.99 | 0.75 | 1 | 0.84 | 0.98 | 0.87 | 0.76 | 0.67 | 0.86 | 0.52 | 0.58 | 0.43 | 0.68 | 0.54 |  |
| Huebra | 0.34 | 0.97 | 0.44 | 0.97 | 0.52 | 0.96 | 0.66 |  | 0.3 |  |  |  | 0.36 | 0.58 | 0.53 | 0.65 |

**Table S5.** Results of the Puechmaille method and the ΔK method for different values of K, considering the populations of both *C. paludica* and *C. vettonica* and only those of *C. vettonica*.

|  | **Puechmaille method** | | | | | | **ΔK method** | | | | |
| --- | --- | --- | --- | --- | --- | --- | --- | --- | --- | --- | --- |
|  | **K** | **Reps** | **MedMed** | **MedMean** | **MaxMed** | **MaxMean** | **Mean LnP(K)** | **Stdev LnP(K)** | **Ln'(K)** | **\|Ln''(K)\|** | **ΔK** |
| *C. vettonica* + *C. paludica* | 1 | 10 | 1 | 1 | 1 | 1 | -417450.72 | 9.30266 | NA | NA | NA |
|  | 2 | 10 | 2 | 2 | 2 | 2 | -298911.31 | 16.96797 | 118539.41 | 87652.43 | 5165.75725 |
|  | 3 | 10 | 3 | 3 | 3 | 3 | -268024.33 | 753.95264 | 30886.98 | 9514.24 | 12.61915 |
|  | 4 | 10 | 4 | 4 | 4 | 4 | -246651.59 | 37.93636 | 21372.74 | 15762.43 | 415.4966 |
|  | 5 | 10 | 5 | 5 | 5 | 5 | -241041.28 | 2477.0614 | 5610.31 | 2311.93 | 0.93334 |
|  | 6 | 10 | 5 | 5 | 6 | 6 | -237742.9 | 4678.81323 | 3298.38 | 1109158.87 | 237.05987 |
|  | 7 | 10 | 5 | 5 | 6 | 6 | -1343603.39 | 3506856.566 | -1105860.49 | 173280.61 | 0.04941 |
|  | 8 | 10 | 5 | 5 | 6 | 6 | -2622744.49 | 4154430.413 | -1279141.1 | 3656018.8 | 0.88003 |
|  | 9 | 10 | 5 | 5 | 6 | 6 | -245866.79 | 19857.88545 | 2376877.7 | 3896356.19 | 196.21204 |
|  | 10 | 10 | 5 | 5 | 5 | 5 | -1765345.28 | 3516514.633 | -1519478.49 | 1506164.7 | 0.42831 |
|  | 11 | 10 | 5 | 5 | 6 | 6 | -1778659.07 | 3585999.984 | -13313.79 | 128319.03 | 0.03578 |
|  | 12 | 10 | 5 | 5 | 6 | 6 | -1663653.83 | 3155816.949 | 115005.24 | 1970965.35 | 0.62455 |
|  | 13 | 10 | 5 | 5 | 6 | 6 | -3519613.94 | 5374467.812 | -1855960.11 | 794401.5 | 0.14781 |
|  | 14 | 10 | 5 | 5 | 6 | 6 | -4581172.55 | 6299608.254 | -1061558.61 | 2964726.8 | 0.47062 |
|  | 15 | 10 | 6 | 6 | 6 | 6 | -2678004.36 | 4373326.803 | 1903168.19 | 2501014.89 | 0.57188 |
|  | 16 | 10 | 6 | 6 | 6 | 6 | -3275851.06 | 4781295.799 | -597846.7 | 207757.56 | 0.04345 |
|  | 17 | 10 | 5 | 5 | 6 | 6 | -3665940.2 | 4641233.514 | -390089.14 | NA | NA |
|  |  |  | MedMedK=6 | MedMeaK=6 | MaxMedK=6 | MaxMeaK=6 |  |  |  |  |  |
| *C. vettonica* | 1 | 10 | 1 | 1 | 1 | 1 | -196871.89 | 12.34706 | NA | NA | NA |
|  | 2 | 10 | 2 | 2 | 2 | 2 | -156750.04 | 12.51623 | 40121.85 | 17995.29 | 1437.75648 |
|  | 3 | 10 | 3 | 3 | 3 | 3 | -134623.48 | 11.38194 | 22126.56 | 14563.76 | 1279.55045 |
|  | 4 | 10 | 4 | 4 | 4 | 4 | -127060.68 | 26.12401 | 7562.8 | 22852.53 | 874.77107 |
|  | 5 | 10 | 4 | 4 | 4 | 4 | -142350.41 | 50523.35434 | -15289.73 | 15132.98 | 0.29952 |
|  | 6 | 10 | 4 | 4 | 4 | 4 | -142507.16 | 35675.63309 | -156.75 | 27144.86 | 0.76088 |
|  | 7 | 10 | 4 | 4 | 4 | 4 | -169808.77 | 69819.26493 | -27301.61 | 71075.97 | 1.018 |
|  | 8 | 10 | 4 | 4 | 4 | 4 | -126034.41 | 448.90798 | 43774.36 | 66656.78 | 148.48651 |
|  | 9 | 10 | 4 | 4 | 4 | 4 | -148916.83 | 44654.20503 | -22882.42 | NA | NA |
|  |  |  | MedMedK=4 | MedMeaK=4 | MaxMedK=4 | MaxMeaK=4 |  |  |  |  |  |
